# Discovery of a Novel Potent and Selective HSD17B13 Inhibitor, BI-3231, a Well-Characterized Chemical Probe Available for Open Science

**DOI:** 10.1021/acs.jmedchem.2c01884

**Published:** 2023-02-02

**Authors:** Sven Thamm, Marina K. Willwacher, Gary E. Aspnes, Tom Bretschneider, Nicholas F. Brown, Silke Buschbom-Helmke, Thomas Fox, Emanuele M. Gargano, Daniel Grabowski, Christoph Hoenke, Damian Matera, Katja Mueck, Stefan Peters, Sophia Reindl, Doris Riether, Matthias Schmid, Christofer S. Tautermann, Aaron M. Teitelbaum, Cornelius Trünkle, Thomas Veser, Martin Winter, Lars Wortmann

**Affiliations:** † Boehringer Ingelheim Pharma GmbH & Co. KG, 88397 Biberach an der Riß, Germany; ‡ Boehringer Ingelheim Pharmaceuticals, Inc., 900 Ridgebury Road, PO Box 368, Ridgefield, Connecticut 06877-0368, United States

## Abstract

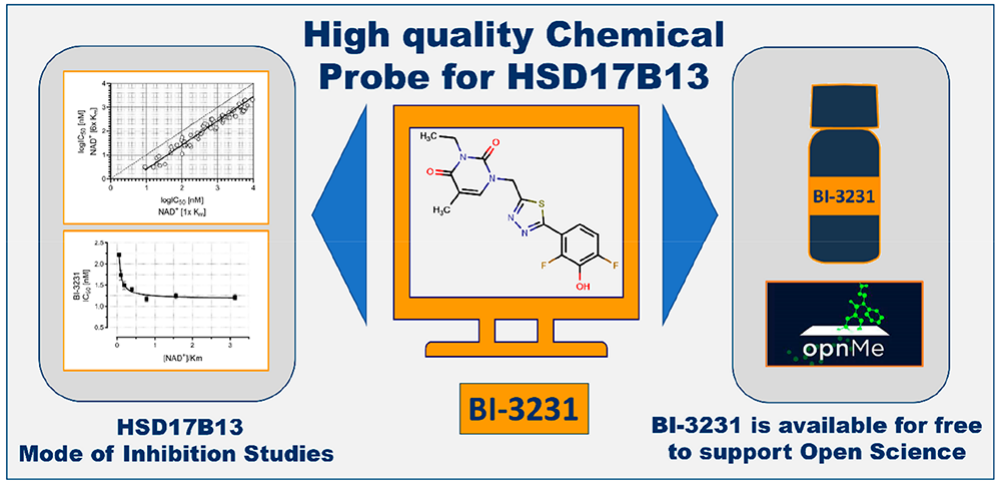

Genome-wide association studies in patients revealed HSD17B13 as a potential new target for the treatment of nonalcoholic steatohepatitis (NASH) and other liver diseases. However, the physiological function and the disease-relevant substrate of HSD17B13 remain unknown. In addition, no suitable chemical probe for HSD17B13 has been published yet. Herein, we report the identification of the novel potent and selective HSD17B13 inhibitor **BI-3231**. Through high-throughput screening (HTS), using estradiol as substrate, compound **1** was identified and selected for subsequent optimization resulting in compound **45 (BI-3231)**. In addition to the characterization of compound **45** for its functional, physicochemical, and drug metabolism and pharmacokinetic (DMPK) properties, NAD^+^ dependency was investigated. To support Open Science, the chemical HSD17B13 probe **BI-3231** will be available to the scientific community for free via the opnMe platform, and thus can help to elucidate the pharmacology of HSD17B13.

## Introduction

The unrelenting rise in the worldwide prevalence of obesity, metabolic syndrome and Type 2 diabetes^1,2^ engenders an increasing burden of associated complications and co-morbidities including cardiovascular disease, nephropathy, neuropathy, retinopathies and nonalcoholic fatty liver disease (NAFLD).^3,4^ The increased incidence of NAFLD that may progress to nonalcoholic steatohepatitis (NASH) and cirrhosis represents a looming critical burden on clinical and economic resources.^3^ Presenting initially as a silent accumulation of neutral lipids in the liver, disease progression is characterized by development of severe hepatic inflammation and advancing fibrosis, with an elevated risk of hepatocellular carcinoma (HCC) and ultimate loss of liver function (end-stage liver disease, ESLD).^5^ Currently, liver transplant is the only option for patients with ESLD.

Thus, there is a compelling interest in identifying novel drug targets that may lead to more widely applicable pharmacological therapies to halt or reverse liver disease progression. One of these potential drug targets is HSD17B13 (hydroxysteroid 17ß-dehydrogenase 13), a lipid-droplet associated member of the family of 17ß-hydroxysteroid dehydrogenases (HSD17B), that collectively acts on a range of lipid substrates.^6^

A link between HSD17B13 and liver disease was first indicated by genome-wide association studies (GWAS) that revealed a strong association between a loss-of-function (LoF) SNP rs72613567 and levels of serum alanine aminotransferase (ALT), a clinical biomarker of liver dysfunction.^7^ The initial observation has been reinforced by multiple studies of diverse cohorts, demonstrating an association between this and other LoF SNPs and risk for NASH, alcoholic liver disease, cirrhosis, and hepatocellular carcinoma.^6−16^ Primarily expressed in hepatocytes,^17^ HSD17B13 is upregulated in the liver of NAFLD patients^18^ and, preclinically, AAV-mediated HSD17B13 overexpression in mouse liver promoted lipid accumulation, indicating a strong association of HSD17B13 with fatty acid metabolism.^18^ Direct clinical support for HSD17B13 as a therapeutic target was generated when a hepatocyte-directed small interfering RNA (siRNA) designed to deplete HSD17B13 in human liver was found to decrease serum alanine aminotransferase (ALT) activity in five patients with presumed NAFLD.^19^

However, both the physiological function and the disease-relevant substrate of this enzyme are still unclear. Several substrates were identified, including steroids (e.g., estradiol) and other bioactive lipids (e.g., leukotriene B_4_), using an *in vitro* enzyme assay system in which NAD^+^ (nicotinamide adenine dinucleotide, oxidized form) acted as co-substrate.^7^ Thus, numerous lines of evidence suggest HSD17B13 is a promising target for pharmacological treatment of NASH. The lack of well-characterized small-molecule HSD17B13 modulators in the literature triggered our discovery efforts toward the identification and optimization of potent and selective small-molecule inhibitors, described in the present study.

## Results and Discussion

### Substrate Selection for High-Throughput Screening

**Figure 1:**
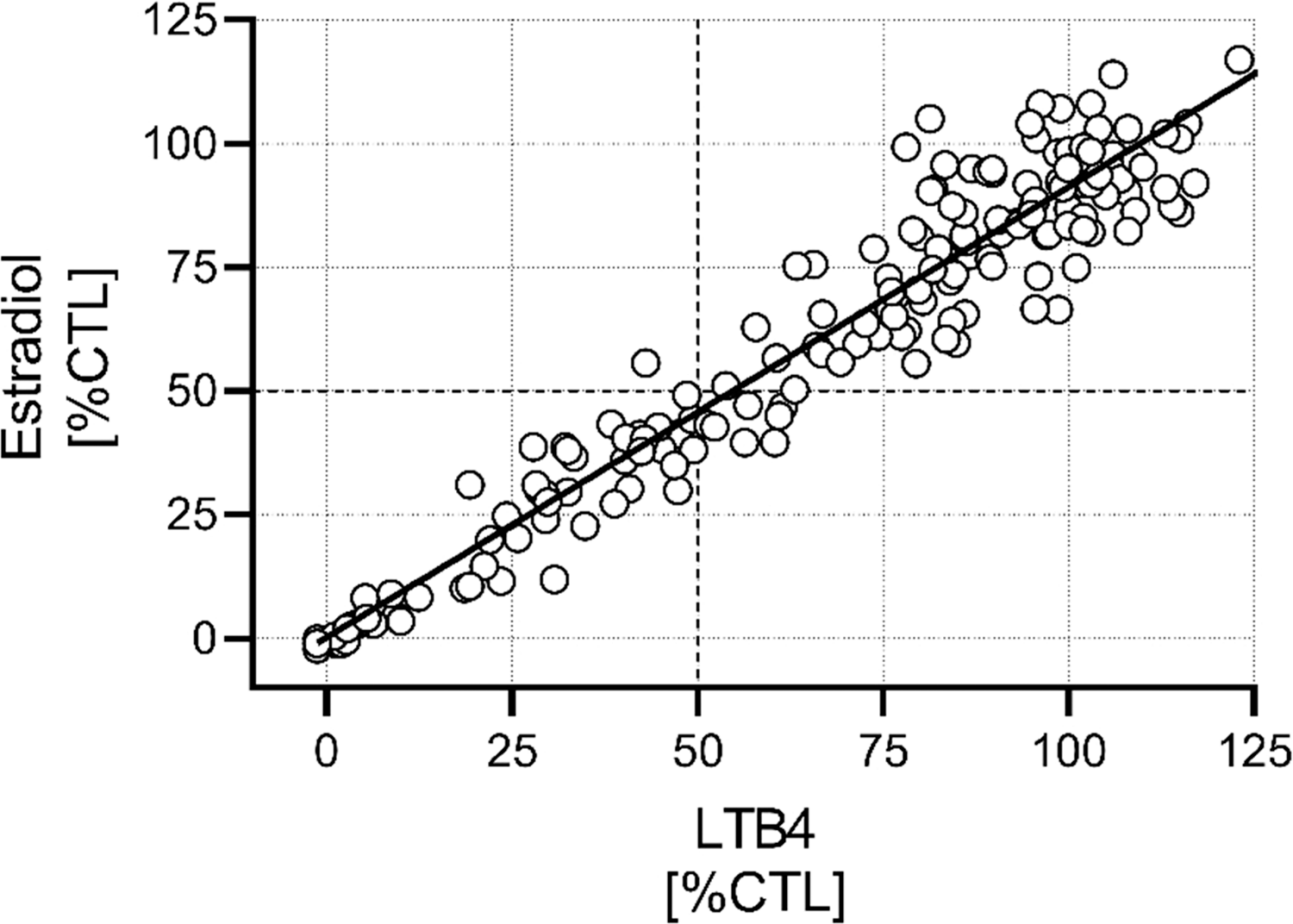
Evaluation of the risk for substrate-biased hits. Percent of control values (%CTL) of a diverse set of 175 compounds assayed at 10 μM in the human HSD17B13 enzyme assay using LTB_4_ or estradiol as substrates (Pearson *r* = 0.93; linear regression (*r*^2^ = 0.87) indicated by solid line).

The disease-relevant substrate of HSD17B13 is unknown, but approaches for the identification of HSD17B13 inhibitors using purified enzyme and known substrates were recently published.^20^ To evaluate a potential risk of substrate-biased inhibitors,^21−23^ we tested a small subset of compounds predictive for our full-diversity library, using purified human HSD17B13 enzyme and estradiol or leukotriene B_4_ (LTB_4_) as substrate in the presence of NAD^+^. We obtained a strong correlation between LTB_4_ and estradiol %CTL values at 10 μM compound concentration (Figure 1). Based on these results, we concluded the absence of a substrate bias and selected estradiol as substrate for the high-throughput screening campaign, due to its advantages in handling.

### High-Throughput Screening and Profiling of Screening Hit **1**

**Figure 2:**
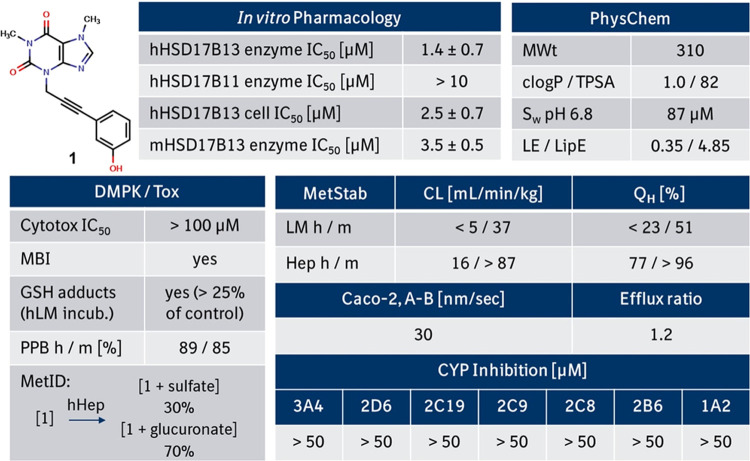
*In vitro* pharmacological, drug metabolism and pharmacokinetic (DMPK), and physicochemical properties of compound **1**. Metabolic clearance values are upscaled from *in vitro* assays to reflect the *in vivo* situation. Abbreviations are described at the end of the manuscript.

**Figure 3:**
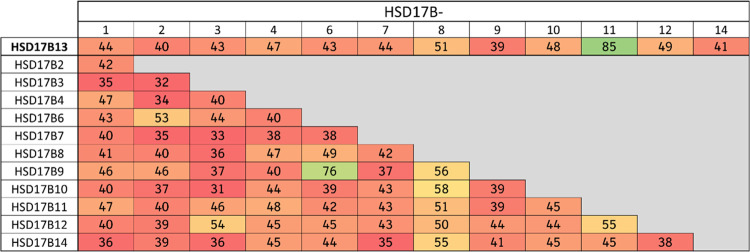
Sequence similarities (calculated using the alignment tool in MOE^36^) in % between HSD17B13 and other members of the short-chain dehydrogenase/reductase (SDR) family indicate HSD17B11 as the closest homolog.

For the discovery of small-molecule inhibitors of HSD17B13, we screened ∼1.1 million compounds from Boehringer Ingelheim’s full-diversity library against the enzymatic activity of human HSD17B13 in the presence of estradiol and NAD^+^ on our fully automated, high-throughput screening compatible matrix-assisted laser desorption ionization–time-of-flight mass spectrometry (MALDI-TOF-MS) platform^24−26^ (see Supporting Information, Figure S1). Beyond well-known steroid-like and steroid-derived inhibitors of HSD17B13,^27^ we identified and confirmed a phenol cluster, and selected alkynyl phenol **1** with an IC_50_ value of 1.4 μM as a starting point for further evaluation. Notably, other examples of phenol-derived inhibitors of HSD17B13 were reported in the recent patent literature.^28−34^ To identify potential liabilities of screening hit **1**, we thoroughly profiled this compound in several *in vitro* assays (Figure 2). Compound **1** revealed a moderate activity in the enzymatic human and mouse HSD17B13 enzymatic assays with good selectivity versus the phylogenetically closest related isoform HSD17B11 (Figure 3) and showed moderate activity in the human HSD17B13 cellular assay. We also tested compound **1** in the presence of retinol instead of estradiol in the human HSD17B13 enzymatic assay and again confirmed the absence of a substrate bias (IC_50,retinol_ = 2.4 ± 0.1 μM vs IC_50,estradiol_ = 1.4 ± 0.7 μM). In addition, **1** showed a good balance between solubility and lipophilicity, high permeability, and no inhibition of cytochrome P450 enzymes. While **1** demonstrated a high metabolic stability in liver microsomes, low metabolic stability in hepatocytes pointed toward a significant contribution of phase II metabolism. Metabolite identification of phenol **1** confirmed a strong phase II metabolism,^35^ leading to 70% glucuronidation and 30% sulfation of the parent phenol **1** after incubation with human hepatocytes (Figure 2).

### Structure–Activity Relationship (SAR) Investigations and Optimization of Screening Hit **1**

We began our hit optimization by addressing the identified liabilities of the strong phase II metabolism and reactive metabolite formation of screening hit **1**.^35^ As phase II metabolism is a well-known liability for phenols,^37,38^ we envisaged to replace this moiety with a variety of suitable bioisosteres.^39^ Unfortunately, all our attempts resulted in a complete loss of HSD17B13 activity (selected examples shown in Table 1).

**Table 1:**
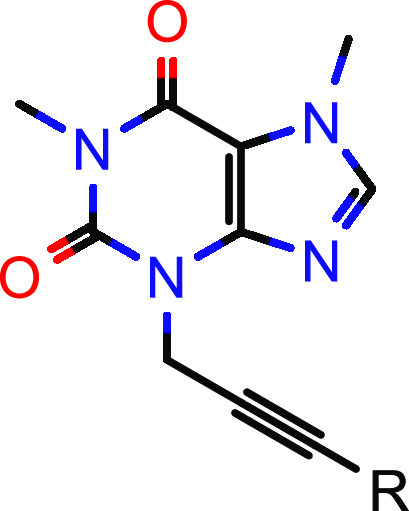

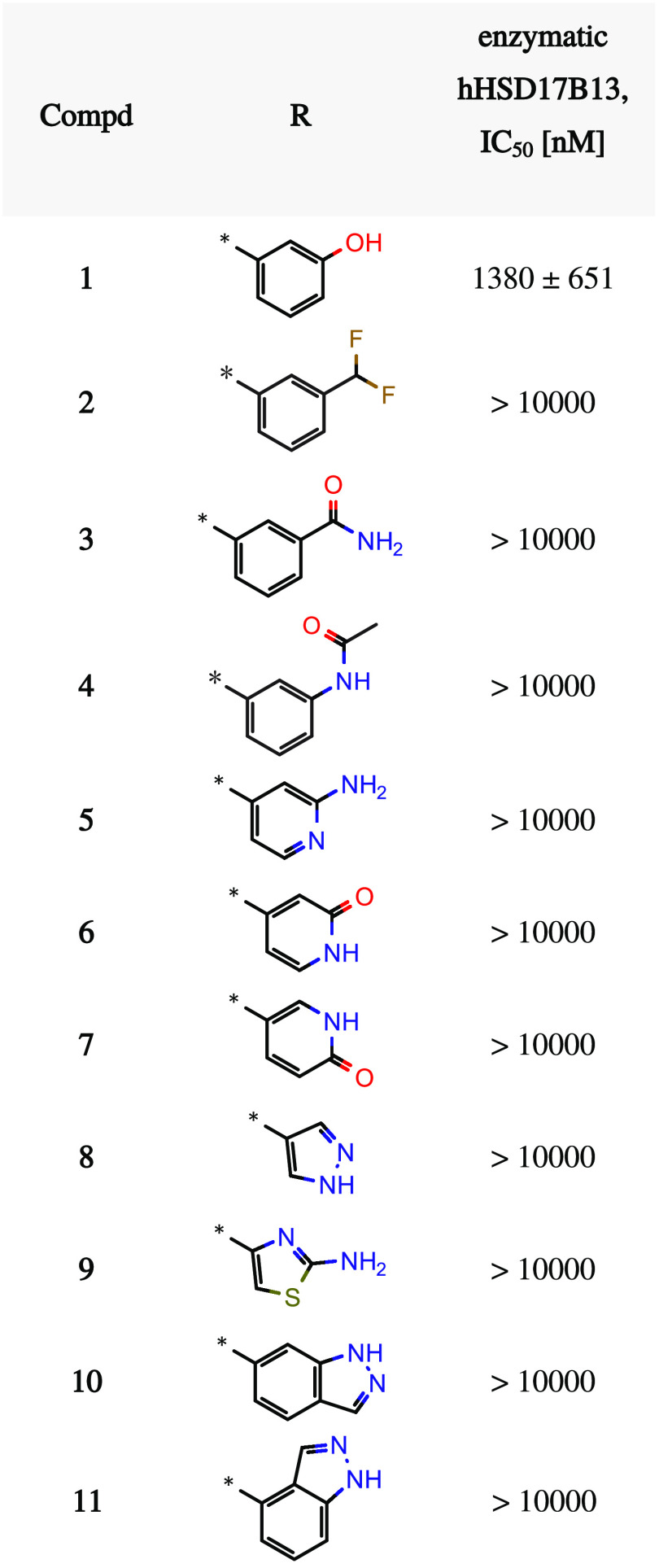
Selected Bioisosteric Replacements of the Phenol Moiety^a^

a IC_50_ values are geometric means of multiple independent measurements (*n* ≥ 3).

In parallel, we aimed to improve the ligand efficiency of screening hit **1**.^40,41^ Removal of the annulated five-membered ring of the xanthine in the north increased the ligand efficiency (LE) as well as the lipophilic efficiency (LipE)^42^ from 0.35 (**1**, Table 1) to 0.40 (**12**, Table 2) and from 4.85 (**1**) to 5.07 (**12**), respectively. With this more attractive compound in hand, we further focused our optimization efforts on metabolic stability. Structural alerts can support medicinal chemistry design teams to raise awareness and to assess the risk of certain structural motifs.^43^ Alkynes, for example, have an increased risk of cytochrome P450 mediated formation of reactive metabolites.^44,45^ Indeed, the formation of reactive metabolites was identified in a GSH adduct formation assay after incubation with human liver microsomes.^46^ Therefore, we explored the replacement of the linear central part of the molecule. As shown in Table 2, the alkyne moiety could be exchanged by several heteroaromatic groups. In particular, five-membered heterocycles such as thiadiazole **13** and thiazole **14** were able to significantly boost hHSD17B13 activity in the enzymatic as well as the cellular assays. Compared to most five-membered heterocycles, the corresponding six-membered heterocycles, exemplified by compound **22**, were less active.

**Table 2:**
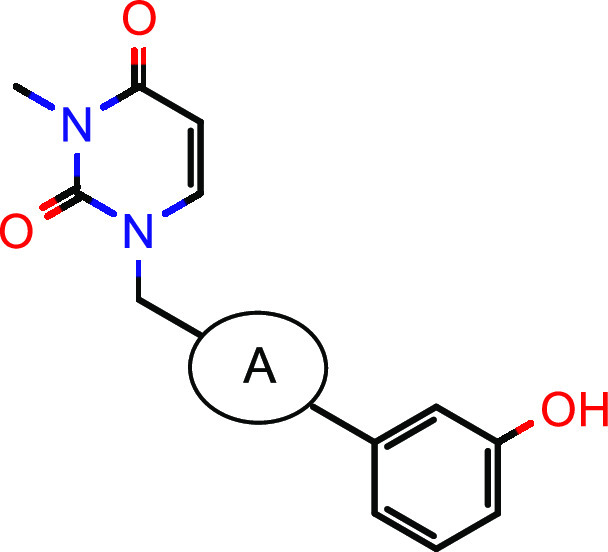

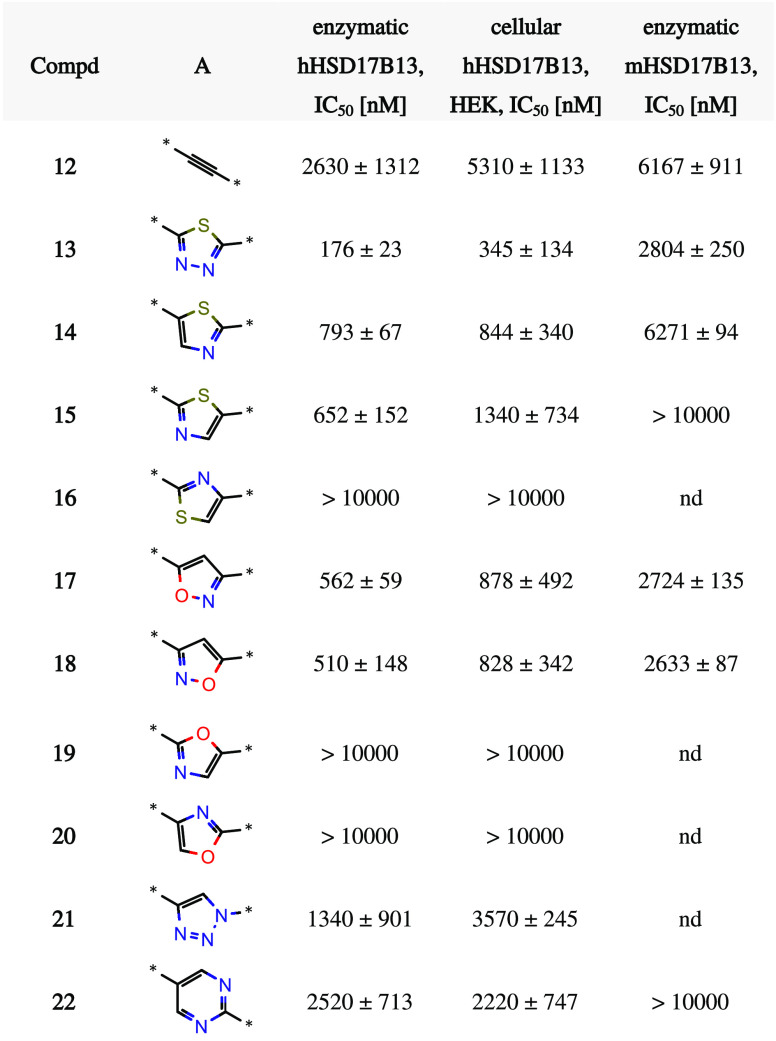
SAR Exploration of the Central Part^a^

a IC_50_ values are geometric means of multiple independent measurements (*n* ≥ 3 for enzymatic and cellular hHSD17B13, *n* = 2 for enzymatic mHSD17B13). nd = not determined.

Having removed one potential metabolic hotspot, we resumed our SAR activities around the phenol moiety to mitigate the phase II metabolism. With its well-balanced profile, compound **14** served as the basis for a systematic investigation of additional substitutions on the southern phenol (Table 3). Starting with the 2-position, small substituents such as halogens or methyl were tolerated (compare compounds **14** and **23–25**). Interestingly, the substituents in compounds **24** (chloro) and **25** (methyl) have a similar steric demand but differ in their electron-withdrawing properties and thus modulate the p*K*_a_ value of the adjacent phenol. The fact that **24** was over 100-fold more potent than **25** in the enzyme assay indicated that increasing acidity of the phenol OH is beneficial for potency. However, different dihedral angles of the chloro and methyl derivatives might also contribute to the observed potency changes. A similar trend was observed for substitution of the 6-position (compare compounds **14** and **26–29**). Halogens such as fluoro (**26**) and chloro (**27**) boosted the potency in the human HSD17B13 enzyme assay, but larger (**28**) or more polar (**29**) electron-withdrawing groups led to significantly higher IC_50_ values. We note that substituents in the 5- and 4-position of the phenol showed similar trends (compounds **30–32**/**33** in Table 3). In these positions, not only halogens (see compounds **30, 33**) but also slightly larger (**31**) residues were tolerated. Finally, we investigated double halogen substitutions (compounds **34–36, 38** in Table 3), in which a 2,6-difluoro substitution (compound **34**) turned out to be optimal to achieve double-digit nanomolar potency in the enzymatic human and mouse and the cellular human HSD17B13 assays. The low metabolic stability in human hepatocytes of compound **14** (Table 3) could be slightly improved (compound **34**) but remained dissatisfying.

**Table 3:**
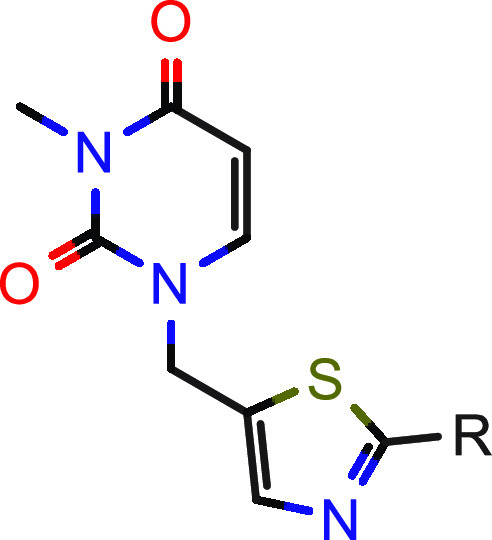

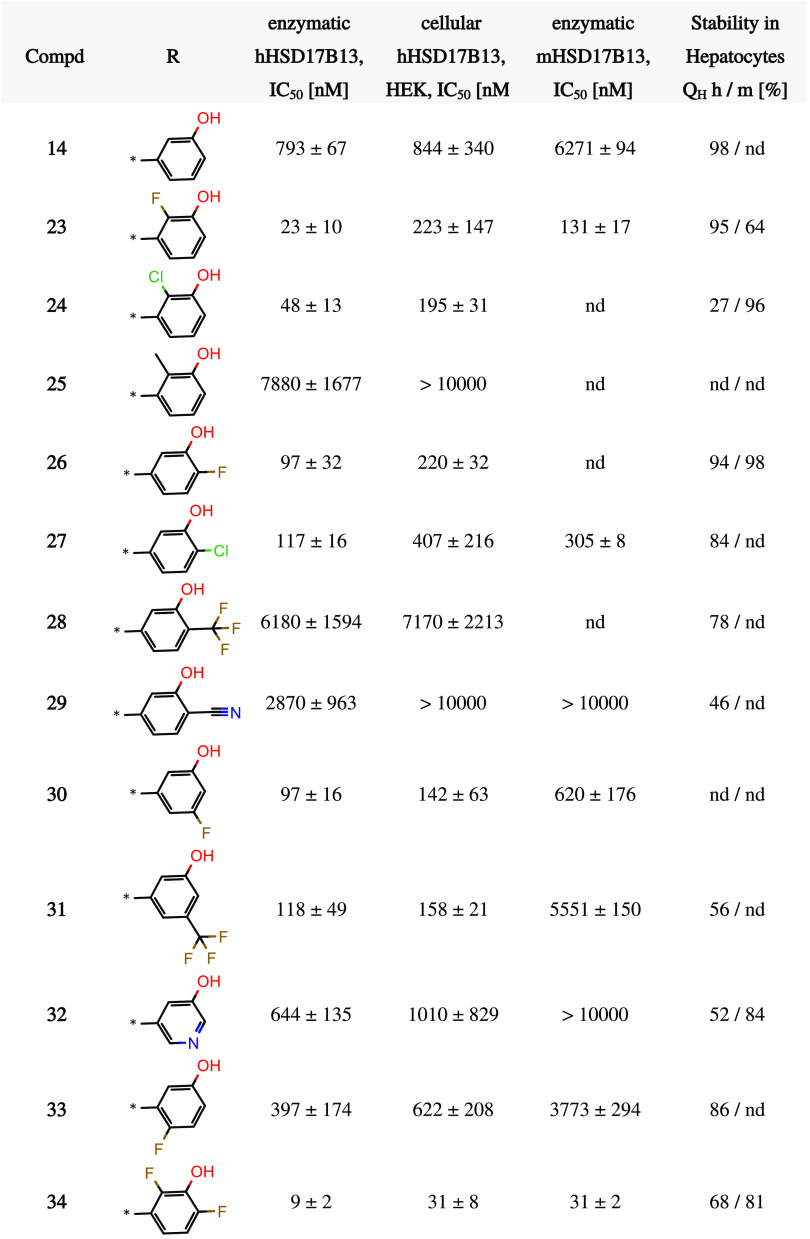

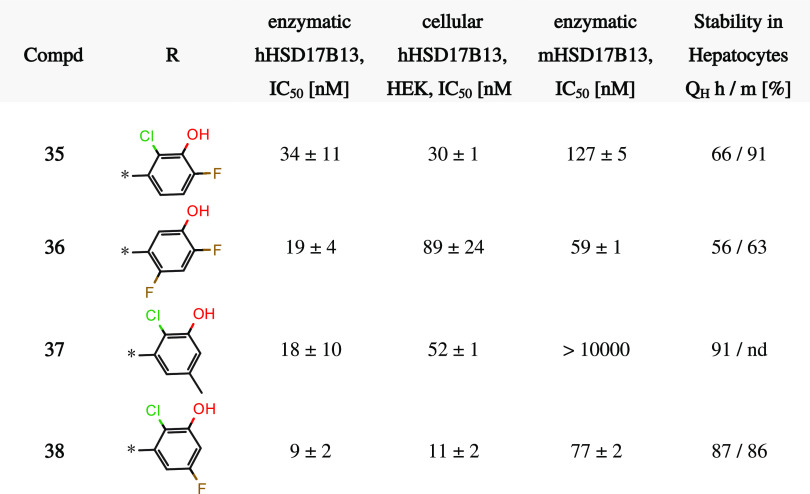
SAR Exploration of the Southern Part^a^

a IC_50_ values are geometric means of multiple independent measurements (*n* ≥ 3 for enzymatic and cellular hHSD17B13, *n* = 2 for enzymatic mHSD17B13). nd = not determined.

Next, we investigated the northern part of the lead series (Table 4) in combination with the best five-membered heterocycles (thiadiazole **13** and thiazole **14**, Table 2) and the optimized 2,6-difluorophenol moiety (see **34**, Table 3). All synthesized compounds (**39–48**, Table 4) showed single-digit nanomolar potency in the human HSD17B13 enzyme assay which translated well into a double-digit nanomolar potency in the cellular human HSD17B13 assay. Achieving a potency range in the enzymatic human and mouse HSD17B13 assays, where the IC_50_ values were in a similar range as the enzyme concentration and thereby hitting the assay wall,^47^ compound optimization was guided by the respective *K*_i_ values for tight binding inhibition using Morrison’s equation.^48,49^ Overall, *in vitro* metabolic stability in human and mouse hepatocytes remained moderate.

**Table 4:**
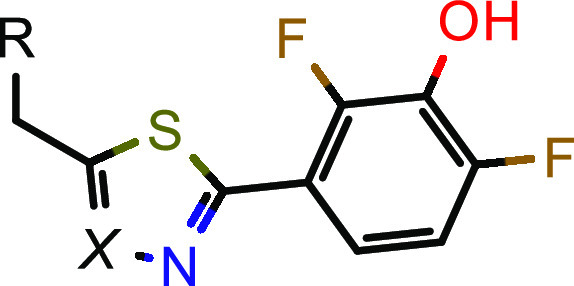

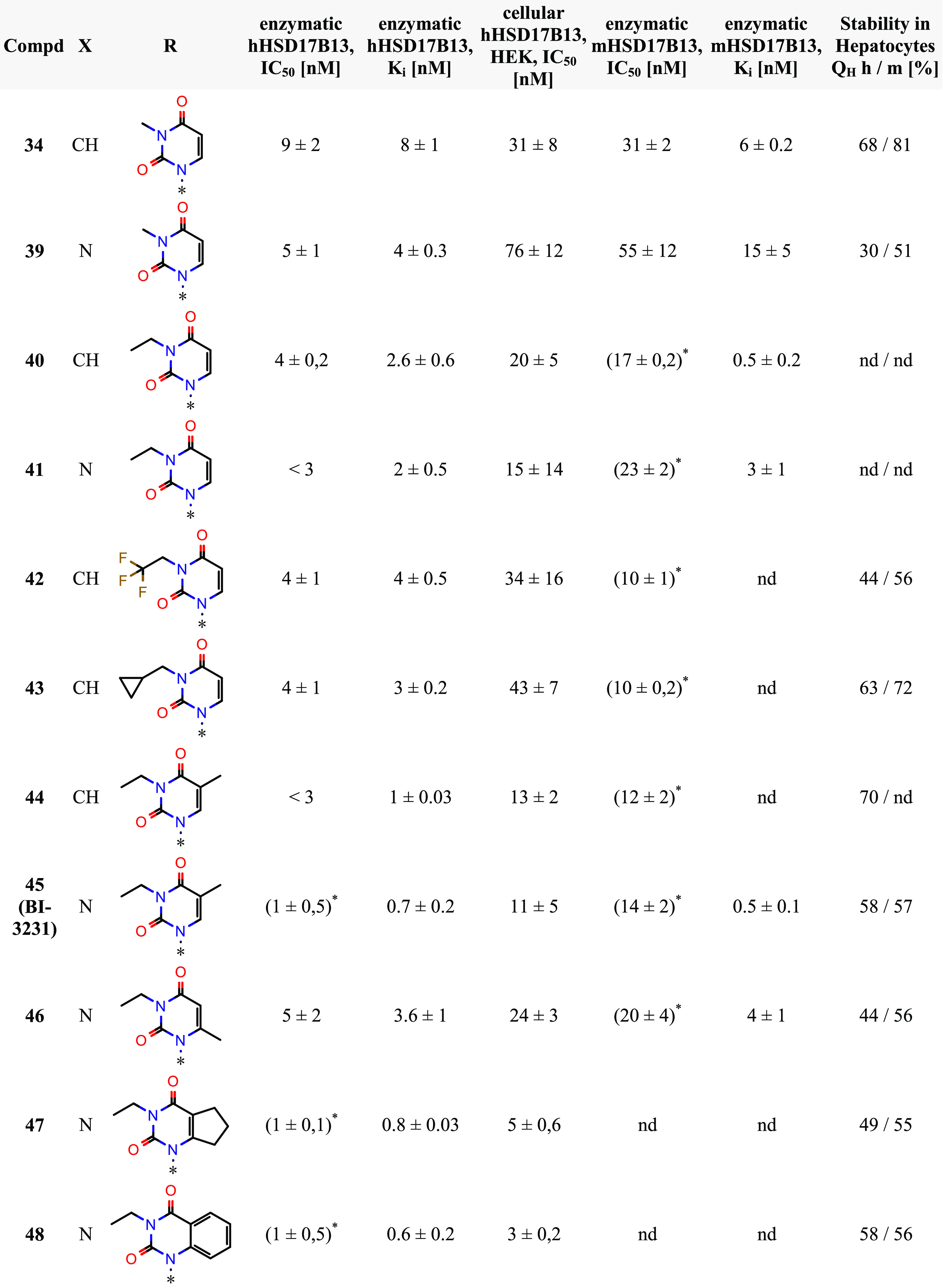
SAR Exploration of the Northern Part^a^

a IC_50_ and *K*_i_ values are geometric means of multiple independent measurements (*n* ≥ 3 for enzymatic and cellular hHSD17B13, *n* = 2 for enzymatic mHSD17B13). nd = not determined. *Real IC_50_ value unclear due to limits of the assay wall; *K*_i_ values (NAD^+^) should be used for comparison.

Due to its promising profile, compound **45 (BI-3231)** was selected for further *in vitro* profiling, focusing mainly on the investigation of on-target binding behavior, elucidation of mode of inhibition and DMPK characterization.

### *In Vitro* Profiling of Compound **45** (**BI-3231**)

**Figure 4:**
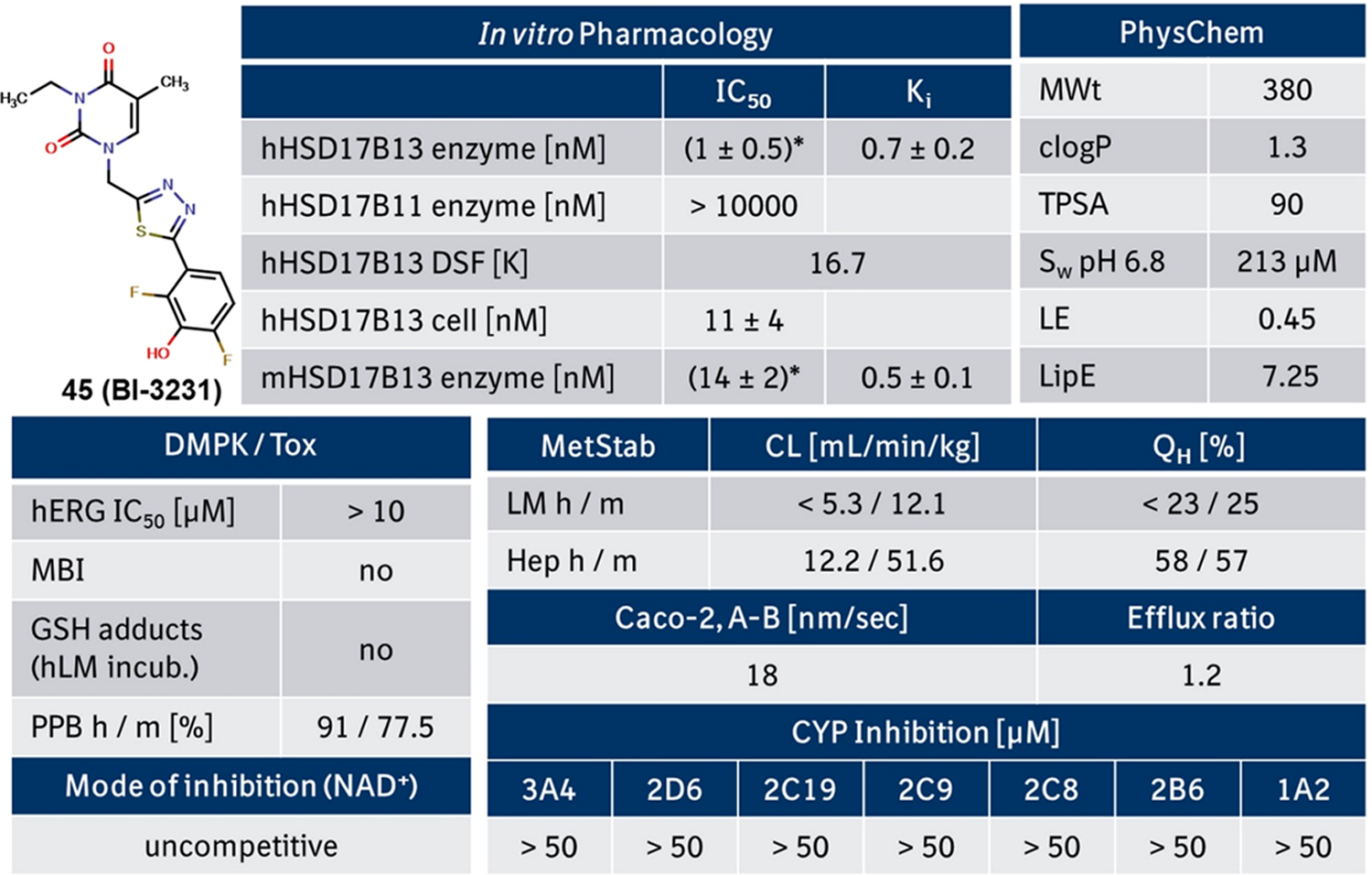
*In vitro* pharmacological, DMPK and physicochemical properties of **45 (BI-3231)**. Metabolic clearance values are upscaled from *in vitro* assays to reflect the *in vivo* situation. Abbreviations are described at the end of the manuscript. *Real IC_50_ value unclear due to limits of the assay wall; *K*_i_ values (NAD^+^) should be used for comparison.

**Figure 5:**
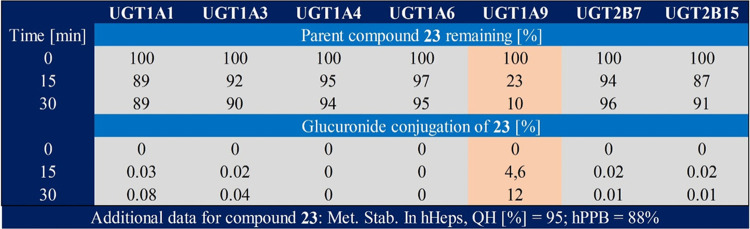
UGT phenotyping of compound **23** (Table 3) revealed UGT1A9 as the main driver for glucuronidation.

Compound **45** was profiled in several *in vitro* assays (see Figure 4). It revealed a single-digit nanomolar activity on the human and the mouse HSD17B13 enzyme (based on *K*_i_ values), which translated well into a double-digit nanomolar activity in the human HSD17B13 cellular assay. Furthermore, excellent selectivity versus the structurally related homolog HSD17B11 was achieved (Figures 3 and 4) as well as good selectivity in a commercial SafetyScreen44 panel, Cerep (see Supporting Information, Table S2). With a clog *P* of 1.3 and a topological polar surface area (TPSA) of 90, **45** exhibited a good balance between polarity and lipophilicity resulting in good aqueous solubility and high permeability in the Caco-2 assay. With no inhibition of cytochrome P450 and hERG, the safety and DDI victim profile for **45** looked favorable. In addition, no GSH adducts after metabolic activation with human liver microsomes were identified.^46^ Compared to screening hit **1** (Figure 2), compound **45** demonstrated high metabolic stability in liver microsomes and improved (but still moderate) metabolic stability in hepatocytes (Figure 4). Phenotyping of the close analogue **23** (Figure 5) revealed that UGT1A9 is the main mediator for glucuronidation and can explain the observed differences between metabolic stability in liver microsomes and hepatocytes.

### *In Vivo* Profiling of Compound **45** (**BI-3231**)

**Figure 6:**
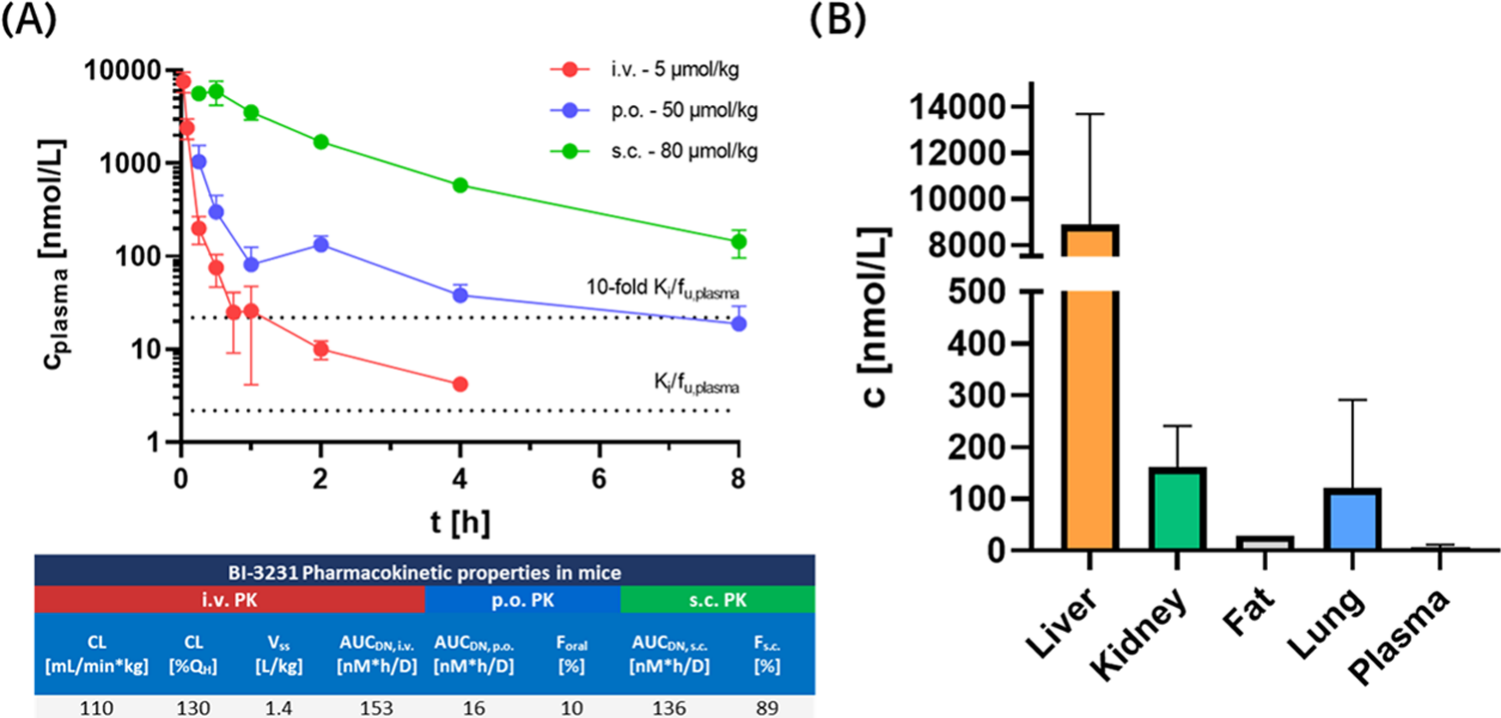
*In vivo* pharmacokinetics and tissue distribution of **45** (**BI-3231**) in mice (*n* = 3, standard deviation (SD) indicated by error bars). (A) Plasma pharmacokinetics after intravenous and oral administration in mice was characterized by a biphasic and rapid plasma clearance that exceeded the hepatic blood flow and a low oral bioavailability of 10%. Bioavailability was significantly increased by subcutaneous dosing. Relevant systemic exposure corresponding to >10-fold *in vitro* mouse *K*_i_ in unbound plasma concentration could be maintained over 8 h in mice. (B) Tissue exposure 1 h after i.v. administration indicated extensive hepatic accumulation compared to plasma and other tissues, despite comparable *in vitro* tissue binding properties (PPB = 77.5%, TB_liver_ = 87.1%, TB_kidney_ = 77.8%, TB_lung_ = 70.4%).

**Figure 7:**
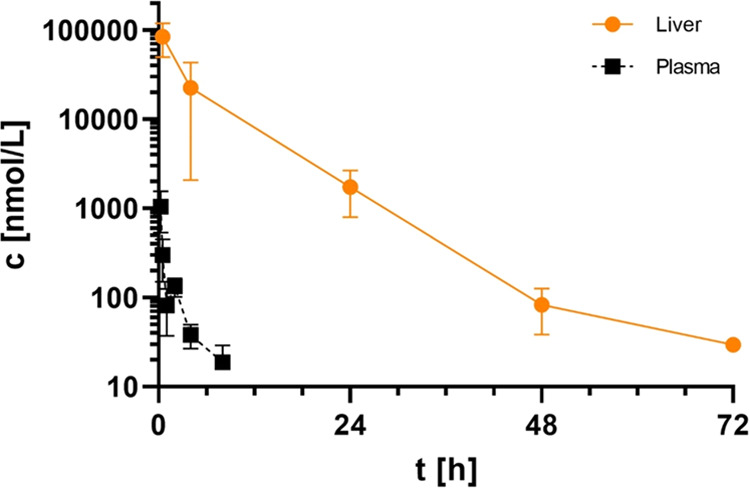
Plasma and liver pharmacokinetics in mice after single oral administration of 50 μmol/kg **45** (**BI-3231**) showing extensive compound distribution and retention in the liver compared to plasma (*n* = 3, SD indicated by error bars).

**Figure 8:**
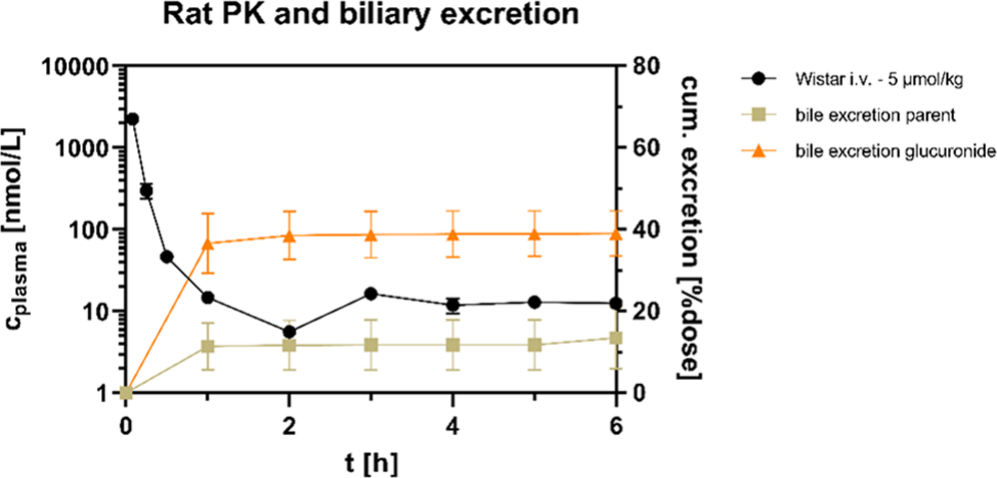
*In vivo* pharmacokinetic and bile excretion studies of **45** (**BI-3231**) in rats (*n* = 3, SD indicated by error bars). Characteristic biphasic and rapid plasma clearance was also observed in rats after intravenous administration. Biliary excretion of parent compound and its glucuronide was identified as major contributor to the overall *in vivo* plasma clearance with ∼50% of the administered dose being rapidly eliminated via the bile within the first hour of the study.

Due to its promising *in vitro* profile, compound **45** (**BI-3231**) was subsequently subjected to mouse and rat PK studies, as well as tissue distribution and excretion studies to further elucidate the fate of **45***in vivo*. Plasma pharmacokinetics in mice after intravenous and oral administration was characterized by a biphasic and rapid plasma clearance which exceeded the hepatic blood flow and low oral bioavailability. Systemic bioavailability could be significantly increased through subcutaneous administration, avoiding hepatic first-pass effects after oral absorption of **45** (Figure 6A), suggesting the involvement of hepatic uptake transporters in the *in vivo* disposition of **45**,^50^ which is not reflected by *in vitro* suspension hepatocyte clearance. While no mechanistic studies were performed to elucidate the contribution of a specific hepatic transporter protein, functional investigation of tissue exposure after intravenous application revealed a strong accumulation of **45** (**BI-3231**) in liver compared to plasma and other tissues (Figure 6B). As the target protein HSD17B13 is primarily expressed and located in hepatocytes,^17^ we wanted to understand the tissue pharmacokinetics as well as the underlying mechanism of the observed liver accumulation. Therefore, we determined liver and plasma exposure of **45** in mice time-dependently after single oral administration over 72 h (Figure 7) and observed extensive exposure and retention in the liver compared to plasma. Physicochemical properties of **45** (like acidity, low molecular weight, low lipophilicity and low protein binding) can be indicators for involvement of OATPs or OATs in hepatic drug disposition,^51,52^ as the dissociated form of **45** (corresponding phenolate anion) carries a negative charge. In addition, phenolic compounds are susceptible to phase II metabolic conjugation (i.e., glucuronidation, sulfation) in the liver,^53^ followed by excretion to the hepatic bile ducts, often mediated by apical transport mechanisms while constant OAT/OATP mediated reuptake of such conjugates from systemic circulation occurs.^54,55^ Subsequently, they often undergo enterohepatic circulation. A close analogue of **45** (compound **23**) has been demonstrated to undergo mainly UGT1A9 mediated glucuronidation, leading to loss of HSD17B13 inhibitory activity (Figure 5). As the bile excreted fraction of compound **45** is not available for interaction with an intracellular target, it was important to understand the biliary excretion of the compound in the context of extensive liver accumulation in more detail. For this purpose, plasma pharmacokinetics after i.v. administration and biliary excretion of parent compound **45** as well as the respective glucuronide was assessed in rats, revealing its glucuronidation and biliary excretion (Figure 8) as major contributors to the observed *in vivo* clearance.

In summary, in depth *in vivo* pharmacokinetic profiling of compound **45 (BI-3231)** in rodents revealed a more pronounced plasma clearance than expected from *in vitro* hepatocyte studies with a biphasic profile, which had previously been observed for other phenolic structures.^56,57^ As a large fraction of the administered dose has been found in the form of glucuronide in bile fluid, involvement of enterohepatic circulation seems the likely driver for the terminally flat PK. While **45** was rapidly cleared from plasma, considerable hepatic exposure was maintained over 48 h. However, impacts of *in vivo* tissue binding, biliary excretion and potential involvement of transporter mediated hepatic uptake complicate a quantitative assessment of the fraction that is available for direct target interaction. Since it is unclear to which extent the hepatic enrichment of compound **45 (BI-3231)** beneficially contributes to the inhibition of HSD17B13, reliable methods to assess direct *in vivo* HSD17B13 target engagement need to be established. We conclude that substantial multiples of *in vitro* pharmacologically active concentrations could be achieved and maintained systemically in mice using conventional dosing approaches, potentially enabling further *in vivo* characterization and the study of pharmacodynamic effects of **45 (BI-3231)** in subchronic murine models of NASH.

Knowing the *in vivo* PK profile of compound **45 (BI-3231)**, we focused further efforts on the *in vitro* characterization of this promising HSD17B13 inhibitor to elucidate its binding properties and mode of inhibition.

### On-Target Binding and Mode of Inhibition of Compound **45** (**BI-3231**)

**Figure 9:**
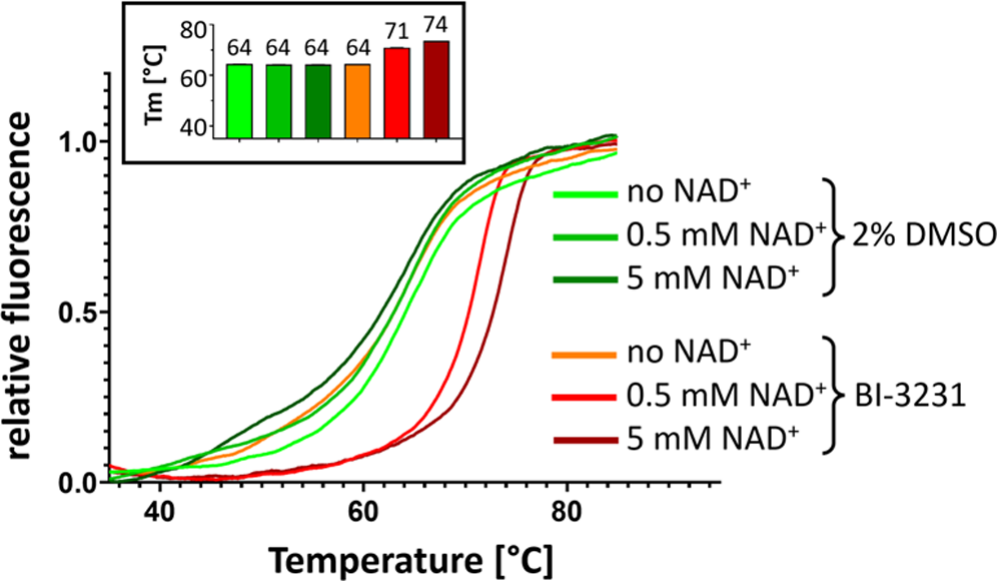
NAD^+^ dependency of compound **45 (BI-3231)** binding: hHSD17B13 melting curves from Thermal Shift Assay experiment (nanoDSF) in the presence of 2% DMSO or **BI-3231** at increasing NAD^+^ concentrations (0, 0.5, and 5 mM) showing thermal stabilization by **BI-3231** only in the presence of NAD^+^. Inset: corresponding melting temperatures (*n* = 4, SD indicated by error bars).

We tested compound **45** for its binding properties on the recombinant human HSD17B13 enzyme via Thermal Shift Assay experiments (nanoDSF) to confirm on-target binding.^58^ In the presence of NAD^+^, the melting temperature of HSD17B13 treated with 5 μM **BI-3231** was significantly higher than the dimethyl sulfoxide (DMSO) control (*T*_m_ shift = 16.7 K), confirming specific binding to human HSD17B13 (Figure 9, shown in dark green and dark red). Surprisingly, no thermal stabilization of HSD17B13 could be observed with NAD^+^ alone. The stabilizing effect of **BI-3231** is highly dependent on the presence of NAD^+^ (Figure 9), indicating that the ligand binding pocket might only be formed after NAD^+^ has bound, suggesting an ordered bi–bi mechanism.^59^

**Figure 10:**
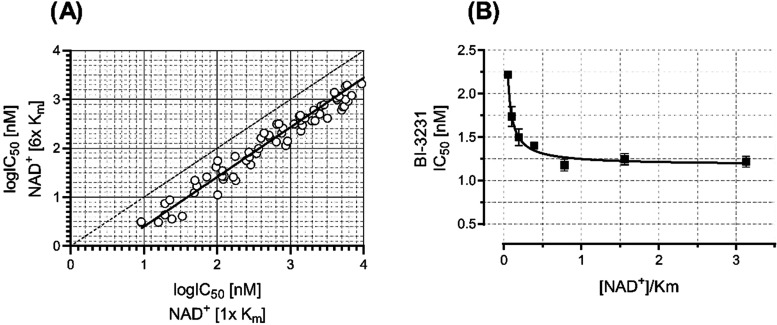
Importance of NAD^+^ for potency of the phenol lead class and compound **45 (BI-3231)**. (A) Log IC_50_ values of representative compounds of the phenol lead class assayed in the presence of NAD^+^ at concentrations at *K*_m_ and 6-fold *K*_m_ (linear regression indicated by solid line (*r*^2^ = 0.95), 1:1 correlation indicated by dotted line). (B) IC_50_ values of compound **45** plotted against [NAD^+^]/*K*_m_. Decreasing IC_50_ values (*n* = 2, SD indicated by error bars) at increasing [NAD^+^]/*K*_m_ values (logistic regression indicated by solid line, *r*^2^ = 0.96), indicate an uncompetitive mode of inhibition of **45** against NAD^+^.

To further investigate the NAD^+^ dependency of the phenol lead class, we performed cross-titrations of test compounds and NAD^+^ at varying concentrations in the human HSD17B13 enzyme assay, while keeping estradiol constant at the highest, practically feasible concentration. In these experiments, we observed a significant NAD^+^-dependent decrease of the IC_50_ values for the phenols (Figure 10A) and compound **45 (BI-3231)** (Figure 10B), illustrating an uncompetitive mode of inhibition.^49,60−62^ One might expect that NAD^+^ concentrations greater than *K*_m_ would lead to even lower IC_50_ values, but with an enzyme concentration of 1 nM in the human HSD17B13 assay, the activity of **45 (BI-3231)** is most probably beyond the detection limit (“assay wall”)^47^ so that its IC_50_ for NAD^+^ concentrations greater than *K*_m_ reflects the upper limit to its real potency.

Our experimental data showed the importance of NAD^+^ for binding and potency of the phenol lead class including compound **45 (BI-3231)** and motivated us for computational modeling approaches potentially explaining our observations.

### Computational Modeling: Binding Hypothesis for Compound **45** (**BI-3231**)

**Figure 11:**
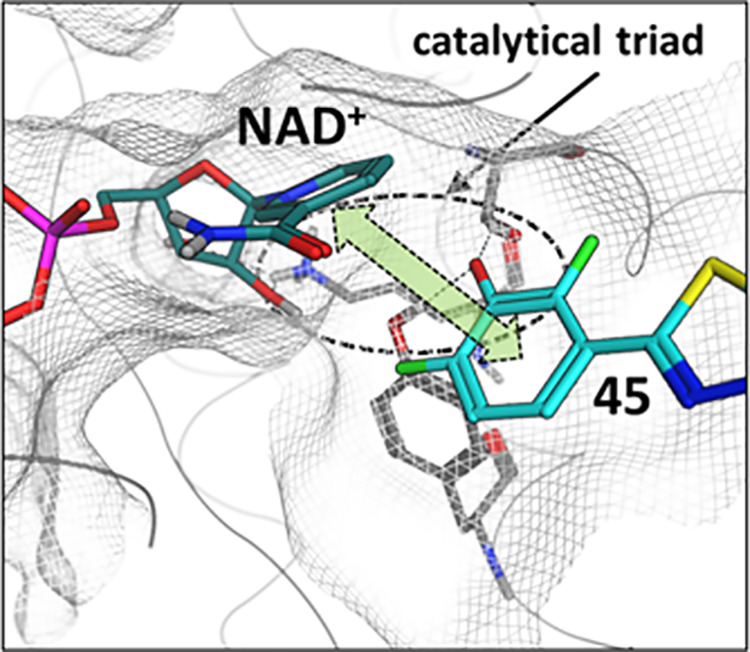
Binding hypothesis for compound **45 (BI-3231)**. Postulated interaction of **45** (cyan) and NAD^+^ (dark cyan). The phenol group of **45** interacts with Ser172 and Tyr185 from the catalytical triad (gray residues), thereby inducing charge transfer and dispersion interactions (green arrow) between **45** and NAD^+^.

The NAD^+^ dependency of the phenol class including compound **45 (BI-3231)** is supported by a computational homology model revealing the interaction of **45** with NAD^+^. Based on functional data and the homology model, we postulate that the positively charged NAD^+^ in the co-factor binding pocket leads to an increased binding affinity of the spatially adjacent negatively charged phenol **45** (Figure 11), resulting in a NAD^+^ dependency not only for binding, but also for inhibition of the enzymatic activity of HSD17B13.

### HSD17B13 Inhibitor **45** (**BI-3231**) as Chemical Probe for Open Science

**Figure 12:**
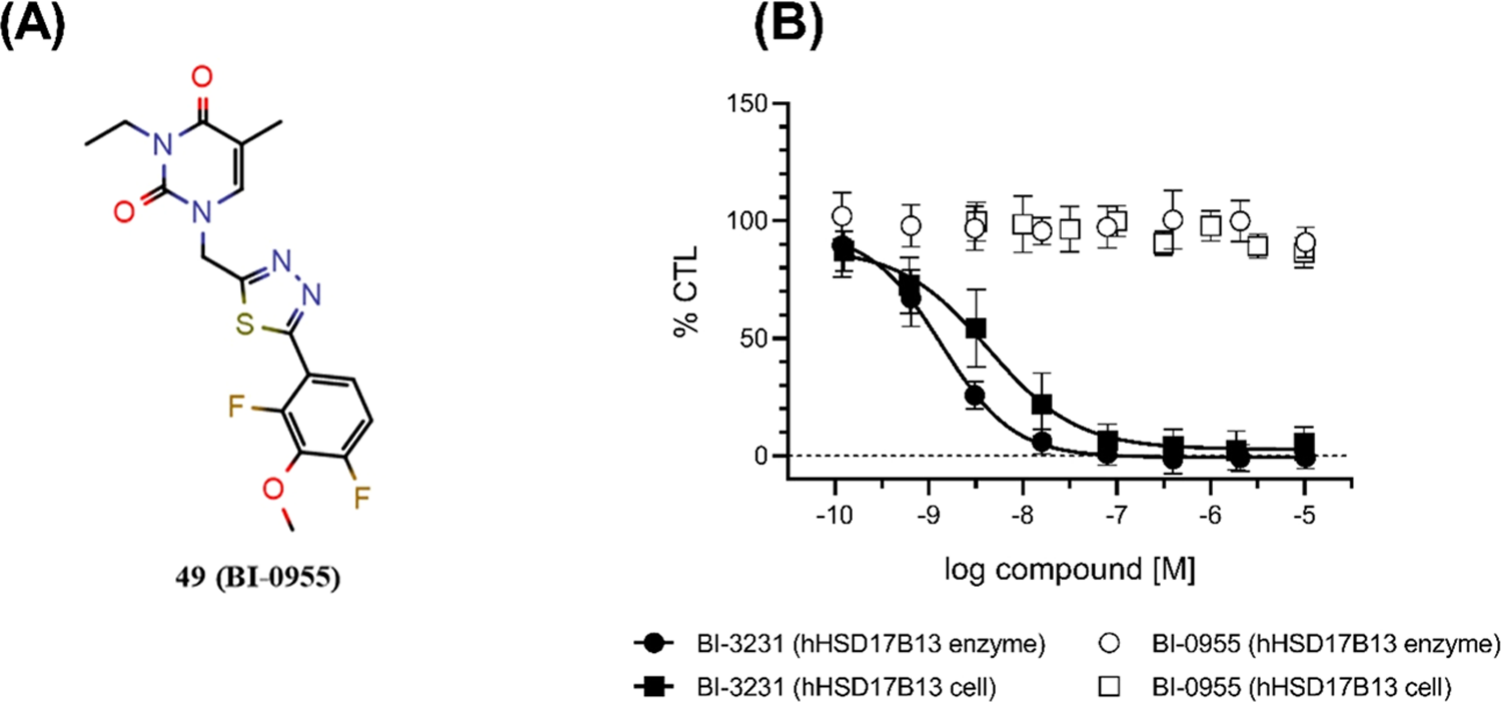
(A) Inactive HSD17B13 control compound **49 (BI-0955)**. (B) Dose–response curves of **BI-0955 (49)** and **BI-3231 (45)** in the hHSD17B13 enzyme and cellular assays (all *n* ≥ 3; SD indicated by error bars; solid lines show fitting of a four-parameter logistical equation).

The public availability of well-characterized chemical probe molecules can help to elucidate pharmacology and mode of action of a target of interest. In recent years, the “Structural Genomics Consortium” (SGC) and its partners have made a concerted effort to define clear criteria for high-quality chemical probes.^63−65^ Once accepted, the SGC^66^ and other platforms such as opnMe^67,68^ or EUbOPEN^69^ provide information of in-depth profiled compounds to the scientific community and support Open Science by free worldwide shipments of chemical probe samples. An ambition of the SGC and its partners is to discover a pharmacological modulator for every protein in the human proteome by the year 2035 (“Target 2035”).^70,71^ Therefore, we are pleased to report the discovery of the novel potent and selective HSD17B13 inhibitor **BI-3231** (compound **45**). Together with **BI-0955** (compound **49**, Figure 12A), which can be used as an inactive control (Figure 12B), we make **BI-3231** as well-characterized chemical probe available to the worldwide scientific community via the opnMe platform.^67,68^

### Syntheses of Screening Hit **1** and Chemical Probe **45** (**BI-3231**)

**Scheme 1:**
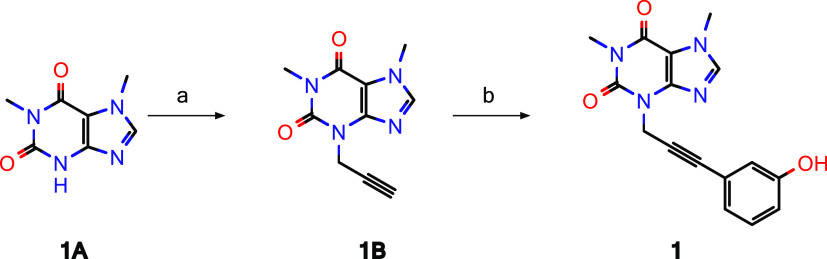
Synthesis of Screening Hit **1** Reagents and conditions: (a) propargyl bromide, potassium carbonate, *N*,*N*-dimethylformamide (DMF), 70% yield; (b) 3-iodo phenol, copper(I) iodide triethylamine, tetrakis(triphenylphosphine)palladium(0), DMF, 36% yield.

Screening hit **1** was synthesized as outlined in Scheme 1. Commercially available 1,7-dimethyl-2,3,6,7-tetrahydro-1*H*-purine-2,6-dione (=paraxanthine, **1A**) was alkylated with propargyl bromide and subsequently reacted with 3-iodo phenol under Sonogashira conditions to furnish compound **1**.

**Scheme 2:**
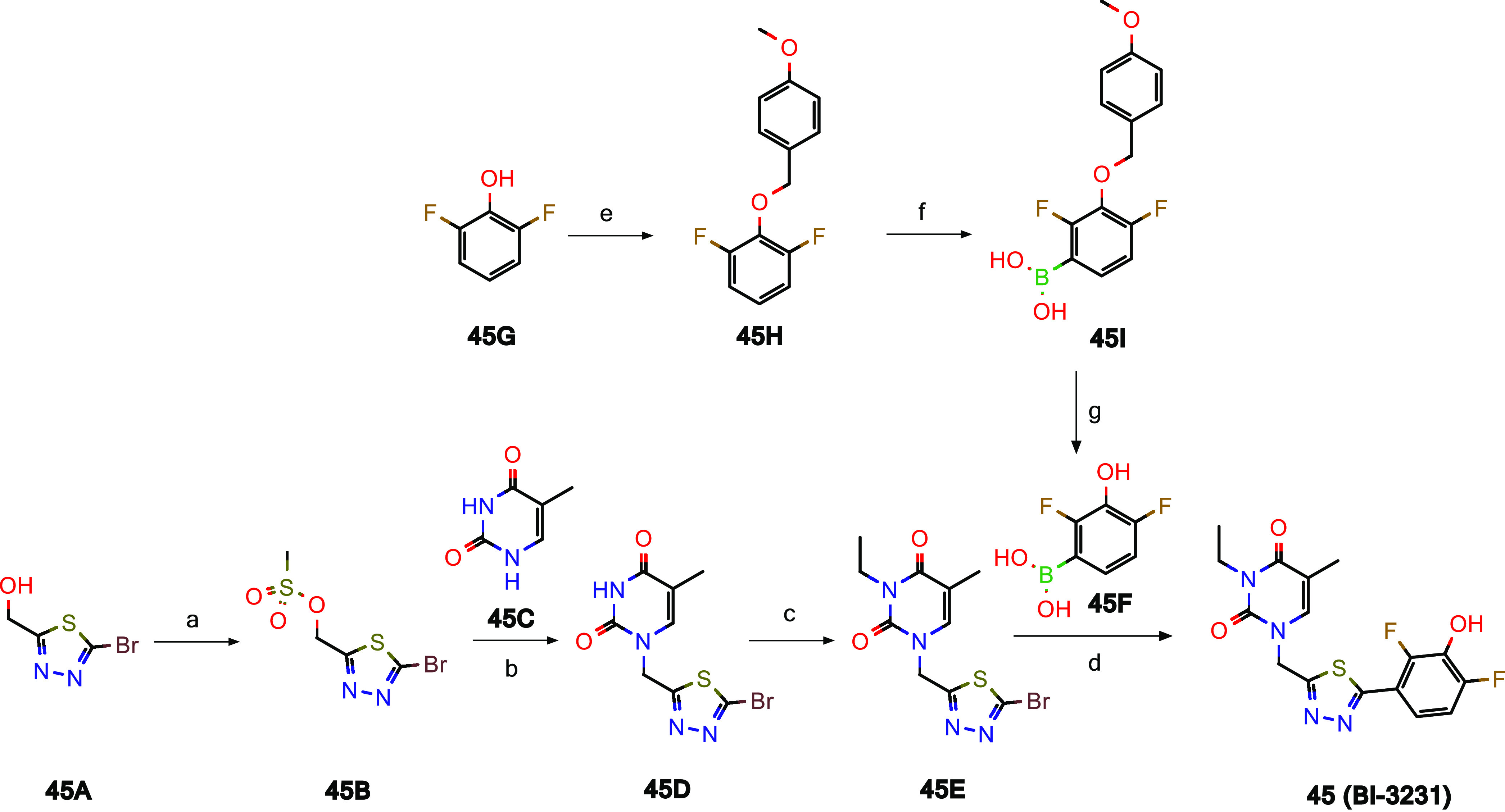
Synthesis of HSD17B13 Chemical Probe **45 (BI-3231)** Reagents and conditions: (a) MeSO_2_Cl, NEt_3_, CH_2_Cl_2_, 90% yield; (b) *N*,*O*-bis(trimethylsilyl)acetamide, MeCN, 76% yield; (c) EtI, K_2_CO_3_, DMF, 69% yield; (d) [bis(2,6-di-3-pentylphenyl)imidazol-2-ylidene](3-chloropyridyl)palladium(II) dichloride, EtOH, water, 51% yield; (e) 1-(chloromethyl)-4-methoxybenzene, K_2_CO_3_, MeCN, quant. yield; (f) *n*-BuLi, tetrahydrofuran (THF), −78 °C, trimethyl borate, then 4 M HCl, 91% yield; (g) trifluoroacetic acid (TFA), CH_2_Cl_2_, 69% yield.

As outlined in Scheme 2, chemical probe **45 (BI-3231)** was synthesized in four steps starting from commercially available **45A**. Alcohol **45A** could be converted to the corresponding mesylate **45B**, which was directly used for the alkylation of thymine **45C** giving rise to intermediate **45D**. The synthesis of **45 (BI-3231)** was completed by alkylation of the free NH of **45D** with ethyl iodide followed by Suzuki coupling with boronic acid **45F**. The boronic acid **45F** itself was prepared in three steps as depicted in Scheme 2.

## Conclusions

**BI-3231** (compound **45**) is the first potent and selective chemical probe reported for HSD17B13, a potential new target for the treatment of NASH and other liver diseases. With a high-throughput screening campaign, specific HSD17B13 inhibitors were identified. The weakly active compound **1** was subsequently optimized resulting in **45 (BI-3231)**, with improved functional and physicochemical properties as well as an improved DMPK profile. The phenol lead series, including **BI-3231**, showed a strong NAD^+^ dependency for binding and inhibition of HSD17B13. **BI-3231** was investigated in pharmacokinetic studies revealing a disconnect between *in vitro* and *in vivo* clearance while showing extensive liver tissue accumulation.^72^ We note that pronounced phase II metabolic biotransformation may limit its use in metabolically competent systems. Nonetheless, due to its improved overall profile, we suggest the well-characterized specific HSD17B13 inhibitor **BI-3231** as a valuable chemical probe to further elucidate the biological function of HSD17B13. As the *in vivo* pharmacokinetic/pharmacodynamic (PK/PD) relationship of **BI-3231** including target engagement biomarkers for HSD17B13 inhibition are not known, further *in vivo* evaluation in relevant models of NASH is required. Given the high clearance and short half-life of **BI-3231**, a tailored approach such as multiple daily administrations or the development of an extended-release formulation might be needed to maintain relevant target exposure in subchronic animal models. To support further studies on HSD17B13 via Open Science, **BI-3231** will be available for free to the scientific community through the opnMe^67,68^ platform (Please place your free order for **BI-3231** here: https://opnme.com/molecules/hsd17b13-inhibitor-bi-3231). **BI-3231** could also be used as a potential starting point for the synthesis of Proteolysis Targeting Chimeras (PROTACs)^73^ which would allow us to compare phenotypes resulting from inhibition versus degradation of HSD17B13.

## Experimental Section

### Compound Synthesis

All commercially available chemicals were used as received from their commercial supplier. Anhydrous solvents were either purchased or prepared according to standard procedures^74^ and stored over molecular sieves under argon. Unless stated otherwise, all reactions were carried out in oven-dried (at 120 °C) glassware under an inert atmosphere of argon. A Biotage Initiator Classic microwave reactor was used for reactions conducted in a microwave oven. Reactions were monitored by thin-layer chromatography (TLC) on aluminum-backed plates coated with Merck Kieselgel 60 F 254 with visualization under UV light at 254 nm, and with high-performance liquid chromatography–mass spectrometry (HPLC-MS) analysis (for HPLC-MS methods, see Supporting Information, Table S1). Unless stated otherwise, crude products were purified by flash column chromatography on silica (using a Biotage IsoleraOne, Biotage IsoleraFour or CombiFlash Teledyne Isco system) or by (semi)-preparative reversed-phase HPLC (Agilent or Waters). Unless specified otherwise, the purity of all final compounds was determined to be ≥95% by liquid chromatography–mass spectrometry (LC–MS). Nuclear magnetic resonance (NMR) spectra were recorded at room temperature (22 ± 1 °C), on a Bruker Avance 400 spectrometer with tetramethylsilane as an internal reference. Chemical shifts δ are reported in parts per million (ppm). ^1^H NMR spectra were referenced to the residual partially nondeuterated solvent signal of DMSO (δ = 2.50 ppm). Coupling constants *J* are reported in Hz, and splitting patterns are described as br = broad, s = singlet, d = doublet, t = triplet, q = quartet, quin = quintet and m = multiplet. High-resolution mass spectra were recorded on a Thermo Scientific LTQ Orbitrap XL using electrospray ionization in positive ion mode (ESI+). MarvinSketch software version 20.19.1 was used to generate compound names.

### Preparation of Compounds

#### 1,7-Dimethyl-3-(prop-2-yn-1-yl)-2,3,6,7-tetrahydro-1*H*-purine-2,6-dione (**1B**)

Step a (Scheme 1): To a stirred solution of 1,7-dimethyl-2,3,6,7-tetrahydro-1*H*-purine-2,6-dione (**1A**, 10.0 g, 56.0 mmol, commercially available, CAS-RN: [611-59-6]) in DMF (100 mL) was added potassium carbonate (15.3 g, 111 mmol), and the resulting reaction mixture was stirred at rt for 10 min. Next, propargyl bromide (9.91 g, 83.0 mmol, CAS-RN: [106-96-7]) was added and the resulting mixture was stirred at rt for 15 h. The reaction mixture was filtered, diluted with EtOAc (350 mL), and washed with ice water (2–3×). The organic layer was dried over Na_2_SO_4_, filtered, and concentrated under reduced pressure to yield the crude product as a liquid. The crude liquid was washed with *n*-pentane (3×) to obtain the pure compound **1B** (8.50 g, 70% yield). LC-MS (method 1): *t*_R_ = 0.41 min; MS (ESI^+^): *m*/*z* = 219 [M + H]^+^.

#### 3-[3-(3-Hydroxyphenyl)prop-2-yn-1-yl]-1,7-dimethyl-2,3,6,7-tetrahydro-1*H*-purine-2,6-dione (**1**)

Step b (Scheme 1): A solution of **1B** (0.60 g, 3.00 mmol) in DMF (20 mL) was degassed with argon gas for 10 min. Then, 3-iodo phenol (1.21 g, 5.00 mmol CAS-RN: [626-02-8]), copper(I) iodide (21.0 mg, 0.11 mmol) and triethylamine (1.11 g, 11.0 mmol) were added, and the reaction mixture was degassed again. Tetrakis(triphenylphosphine)-palladium(0) (191 mg, 0.17 mmol) was added, and the reaction mixture was heated to 100 °C for 6 h. The reaction was monitored by TLC (10% MeOH in dichloromethane (DCM)). Upon completion, the reaction mixture was filtered, concentrated under reduced pressure, and the residue was purified by flash column chromatography (silica gel 100–200 mesh, 2–3% MeOH in DCM) to obtain pure compound **1** (0.31 g, 36% yield). LC-MS (method 4): *t*_R_ = 1.77 min; MS (ESI^+^): *m*/*z* = 311 [M + H]^+^. ^1^H NMR (400 MHz, DMSO-*d*_6_) δ ppm: 3.25 (s, 3H), 3.90 (s, 3H), 4.97 (s, 2H), 6.71–6.82 (m, 3H), 7.14 (t, *J* = 7.86 Hz, 1H), 8.06 (s, 1H), 9.60 (s, 1H). HRMS (ESI, [M + H]^+^): calcd for C_16_H_15_N_4_O_3_: 311.1139, found: 311.1140.

#### 1-{[5-(2,4-Difluoro-3-hydroxyphenyl)-1,3,4-thiadiazol-2-yl]methyl}-3-ethyl-5-methyl-1,2,3,4-tetrahydropyrimidine-2,4-dione (**45, BI-3231**)

Step a (Scheme 2): (5-Bromo-1,3,4-thiadiazol-2-yl)methanol (**45A**, 1.00 g, 5.13 mmol, commercially available, CAS-RN: [1339055-00-3]) was dissolved in DCM (30 mL) and triethylamine (1.10 mL, 7.89 mmol). Then, methane sulfonyl chloride (0.60 mL, 7.75 mmol) was added dropwise and the reaction mixture was stirred at rt for 1 h. The mixture was partitioned between an aqueous solution of citric acid and DCM. The organic layer was separated and concentrated under reduced pressure to furnish (5-bromo-1,3,4-thiadiazol-2-yl)methyl methanesulfonate (**45B**, 1.26 g, 90% yield). The crude product was used in the next step without further purification. Step b (Scheme 2): Thymine **45C** (500 mg, 3.97 mmol, commercially available, CAS-RN: [65-71-4]) was suspended in acetonitrile (ACN, 15 mL), *N*,*O*-bis(trimethylsilyl) acetamide (2.42 mL, 9.90 mmol) was added, and the mixture was stirred at rt for 4 h. **45B** (1.20 g, 4.39 mmol) was dissolved in ACN (10 mL) and added to the reaction mixture. Tetrabutylammonium iodide (300 mg, 0.81 mmol) was added, and the resulting mixture was stirred at 80 °C for 6 h, then cooled to rt, and the reaction was carefully quenched with water (30 mL). The precipitate was filtered and washed with water, then with ACN (2 × 1 mL) followed by diethyl ether (2 × 5 mL). The crude product was dried at 60 °C for 1 h to yield 1-[(5-bromo-1,3,4-thiadiazol-2-yl)methyl]-5-methyl-1,2,3,4-tetrahydro-pyrimidine-2,4-dione (**45D**, 910 mg, 76% yield). LC-MS (method 3): *t*_R_ = 0.67 min; MS (ESI^+^): *m*/*z* = 303 [M + H]^+^. The crude product was used in the next step without further purification. Step c (Scheme 2): **45D** (900 mg, 2.97 mmol) was dissolved in DMF (5 mL). Potassium carbonate (820 mg, 5.93 mmol) and iodoethane (360 μL, 4.48 mmol) were added, and the resulting mixture was stirred at 70 °C for 2 h. Next, the reaction mixture was cooled to rt and poured on water (30 mL), stirred for additional 10 min, and then filtered. The crude product was washed with water followed by MeOH (2 × 1 mL) and diethyl ether (2 × 3 mL), before being dried at 60 °C in the drying chamber to provide 1-[(5-bromo-1,3,4-thiadiazol-2-yl)methyl]-3-ethyl-5-methyl-1,2,3,4-tetrahydropyrimidine-2,4-dione (**45E**, 680 mg, 69% yield). The crude product was used in the next step without further purification. Step e (Scheme 2): 2,6-Difluorophenol **45G** (50.0 g, 384 mmol, commercially available, CAS-RN: [28177-48-2]) was dissolved in ACN (600 mL). Potassium carbonate (81.2 g, 588 mmol) was added, followed by addition of 1-(chloromethyl)-4-methoxybenzene (54.2 mL, 400 mmol, commercially available, CAS-RN: [824-94-2]). The pale brown suspension was stirred at 70 °C for 90 min and then at rt overnight. The reaction mixture was filtered and concentrated under reduced pressure. The resulting light brown oil was dissolved in EtOAc (600 mL) and washed with 50% saturated aq. NaHCO_3_ (2 × 200 mL). The organic portion was dried over MgSO_4_, filtered, and concentrated to yield **45H** (106 g, quantitative yield). The product was re-dried: dissolved in DCM, dried over Na_2_SO_4_, filtered, concentrated under reduced pressure, and stored under vacuum for 24 h. This material was used in the next step without further purification. Step f (Scheme 2): **45H** (12.0 g, 48.0 mmol) was dissolved in anhydrous THF (200 mL) and cooled in an acetone/dry ice bath (internal temperature −74 °C). *n*-Butyllithium (2.5 M, 25.0 mL, 62.4 mmol) was added dropwise over a period of 15 min (internal temperature kept below −70 °C). After the addition, the solution was stirred at −74 °C for 1 h. Trimethyl borate (7.49 mL, 67.2 mmol) was added dropwise to the reaction mixture, and stirring was continued at −70 °C for 10 min, before the cooling bath was removed, and the reaction mixture was stirred for 1 h warming to rt. The reaction was slowly quenched with aq. HCl (4 M, 10 mL), and the resulting mixture was diluted with EtOAc and water. The layers were separated, and the aqueous layer was further extracted with EtOAc. The combined organic layers were dried over MgSO_4_, filtered, and concentrated under reduced pressure giving rise to {2,4-difluoro-3-[(4-methoxyphenyl)methoxy]phenyl}boronic acid (**45I**, 16 g, 91% yield) as a yellow oil/solid mixture. The crude product was used in the next step without further purification. Step g (Scheme 2): **45I** (30.0 g, 91.8 mmol) was suspended in DCM (200 mL). Then, TFA (20.0 mL, 259.2 mmol) was added dropwise (reaction became a solution, then reformed a precipitate). The resulting mixture was stirred at rt for 1 h. A purple suspension formed, the solid was collected by suction filtration, and dried under vacuum to give (2,4-difluoro-3-hydroxyphenyl)boronic acid (**45F**, 11 g, 69% yield). LC-MS (method 2): *t*_R_ = 0.25 min. The product was used for step d without further purification. Step d (Scheme 2): **45E** (58.0 mg, 0.18 mmol), **45F** (46.0 mg, 0.26 mmol), and cesium carbonate (143 mg, 0.44 mmol) were suspended in EtOH (2 mL) and H_2_O (0.5 mL). Pd-PEPPSI 2Me-IPent Cl (7.40 mg, 0.01 mmol) was added, and the resulting mixture was stirred at 80 °C for 75 min. The reaction mixture was diluted with DMF (1 mL), filtered, acidified with TFA, and purified by preparative HPLC (Sunfire C18, ACN, H_2_O/TFA) to give **45** (34 mg, 51% yield). LC-MS (method 3): *t*_R_ = 0.90 min; MS (ESI^+^): *m*/*z* = 381 [M + H]^+^. ^1^H NMR (400 MHz, DMSO-*d*_6_) δ ppm: 1.10 (t, *J* = 7.03 Hz, 3H), 1.84 (d, *J* = 1.01 Hz, 3H), 3.87 (q, *J* = 6.97 Hz, 2H), 5.42 (s, 2H), 7.25 (td, *J* = 9.57, 1.77 Hz, 1H), 7.64 (ddd, *J* = 8.93, 7.54, 5.83 Hz, 1H), 7.79 (q, *J* = 1.14 Hz, 1H), 10.75 (s, 1H). HRMS (ESI, [M + H]^+^): calcd for C_16_H_15_F_2_N_4_O_3_S: 381.0827, found: 381.0826.

### Computational Modeling Procedures

Due to the lack of a high-resolution crystal structure of HSD17B13, we generated a homology model for SAR explanation and compound design. An X-ray-structure of the close homolog HSD17B11 is available (pdb code: 1YB1); however, the substrate binding pocket in this structure is in a closed state. The same holds true for the Alphafold2 model of HSD17B13, making it not useful for our purposes. Therefore, we decided to employ an estradiol/NADP^+^ bound structure (pdb code: 1FDU)^75^ of the more distantly related HSD17B1 as template because both, B1 and B13, bind estradiol/estrone as substrates, yielding a fair chance of a rather accurate model of the ligand binding site. Based on the sequence alignment (see Supporting Information, Figure S2), we generated the homology model using MOE^36^ (version 2020.09) with standard settings, followed by a constrained minimization (backbone fixed) using the Amber10:EHT force field to resolve potential remaining strains in the structure. The difference in the co-factors of template and target (NADP^+^ vs NAD^+^) is not regarded as an issue in model building as the phosphate pocket is solvent exposed, not being composed of conserved structural elements. In particular, the phosphate binding pocket is not conserved between B1 and B13, explaining the co-factor specificity for the isoforms. Compound **45** was optimized by DFT (ωB97XD/cc-pVDZ, with Gaussian16), and its lowest energy conformer was manually placed into the binding site so that the phenol points toward the catalytical triad of HSD17B13 and the northern part is facing the solvent, reflecting the observed SAR. A final constrained energy minimization in MOE^36^ led to the binding mode shown in Figure 11.

### Protein Production

Full-length human HSD17B13 (Uniprot ID: Q7Z5P4), human HSD17B11 (Uniprot ID: Q8NBQ5) and mouse HSD17B13 (Uniprot ID: Q8VCR2) were recombinantly expressed with C-terminal Histidine-tag in HEK293 cells. Expression was performed at Immunoprecise, Netherlands. Cell pellets were lysed in 25 mM Tris pH 7.5, 500 mM NaCl, 10 mM Imidazole, 0.5 mM TCEP, 5% Glycerin, 0.3% Triton-X supplemented with EDTA-free Complete Protease Inhibitor (Roche) and DNase I (Roche) by sonification. After centrifugation at 50.000 rpm at 4 °C for 1 h, the supernatants containing the HSD17 proteins were purified by Nickel affinity chromatography on a HisTrap column (Cytiva) in 25 mM Tris pH 7.5, 500 mM NaCl, 10 mM Imidazole, 0.5 mM TCEP, 0.01% LMNG and eluted by an imidazole gradient with a final concentration of 500 mM. Fractions containing the purified HSD17 proteins were further purified on a Superdex 200 size exclusion column (Cytiva) in PBS supplemented with 500 mM NaCl. The pure protein was then concentrated to the desired concentration using an Amicon filter devise with a cutoff of 10 kDa. Proteins were stored at −80 °C.

### Human HSD17B13 Enzyme Activity Assay for High-Throughput Screening via MALDI-TOF MS

Enzymatic reactions were set up in assay buffer containing 100 mM TRIS pH 7.5, 100 mM NaCl, 0.5 mM EDTA, 0.1 mM TCEP, 0.05% BSA, and 0.001% Tween20. First, 50 nL of test compound (final concentration: 5 or 50 μg/mL) or DMSO was placed into the wells of a 1536-well assay plate using a CyBio Well vario (Analytik Jena, Jena, Germany) liquid handling unit equipped with a capillary head. For dose–response experiments, 8-fold dilution series of compound solutions were prepared in DMSO in 1:3.16 dilution steps starting from 10 mM or 5 mg/mL stock solutions, respectively. Next, 2.5 μL of 2× concentrated human recombinant HSD17B13 enzyme in assay buffer (final concentration: 50 nM, columns 1–46) or plain assay buffer (columns 47 + 48) were added by a Certus Flex Micro Dispenser (Gyger, CH). The plates were then incubated for 10 min in a humidified incubator at 24 °C. Subsequently, 2.5 μL of substrate mixture (final concentration: estradiol 30 μM and NAD^+^ 0.5 mM, row 1–48) were added to each well. The reactions were mixed for 30 s at 1000 rpm and subsequently incubated for 40 min in a humidified incubator at 24 °C. After incubation, the enzymatic reaction was stopped and derivatization initiated by adding 1 μL of internal standard *d*_4_-estrone (final concentration: 0.7 μM) together with 2 μL of Girard’s Reagent P (final concentration: 12.5 mM, dissolved in methanol:formic acid 9:1 v/v). Dispensing steps were executed with the aid of a Certus Flex Micro Dispenser (Gyger, CH). The plates were then sealed with an adhesive foil, mixed for 30 s at 1000 rpm, and stored at room temperature until preparation of the MALDI target plates. Time for derivatization should be >10 h to assure full conjugate formation (in most cases plates were stored overnight prior to MALDI target-plate preparation). Each 1536-well assay plate contained high (no compound; columns 45–46) and low (no compound and assay buffer instead of enzyme; columns 47–48) controls to assess compound-related activity loss of the enzyme. Sample preparation was performed as described previously with slight modifications.^26^ Briefly, a saturated solution of α-cyano-4-hydroxycinnamic acid (HCCA) was prepared in 50% ACN and 50% water (TA50, v/v) containing 0.05% TFA. The CyBio Well vario liquid handling system (Analytik Jena, GER) equipped with ceramic tips and operated in 1536-well format was employed to conduct double-layer spotting providing highly homogeneous spot shapes. Here, 100 nL of matrix solution was spotted onto plain steel MALDI target plates and dried in a vacuum chamber. Subsequently, assay plates were centrifuged at 1000 rpm for 60 s and the seals were removed before 100 nL of matrix solution and 100 nL of sample were aspirated successively from the matrix reservoir and the assay plate, respectively, and dispensed together onto the dried matrix spots. The MALDI target plate was then dried under vacuum and stored until analysis. Finally, samples were washed on-target by transferring 0.3 μL of 0.1% TFA (v/v)/10 mM ammonium dihydrogenphosphate onto each spot with subsequent removal after 2 s of incubation.

#### MALDI-TOF-MS-Based Estrone Measurements and Data Analysis

Mass spectra were acquired with a rapifleX MALDI-TOF/TOF instrument from Bruker Daltonics including a Smartbeam 3D laser. FlexControl (v 4.0), FlexAnalysis (v4.0), and MALDI Pharma Pulse (v 2.2) were used for MS acquisition and data analysis. Target plates were loaded onto an Orbitor RS (Thermo Scientific) robotic system controlled by the laboratory automation software Momentum (v 4.2.3, Thermo Scientific) and automatically inserted into the MALDI-TOF device. Mass spectra were acquired in the mass range of *m*/*z* 380–500, respectively, to cover product and internal standard of the enzyme assay. Therefore, 1000 laser shots per sample spot were accumulated in positive ionization mode. The laser power was adjusted manually before every start of a batch process to reach a sufficient signal intensity for the internal standard. The acquired spectra were processed with a centroid peak detection set to a signal-to-noise ratio of *S*/*N* = 3 and a Gaussian smoothing (0.02 *m*/*z*; 1 cycle). Internal calibration was performed using the monoisotopic peak of the internal standard for the respective assays: [*d*_4_-estrone-GP]+ = 408.2584. MALDI-TOF data, processed with flexAnalysis or MALDI Pharma Pulse, were exported as a comma delimited (.csv) file. Datasets were further processed with either GraphPad Prism (v9.00; GraphPad Software, La Jolla, CA) or in-house laboratory information management system (LIMS) software. HSD17B13 activity was tracked by analyzing the measured intensity for the enzymatic product ([estrone-GP]+ = 404.2333) as well as for the corresponding internal standard ([*d*_4_-estrone-GP]+ = 408.2584). The signal ratio of the reaction product to the respective internal standard was calculated to diminish variations ascribed to the sample preparation and MALDI-TOF analysis. Average control values were calculated and set to 100% activity (high controls) and 0% activity (low controls) while the response values of compound-containing wells were normalized against the controls and expressed as percentage of control (PoC). The assignment of compounds to the corresponding measurements was achieved by software-aided deconvolution of every 1536-well assay plate to the corresponding 384-well substance plates. Determination of compound potencies was obtained by fitting the dose–response data to a four-parameter logistical equation.

### Enzyme Activity Assays for Compound Profiling and Mode of Inhibition Studies via RapidFire MS

All enzymatic reactions were performed in assay buffer containing 100 mM TRIS, 100 mM sodium chloride, 0.5 mM EDTA, 0.1% TCEP, 0.05% protease and fatty acid free BSA fraction V and 0.001% Tween20. Compounds were serially diluted in 100% DMSO and 50 nL spotted on a 384-well, PP, V-bottom microtiter plate (Greiner, Cat# 781280) by a Labcyte Echo 55× (1% DMSO final concentration in the assay). Experiments to select the high-throughput screening substrate were performed using commercially available recombinant human HSD17B13 (transcript variant A, OriGene cat# TP313132; final conc. 228 nM). Compound profiling and mode of inhibition studies were performed using purified, recombinant human HSD17B13 (final conc. 1 nM), human HSD17B11 (final conc. 35 nM) and mouse HSD17B13 (final conc. 50 nM) using estradiol (final conc. 30 μM) or LTB_4_ (in DMSO, final conc. 30 μM) as substrates and NAD^+^ (final conc. 0.5 mM for human HSD17B13 and human HSD17B11, 10 mM for mouse HSD17B13) as co-substrate. Compounds were also tested on the human HSD17B13 enzyme (final conc. 50 nM) using retinol (final conc. 30 μM; retinol stocks in 100% DMSO + 10% BHT prepared under anaerobic conditions in the BACTRON 900-2 anaerobic chamber). Substrate and co-substrate concentrations represent their experimentally determined *K*_m_ values. The assays were performed with the following protocol: 6 μL of diluted purified recombinant protein were added to each well of the compound-spotted microtiter plate and incubated for 15 min at room temperature (RT). After this incubation, 6 μL of substrate/co-substrate-mix were added to the compound-enzyme mix and incubated for 4 h at RT. Enzymatic reaction was stopped and analytes derivatized by adding 1 μL of analyte-specific internal standard (final. conc. 50 nM) and 2.4 μL of Girard’s Reagent P (GP) (final conc. 6.5 mM) dissolved in 90% Methanol and 10% formic acid to the reaction mixtures followed by an overnight incubation at RT. For the measurements of estrone, D_4_-estrone; for oxo-LTB_4_, arachidonic acid; and for retinal, D_6_-retinal were used as internal standards. To increase the sample volume, 70 μL of dH_2_O was added to the samples before the analyte levels had been measured via RapidFire MS.

#### RapidFire MS/MS-Based Estrone, Oxo-LTB_4_, and Retinal Measurements

The analytical sample handling was performed by a RapidFire autosampler system (Agilent, Waldbronn, Germany) coupled to a triple quadrupole mass spectrometer (Triple Quad 6500, AB Sciex Germany GmbH, Darmstadt, Germany). Liquid sample was aspirated by a vacuum pump into a 10 μL sample loop for 250 ms and subsequently flushed for 3000 ms onto a C18 cartridge for estrone and oxo-LTB_4_ (Agilent, Waldbronn, Germany) and a C4 cartridge for retinal with mobile phase A (for estrone: 99.9% water, 0.09% acetic acid, 0.01% TFA, flow rate 1.5 mL/min. For oxo-LTB_4_: 1 L water + 50 μL of 25% NH_3_, flow rate 1.5 mL/min. For retinal: 99.5% water, 0.49% acetic acid, 0.01% TFA, flow rate 1.5 mL/min). The analyte was backflushed from the cartridge for 3000 ms with mobile phase B (for estrone: 475 mL of methanol, 475 mL of ACN, 50 mL of water, 90 μL of acetic acid, 10 μL of TFA, flow rate 1.25 mL/min. For oxo-LTB_4_: 475 mL of methanol, 475 mL of acetonitrile, 50 mL of water, 50 μL of 25% NH_3_, flow rate 1.5 mL/min. For retinal: 49.75% methanol, 49.75% acetonitrile, 0.49% acetic acid, 0.01% TFA, flow rate 1.25 mL/min) and flushed into the mass spectrometer for detection in MRM mode. The MRM transition for estrone-GP was *Q*1/*Q*3: 404.1/157.1 Da (declustering potential 27 V, collision energy 43 V) and for the internal standard D_4_-estrone-GP *Q*1/*Q*3: 408.1/159.1 Da (declustering potential 27 V, collision energy 43 V). The mass spectrometer was operated in positive ionization mode (curtain gas 35 Au, collision gas medium, ion spray voltage 4200 V, temperature 550 °C, ion source gas 1 65 Au, ion source gas 2 80 Au). The MRM transition for the oxo-LTB_4_ was *Q*1/*Q*3: 336/195.1 Da (declustering potential 27 V, collision energy 43 V) and for the internal standard arachidonic acid *Q*1/*Q*3: 303.2/259.3 Da (declustering potential 27 V, collision energy 43 V). The MRM transition for retinal-GP3 was *Q*1/*Q*3: 418.3/94.9 (declustering potential 10 V, collision energy 23 V) and for the internal standard D_6_-retinal-GP *Q*1/*Q*3: 424.3/136.9 (declustering potential 66 V, collision energy 39 V). Dwell time for all analytes and each MRM transition was 25 ms and pause time between MRMs was 5 ms. For oxo-LTB_4_ and retinal: the mass spectrometer was operated in negative ionization mode (curtain gas 35 Au, collision gas medium, ion spray voltage 4200 V, temperature 550 °C, ion source gas 1 65 Au, ion source gas 2 80 Au). The solvent delivery setup of the RapidFire system consists of two continuously running and isocratically operating HPLC pumps (G1310A, Agilent, Waldbronn, Germany) and one binary HPLC pump channel B (G4220A, Agilent, Waldbronn, Germany).

#### Data Analysis

MS data processing was performed in GMSU (Alpharetta, GA), and peak area ratio analyte/internal standard was reported for IC_50_ calculation. Area under the curve values were uploaded to our data analysis software (Megalab Software, in-house development) and peak area ratios were calculated (analyte/internal standard). We normalized the peak area ratio data by assigning negative control values (all assay components) to 100% and positive control values (no enzyme) to 0%. For IC_50_ determination, we used a four-parameter logistical equation. *K*_i_ values were calculated using Morrison equation for tight binding (GraphPad Prism 9.3.1, GraphPad Software, San Diego, California). Based on repeated independent measurements (*n* = 58) of an internal assay reference, the IC_50_ values determined in the hHSD17B13 enzyme assay showed a variability of a factor of ±2.2.

#### Mode of Inhibition Studies

The *K*_m_ value of NAD^+^ on human HSD17B13 was experimentally determined (*K*_m_ = 1.4 ± 0.2 mM; *n* = 3) in the hHSD17B13 enzyme assay. To elucidate the mode of inhibition, compounds were tested in a dose-responsive manner at [NAD^+^]/*K*_m_ ratios ranging from 0.05 to 3, keeping the substrate estradiol constant at the highest practically achievable concentration of 100 μM. IC_50_ values had been plotted as a function of the ratio [NAD^+^]/*K*_m_ and the curve pattern of logistic regressions had been interpreted accordingly.^49,60−62^

### Cellular Human HSD17B13 Activity Assay and Cell Viability

Custom-made stably overexpressing hHSD17B13-Myc/DDK HEK293 cells (LakePharma, Inc.) and estradiol were prepared in serum free Dulbecco’s modified Eagle’s medium (DMEM) medium containing 10% heat inactivated FBS, 1× Glutamax, and 1× sodium pyruvate; 24 h prior to compound testing, 25 μL of a 0.4 × 10^6^ cells/mL dilution were seeded on 384-well Microplate (culture plate, PerkinElmer, Cat# 6007680). Compounds were serially diluted in 100% DMSO and 50 nL of the compound dilution were spotted on the preseeded cell plate by a Labcyte Echo 55× (1% DMSO in the Assay) and incubated for 30 min at 37 °C in a humidified incubator (rH = 95%, CO_2_ = 5%). After that incubation step, 25 μL of a 60 μM estradiol dilution were added to each well of the microtiter plate and incubated for 3 h at 37 °C in the humidified incubator. Finally, 20 μL of supernatant were taken and 2.5 μL of *d*_4_-estrone as an internal standard (final conc. 50 nM) were added. To derivatize the analytes, 5 μL of Girard’s Reagent P (6.5 mM final) dissolved in 90% methanol and 10% formic acid were added to the samples followed by overnight incubation at RT before adding 60 μL of dH_2_O to increase the sample volume for the RapidFire MS/MS measurements of estrone levels as described previously. To exclude impact on cell viability as cause for reduced estrone levels, we also performed a CellTiter-Glo Luminescent Cell Viability Assay (CellTiter-Glo, Promega, Cat# G9242) with the remaining cell samples of the initial assay plate, according to manufacturer protocol. Luminescence was measured using a PHERAstar FSX (BMG Labtech, Ortenberg, Germany).

#### Data analysis

Raw data were normalized by assigning negative control values (all assay components) to 100% and positive control values (no cells) to 0%. For IC_50_ determination, we used a four-parameter logistical equation.

### Differential Scanning Fluorimetry (nanoDSF)

The Prometheus NT.48 instrument (NanoTemper Technologies) was used to determine the melting temperatures. hHSD17B13 (10 μM) was preincubated with NAD^+^ (final conc. 0, 0.5 or 5 mM) and DMSO (final conc. of 2%) or compound **45** (final conc. 100 μM). The capillaries were filled with 10 μL of sample and placed on the sample holder. Four independent measurements were performed for each sample. A temperature gradient of 1 °C·min^–1^ from 25 to 95 °C was applied, and the intrinsic protein fluorescence at 330 and 350 nm was recorded. The melting point was determined as the maximum of the first derivative of the melting curve.

### DMPK Assays

#### Metabolic Stability in Liver Microsomes

The metabolic degradation of the test compound was assayed at 37 °C with pooled liver microsomes. The final incubation volume of 60 μL per time point contained TRIS buffer pH 7.6 at RT (0.1 M), magnesium chloride (5 mM), microsomal protein (0.5–2 mg/mL), and the test compound at a final concentration of 1 μM. Following a short preincubation period at 37 °C, the reactions were initiated by addition of β-nicotinamide adenine dinucleotide phosphate, reduced form (nicotinamide adenine dinucleotide phosphate (NADPH), 1 mM), and terminated by transferring an aliquot into solvent after different time points. The quenched incubations were pelleted by centrifugation (10,000*g*, 5 min). An aliquot of the supernatant was assayed by LC-MS/MS for the amount of remaining parent compound. The half-life was determined by the slope of the semilogarithmic plot of the concentration–time profile. The intrinsic clearance (CL_int_) was calculated by considering the amount of protein in the incubation: CL_int_ [μL/min/mg protein] = (Ln 2/(half-life [min] × protein content [mg/mL])) × 1000.

#### Metabolic Stability in Human/Mouse Hepatocytes

An assay in human hepatocytes was performed to assess the metabolic stability of compounds. The metabolic degradation of a test compound was assayed in a human and mouse hepatocyte suspension. After recovery from cryopreservation, hepatocytes were diluted in DMEM (supplemented with 3.5 μg glucagon/500 mL, 2.5 mg insulin/500 mL, 3.75 mg hydrocortisone/500 mL, 5% species serum) to obtain a final cell density of 1.0 × 10^6^ cells/mL or 4.0 × 10^6^ cells/mL, depending on the metabolic turnover rate of the test compound. Following a 30 min preincubation in a cell culture incubator (37 °C, 10% CO_2_), test compound solution was spiked into the hepatocyte suspension, resulting in a final test compound concentration of 1 μM and a final DMSO concentration of 0.05%. The cell suspension was incubated at 37 °C (cell culture incubator, horizontal shaker) and samples were removed from the incubation after 0, 0.5, 1, 2, 4, and 6 h. Samples were quenched with acetonitrile (containing internal standard) and pelleted by centrifugation. The supernatant was transferred to a 96-deep well plate, and prepared for analysis of decline of parent compound by HPLC-MS/MS. The percentage of remaining test compound was calculated using the peak area ratio (test compound/internal standard) of each incubation time point relative to the time point 0 peak area ratio. The log-transformed data were plotted versus incubation time, and the absolute value of the slope obtained by linear regression analysis was used to estimate *in vitro* half-life. *In vitro* intrinsic clearance (CL_int_) was calculated from *in vitro* half-life and scaled to whole liver using a hepatocellularity of 120 × 10^6^ cells/g liver, a human liver per body weight of 25.7 g liver/kg as well as *in vitro* incubation parameters. Hepatic *in vivo* blood clearance (CL) was predicted according to the well-stirred liver model considering an average liver blood flow (*Q*_H_) of species. Results were expressed as percentage of hepatic blood flow: *Q*_H_ [%] = CL [mL/min/kg]/hepatic blood flow [mL/min/kg].

#### Plasma Protein Binding (Human and Mouse), Tissue Binding

Equilibrium dialysis (ED) technique with Dianorm Teflon dialysis cells (micro 0.2) was used to determine the approximate *in vitro* fractional binding of test compounds to plasma proteins. Each cell consists of a donor and an acceptor chamber, separated by an ultrathin semipermeable membrane with a 5 kDa molecular weight cutoff. Stock solutions for each test compound were prepared in DMSO at 1 mM and diluted to a final concentration of 1.0 μM. The subsequent dialysis solutions were prepared in pooled human or mouse plasma (with NaEDTA) from male and female donors. Aliquots of 200 μL dialysis buffer (100 mM potassium phosphate, pH 7.4) were dispensed into the buffer chamber. Aliquots of 200 μL test compound dialysis solution were dispensed into the plasma chambers. Incubation was carried out for 2 h under rotation at 37 °C. At the end of the dialysis period, the dialysate was transferred into reaction tubes. The tubes for the buffer fraction contained 0.2 mL of ACN/water (80/20). Aliquots of 25 μL of the plasma dialysate were transferred into deep well plates and mixed with 25 μL of ACN/water (80/20), 25 μL of buffer, 25 μL of calibration solution, and 25 μL of Internal Standard solution. Protein precipitation was done by adding 200 μL of ACN. Aliquots of 50 μL of the buffer dialysate were transferred into deep well plates and mixed with 25 μL of blank plasma, 25 μL of Internal Standard solution, and 200 μL of ACN. Samples were measured on HPLC-MS/MS–Systems and evaluated with Analyst-Software to determine plasma protein binding (PPB [%]) and fraction unbound in the donor chamber. Tissue binding was determined via equilibrium dialysis in analogy to PPB in mouse liver, kidney, and lung tissue homogenates (0.165 mg/mL tissue in PBS pH 7.4) at a test compound concentration of 1 μM.

#### Caco-2 Permeability

For the measurement of permeability across polarized, confluent human cancer colon carcinoma cells 2 (Caco-2), cell monolayers grown on permeable filter supports were used as an *in vitro* absorption model. Apparent permeability coefficients (PE) of the compounds across the Caco-2 monolayers were measured (pH 7.2, 37 °C) in apical-to-basal (AB) (absorptive) and basal-to-apical (BA) (secretory) transport direction. Identical or similar permeabilities in both transport directions indicate passive permeation, vectorial permeability points to additional active transport mechanisms. Caco-2 cells (1–2 × 10^5^ cells/cm^2^ area) are seeded on filter inserts (Costar transwell polycarbonate or PET filters, 0.4 μm pore size) and cultured (DMEM) for 10–25 days. Compounds were dissolved in appropriate solvent (DMSO, 10 mM stock solutions). Stock solutions were diluted with HTP-4 buffer (128.13 mM NaCl, 5.36 mM KCl, 1 mM MgSO_4_, 1.8 mM CaCl_2_, 4.17 mM NaHCO_3_, 1.19 mM Na_2_HPO_4_·7H_2_O, 0.41 mM NaH_2_PO_4_·H_2_O, 15 mM HEPES, 20 mM glucose, pH 7.2) to prepare the transport solutions (10 μM compound, final DMSO ≤ 0.5%). The transport solution (TL) was applied to the apical or basolateral donor side for measuring A–B or B–A permeability (3 filter replicates), respectively. The receiver side contains HTP-4 buffer supplemented with 0.25% BSA. Samples were collected at the start and end of experiment from the donor and at various time intervals for up to 2 h also from the receiver side for concentration measurement by LC-MS/MS. Sampled receiver volumes were replaced with fresh receiver solution.

#### CYP Inhibition

The inhibition of the conversion of a specific substrate to its metabolite was assessed at 37 °C using human liver microsomes and to determine the inhibition of cytochrome P450 isoenzymes by a test compound. For the following cytochrome P450 isoenzymes, turnover of the respective substrates was monitored: CYP3A4: Midazolam; CYP2D6: Dextromethorphan; CYP2C8: Amodiaquine; CYP2C9: Diclofenac; CYP2C19: Mephenytoin; CYP2B6: Bupropion; CYP1A2: Tacrine. The final incubation volume contained TRIS buffer (0.1 M), MgCl_2_ (5 mM), human liver microsomes dependent on the P450 isoenzyme measured (ranging from 0.05 to 0.5 mg/mL), and the individual substrate for each isoenzyme (ranging from 1 to 80 μM). The effect of the test compound on substrate turnover was determined at five concentrations in duplicate (e.g., highest concentration 50 μM with subsequent serial 1:4 dilutions) or without test compound (high control). Following a short preincubation period, reactions were started with the co-factor (NADPH, 1 mM) and stopped by cooling the incubation down to 8 °C, followed by addition of one volume of acetonitrile. An internal standard solution is added after quenching of incubations. Peak area of analyte and internal standard is determined via LC-MS/MS. The resulting peak area ratio of analyte to internal standard in these incubations is compared to a control activity containing no test compound to determine the inhibitory IC_50_.

#### Mechanism-Based CYP3A4 Inhibition

Time-dependent inhibition of CYP3A4 was assessed in human liver microsomes using midazolam as a substrate. The test compounds and water control (wells without test compound) were preincubated in the presence of NADPH (1 mM) with human liver microsomes (0.2 mg/mL) at a concentration of 0, 5 and 25 μM for 0, 10 and 30 min. After preincubation, the incubate was diluted 1:10 (to 0.02 mg/mL) and the substrate midazolam (15 μM) was added for the main incubation (10 min). The main incubation was quenched with acetonitrile, and the formation of hydroxy midazolam was quantified via LC/MS-MS. The formation of hydroxy midazolam from the 30 min preincubation relative to the formation from the 0 min preincubation was used as a readout. Values of less than 100% mean that the substrate midazolam is metabolized to a lower extent upon 30 min preincubation compared to 0 min preincubation. In general, low effects upon 30 min preincubation are desirable.

#### UGT Phenotyping

Microsomes from baculovirus insect cells expressing human UGT isoforms (Supersomes) were obtained from Corning GmbH, Germany. These included microsomes from baculovirus-infected insect cells expressing UGT 1A1, 1A3, 1A4, 1A6, 1A9, 2B7, and 2B15 Supersomes (total protein content 5 mg/mL) prepared from cells without the human liver UGT cDNA insert. Microsomes were stored at −80 °C until used for experiments. Microsomal preparations were diluted in 0.1 M TRIS pH 7.4 buffer to a final protein concentration of 1 mg/mL. Compound solutions with a final concentration of 10 μM were prepared from 1 mM DMSO stock solution in double-distilled H_2_O. UDPGA (25 mM) (uridine-diphosphate-glucuronic acid), 50 mM Saccharolacton, and 50 μg/mL Alamethicin stock solutions were prepared in double-distilled H_2_O. For the experiment, 10 μL of 1 mg/mL microsomal preparations, 27.5 μL of H_2_O, 22.5 μL of 400 mM TRIS pH 7.5/40 mM MgCl_2_ buffer, 10 μL of Saccharolacton stock solution, and 10 μL of Alamethicin stock solution were mixed and preincubated for 5 min at 4 °C. Subsequently, 10 μL of 10 μM compound solution and 10 μL of 25 mM UDPGA solution were added to achieve a total of 100 μL incubation volume. The reaction was started by heating to 37 °C and stopped after 15 and 30 min, respectively, by cooling to 4 °C and adding 50 μL of 33% ACN in H_2_O. Samples were centrifuged at 4000 rpm, 4 °C, and supernatants were transferred to 96-well plates for quantification of parent compound depletion and glucuronide formation via HPLC-MS/MS.

### *In Vivo* Pharmacokinetics

#### Pharmacokinetics in Mice and Rats

Pharmacokinetic studies were performed in male C57BL6/N mice (Janvier, France; mean body weight 25 g) to evaluate the pharmacokinetic properties and tissue distribution of test compounds. A compound suspension (0.5% Natrosol solution with 0.015% Tween-80) was dosed orally by gavage or subcutaneously to mice at a dose of 50 or 80 μmol/kg, respectively. Blood samples from mice (20 μL) were taken via puncture of the hindleg vein (*V. saphena*) at several time points post application, anticoagulated, and centrifuged. Compound distribution to the liver was determined in separate studies at multiple terminal timepoints following oral administration. Plasma and tissue samples were stored at −20 °C prior to bioanalysis. For bioanalysis, plasma protein was precipitated with acetonitrile. Tissue samples were transferred to Precellys vials, and three parts of acetonitrile/methanol (1:1) and one part of acidified water were added for glucuronide stabilization prior to homogenization. Homogenates were centrifuged and supernatant was collected for bioanalysis. The concentration of the administered compound in plasma and tissue samples was quantified via high-performance liquid chromatography coupled with tandem mass spectrometry. In addition, pharmacokinetics following intravenous injection to the tail vein (solution with 20% HP-ß-cyclodextrin, 5 μmol/kg) were conducted in male C57BL6/N mice as well as male Han Wistar rats (Janvier, France; mean body weight 270 g) accordingly. Blood samples from rats (50 μL) were taken via puncture of the sublingual vein in short-term isoflurane anesthesia. Tissue distribution was determined terminally 1 h after administering a second intravenous dose. Pharmacokinetic parameters (AUC, oral bioavailability, *V*_ss_, Clearance) were calculated using noncompartmental analysis methods.

#### Bile Excretion Studies in Rats

Bile excretion studies were conducted in male Han Wistar rats (Janvier, France; mean body weight 270 g). Studies were performed under constant intratracheal intubation anesthesia using isoflurane over 6 h. Vital signs (breathing, body temperature, heart rate, blood pressure) were monitored. A constant rate infusion of ringer-acetate solution (2 mL/h) was applied via catheterization of the *V. jugolaris* to maintain physiologic hydration over the course of the study. After surgical incision of the abdominal cavity, the bile duct (*Ductus choledochus*) was ligated, catheterized, and fixated. The compound was delivered intravenously at 5 μmol/kg (solution with 20% HP-ß-cyclodextrin) to the tail vein. Subsequently, bile flow was collected in constantly cooled (4 °C) picovials in 1 h intervals over the study duration of 6 h. At the end of the study, the animals were euthanized. For the quantitative assessment of parent compound and corresponding glucuronide/sulfate conjugate biliary excretion, 5 μL of bile sample was incubated with 5 μL of ammonium acetate buffer in the presence and absence of *Helix Pomatia*-ß-Glucuronidase (≥100,000 units/mL, 1:4 dilution in buffer) for 1 h at 37 °C under constant shaking. As a positive control for enzymatic conjugate cleavage, 20 μM phenolphthalein-glucuronide and phenolphthalein-sulfate solutions in rat bile were incubated in parallel, resulting in a complete turnover to phenolphthalein after 1 h. The enzymatic digestion was stopped by adding 150 μL of ACN containing internal standard. All samples were centrifuged, and 100 μL of 0.1% HCOOH solution was added to 20 μL of supernatant and quantified via HPLC-MS/MS. All animal experiments were approved by the local German authorities (Regierungspräsidium Tübingen, Baden-Württemberg, Germany) and conducted in compliance with the German and European Animal Welfare Acts.

### Physicochemical Assays

#### Determination of Water Solubility from DMSO Stock Solutions

The aqueous solubility of the test compound was determined by comparing the amount dissolved in buffer to the amount in an acetonitrile/water (1:1) solution. Starting from a 10 mM DMSO stock solution, aliquots were diluted with acetonitrile/water (1:1) or buffer, respectively. After 24 h of shaking, the solutions were filtrated and analyzed by LC-UV. The amount dissolved in buffer was compared to the amount in the acetonitrile solution. Solubility will usually be measured from 0.001 to 0.125 mg/mL at a DMSO concentration of 2.5%. If more than 90% of the compound is dissolved in buffer, the value is marked with “>”.

## Supporting Information Available

HTS results; compound syntheses of **2**–**44** and **46**–**49**; selectivity panel data for **45** (**BI-3231**); sequence alignment for homology modeling; UPLC traces; and NMR spectra of key compounds (PDF)
Coordinates of the homology model (PDB)
Molecular formula strings (CSV)

## Supplementary Material

jm2c01884_si_001.pdf

jm2c01884_si_002.pdb

## Abbreviations Used

AAV: adeno-associated virus
ALT: alanine transaminase
AUC: area under the curve
AUC_DN_: dose-normalized AUC
BHT: butylhydroxytoluol
CTL: control
CYP: cytochrome P450
DDI: drug–drug interaction
DMPK: drug metabolism and pharmacokinetics
DSF: differential scanning fluorimetry
ESLD: end-stage liver disease
FA: formic acid
*f*_u,plasma_: fraction unbound in plasma
GP: Girard’s Reagent P
GWAS: genome-wide association study
Hep: hepatocytes
hERG: human ether-a-go-go-related gene
HSD17B11: 17-β hydroxysteroid dehydrogenase 11
HSD17B13: 17-β hydroxysteroid dehydrogenase 13
HTS: high-throughput screening
IPA: isopropyl alcohol
LE: ligand efficiency
LipE: lipophilic efficiency
LM: liver microsomes
MALDI-TOF-MS: matrix-assisted laser desorption/ionization–time-of-flight mass spectrometry
MBI: mechanism-based CYP inhibition
MedChem: Medicinal Chemistry
MetID: metabolite identification
Met. Stab: metabolic stability
NAD: nicotinamide adenine dinucleotide
NAFLD: nonalcoholic fatty liver disease
NASH: nonalcoholic steatohepatitis
nd: not determined
OAT: organic anion transporter
OATP: organic anion transporting polypeptide
PD: pharmacodynamics
PK: pharmacokinetics
PPB: plasma protein binding
PROTAC: Proteolysis Targeting Chimera
QH: hepatic blood flow
SAR: structure–activity relationship
SD: standard deviation
SDR: short-chain dehydrogenase/reductase
TB: tissue binding
TFA: trifluoroacetic acid
