# Supplementary material for: Discovery of a Novel Potent and Selective HSD17B13 Inhibitor, BI-3231, a Well-Characterized Chemical Probe Available for Open Science
Source: J Med Chem. 2023 Feb 2;66(4):2832–50. doi: 10.1021/acs.jmedchem.2c01884 (PMC9969402; doi:10.1021/acs.jmedchem.2c01884)
Supplement: Supplementary file 1 — jm2c01884_si_001.pdf [file jm2c01884_si_001.pdf]

Supporting Information

Discovery of a novel potent and selective HSD17B13 Inhibitor, BI-3231, a well-characterized Chemical Probe available for Open Science

Sven Thamm,[1*] Marina K. Willwacher,[1*] Gary E. Aspnes,[1] Tom Bretschneider,[1] Nicholas F. Brown,[2] Silke Buschbom-Helmke,[1] Thomas Fox,[1] Emanuele M. Gargano,[1] Daniel Grabowski,[1] Christoph Hoenke,[1] Damian Matera,[2] Katja Mueck,[1] Stefan Peters,[1] Sophia Reindl,[1] Doris Riether[1] Matthias Schmid,[1] Christofer S. Tautermann,[1] Aaron M. Teitelbaum,[1] Cornelius Trünkle,[1] Thomas Veser,[1] Martin Winter,[1] and Lars Wortmann[1*]

[1]Boehringer Ingelheim Pharma GmbH & Co. KG, 88397 Biberach an der Riß, Germany

[2]Boehringer Ingelheim Pharmaceuticals, Inc., 900 Ridgebury Road, PO Box 368, Ridgefield, CT 06877-0368, USA

*Corresponding Authors. Email addresses: (S. T.) Sven.Thamm@boehringer-ingelheim.com, (M. K. W.) Marina.Willwacher@boehringer-ingelheim.com, (L. W.) Lars.Wortmann@boehringer-ingelheim.com

Content: HTS results; compound syntheses of **2-44** and **46-49**; selectivity panel data for **45** (**BI-3231**); sequence alignment for homology modelling; UPLC traces and NMR spectra for key compounds.

Table of Contents

1. High-throughput Screening Results S3
2. Compound Synthesis S4
   2.1. General Methods and Materials S4
   2.2. HPLC-MS methods S5
   2.3. Synthesis of Compounds **2-11** S8
   2.4. Synthesis of Compounds **12-22** S16
   2.5. Synthesis of Compounds **23-38** S29
   2.6. Synthesis of Compounds **39-44** and **46-48** S39
   2.7. Synthesis of Negative Probe **49** S47
3. Selectivity Panel Data for **45** (**BI-3231**) S48
4. Sequence Alignment for Homology Modelling S49
5. References S50
6. UPLC traces for key compounds S50
7. ^{1}H NMR spectra for key compounds S61

1. High-throughput Screening Results

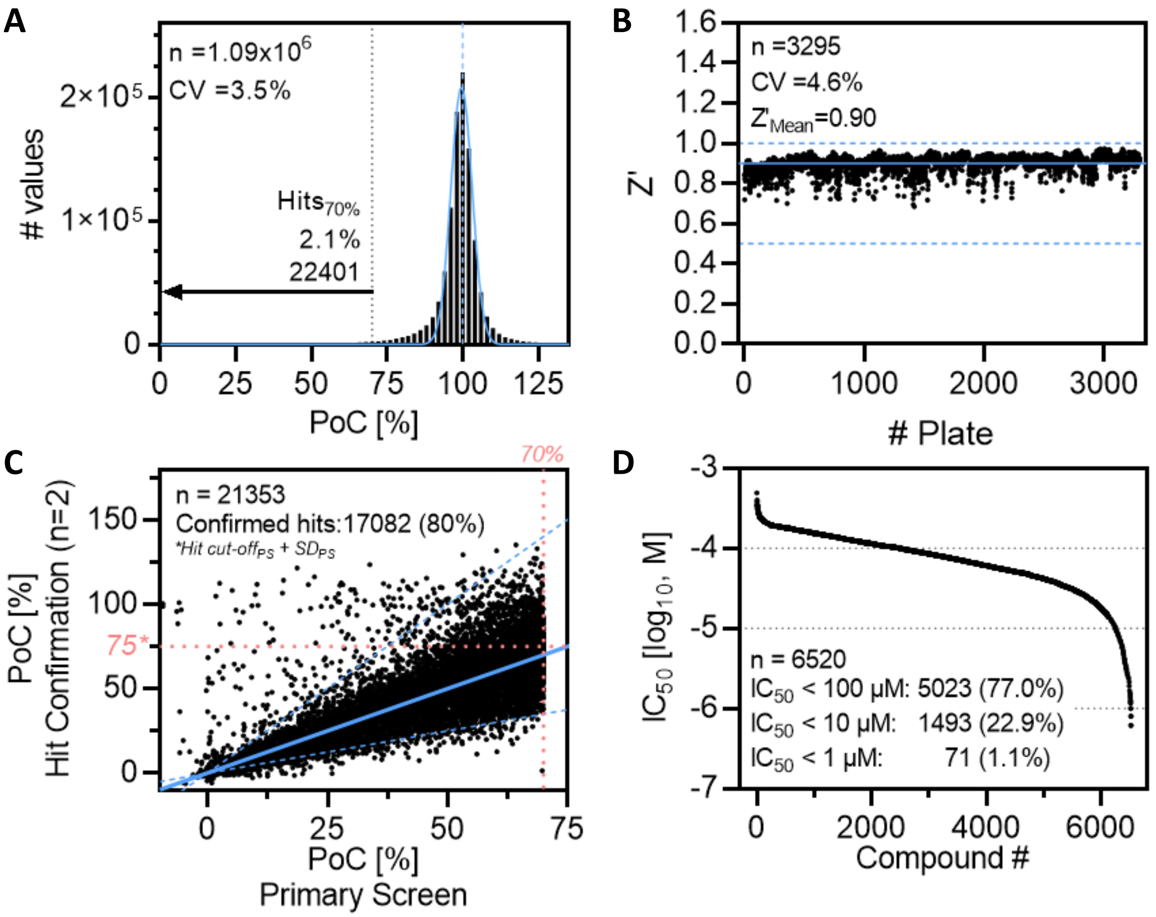

Figure S1. Results from the full-diversity screening campaign for novel HSD17B13 inhibitors. (**A**) Histogram for the single-dose experiments of the primary screening campaign. In total, 1.09 million compounds were tested, and a hit rate of 2.1% (total: 22,401 compounds) was observed when applying a hit threshold of 70% residual enzyme activity being indicated by the dashed grey line. (**B**) Assay quality parameter Z' was monitored throughout the entire screening campaign. Each of the 3.295 compound plates (384-well plate containing 16 high and low controls each) stayed within the predefined quality threshold of $Z' \geq 0.5$. (**C**) Confirmation of primary screening hits. The average PoC values of duplicate single-dose determinations in the hit confirmation experiments are plotted against PoC values from the primary screen. Compounds are considered as confirmed hits when they lead to an average residual enzyme activity below 75% (= hit cut-off$_{PS}$ + ~1xSD$_{PS}$). Overall, a confirmation rate of 80% (total: 17,082 compounds) was obtained. Two-fold deviation between runs is indicated by the blue dashed line and the solid line represents the line of identity. SD: standard deviation; PS: primary screen (**D**) Determination of compound potencies of selected hits from the screening campaign. The average IC_{50} values from two individual measurements are plotted in decreasing order.

2. Compound Synthesis

2.1. General Methods and Materials

All commercially available chemicals were used as received from their commercial supplier. Anhydrous solvents were either purchased or prepared according to standard procedures[S1] and stored over molecular sieves under argon. Unless stated otherwise, all reactions were carried out in oven-dried (at 120 °C) glassware under an inert atmosphere of argon. A Biotage Initiator Classic microwave reactor was used for reactions conducted in a microwave oven. Reactions were monitored by TLC on aluminum-backed plates coated with Merck Kieselgel 60 F 254 with visualization under UV light at 254 nm, and with HPLC-MS analysis (for HPLC-MS methods, *see Table S1*). Unless stated otherwise, crude products were purified by flash column chromatography on silica (using a Biotage IsoleraOne, Biotage IsoleraFour or CombiFlash® Teledyne Isco system) or by (semi)-preparative reversed-phase HPLC (Agilent Acquity or Waters instruments). Unless specified otherwise, the purity of all final compounds was determined to be ≥ 95% by LC-MS. Nuclear magnetic resonance (NMR) spectra were recorded at room temperature ((22 ± 1 °C), on a Bruker Avance 400 spectrometer with tetramethylsilane as an internal reference. Chemical shifts δ are reported in parts per million (ppm). ^{1}H NMR spectra were referenced to the residual partially non-deuterated solvent signal of DMSO (δ = 2.50 ppm). Coupling constants *J* are reported in Hz, and splitting patterns are described as br = broad, s = singlet, d = doublet, t = triplet, q = quartet, quin = quintet and m = multiplet. High-resolution mass spectra were recorded on a Thermo Scientific LTQ Orbitrap XL using electrospray ionization in positive ion mode (ESI+). MarvinSketch software version 20.19.1 was used to generate compound names.

2.2. HPLC-MS methods

Table S1. HPLC-MS methods

Method Name:	***Method 1***			
Device description:	Waters Acquity with DA- and MS-Detector			
Column:	Sunfire C18_3.0 × 30 mm_2.5 µm			
Column producer:	Waters			
Gradient/Solvent Time [min]	% Sol [Water 0.1% TFA (v/v)]	% Sol [Acetonitrile]	Flow [ml/min]	Temp [°C]
0.0	95.0	5.0	1.5	60.0
1.3	0.0	100.0	1.5	60.0
1.5	0.0	100.0	1.5	60.0
Method Name:	***Method 2***			
Device description:	Waters Acquity with DA- and MS-Detector			
Column:	Sunfire C18_2.1 × 30 mm_2.5 µm			
Column producer:	Waters			
Gradient/Solvent Time [min]	% Sol [Water 0.1% TFA (v/v)]	% Sol [Acetonitrile]	Flow [ml/min]	Temp [°C]
0.0	99.0	1.0	1.5	60.0
0.02	99.0	1.0	1.5	60.0
1.0	0.0	100.0	1.5	60.0
1.1	0.0	100.0	1.5	60.0
Method Name:	***Method 3***			
Device description:	Agilent 1200 with DA- and MS-Detector			
Column:	Sunfire C18_3.0 × 30 mm_2.5 µm			
Column producer:	Waters			
Gradient/Solvent Time [min]	% Sol [Water 0.1% TFA (v/v)]	% Sol [Acetonitrile]	Flow [ml/min]	Temp [°C]
0.0	97.0	3.0	2.2	60.0
0.2	97.0	3.0	2.2	60.0
1.2	0.0	100.0	2.2	60.0
1.25	0.0	100.0	3.0	60.0
1.4	0.0	100.0	3.0	60.0
Method Name:	***Method 4***			
Device description:	Waters Acquity-UPLC-SQ Detector-2			
Column:	AQUITY UPLC BEH C18_2.1 × 50 mm_1.7µm			
Column producer:	Waters			
Voltage:	Capillary Voltage 3.50 Kv cone voltage 50V, Disolvation gas 750 L/h, Disolvation Temp 350 °C			
MS mode	ESI			
Gradient/Solvent Time [min]	% Sol [Water 0.05% FA (v/v)]	% Sol [ACN 0.05% FA (v/v)]	Flow [ml/min]	Temp [°C]
0.0	98.0	2.0	0.6	40.0
0.4	98.0	2.0	0.6	40.0

2.3	98.0	2.0	0.6	40.0
3.4	98.0	2.0	0.6	40.0
3.5	2.0	98.0	0.6	40.0
4.0	2.0	98.0	0.6	40.0

Method Name:	***Method 5***			
Device description:	Agilent 1290 Infinity, Agilent SQD			
Column:	AQUITY UPLC BEH C18_2.1 × 50 mm_1.7µm			
Column producer:	Waters			
Voltage:	Capillary Voltage 3500, drying gas flow 10.0 mL/min, drying gas Temp 300-350 °C			
MS mode	ESI			
Gradient/Solvent Time [min]	% Sol [Water 0.1% FA (v/v)]	% Sol [ACN 0.1% FA (v/v)]	Flow [ml/min]	Temp [°C]
0.0	98.0	2.0	0.6	45.0
0.2	98.0	2.0	0.6	45.0
1.5	2.0	98.0	0.6	45.0
3.3	2.0	98.0	0.6	45.0
3.4	98.0	2.0	0.6	45.0
3.8	98.0	2.0	0.6	45.0

Method Name:	***Method 6***			
Device description:	Waters Acquity-UPLC-SQ Detector-2			
Column:	AQUITY UPLC BEH C18_2.1 × 50 mm_1.7µm			
Column producer:	Waters			
Voltage:	Capillary Voltag 3.50 Kv cone voltage 50V, Disolvation gas 750 L/h, Disolvation Temp 350 °C			
MS mode	ESI			
Gradient/Solvent Time [min]	% Sol [Water 0.05% FA (v/v)]	% Sol [ACN 0.05% FA (v/v)]	Flow [ml/min]	Temp [°C]
0.0	97.0	3.0	0.6	35.0
0.3	97.0	3.0	0.6	35.0
2.2	2.0	98.0	0.6	35.0
3.3	2.0	98.0	0.6	35.0
4.5	2.0	98.0	0.6	35.0
4.51	97.0	3.0	0.6	35.0

Method Name:	***Method 7***			
Device description:	Waters Acquity, QDa Detector			
Column:	Sunfire C18_3.0 x 30 mm_2.5 µm			
Column producer:	Waters			
Gradient/Solvent Time [min]	% Sol [Water 0.1% TFA (v/v)]	% Sol [ACN 0.08% TFA (v/v)]	Flow [ml/min]	Temp [°C]
0.0	95.0	5.0	1.5	60.0
1.3	0.0	100.0	1.5	60.0
1.5	0.0	100.0	1.5	60.0
1.6	95.0	5.0	1.5	60.0

Method Name:	***Method 8***
Device description:	Waters Acquity, QDa Detector
Column:	XBridge C18_3.0 x 30 mm_2.5 µm

Column producer:	Waters			
Gradient/Solvent Time [min]	% Sol [Water 0.1% NH_3 (v/v)]	% Sol [ACN]	Flow [ml/min]	Temp [°C]
0.0	95.0	5.0	1.5	60.0
1.3	0.0	100.0	1.5	60.0
1.5	0.0	100.0	1.5	60.0
1.6	95.0	5.0	1.5	60.0

Method Name:	***Method 9***			
Device description:	Agilent 1200 with DA- and MS-Detector			
Column:	XBridge C18_3.0 × 30 mm_2.5 µm			
Column producer:	Waters			
Gradient/Solvent Time [min]	% Sol [Water 0.1% NH_3 (v/v)]	% Sol [ACN]	Flow [ml/min]	Temp [°C]
0.0	97.0	3.0	2.2	60.0
0.2	97.0	3.0	2.2	60.0
1.2	0.0	100.0	2.2	60.0
1.25	0.0	100.0	3.0	60.0
1.4	0.0	100.0	3.0	60.0

Mobile phase preparations

Examples:

- The mobile phase “Water 0.1% TFA (v/v)” is prepared by adding 1 ml of a commercially available TFA solution to 999 ml water.
- The mobile phase “Water 0.1% NH3” is prepared by adding 4 ml of a commercially available concentrated ammonium hydroxide solution (25 wt%) to 996 ml water.

Syntax for column description

Description_Dimensions ID x length_Particle Size

Convention: Sections separated by underscores; blanks between numbers and unit; ID and length in mm, particle size in µm; ID and particle size always with one digit

e.g.: XBridge C18_4.6 x 50 mm_3.5 µm

2.3. Synthesis of Compounds 2-11

For a general synthetic strategy for compounds 1-11, see *Scheme 1* and *Experimental Section* in main text.

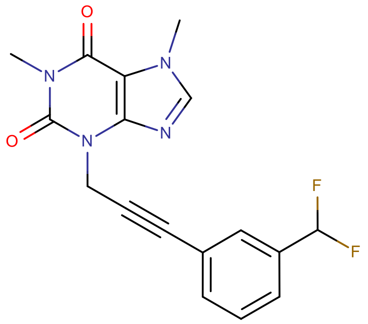

3-{3-[3-(difluoromethyl)phenyl]prop-2-yn-1-yl}-1,7-dimethyl-2,3,6,7-tetrahydro-1H-purine-2,6-dione (**2**). Compound **2** was prepared analogously to **1**, from **1B** (250 mg, 1.00 mmol) and 1-bromo-3-(difluoromethyl)benzene (356 mg, 2.00 mmol, commercially available, CAS-RN: [29848-59-7]) at 90 °C for 3 h. Work up and purification: the reaction mixture was diluted with EtOAc, filtered through a pad of Celite® and the filtrate was washed with water. The organic layer was dried over Na_2SO_4, filtered and concentrated. The residue was purified by flash column chromatography on silica gel (100-200 mesh, 45% EtOAc in petroleum ether) to yield compound **2** (105 mg, 27% yield). LC-MS (*method 4*): ^{t}R = 1.68 min; MS (ESI$^+$): *m/z* = 345 $[M+H]^+$. ^1H NMR (400 MHz, DMSO-d_6) δ ppm: 3.26 (s, 3 H), 3.90 (s, 3 H), 5.02 (s, 2 H), 7.00 (t, J = 55.7 Hz, 1 H), 7.47–7.54 (m, 1H), 7.54–7.61 (m, 3H), 8.06 (s, 1H). HRMS (ESI, $[M+H]^+$): calcd for $C_{17}H_{15}F_2N_4O_2$: 345.1158, found: 345.1157.

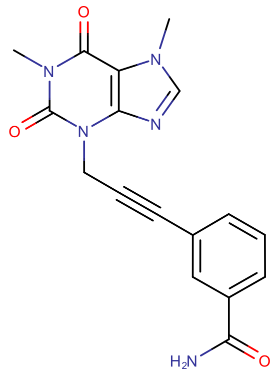

3-[3-(1,7-dimethyl-2,6-dioxo-2,3,6,7-tetrahydro-1H-purin-3-yl)prop-1-yn-1-yl]benzamide (**3**). Step 1: Methyl 3-[3-(1,7-dimethyl-2,6-dioxo-2,3,6,7-tetrahydro-1H-purin-3-yl)prop-1-yn-1-yl]benzoate (**S-3A**) was prepared analogously to **1**, from **1B** (200 mg, 0.92 mmol) and methyl 3-iodobenzoate (288 mg, 1.10 mmol, commercially available, CAS-RN: [618-91-7]) at 60 °C for 3 h. Work up and purification: The reaction mixture was cooled to rt and filtered through a pad of Celite®. The filtrate was concentrated, and the

residue was purified by flash column chromatography on silica gel (100-200 mesh, 50% EtOAc in petroleum ether) to yield compound **S-3A** (200 mg, 62% yield). LC-MS (*method 4*): ^{t}R = 1.62 min; MS (ESI^{+}): *m/z* = 353 [M+H]$^{+}$. Benzoate **S-3A** was used in the next step. Step 2: To a stirred solution of benzoate **S-3A** (100 mg, 0.28 mmol) in THF (15 mL) and water (5 mL) was added lithium hydroxide (18.0 mg, 0.43 mmol) and the resulting reaction mixture was stirred at rt for 3 h. The reaction mixture was concentrated under reduced pressure. The crude liquid was dissolved in water and acidified with aq. HCl (1N) to pH ~2, the resulting precipitate was filtered off and dried under vacuum to yield 3-[3-(1,7-dimethyl-2,6-dioxo-2,3,6,7-tetrahydro-1H-purin-3-yl)prop-1-yn-1-yl]benzoic acid (**S-3B**) as a white solid (0.06 g, 63% yield), which was used in the next step without further purification. LC-MS (*method 4*): ^{t}R = 1.41 min; MS (ESI^{+}): *m/z* = 339 [M+H]$^{+}$. Step 3: To a stirred ice-cold solution of acid **S-3B** (100 mg, 0.30 mmol) in DCM (5 mL) was added DMF (0.05 mL) and oxalyl chloride (56.0 mg, 0.44 mmol). The reaction mixture was allowed to slowly warm to rt and was stirred for 1 h. The mixture was concentrated under reduced pressure to provide 3-[3-(1,7-dimethyl-2,6-dioxo-2,3,6,7-tetrahydro-1H-purin-3-yl)prop-1-yn-1-yl]-benzoyl chloride (**S-3C**) as a crude liquid (0.12 g, quantitative yield). The crude product was used in the next step without further purification. Step 4: To a stirred ice-cold solution of acid chloride **S-3C** (0.12 g, 0.34 mmol) in DCM (5 mL) was added NH_3 (0.5M in THF, 5 mL). The reaction mixture was allowed to slowly warm to rt and was stirred for 1 h. The mixture was concentrated under reduced pressure and the residue was washed with diethyl ether and n-pentane to yield product **3** as a solid (60 mg, 53% yield). LC-MS (*method 4*): ^{t}R = 1.28 min; MS (ESI^{+}): *m/z* = 338 [M+H]$^{+}$. ^{1}H NMR (400 MHz, DMSO-d_6) δ ppm: 3.26 (s, 3 H), 3.90 (s, 3 H), 5.02 (s, 2 H), 7.41 (br d, *J* = 8.49 Hz, 1 H), 7.45 (br d, *J* = 7.48 Hz, 1 H), 7.54 (br d, *J* = 7.35 Hz, 1 H), 7.80–7.94 (m,

2H), 8.03 (br s, 1H), 8.07 (s, 1H). HRMS (ESI, $[M+H]^+$): calcd for $C_{17}H_{16}N_5O_3$: 338.1248, found: 338.1245.

N-{3-[3-(1,7-dimethyl-2,6-dioxo-2,3,6,7-tetrahydro-1H-purin-3-yl)prop-1-yn-1-yl]phenyl}acet-amide (**4**).

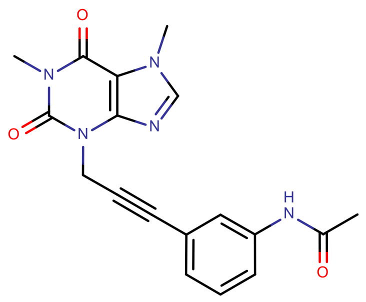

Step 1: 3-[3-(3-aminophenyl)prop-2-yn-1-yl]-1,7-dimethyl-2,3,6,7-tetrahydro-1H-purine-2,6-dione (**S-4A**) was prepared analogously to **1**, from **1B** (200 mg, 0.92 mmol) and 3-iodoaniline (301 mg, 1.37 mmol, commercially available, CAS-RN: [626-01-7]) at 60 °C for 3 h. Work up and purification: The reaction mixture was diluted with DCM, then filtered through a pad of Celite® and washed with 5% MeOH in DCM. The filtrate was partitioned between water and DCM. The organic layer was dried over Na_2SO_4, filtered and concentrated. The residue was purified by flash column chromatography (silica gel 100-200 mesh, 5% MeOH in DCM) to yield aniline **S-4A** (100 mg, 35% yield), which was used in the next step. Step 2: To an ice-cold stirred solution of **S-4A** (80.0 mg, 0.26 mmol) in DCM (3.00 mL) was dropwise added acetic anhydride (80.0 mg) and the reaction mixture was stirred at rt for 3 h. The mixture was diluted with DCM (20 mL) and washed with saturated aq. $NaHCO_3$ solution. The organic layer was dried over anhydrous Na_2SO_4 and concentrated under reduced pressure. The residue was purified by flash column chromatography (silica gel 100-200 mesh, 2-3% MeOH in DCM) to provide the pure product **4** (42 mg, 46% yield). LC-MS (*method 4*): ^tR = 1.38 min; MS (ESI^+): m/z = 352 $[M+H]^+$. ^1H NMR (400 MHz, DMSO-d_6) δ ppm: 2.02 (s, 3 H), 3.26 (s, 3 H), 3.90 (s, 3H), 4.99 (s, 2 H), 7.04 (dt, J = 7.67, 1.11 Hz, 1 H), 7.26 (t, J = 7.92 Hz, 1 H), 7.48 (dd, J = 8.24, 1.01 Hz, 1 H), 7.70 (s, 1H), 8.06 (s, 1H), 9.97 (s, 1H). HRMS (ESI, $[M+H]^+$): calcd for $C_{18}H_{18}N_5O_3$: 352.1404, found: 352.1406.

3-[3-(2-aminopyridin-4-yl)prop-2-yn-1-yl]-1,7-dimethyl-2,3,6,7-tetrahydro-1H-purine-2,6-dione

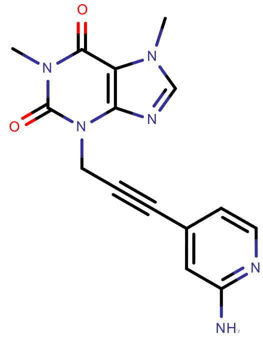

(**5**). Amino pyridine **5** was prepared analogously to **1**, from **1B** (250 mg, 1.00 mmol) and 4-iodopyridin-2-amine (302 mg, 1.00 mmol, commercially available, CAS-RN: [552331-00-7]) at 60 °C for 3 h. Work up and purification: The reaction mixture was diluted with DCM, filtered through a pad of Celite® and washed with 5% MeOH in DCM. The filtrate was partitioned between water and DCM. The organic layer was dried over Na_2SO_4, filtered and concentrated. The residue was purified by flash column chromatography (silica gel 100-200 mesh, 5% MeOH in DCM) to yield amino pyridine **5** (115 mg, 32% yield). LC-MS (*method 4*): ^{t}R = 1.05 min; MS (ESI$^+$): *m/z* = 311 [M+H]$^+$. ^{1}H NMR (400 MHz, DMSO-d_6) δ ppm: 3.25 (s, 3 H), 3.90 (s, 3H), 4.98 (s, 2 H), 6.01 (s, 2H), 6.38–6.42 (m, 2H), 7.85 (dd, J = 5.13, 0.82 Hz, 1 H), 8.06 (s, 1H). HRMS (ESI, [M+H]$^+$): calcd for $C_{15}H_{15}N_6O_2$: 311.1251, found: 311.1249.

1,7-dimethyl-3-[3-(2-oxo-1,2-dihydropyridin-4-yl)prop-2-yn-1-yl]-2,3,6,7-tetrahydro-1H-purine-2,6-dione

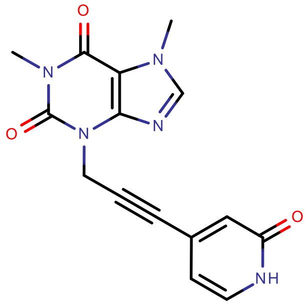

(**6**). Compound **6** was prepared analogously to **1**, from **1B** (250 mg, 1.00 mmol) and 4-bromo-1,2-dihydropyridin-2-one (299 mg, 2.00 mmol, commercially available, CAS-RN: [36953-37-4]) at 70 °C for 3 h. Work up and purification was carried out analogously to compound **5** giving the desired product **6** (130 mg, 37% yield). LC-MS (*method 5*): ^{t}R = 1.63 min; MS (ESI$^+$): *m/z* = 312 [M+H]$^+$. ^{1}H NMR (400 MHz, DMSO-d_6) δ ppm: 3.25 (s, 3 H), 3.90 (s, 3H), 5.00 (s, 2 H), 6.06 (br d, J = 3.80 Hz, 1H), 6.31 (br s, 1H),

7.33 (br d, $J = 4.44$ Hz, 1H), 8.06 (s, 1H), 11.66 (br s, 1H). HRMS (ESI, $[M+H]^+$): calcd for $C_{15}H_{14}N_5O_3$: 312.1091, found: 312.1089.

1,7-dimethyl-3-[3-(6-oxo-1,6-dihydropyridin-3-yl)prop-2-yn-1-yl]-2,3,6,7-tetrahydro-1H-purine-2,6-dione (**7**). Compound **7** was prepared analogously to **1**, from **1B** (250 mg, 1.00 mmol) and 5-iodo-1,2-dihydropyridin-2-one (304 mg, 1.00 mmol, commercially available, CAS-RN: [13472-79-2]) at 60 °C for 3 h. Work up and purification was carried out analogously to compound **5** giving the desired product **7** (90 mg, 25% yield). LC-MS (*method 4*): $^{t}R = 1.15$ min. MS (ESI$^+$): $m/z = 312$ $[M+H]^+$. ^1H NMR (400 MHz, DMSO-d_6) δ ppm: 3.25 (s, 3 H), 3.89 (s, 3H), 4.94 (s, 2 H), 6.28 (d, $J = 9.51$ Hz, 1H), 7.35 (dd, $J = 9.51$, 2.53 Hz, 1H), 7.56 (d, $J = 2.28$ Hz, 1H), 8.05 (s, 1H), 11.81 (br s, 1H). HRMS (ESI, $[M+H]^+$): calcd for $C_{15}H_{14}N_5O_3$: 312.1091, found: 312.1090.

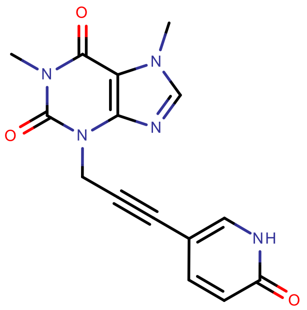

1,7-dimethyl-3-[3-(1H-pyrazol-4-yl)prop-2-yn-1-yl]-2,3,6,7-tetrahydro-1H-purine-2,6-dione (**8**). Step 1: tert-butyl 4-[3-(1,7-dimethyl-2,6-dioxo-2,3,6,7-tetrahydro-1H-purin-3-yl)prop-1-yn-1-yl]-1H-pyrazole-1-carboxylate (**S-8A**) was prepared analogously to **1**, from **1B** (300 mg, 1.38 mmol) and tert-butyl 4-iodo-1H-pyrazole-1-carboxylate (485 mg, 1.65 mmol, commercially available, CAS-RN: [121669-70-3]) at 60 °C for 3 h. Work up and purification: The reaction mixture was cooled to rt and filtered through a pad of Celite®. The filtrate was evaporated under reduced pressure and the residue purified by preparative HPLC (Aquity BEH, ACN, H_2O/FA) to yield the desired product

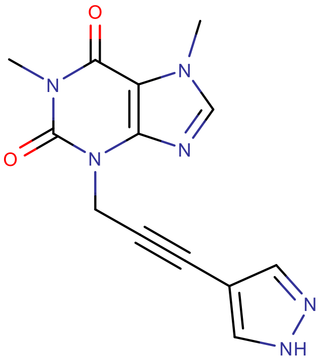

S-8A (100 mg, 19% yield), which was used in the next step. Step 2: To a stirred solution of **S-8A** (100 mg, 0.26 mmol) in DCM (10.0 mL) was added 4N HCl in dioxane (1.00 mL). The reaction mixture was allowed to slowly warm to rt and stirred for 2 h, before being concentrated under reduced pressure. The residue was triturated with n-pentane, then the HCl salt was neutralized with saturated aq. $NaHCO_3$ solution and extracted with EtOAc (3×). The combined organic layers were dried over Na_2SO_4, filtered and concentrated under reduced pressure to provide product **8** as a solid (45 mg, 61% yield). LC-MS (*method 4*): ^tR = 1.19 min; MS (ESI$^+$): *m/z* = 285 $[M+H]^+$. ^1H NMR (400 MHz, DMSO-d_6) δ ppm: 3.25 (s, 3 H), 3.89 (s, 3H), 4.94 (s, 2 H), 7.54–8.00 (m, 2H), 8.05 (s, 1H), 13.06 (br s, 1H). HRMS (ESI, $[M+H]^+$): calcd for $C_{13}H_{13}N_6O_2$: 285.1095, found: 285.1093.

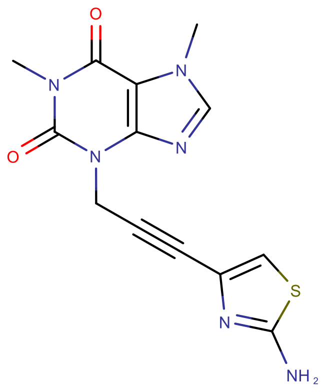

3-[3-(2-amino-1,3-thiazol-4-yl)prop-2-yn-1-yl]-1,7-dimethyl-2,3,6,7-tetrahydro-1H-purine-2,6-dione; trifluoroacetic acid (**9**). Step 1: To a degassed solution of **1B** (150 mg, 0.69 mmol) in DMF (4 mL) were added tert-butyl-*N*-(4-bromo-1,3-thiazol-2-yl)carbamate (192 mg, 0.69 mmol, commercially available, CAS-RN: [944804-88-0]), copper(I)iodide (26.0 mg, 0.14 mmol), triethylamine (0.29 mL, 2.07 mmol) and Pd(dppf)Cl_2·DCM (56.0 mg, 0.07 mmol). The reaction mixture was irradiated in the microwave at 100 °C for 1 h. Work up and purification: the reaction mixture was diluted with EtOAc, filtered through a pad of Celite®, and the filtrate was concentrated under reduced pressure. The residue was purified by flash column chromatography (CombiFlash, 40 g column, 30% EtOAc in petroleum ether) to provide tert-butyl N-{4-[3-(1,7-dimethyl-2,6-dioxo-2,3,6,7-tetrahydro-1H-purin-3-yl)prop-1-yn-1-yl]-1,3-thiazol-2-yl}carbamate (**S9-A**, 60 mg, 21%

yield). LC-MS (*method 4*): ^{t}R = 1.70 min; MS (ESI$^+$): *m/z* = 417 [M+H]$^+$. Step 2: To an ice-cold solution of tert-butyl-*N*-{4-[3-(1,7-dimethyl-2,6-dioxo-2,3,6,7-tetrahydro-1H-purin-3-yl)prop-1-yn-1-yl]-1,3-thiazol-2-yl}carbamate (60 mg, 0.14 mmol) in DCM (2 mL) was added TFA (0.50 mL) dropwise. The reaction mixture was stirred at rt for 3 h. The solvent was evaporated under reduced pressure. The residue was triturated with Et_2O to yield compound **9** (33 mg, 53% yield). LC-MS (*method 6*): ^{t}R = 1.80 min. MS (ESI$^+$): *m/z* = 317 [M+H]$^+$. ^{1}H NMR (400 MHz, DMSO-*d*$_6$) δ ppm: 3.25 (s, 3 H), 3.90 (s, 3H), 4.93 (s, 2 H), 6.85–7.25 (m, 3H), 8.05 (s, 1H). HRMS (ESI, [M+H]$^+$): calcd for $C_{13}H_{13}N_6O_2S$: 317.0815, found: 317.0814.

3-[3-(1H-indazol-6-yl)prop-2-yn-1-yl]-1,7-dimethyl-2,3,6,7-tetrahydro-1H-purine-2,6-dione

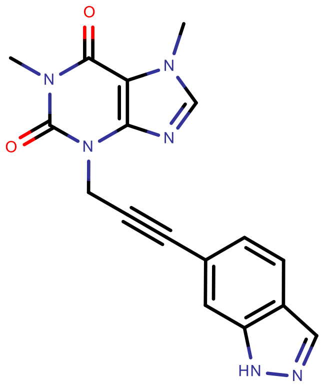

(**10**). Compound **10** was prepared analogously to **1**, from **1B** (250 mg, 1.00 mmol) and 6-bromo-1H-indazole (339 mg, 2.00 mmol, commercially available, CAS-RN: [79762-54-2]) at 70 °C for 3 h. Work up and purification was carried out analogously to **5** giving the desired product **10** (75 mg, 20% yield). LC-MS (*method 4*): ^{t}R = 1.41 min; MS (ESI$^+$): *m/z* = 335 [M+H]$^+$. ^{1}H NMR (400 MHz, DMSO-*d*$_6$) δ ppm: 3.27 (s, 3 H), 3.91 (s, 3H), 5.03 (s, 2 H), 7.07 (d, *J* = 8.24 Hz, 1H), 7.58 (s, 1H), 7.73 (d, *J* = 8.36 Hz, 1H), 8.07 (s, 1H), 8.09 (s, 1H), 13.27 (br s, 1H). HRMS (ESI, [M+H]$^+$): calcd for $C_{17}H_{15}N_6O_2$: 335.1251, found: 335.1253.

3-[3-(1H-indazol-4-yl)prop-2-yn-1-yl]-1,7-dimethyl-2,3,6,7-tetrahydro-1H-purine-2,6-dione

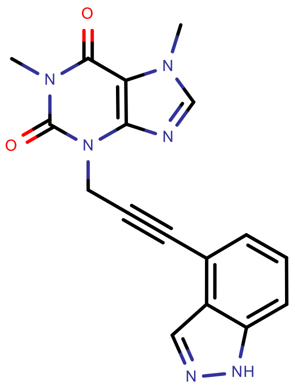

(**11**). Compound **11** was prepared analogously to **1**, from **1B** (300 mg, 1.00 mmol) and 4-bromo-1H-indazole (298 mg, 2.00 mmol, commercially available, CAS-RN: [186407-74-9]) at 90 °C for 80 min in the microwave. Work up and purification: the reaction mixture was diluted with HCl (1N) and extracted with EtOAc (2-3 times), washed with brine, dried over Na_2SO_4, filtered and concentrated. The residue was purified by preparative HPLC (Aquity BEH, ACN, H_2O/FA) giving compound **11** (33 mg, 7% yield). LC-MS (*method 4*): ^{t}R = 1.40 min; MS (ESI^+): *m/z* = 335 $[M+H]^+$. ^1H NMR (400 MHz, DMSO-d_6) δ ppm: 3.28 (s, 3 H), 3.91 (s, 3H), 5.10 (s, 2 H), 7.17 (d, *J* = 6.97 Hz, 1H), 7.31 (dd, *J* = 8.36, 7.10 Hz, 1H), 7.57 (d, *J* = 8.49 Hz, 1H), 7.97 (s, 1H), 8.10 (s, 1H). HRMS (ESI, $[M+H]^+$): calcd for $C_{17}H_{15}N_6O_2$: 335.1251, found: 335.1249.

2.4. Synthesis of Compounds 12-22

1-[3-(3-hydroxyphenyl)prop-2-yn-1-yl]-3-methyl-1,2,3,4-tetrahydropyrimidine-2,4-dione (**12**).

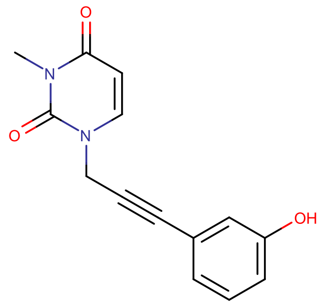

Step 1: To a degassed solution of 3-iodophenol (500 mg, 2.27 mmol, commercially available, CAS-RN: [626-02-8]) in ACN (5 mL) were added triethylamine (348 µL, 2.50 mmol), prop-2-yn-1-ol (393 µL, 6.82 mmol, commercially available, CAS-RN: [107-19-7]), copper(I) iodide (43.0 mg, 0.23 mmol) and tetrakis(triphenylphosphine)-palladium(0) (262 mg, 0.23 mmol). The reaction mixture was stirred at 65 °C for 2 h. The mixture was concentrated under reduced pressure. The residue was purified by flash column chromatography (25 g column, 50% EtOAc in cyclohexane). The desired fractions were concentrated to provide 3-(3-hydroxyprop-1-yn-1-yl)phenol (**S-12A**, 313 mg, 93% yield). LC-MS (*method 3*): $^{t}R = 0.70$ min; MS (ESI^{+}): $m/z = 149$ $[M+H]^{+}$. The product was used in the next step. Step 2: The reaction was conducted under Argon. A solution of propargyl alcohol **S-12A** (178 mg, 1.20 mmol) was dissolved in DCM (7 mL). Triphenylphosphine (410 mg, 1.56 mmol) was added and the solution was cooled with water/ice. Tetrabromomethane (518 mg, 1.56 mmol) was added and the reaction mixture was stirred in a thawing ice bath for 3 h. The mixture was concentrated under reduced pressure and the residue was purified by flash column chromatography (10 g column, 20% EtOAc in cyclohexane). The desired fractions were concentrated to provide 3-(3-bromoprop-1-yn-1-yl)phenol (**S-12B**, 81 mg, 32% yield). LC-MS (*method 3*): $^{t}R = 0.98$ min; MS (ESI^{+}): $m/z = 212$ $[M+H]^{+}$. The product was used in the next step. Step 3: To a stirred solution of propargyl bromide **S-12B** (81.0 mg, 0.38 mmol) in DMF (2 mL) were added 3-Methyluracil (48.0 mg, 0.38 mmol, commercially available, CAS-RN: [608-34-4]) and potassium carbonate (106 mg, 0.77 mmol). The reaction mixture was stirred at rt for 2 h. The mixture was diluted with DMF (2

mL), filtered through a syringe filter and purified by semi-preparative HPLC (Sunfire C18, ACN, H_2O/TFA) to provide product **12** (67 mg, 68% yield). LC-MS (*method 3*): ^tR = 0.82 min. MS (ESI$^+$): *m/z* = 257 [M+H]$^+$. ^1H NMR (400 MHz, DMSO-d_6) δ ppm: 3.18 (s, 3 H), 4.81 (s, 2 H), 5.78 (d, *J* = 7.86 Hz, 1 H), 6.78–6.82 (m, 2 H), 6.86 (dt, *J* = 7.67, 1.11 Hz, 1 H), 7.14–7.20 (m, 1 H), 7.83 (d, *J* = 7.98 Hz, 1 H), 9.66 (br s, 1 H). HRMS (ESI, [M+H]$^+$): calcd for $C_{14}H_{13}N_2O_3$: 257.0920, found: 257.0919.

1-{[5-(3-hydroxyphenyl)-1,3,4-thiadiazol-2-yl]methyl}-3-methyl-1,2,3,4-tetrahydropyrimidine-2,4-dione (**13**). Step 1: Methane sulfonate formation: To a stirred solution of (5-bromo-1,3,4-thiadiazol-2-yl)methanol (390 mg, 2.00 mmol, commercially available, CAS-RN: [1339055-00-3]) in DCM (10 mL) was added triethylamine (380 µL, 2.74 mmol) followed by dropwise addition of methane sulfonyl chloride (210 mL, 2.71 mmol). The reaction mixture was stirred at rt for 2 h. Upon completion, the mixture was diluted with DCM and H_2O. The organic layer was separated via a phase separation cartridge and concentrated under reduced pressure. Alkylation: The residue was dissolved in DMF (5 mL), and 3-methyluracil (230 mg, 1.82 mmol, commercially available, CAS-RN: [608-34-4]) and potassium carbonate (630 mg, 4.57 mmol) were added. The reaction mixture was stirred overnight at rt, filtered and concentrated. The residue was purified by preparative HPLC (Sunfire C18, ACN, H_2O/TFA) to provide 1-[(5-bromo-1,3,4-thiadiazol-2-yl)methyl]-3-methyl-1,2,3,4-tetrahydropyrimidine-2,4-dione (**S13-A**, 357 mg, 65% yield). LC-MS (*method 3*): ^tR = 0.72 min; MS (ESI$^+$): *m/z* = 304 [M+H]$^+$. The product was used in the next step. Step 2: A mixture of **S-13A** (30.0 mg, 0.10 mmol), (3-hydroxyphenyl)boronic acid (18.0 mg, 0.13 mmol, commercially

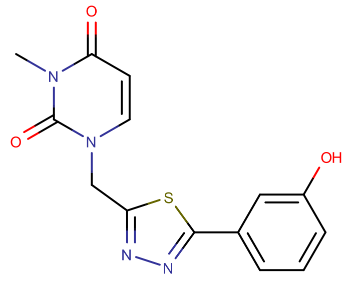

available, CAS-RN: [87199-18-6]), sodium carbonate (2 N aqueous solution, 130 µL, 0.26 mmol) and XPhos Pd G2 (5.00 mg, 0.01 mmol, commercially available, CAS-RN: [1310584-14-5]) in ethanol (1 mL) was stirred at 120 °C in the microwave for 10 min. The reaction mixture was diluted with DMF, filtered and purified by preparative HPLC (Sunfire C18, ACN, H_2O/TFA) to yield the desired product **13** (20 mg, 63% yield). LC-MS (*method 7*): ^tR = 0.52 min; MS (ESI^+): *m/z* = 317 $[M+H]^+$. ^1H NMR (400 MHz, DMSO-d_6) δ ppm: 3.18 (s, 3 H), 5.24 (s, 2 H), 5.82 (d, *J* = 7.86 Hz, 1 H), 6.93–6.98 (m, 1 H), 7.33–7.37 (m, 3H), 7.90 (d, *J* = 7.86 Hz, 1 H), 9.88 (br s, 1 H). HRMS (ESI, $[M+H]^+$): calcd for $C_{14}H_{13}N_4O_3S$: 317.0703, found: 317.0704.

1-{[2-(3-hydroxyphenyl)-1,3-thiazol-5-yl]methyl}-3-methyl-1,2,3,4-tetrahydropyrimidine-2,4-dione (**14**).

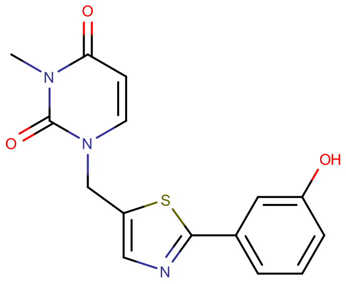

Step 1: A mixture of 3-methyluracil (2.00 g, 15.9 mmol, commercially available, CAS-RN: [608-34-4]), (2-bromo-1,3-thiazol-5-yl)methanol (4.00 g, 20.6 mmol, commercially available, CAS-RN: [687636-93-7]) and triphenylphosphine (6.40 g, 24.3 mmol) in THF (25 mL) was stirred at rt for 5 min, before di-tert-butyl azodicarboxylate (5.60 g, 24.3 mmol, commercially available, CAS-RN: [870-50-8]) dissolved in THF (25 mL) was added dropwise. The reaction mixture was stirred at rt overnight. The reaction mixture was concentrated under reduced pressue and the residue was purified by preparative HPLC (Sunfire C18, ACN, H_2O/TFA) to provide 1-[(2-bromo-1,3-thiazol-5-yl)methyl]-3-methyl-1,2,3,4-tetrahydropyrimidine-2,4-dione (**S-14A**, 2.80 g, 58% yield). LC-MS (*method 3*): ^tR = 0.81 min; MS (ESI^+): *m/z* = 302 $[M+H]^+$. The product was used in the next step. Step 2: To a stirred mixture of **S-14A** (71.0 mg, 0.24 mmol) and (3-hydroxyphenyl)boronic acid (40.0 mg, 0.29 mmol, commercially available, CAS-RN: [87199-18-6]) in dioxane (2 mL) was

added sodium carbonate (2 N aqueous solution, 300 µL, 0.60 mmol) and XPhos Pd G2 (10.0 mg, 0.01 mmol, commercially available, CAS-RN: [1310584-14-5]). The reaction mixture was stirred at 80 °C overnight. The reaction mixture was concentrated, the residue diluted with DMF, filtered and purified by preparative HPLC (XBridge C18, ACN, H_2O/NH_3) to provide the desired product **14** (30 mg, 74% yield). LC-MS (*method 8*): ^tR = 0.42 min; MS (ESI$^+$): *m/z* = 316 $[M+H]^+$. ^1H NMR (400 MHz, DMSO-d_6) δ ppm: 3.17 (s, 3 H), 5.17 (s, 2 H), 5.77 (d, *J* = 7.98 Hz, 1 H), 6.84–6.88 (m, 1 H), 7.25–7.33 (m, 3 H), 7.88 (d, *J* = 7.98 Hz, 1 H), 7.90 (s, 1 H), 9.73 (br s, 1 H). HRMS (ESI, $[M+H]^+$): calcd for $C_{15}H_{14}N_3O_3S$: 316.0750, found: 316.0752.

1-{[5-(3-hydroxyphenyl)-1,3-thiazol-2-yl]methyl}-3-methyl-1,2,3,4-tetrahydropyrimidine-2,4-dione (**15**).

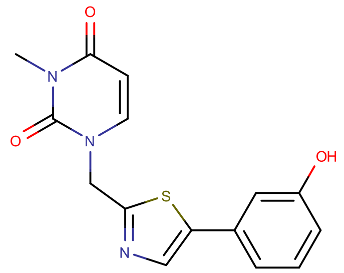

Compound **15** was prepared analogously to **14** in two steps. For step 1, 3-methyluracil (50.0 mg, 0.40 mmol, commercially available, CAS-RN: [608-34-4]) and (5-chloro-1,3-thiazol-2-yl)methanol (70.0 mg, 0.47 mmol, commercially available, CAS-RN: [50398-78-2]) were used to provide 1-{[5-(3-hydroxyphenyl)-1,3-thiazol-2-yl]methyl}-3-methyl-1,2,3,4-tetrahydro-pyrimidine-2,4-dione (**S-15A**, 65 mg, quantitative yield). LC-MS (*method 9*): ^tR = 0.77 min; MS (ESI$^+$): *m/z* = 258 $[M+H]^+$. The intermediate **S-15A** was used in step 2 with (3-hydroxyphenyl)boronic acid (45.0 mg, 0.33 mmol, commercially available, CAS-RN: [87199-18-6]) to yield the final compound **15** (43 mg, 54% yield). LC-MS (*method 7*): ^tR = 0.57 min; MS (ESI$^+$): *m/z* = 316 $[M+H]^+$. ^1H NMR (400 MHz, DMSO-d_6) δ ppm: 3.17 (s, 3 H), 5.26 (s, 2 H), 5.80 (d, *J* = 7.86 Hz, 1 H), 6.77 (ddd, *J* = 8.11, 2.34, 0.82, 1 H), 6.99 (t, *J* = 1.96 Hz, 1 H), 7.03–7.09 (m, 1 H), 7.19–7.27 (m., 1 H), 7.86 (d, *J* = 7.86

Hz, 1 H), 8.05 (s, 1 H), 9.63 (br s, 1 H). HRMS (ESI, [M+H]$^+$): calcd for $C_{15}H_{14}N_3O_3S$: 316.0750, found: 316.0750.

1-{[4-(3-hydroxyphenyl)-1,3-thiazol-2-yl]methyl}-3-methyl-1,2,3,4-tetrahydropyrimidine-2,4-dione (**16**). Compound **16** was prepared analogously to **14** in two steps. For step 1, 3-methyluracil (50.0 mg, 0.40 mmol, commercially available, CAS-RN: [608-34-4]) and (4-bromo-1,3-thiazol-2-yl)methanol (90.0 mg, 0.46 mmol, commercially available, CAS-RN: [204513-31-5]) were used to yield 1-[(4-bromo-1,3-thiazol-2-yl)methyl]-3-methyl-1,2,3,4-tetrahydropyrimidine-2,4-dione (**S-16A**, 102 mg, 85% yield). LC-MS (*method 3*): ^tR = 0.79 min; MS (ESI$^+$): *m/z* = 302 [M+H]$^+$. This intermediate **S-16A** was used in step 2 with (3-hydroxyphenyl)boronic acid (55.0 mg, 0.40 mmol, commercially available, CAS-RN: [87199-18-6]) to provide the final compound **16** (26 mg, 25% yield). LC-MS (*method 7*): ^tR = 0.59 min; MS (ESI$^+$): *m/z* = 316 [M+H]$^+$. ^1H NMR (400 MHz, DMSO-d_6) δ ppm: 3.18 (s, 3 H), 5.30 (s, 2 H), 5.83 (d, J = 7.86 Hz, 1 H), 6.74 (ddd, J = 7.98, 2.34, 1.08 Hz, 1 H), 7.18–7.25 (m, 1 H), 7.31–7.35 (m, 2 H), 7.91 (d, J = 7.98 Hz, 1 H), 7.99 (s, 1 H), 9.49 (s, 1 H). HRMS (ESI, [M+H]$^+$): calcd for $C_{15}H_{14}N_3O_3S$: 316.0750, found: 316.0751.

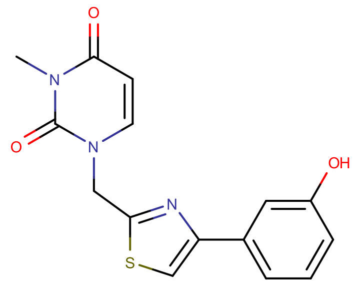

1-{[3-(3-hydroxyphenyl)-1,2-oxazol-5-yl]methyl}-3-methyl-1,2,3,4-tetrahydropyrimidine-2,4-dione (**17**).

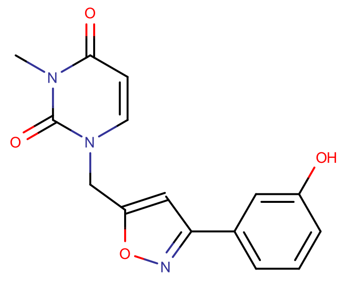

Step 1: To an ice-cold stirred solution of methyl 3-(3-hydroxyphenyl)-1,2-oxazole-5-carboxylate (200 mg, 0.91 mmol) in DMF (15 mL) was added sodium hydride (60% in mineral oil, 55.0 mg, 1.37 mmol) and the mixture was stirred at 0 °C for 20 min. Methyl iodide in MTBE (2M, 547 µL, 1.10 mmol) was added and the mixture was stirred at rt for 1 h. Water (2 mL) and DMF (3 mL) were added and the solution was purified by semi-preparative HPLC (Sunfire C18, ACN, H_2O/TFA) to give methyl 3-(3-methoxyphenyl)-1,2-oxazole-5-carboxylate (**S-17A**, 80 mg, 38% yield). LC-MS (*method 3*): ^tR = 1.03 min; MS (ESI^+): *m/z* = 234 $[M+H]^+$. The product was used in the next step. Step 2: To an ice-cold stirred solution of **S-17A** (80.0 mg, 0.34 mmol) in MeOH (0.75 mL) and THF (0.75 mL) was added sodium borohydride (55.0 mg, 1.44 mmol) and the reaction mixture was stirred at rt for 30 min. H_2O (2 mL) was added and aqueous layer was extracted with EtOAc (3 x 3 mL). The combined organic layers were dried over $MgSO_4$, filtered and concentrated to provide [3-(3-methoxyphenyl)-1,2-oxazol-5-yl]methanol (**S-17B**, 76 mg, quantitative yield). LC-MS (*method 3*): ^tR = 0.86 min; MS (ESI^+): *m/z* = 206 $[M+H]^+$. The crude product **S-17B** was used in the next step without further purification. Step 3: A mixture of 3-methyluracil (45.0 mg, 0.36 mmol, commercially available, CAS-RN: [608-34-4]), **S-17B** (73.0 mg, 0.36 mmol) and triphenylphosphine (560 mg, 2.13 mmol) in THF (2 mL) was stirred for 10 min, before di-tert-butyl azodicarboxylate (491 mg, 2.13 mmol, commercially available, CAS-RN: [870-50-8]) was added and the reaction mixture was stirred at 50 °C overnight. The reaction mixture was concentrated under reduced pressure and the residue was purified by semi-preparative HPLC (Sunfire C18, ACN, H_2O/TFA) to yield 1-{[3-(3-methoxyphenyl)-1,2-oxazol-5-yl]methyl}-3-

methyl-1,2,3,4-tetrahydropyrimidine-2,4-dione (**S-17C**, 92 mg, 83% yield). LC-MS (*method 3*): ^{t}R = 0.92 min; MS (ESI^{+}): *m/z* = 314 [M+H]$^{+}$. The product was used in the next step. Step 4: To an ice-cold stirred solution of **S-17C** (86.0 mg, 0.27 mmol) in DCM (2 mL) was added boron tribromide in DCM (1M, 823 µL, 0.82 mmol). The reaction mixture was stirred at rt for 1 h. An aqueous solution of Na_2CO_3 (2M, 0.5 mL) was added and the mixture was concentrated under reduced pressure. The residue was purified by preparative HPLC (Sunfire C18, ACN, H_2O/TFA) to provide **17** (21 mg, 26% yield). LC-MS (*method 3*): ^{t}R = 0.81 min; MS (ESI^{+}): *m/z* = 300 [M+H]$^{+}$. ^{1}H NMR (400 MHz, DMSO-d_6) δ ppm: 3.18 (s, 3 H), 5.08 (s, 2 H), 5.78 (d, *J* = 7.86 Hz, 1 H), 6.90 (ddd, *J* = 7.92, 2.41, 1.20 Hz, 1 H), 6.93 (s, 1 H), 7.18–7.21 (m, 1 H), 7.25–7.35 (m, 2 H), 7.81 (d, *J* = 7.86 Hz, 1 H), 9.79 (br s, 1 H). HRMS (ESI, [M+H]$^{+}$): calcd for $C_{15}H_{14}N_3O_4$: 300.0979, found: 300.0980.

1-{[5-(3-hydroxyphenyl)-1,2-oxazol-3-yl]methyl}-3-methyl-1,2,3,4-tetrahydropyrimidine-2,4-dione (**18**). Step 1: To a stirred solution of 3-ethynylphenol (0.50 g, 4.23 mmol, commercially available, CAS-RN: [10401-11-3]) in ACN (12 mL) were added DIPEA (1.46 mL, 8.47 mmol) and ethyl-2-chloro-2-(hydroxyimino)acetate (1.60 g, 10.6 mmol, commercially available, CAS-RN: [95080-93-6]) and the resulting mixture was stirred at 50 °C overnight. The reaction mixture was concentrated under reduced pressure and the residue was partitioned between aq. HCl (1M) and EtOAc. The aqueous layer was and extracted with EtOAc, the combined organic layers were dried over $MgSO_4$, filtered and concentrated under reduced pressure. The residue was dissolved in DMF/MeOH/H_2O, acidified with TFA and purified by preparative HPLC (Sunfire C18, ACN, H_2O/TFA) to yield ethyl 5-(3-hydroxyphenyl)-1,2-

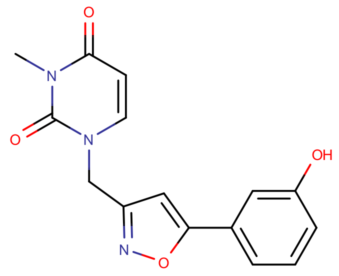

oxazole-3-carboxylate (**S-18A**,345 mg, 35% yield). LC-MS (*method 3*): ^{t}R = 0.93 min; MS (ESI^{+}): *m/z* = 234 [M+H]$^{+}$. The product was used in the next step. Step 2: To a stirred solution of **S-18A** (200 mg, 0.86 mmol) in DMF (5 mL) was added sodium hydride (60% in mineral oil, 36.0 mg, 0.89 mmol) and stirred at rt for 30 min. 1-(bromomethyl)-4-methoxybenzene (136 µL, 0.94 mmol, commercially available, CAS-RN: [2746-25-0]) was added and the mixture stirred at rt for 2 h. The reaction mixture was diluted with MeOH and H_2O, acidified with TFA and purified by preparative HPLC (Sunfire C18, ACN, H_2O/TFA) to give ethyl 5-{3-[(4-methoxyphenyl)methoxy]phenyl}-1,2-oxazole-3-carboxylate (**S-18B**, 126 mg, 42%). LC-MS (*method 3*): ^{t}R = 1.17 min; MS (ESI^{+}): *m/z* = 354 [M+H]$^{+}$. The product was used in the next step. Step 3: **S-18B** (120 mg, 0.34 mmol) was dissolved in THF (10 mL) and cooled to -20 °C. Lithium aluminum hydride (1M in THF, 340 µL, 0.34 mmol) was added dropwise at -20 °C and the resulting mixture was stirred at this temperature for 40 min, before the cooling bath was removed and stirring continued for additional 10 min. H_2O (15 µL) was added, followed by aq. NaOH solution (4M, 15 µL) and the resulting mixture was stirred at rt for 10 min. Next, H_2O (40 µL) was added and the resulting mixture was stirred at rt for 1 h. The mixture was filtered, diluted with H_2O, acidified with TFA and purified by preparative HPLC (Sunfire C18, ACN, H_2O/TFA) to provide (5-{3-[(4-methoxyphenyl)methoxy]phenyl}-1,2-oxazol-3-yl)methanol (**S-18C**, 83 mg, 78% yield). LC-MS (*method 3*): ^{t}R = 1.01 min; MS (ESI^{+}): *m/z* = 312 [M+H]$^{+}$. The product was used in the next step. Step 4: A mixture of **S-18C** (80.0 mg, 0.26 mmol), triphenylphosphine (67 mg, 0.26 mmol) and carbon tetrachloride (124 µL, 1.29 mmol) in ACN (2.00 mL) was flushed with N_2 and stirred at 80 °C for 4 h. The reaction mixture was concentrated under reduced pressure and the residue was purified by flash column chromatography (10 g column, 20% EtOAc in cyclohexanes), to provide 3-[3-(chloromethyl)-1,2-oxazol-5-yl]phenol (**S-18D**, 25 mg, 46%

yield). LC-MS (*method 3*): ${}^{t}R$ = 0.92 min; MS (ESI$^+$): *m/z* = 210 [M+H]$^+$. The product was used in the next step. Step 5: **S-18D** (25.0 mg, 0.12 mmol) was dissolved in DMF (2 mL). 3-Methyluracil (28.0 mg, purity 80%, 0.18 mmol, commercially available, CAS-RN: [608-34-4]) and potassium carbonate (41.0 mg, 0.30 mmol) were added and the resulting mixture was stirred at rt overnight. The reaction mixture was filtered, washed with MeOH, diluted with H_2O, acidified with TFA, and purified by preparative HPLC (Sunfire C18, ACN, H_2O/TFA) to yield the final compound **18** (23 mg, 63% yield). LC-MS (*method 3*): ${}^{t}R$ = 0.79 min; MS (ESI$^+$): *m/z* = 300 [M+H]$^+$. ^{1}H NMR (400 MHz, DMSO-*d*$_6$) δ ppm: 3.18 (s, 3 H), 5.08 (s, 2 H), 5.78 (d, *J* = 7.86 Hz, 1 H), 6.88–6.91 (m, 1 H), 6.93 (s, 1 H), 7.20 (t, *J* = 1.84 Hz, 1 H), 7.25–7.34 (m, 2 H), 7.81 (d, *J* = 7.86 Hz, 1 H), 9.80 (br s, 1 H). HRMS (ESI, [M+H]$^+$): calcd for $C_{15}H_{14}N_3O_4$: 300.0979, found: 300.0978.

1-{[5-(3-hydroxyphenyl)-1,3-oxazol-2-yl]methyl}-3-methyl-1,2,3,4-tetrahydropyrimidine-2,4-

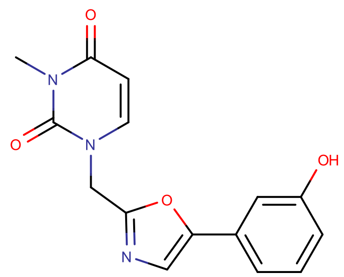

dione (**19**). Step 1: 1-[(5-bromo-1,3-oxazol-2-yl)methyl]-3-methyl-1,2,3,4-tetrahydropyrimidine-2,4-dione (**S-19A**, 75 mg, 55% yield) was prepared analogously to **S-14A**, from 3-methyluracil (60.0 mg, 0.48 mmol, commercially available, CAS-RN: [608-34-4]) and (5-bromo-1,3-oxazol-2-yl)methanol (110 mg, 0.62 mmol, commercially available, CAS-RN: [1454907-14-2]). LC-MS (*method 3*): ${}^{t}R$ = 0.71 min; MS (ESI$^+$): *m/z* = 288 [M+H]$^+$. The product was used in the next step. Step 2: **S-19A** (35.0 mg, 0.12 mmol), (3-hydroxyphenyl)boronic acid (20.0 mg, 0.15 mmol, commercially available, CAS-RN: [87199-18-6]) and sodium carbonate (39.0 mg, 0.37 mmol) were dissolved in dioxane (2 mL) and H_2O (0.5 mL), and degassed with N_2. Pd(dppf)Cl_2 (14.0 mg, 0.01 mmol, commercially available,

CAS-RN: [72287-26-4]) was added and the reaction mixture was stirred at 100 °C for 2 h. The reaction mixture was diluted with DMF and MeOH, filtered, acidified with TFA, and purified by preparative HPLC (Sunfire C18, ACN, H_2O/TFA) to provide **19** (22 mg, 60% yield). LC-MS (*method 3*): ^{t}R = 0.75 min; MS (ESI$^+$): *m/z* = 300 $[M+H]^+$. ^1H NMR (400 MHz, DMSO-d_6) δ ppm: 3.17 (s, 3 H), 5.17 (s, 2 H), 5.82 (d, *J* = 7.86 Hz, 1 H), 6.74–6.80 (m, 1 H), 7.04 (t, *J* = 1.90 Hz, 1 H), 7.11 (d, *J* = 7.86 Hz, 1 H), 7.26 (t, *J* = 7.92 Hz, 1 H), 7.55 (s, 1 H), 7.85 (d, *J* = 7.86 Hz, 1 H), 9.67 (br s, 1 H). HRMS (ESI, $[M+H]^+$): calcd for $C_{15}H_{14}N_3O_4$: 300.0979, found: 300.0977.

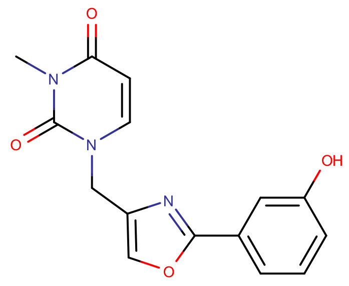

1-{[2-(3-hydroxyphenyl)-1,3-oxazol-4-yl]methyl}-3-methyl-1,2,3,4-tetrahydropyrimidine-2,4-dione (**20**). Step 1: Ethyl 2-(3-hydroxyphenyl)-1,3-oxazole-4-carboxylate (**S-20A**) was prepared analogously to **19** (Step 2), from ethyl 2-bromo-1,3-oxazole-4-carboxylate (300 mg, 1.36 mmol, commercially available, CAS-RN: [460081-20-3]) and (3-hydroxyphenyl)boronic acid (226 mg, 1.64 mmol, commercially available, CAS-RN: [87199-18-6]). Work up and purification: The reaction mixture was partitioned between EtOAc and aq. $NaHCO_3$. The organic layer was washed with water (2 x 50 mL), dried over Na_2SO_4, filtered and concentrated under reduced pressure. The residue was dissolved in MeOH/DMF/H_2O, acidified with TFA, and purified by preparative HPLC (Sunfire C18, ACN, H_2O/TFA) providing **S-20A** (80 mg, 25% yield). The product was used in the next step. Step 2: **S-20A** (40.0 mg, 0.17 mmol) was dissolved in THF (5 ml) and degassed with N_2. The mixture was cooled to -20°C and lithium aluminum hydride (1M in THF, 172 µL, 0.17 mmol) was added dropwise. The resulting mixture was stirred at rt for 30 min. H_2O (7 µL) was added, followed by aq. NaOH (4M, 8 µL) and the resulting mixture was stirred at rt for 30 min. Next, H_2O (20 µL)

was added, the mixture was filtered and concentrated under reduced pressure to provide 3-[4-(hydroxymethyl)-1,3-oxazol-2-yl]phenol (**S-20B**, 19 mg, 59% yield). The crude product was used in the next step without further purification. Step 3: **20** (4.4 mg, 15% yield) was prepared analogously to **14** (Step 1), from **S-20B** (19.0 mg, 0.10 mmol) and 3-methyluracil (13.0 mg, 0.10 mmol, commercially available, CAS-RN: [608-34-4]). The crude product was purified by preparative HPLC (XBridge C18, ACN, H_2O/NH_3) to give **20**. LC-MS (*method 9*): ^tR = 0.69 min; MS (ESI^+): *m/z* = 300 $[M+H]^+$. ^1H NMR (400 MHz, DMSO-d_6) δ ppm: 3.16 (s, 3 H), 4.91 (s, 2 H), 5.76 (d, *J* = 7.98 Hz, 1 H), 6.90 (ddd, *J* = 7.89, 2.44, 1.20 Hz, 1 H), 7.29–7.38 (m, 3 H), 7.81 (d, *J* = 7.98 Hz, 1 H), 8.14 (s, 1 H), 9.85 (br s, 1 H).

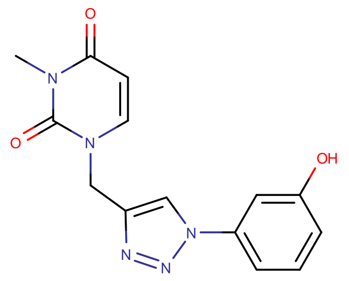

1-{[1-(3-hydroxyphenyl)-1H-1,2,3-triazol-4-yl]methyl}-3-methyl-1,2,3,4-tetrahydropyrimidine-2,4-dione (**21**). Step 1a: 3-methyluracil (300 mg, 2.38 mmol, commercially available, CAS-RN: [608-34-4]) was dissolved in DMF (10 mL). Next, potassium carbonate (822 mg, 5.95 mmol) was added followed by 3-bromoprop-1-yne (354 mg, 2.38 mmol, commercially available, CAS-RN: [106-96-7]). The resulting suspension was stirred at rt overnight. The reaction mixture was partitioned between DCM and water, the layers were separated, and the aqueous layer was further extracted with DCM. The combined organic layers were dried over Na_2SO_4, filtered and concentrated under reduced pressure. The residue was purified by flash column chromatography (40 g column, 0-60% EtOAc in cyclohexane) to provide 3-methyl-1-(prop-2-yn-1-yl)-1,2,3,4-tetrahydropyrimidine-2,4-dione (**S-21A**, 290 mg, 74% yield). LC-MS (*method 3*): ^tR = 0.24 min; MS (ESI^+): *m/z* = 165 $[M+H]^+$. Step 1b: 3-aminophenol (50.0 mg, 0.46 mmol, commercially available, CAS-RN: [591-27-5]) was

dissolved in aq. HCl (2N, 2 mL) and cooled in an ice bath. Sodium nitrite (32.0 mg, 0.46 mmol) in H_2O (1 mL) was added dropwise, turning the colorless solution to orange. The solution was stirred in the ice bath for 30 min. Next, a solution of sodium azide (30.0 mg, 0.46 mmol) in H_2O (1 mL) was added dropwise. The reaction mixture was stirred in the ice bath and warmed to rt overnight. The reaction mixture was diluted with DCM, the aqueous layer separated and extracted with DCM. The combined organic layers were dried over Na_2SO_4, filtered, and concentrated under reduced pressure to provide 3-azidophenol (**S-21B**). The crude material was used in the next step without further purification, assuming 100% conversion (62 mg). Step 2: 3-azidophenol (**S-21B**, 60.0 mg, 0.44 mmol) and **S-21A** (38.0 mg, 0.23 mmol) were dissolved in a mixture of MeOH (1.5 mL) and H_2O (1.5 mL). Sodium ascorbate (18.0 mg, 0.09 mmol) was added followed by copper(II) sulfate pentahydrate (17.0 mg, 0.07 mmol) and the brown suspension was stirred at rt. After 30 min, the reaction became a thick suspension. To improve solubility, DMSO (1 mL) was added and stirring at rt was continued for 6 h. The mixture was diluted with DMF, filtered, and purified by preparative HPLC (Sunfire C18, ACN, H_2O/TFA) to provide **21** (54 mg, 78% yield). LC-MS (*method 2*): ^{t}R = 0.38 min; MS (ESI^+): *m/z* = 300 $[M+H]^+$. ^1H NMR (400 MHz, DMSO-d_6) δ ppm: 3.16 (s, 3 H), 5.09 (s, 2 H), 5.76 (d, *J* = 7.86 Hz, 1 H), 6.85–6.89 (m, 1 H), 7.25–7.30 (m, 2 H), 7.33–7.39 (m, 1 H), 7.85 (d, *J* = 7.86 Hz, 1 H), 8.70 (s, 1 H), 10.01 (br s, 1 H). HRMS (ESI, $[M+H]^+$): calcd for $C_{14}H_{14}N_5O_3$: 300.1091, found: 300.1088.

1-{[2-(3-hydroxyphenyl)pyrimidin-5-yl]methyl}-3-methyl-1,2,3,4-tetrahydropyrimidine-2,4-

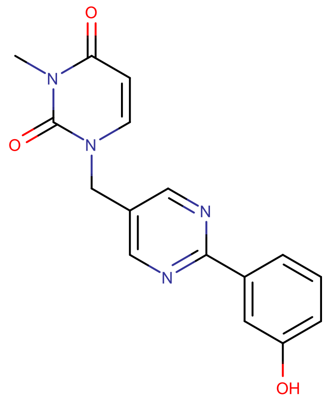

dione (**22**) Step 1: 1-[(2-chloropyrimidin-5-yl)methyl]-3-methyl-1,2,3,4-tetrahydropyrimidine-2,4-dione (**S-22A**, 88 mg, 73% yield) was prepared analogously to **S-14A**, from 3-methyluracil (60.0 mg, 0.48 mmol, commercially available, CAS-RN: [608-34-4]) and (2-chloropyrimidin-5-yl)methanol (89.0 mg, 0.62 mmol, commercially available, CAS-RN: [1046816-75-4]). LC-MS (*method 3*): $^{t}R = 0.67$ min; MS (ESI^{+}): $m/z = 253$ [M+H]$^{+}$. The product was used in the next step. Step 2: Compound **22** (34 mg, 69% yield) was prepared analogously to **19** from 1-[(2-chloropyrimidin-5-yl)methyl]-3-methyl-1,2,3,4-tetrahydropyrimidine-2,4-dione (40.0 mg, 0.16 mmol) and (3-hydroxyphenyl)boronic acid (26.0 mg, 0.19 mmol, commercially available, CAS-RN: [87199-18-6]). LC-MS (*method 7*): $^{t}R = 0.52$ min; MS (ESI^{+}): $m/z = 311$ [M+H]$^{+}$. ^{1}H NMR (400 MHz, DMSO-d_6) δ ppm: 3.15 (s, 3 H), 4.99 (s, 2 H), 5.77 (d, $J = 7.86$ Hz, 1 H), 6.91 (ddd, $J = 8.05, 2.34, 0.89$ Hz, 1 H), 7.31 (t, $J = 8.11$ Hz, 1 H), 7.79–7.84 (m, 2 H), 7.93 (d, $J = 7.86$ Hz, 1 H), 8.88 (s, 2 H), 9.62 (br s, 1 H). HRMS (ESI, [M+H]$^{+}$): calcd for $C_{16}H_{15}N_4O_3$: 311.1139, found: 311.1138.

2.5. Synthesis of Compounds 23-38

1-{[2-(2-fluoro-3-hydroxyphenyl)-1,3-thiazol-5-yl]methyl}-3-methyl-1,2,3,4-tetrahydro-

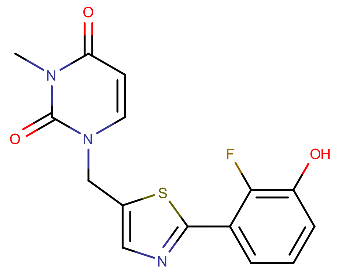

pyrimidine-2,4-dione (**23**). To a mixture of 3-bromo-2-fluorophenol (38.0 mg, 0.20 mmol, commercially available, CAS-RN: [156682-53-0]), bis(pinacolato)diboron (60.0 mg, 0.24 mmol) and potassium acetate (60.0 mg, 0.61 mmol) in ethanol (2 mL) were added XPhos Pd G2 (8.00 mg, 0.01 mmol) and XPhos (10.0 mg, 0.02 mmol). The resulting mixture was stirred at 120 °C for 10 min in the microwave. Then **S-14a** (30.0 mg, 0.10 mmol), aq. sodium carbonate (2 M, 320 µL, 0.64 mmol) and XPhos Pd G2 (8.00 mg, 0.01 mmol) were added and the reaction mixture was stirred at 120 °C for 10 min in the microwave. The mixture was diluted with DMF, filtered, and then purified by preparative HPLC (Sunfire C18, ACN, H_2O/TFA) to give **23** (16 mg, 47% yield). LC-MS (*method 7*): ^tR = 0.56 min; MS (ESI^+): *m/z* = 334 $[M+H]^+$. ^1H NMR (400 MHz, DMSO-d_6) δ ppm: 3.17 (s, 3 H), 5.21 (s, 2 H), 5.77 (d, *J* = 7.98 Hz, 1 H), 7.03–7.15 (m, 2 H), 7.56 (ddd, *J* = 7.79, 6.15, 1.90 Hz, 1 H), 7.91 (d, *J* = 7.86 Hz, 1 H), 8.00 (d, *J* = 2.41 Hz, 1 H), 10.20 (s, 1 H).

1-{[2-(2-chloro-3-hydroxyphenyl)-1,3-thiazol-5-yl]methyl}-3-methyl-1,2,3,4-tetrahydro-

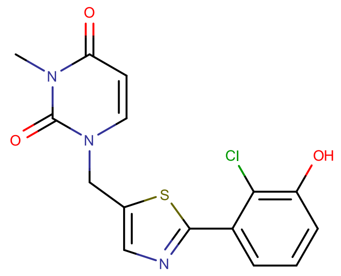

pyrimidine-2,4-dione (**24**). To a mixture of 3-bromo-2-chlorophenol (40.0 mg, 0.19 mmol, commercially available, CAS-RN: [66024-94-0]), bis(pinacolato)diboron (55.0 mg, 0.22 mmol) and potassium acetate (50.0 mg, 0.51 mmol) in dioxane (1 mL) was added Pd(dppf)Cl_2 · DCM (8.00 mg, 0.01 mmol). The mixture was stirred at 120 °C for 10 min in the microwave. Then **S-14A** (40.0 mg, 0.13 mmol), Pd(dppf)Cl_2 · DCM

(8.00 mg, 0.01 mmol) and aq. sodium carbonate (2 M, 260 µL, 0.52 mmol) were added and the reaction mixture was stirred at 140 °C for 10 min in the microwave. The mixture was diluted with DMF, filtered, and then purified by preparative HPLC (Sunfire C18, ACN, H_2O/TFA) to provide **24** (12 mg, 25% yield). LC-MS (*method 7*): ^tR = 0.59 min; MS (ESI$^+$): *m/z* = 350 $[M+H]^+$. ^1H NMR (400 MHz, DMSO-d_6) δ ppm: 3.17 (s, 3 H), 5.21 (s, 2 H), 5.78 (d, *J* = 7.86 Hz, 1 H), 7.07–7.11 (m, 1 H), 7.26 (t, *J* = 7.98 Hz, 1 H), 7.55 (dd, *J* = 7.79, 1.20 Hz, 1 H), 7.91 (d, *J* = 7.86 Hz, 1 H), 8.00 (s, 1 H), 10.51 (br s, 1 H).

1-{[2-(3-hydroxy-2-methylphenyl)-1,3-thiazol-5-yl]methyl}-3-methyl-1,2,3,4-tetrahydro-

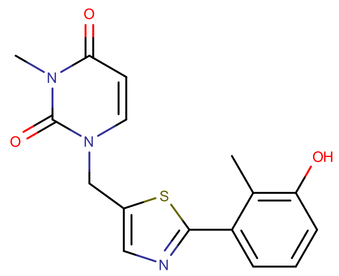

pyrimidine-2,4-dione (**25**). Compound **25** (13 mg, 38% yield) was prepared analogously to **23**, from 3-bromo-2-methylphenol (40.0 mg, 0.21 mmol, commercially available, CAS-RN: [7766-23-6]) and **S-14A** (30.0 mg, 0.10 mmol). LC-MS (*method 7*): ^tR = 0.57 min; MS (ESI$^+$): *m/z* = 330 $[M+H]^+$. ^1H NMR (400 MHz, DMSO-d_6) δ ppm: 2.29 (s, 3 H), 3.17 (s, 3 H), 5.19 (s, 2 H), 5.77 (d, *J* = 7.98 Hz, 1 H), 6.92 (d, *J* = 7.60 Hz, 1 H), 7.03–7.11 (m, 2 H), 7.90 (d, *J* = 7.86 Hz, 1 H), 7.93 (s, 1 H), 9.64 (br s, 1 H). HRMS (ESI, $[M+H]^+$): calcd for $C_{16}H_{16}N_3O_3S$: 330.0907, found: 330.0905.

1-{[2-(4-fluoro-3-hydroxyphenyl)-1,3-thiazol-5-yl]methyl}-3-methyl-1,2,3,4-tetrahydro-

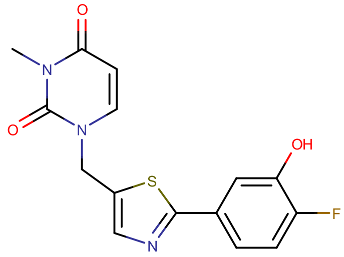

pyrimidine-2,4-dione (**26**). Compound **26** (15 mg, 45% yield) was prepared analogously to **23**, from 5-bromo-2-fluorophenol (40.0 mg, 0.21 mmol, commercially available, CAS-RN: [12204-58-7]) and **S-14A** (30.0 mg, 0.10 mmol). LC-MS (*method 7*): ^tR = 0.59 min; MS (ESI$^+$): *m/z* = 334 $[M+H]^+$. ^1H NMR (400 MHz, DMSO-

d_6) δ ppm: 3.17 (s, 3 H), 5.16 (s, 2 H), 5.77 (d, *J* = 7.98 Hz, 1 H), 7.20–7.27 (m, 1 H), 7.27–7.34 (m, 1 H), 7.51 (dd, *J* = 8.49, 2.15 Hz, 1 H), 7.86–7.91 (m, 2 H), 10.24 (br s, 1 H).

1-{[2-(4-chloro-3-hydroxyphenyl)-1,3-thiazol-5-yl]methyl}-3-methyl-1,2,3,4-tetrahydro-pyrimidine-2,4-dione (**27**). Compound **27** (16 mg, 35% yield) was prepared analogously to **24**, from 5-bromo-2-chlorophenol (40.0 mg, 0.19 mmol, commercially available, CAS-RN: [183802-98-4]) and **S14-A** (40.0 mg, 0.13 mmol). LC-MS (*method 7*): ^tR = 0.68 min; MS (ESI$^+$): *m/z* = 350 $[M+H]^+$. ^{1}H NMR (400 MHz, DMSO-d_6) δ ppm: 3.17 (s, 3 H), 5.17 (s, 2 H), 5.77 (d, *J* = 7.98 Hz, 1 H), 7.32 (dd, *J* = 8.24, 1.77 Hz, 1 H), 7.43 (d, *J* = 8.24 Hz, 1 H), 7.53 (d, *J* = 1.65 Hz, 1 H), 7.88 (d, *J* = 7.86 Hz, 1 H), 7.92 (s, 1 H), 10.55 (s, 1 H). HRMS (ESI, $[M+H]^+$): calcd for $C_{15}H_{13}ClN_3O_3S$: 350.0361, found: 350.0361.

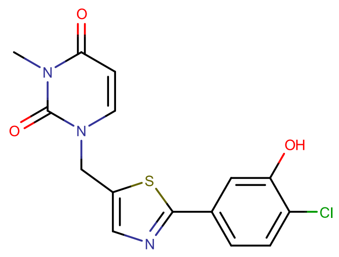

1-({2-[3-hydroxy-4-(trifluoromethyl)phenyl]-1,3-thiazol-5-yl}methyl)-3-methyl-1,2,3,4-tetra-hydropyrimidine-2,4-dione (**28**). Compound **28** (6 mg, 13% yield) was prepared analogously to **24**, from 5-bromo-2-(trifluoromethyl)phenol (50.0 mg, 0.21 mmol, commercially available, CAS-RN: [1121585-15-6]) and **S-14A** (40.0 mg, 0.13 mmol). The product was purified by preparative HPLC (XBridge C18, ACN, H_2O/NH_3) . LC-MS (*method 8*): ^tR = 0.39 min; MS (ESI$^+$): *m/z* = 384 $[M+H]^+$. ^{1}H NMR (400 MHz, DMSO-d_6) δ ppm: 3.17 (s, 3 H), 5.20 (s, 2 H), 5.78 (d, *J* = 7.86 Hz, 1 H), 7.44 (d, *J* = 8.36 Hz, 1 H), 7.56–7.64 (m, 2 H), 7.90 (d, *J* = 7.98 Hz, 1 H), 7.99 (s, 1 H), 10.90 (br s, 1 H). HRMS (ESI, $[M+H]^+$): calcd for $C_{16}H_{13}F_3N_3O_3S$: 384.0624, found: 384.0620.

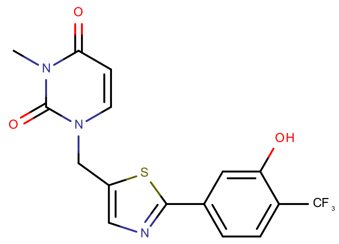

2-hydroxy-4-{5-[(3-methyl-2,4-dioxo-1,2,3,4-tetrahydropyrimidin-1-yl)methyl]-1,3-thiazol-2-yl}

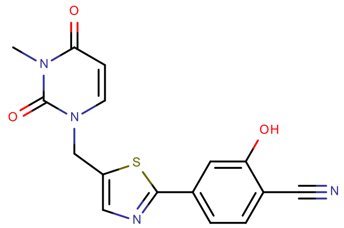

benzonitrile (**29**). Compound **29** (16 mg, 46% yield) was prepared analogously to **14**, from **S-14A** (30.0 mg, 0.10 mmol) and 2-hydroxy-4-(4,4,5,5-tetramethyl-1,3,2-dioxaborolan-2-yl)benzonitrile (37.0 mg, 0.15 mmol, commercially available, CAS-RN: [1350933-21-9]). LC-MS (*method 7*): ^{t}R = 0.60 min; MS (ESI^{+}): *m/z* = 341 [M+H]$^{+}$. ^{1}H NMR (400 MHz, DMSO-*d*$_6$) δ ppm: 3.17 (s, 3 H), 5.20 (s, 2 H), 5.77 (d, *J* = 7.86 Hz, 1 H), 7.44 (dd, *J* = 8.11, 1.52 Hz, 1 H), 7.57 (d, *J* = 1.52 Hz, 1 H), 7.71 (d, *J* = 8.11 Hz, 1 H), 7.89 (d, *J* = 7.86 Hz, 1 H), 8.00 (s, 1 H), 11.42 (s, 1 H). HRMS (ESI, [M+H]$^{+}$): calcd for $C_{16}H_{13}N_4O_3S$: 341.0703, found: 341.0700.

1-{[2-(3-fluoro-5-hydroxyphenyl)-1,3-thiazol-5-yl]methyl}-3-methyl-1,2,3,4-tetrahydro-

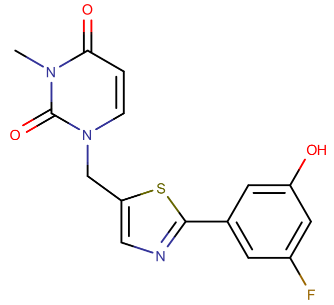

pyrimidine-2,4-dione (**30**). Compound **30** (9.7 mg, 44% yield) was prepared analogously to **14**, from **S-14A** (20.0 mg, 0.07 mmol) and (3-fluoro-5-hydroxyphenyl)boronic acid (16.0 mg, 0.10 mmol, commercially available, CAS-RN: [871329-82-7]). LC-MS (*method 7*): ^{t}R = 0.62 min; MS (ESI^{+}): *m/z* = 334 [M+H]$^{+}$. ^{1}H NMR (400 MHz, DMSO-*d*$_6$) δ ppm: 3.17 (s, 3 H), 5.18 (s, 2 H), 5.77 (d, *J* = 7.86 Hz, 1 H), 6.68 (dt, *J* = 10.68, 2.27 Hz, 1 H), 7.10–7.14 (m, 1 H), 7.15–7.17 (m, 1 H), 7.88 (d, *J* = 7.98 Hz, 1 H), 7.93 (s, 1 H), 10.26 (s, 1 H). HRMS (ESI, [M+H]$^{+}$): calcd for $C_{15}H_{13}FN_3O_3S$: 334.0656, found: 334.0656.

1-({2-[3-hydroxy-5-(trifluoromethyl)phenyl]-1,3-thiazol-5-yl}methyl)-3-methyl-1,2,3,4-tetra-

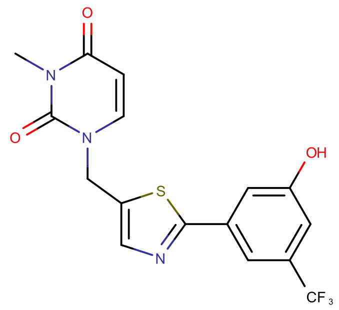

hydropyrimidine-2,4-dione (**31**). Compound **31** (27 mg, 43% yield) was prepared analogously to **23**, from 3-bromo-5-(trifluoromethyl)phenol (84.0 mg, 0.35 mmol, commercially available, CAS-RN: [1025718-84-6]) and **S-14A** (50.0 mg, 0.17 mmol). LC-MS (*method 3*): ^{t}R = 0.69 min; MS (ESI^{+}): *m/z* = 384 [M+H]$^{+}$. ^{1}H NMR (400 MHz, DMSO-d_6) δ ppm: 3.17 (s, 3 H), 5.20 (s, 2 H), 5.78 (d, *J* = 7.98 Hz, 1 H), 7.14 (s, 1 H), 7.54–7.60 (m, 2 H), 7.89 (d, *J* = 7.98 Hz, 1 H), 7.98 (s, 1 H), 10.51 (br s, 1 H). HRMS (ESI, [M+H]$^{+}$): calcd for $C_{16}H_{13}F_3N_3O_3S$, 384.0624; found, 384.0619.

1-{[2-(5-hydroxypyridin-3-yl)-1,3-thiazol-5-yl]methyl}-3-methyl-1,2,3,4-tetrahydropyrimidine-

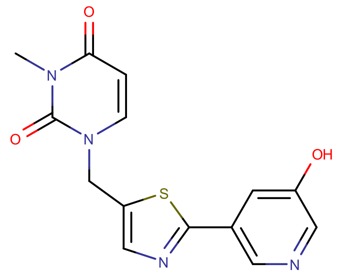

2,4-dione (**32**). Compound **32** (29 mg, 93% yield) was prepared analogously to **14**, from **S14-A** (30.0 mg, 0.10 mmol) and 5-(4,4,5,5-tetramethyl-1,3,2-dioxaborolan-2-yl)pyridin-3-ol (30.0 mg, 0.14 mmol, commercially available, CAS-RN: [1171891-35-2]). LC-MS (*method 7*): ^{t}R = 0.34 min; MS (ESI^{+}): *m/z* = 317 [M+H]$^{+}$. ^{1}H NMR (400 MHz, DMSO-d_6) δ ppm: 3.17 (s, 3 H), 5.20 (s, 2 H), 5.78 (d, *J* = 7.86 Hz, 1 H), 7.71 (t, *J* = 2.09 Hz, 1 H), 7.90 (d, *J* = 7.86 Hz, 1 H), 8.00 (s, 1 H), 8.25 (d, *J* = 2.53 Hz, 1 H), 8.58–8.61 (m, 1 H), 10.55 (br s, 1 H). HRMS (ESI, [M+H]$^{+}$): calcd for $C_{14}H_{13}N_4O_3S$: 317.0703, found: 317.0703.

1-{[2-(2-fluoro-5-hydroxyphenyl)-1,3-thiazol-5-yl]methyl}-3-methyl-1,2,3,4-tetrahydro-

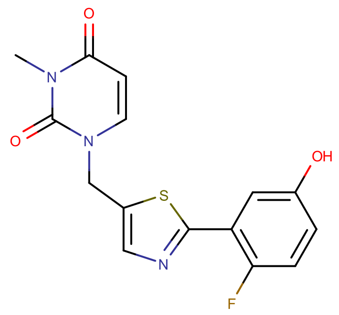

pyrimidine-2,4-dione (**33**). Compound **33** (11 mg, 34% yield) was prepared analogously to **23**, from 3-bromo-4-fluorophenol (40.0 mg, 0.21 mmol, commercially available, CAS-RN: [27407-11-0]) and **S-14A** (30.0 mg, 0.10 mmol). LC-MS (*method 7*): ^{t}R = 0.61 min; MS (ESI$^+$): *m/z* = 334 [M+H]$^+$. ^{1}H NMR (400 MHz, DMSO-d_6) δ ppm: 3.17 (s, 3 H), 5.20 (s, 2 H), 5.77 (d, *J* = 7.98 Hz, 1 H), 6.87 (dt, *J* = 8.78, 3.66 Hz, 1 H), 7.21 (dd, *J* = 10.96, 9.06 Hz, 1 H), 7.56 (dd, *J* = 6.02, 3.10 Hz, 1 H), 7.90 (d, *J* = 7.86 Hz, 1 H), 8.00 (d, *J* = 2.15 Hz, 1 H), 9.71 (s, 1 H). HRMS (ESI, [M+H]$^+$): calcd for $C_{15}H_{13}FN_3O_3S$: 334.0656, found: 334.0656.

1-{[2-(2,4-difluoro-3-hydroxyphenyl)-1,3-thiazol-5-yl]methyl}-3-methyl-1,2,3,4-tetrahydro-

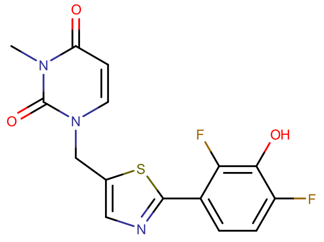

pyrimidine-2,4-dione (**34**). Compound **34** (90 mg, 77% yield) was prepared analogously to **14**, from **S-14A** (100 mg, 0.33 mmol) and 2,6-difluoro-3-(4,4,5,5-tetramethyl-1,3,2-dioxaborolan-2-yl)phenol (100 mg, 0.39 mmol, commercially available) in ethanol (5 mL) for 4 h at 80 °C. LC-MS (*method 3*): ^{t}R = 0.85 min; MS (ESI$^+$): *m/z* = 352 [M+H]$^+$. ^{1}H NMR (400 MHz, DMSO-d6) δ ppm: 3.17 (s, 3 H), 5.20 (s, 2 H), 5.77 (d, *J* = 7.98 Hz, 1 H), 7.18 (td, *J* = 9.57, 1.90 Hz, 1 H), 7.60 (ddd, *J* = 8.93, 7.92, 5.96 Hz, 1 H), 7.90 (d, *J* = 7.86 Hz, 1 H), 8.00 (d, *J* = 2.28 Hz, 1 H), 10.51–10.71 (m, 1 H). HRMS (ESI, [M+H]$^+$): calcd for $C_{15}H_{12}F_2N_3O_3S$: 352.0562, found: 352.0562.

1-{[2-(2-chloro-4-fluoro-3-hydroxyphenyl)-1,3-thiazol-5-yl]methyl}-3-methyl-1,2,3,4-tetra-

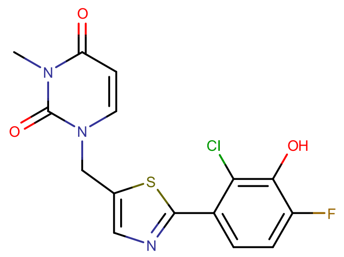

hydropyrimidine-2,4-dione (**35**). Step 1: 1-{[2-(2-chloro-4-fluoro-3-hydroxyphenyl)-1,3-thiazol-5-yl]methyl}-3-methyl-1,2,3,4-tetra-hydropyrimidine-2,4-dione (**S-35A**, 30 mg, 30% yield) was prepared analogously to **14**, from **S-14A** (80.0 mg, 0.27 mmol) and (2-chloro-4-fluoro-3-methoxyphenyl)boronic acid (81.0 mg, 0.40 mmol, commercially available, CAS-RN: [943831-11-6). The reaction mixture was stirred at 80 °C for 6 h, then filtered and purified by preparative HPLC (Sunfire C18, ACN, H_2O/TFA). LC-MS (*method 2*): ^tR = 0.69 min; MS (ESI$^+$): *m/z* = 382 $[M+H]^+$. The product was used in the next step. Step 2: To a solution of **S-35A** (20.0 mg, 0.05 mmol) in dichloroethane (2 mL) was added boron tribromide (1M in DCM, 0.16 mL, 0.16 mmol) and the resulting mixture was stirred at 50 °C for 24 h. Since the conversion of **S-35A** was incomplete, the reaction was cooled to rt, before additional boron tribromide (1M in DCM, 80.0 µL, 0.08 mmol) was added. The resulting mixture was again stirred at 50 °C for 72 h. After this time, reaction monitoring still indicated incomplete conversion, thus, the reaction was cooled to rt and additional boron tribromide (1M in DCM, 40.0 µL, 0.04 mmol) was added. The resulting mixture was again stirred at 50 °C. After 6 h, decomposition started to occur, therefore, the reaction mixture was concentrated, suspended in DMF, filtered and purified by preparative HPLC (C18, ACN, H_2O/TFA, 60 °C) giving **35** (7 mg, 36% yield). LC-MS (*method 2*): ^tR = 0.52 min; MS (ESI$^+$): *m/z* = 368 $[M+H]^+$. ^1H NMR (400 MHz, DMSO-d_6) δ ppm: 3.17 (s, 3 H), 5.21 (s, 2 H), 5.78 (d, *J* = 7.86 Hz, 1 H), 7.29–7.36 (m, 1 H), 7.60 (dd, *J* = 8.87, 5.70 Hz, 1 H), 7.91 (d, *J* = 7.98 Hz, 1 H), 8.00 (s, 1 H), 10.74 (s, 1 H). HRMS (ESI, $[M+H]^+$): calcd for $C_{15}H_{12}ClFN_3O_3S$: 368.0266, found: 368.0265.

1-{[2-(2,4-difluoro-5-hydroxyphenyl)-1,3-thiazol-5-yl]methyl}-3-methyl-1,2,3,4-

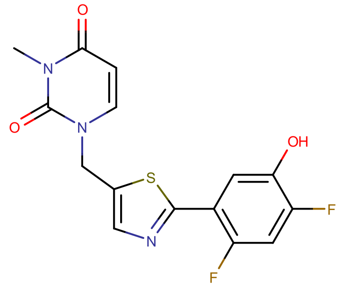

tetrahydropyrimidine-2,4-dione (**36**). Compound **36** (21 mg, 60% yield) was prepared analogously to **14**, from **S-14A** (30.0 mg, 0.10 mmol) and (2,4-difluoro-5-hydroxyphenyl)boronic acid (23.0 mg, 0.13 mmol, commercially available, CAS-RN: [2096330-91-3]). LC-MS (*method 7*): ^{t}R = 0.63 min; MS (ESI^{+}): *m/z* = 352 [M+H]$^{+}$. ^{1}H NMR (400 MHz, DMSO-d_6) δ ppm: 3.16 (s, 3 H), 5.20 (s, 2 H), 5.77 (d, *J* = 7.86 Hz, 1 H), 7.43 (t, *J* = 10.96 Hz, 1 H), 7.76 (dd, *J* = 9.76, 7.35 Hz, 1 H), 7.90 (d, *J* = 7.86 Hz, 1 H), 7.99 (d, *J* = 2.28 Hz, 1 H), 10.24 (s, 1 H). HRMS (ESI, [M+H]$^{+}$): calcd for $C_{15}H_{12}F_2N_3O_3S$: 352.0562, found: 352.0564.

1-{[2-(2-chloro-3-hydroxy-5-methylphenyl)-1,3-thiazol-5-yl]methyl}-3-methyl-1,2,3,4-tetra-

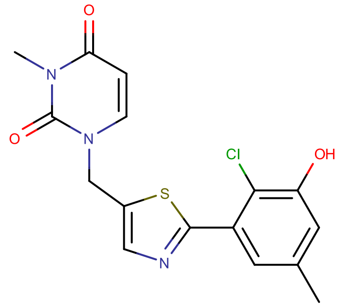

hydropyrimidine-2,4-dione (**37**). Step 1: 1-{[2-(2-chloro-3-methoxy 5-methylphenyl)-1,3-thiazol-5-yl]methyl}-3-methyl-1,2,3,4-tetra-hydropyrimidine-2,4-dione (15 mg, 24% yield) was prepared analogously to **24**, from 1-bromo-2-chloro-3-methoxy-5-methylbenzene (47.0 mg, 0.20 mmol, commercially available, CAS-RN: [1208075-75-5]) and **S-14A** (50.0 mg, 0.17 mmol). LC-MS (*method 2*): ^{t}R = 0.70 min; MS (ESI^{+}): *m/z* = 378 [M+H]$^{+}$. The product was used in the next step. Step 2: Compound **37** (6 mg, 52% yield) was prepared analogously to **35** (step 2), from 1-{[2-(2-chloro-3-methoxy-5-methylphenyl)-1,3-thiazol-5-yl]methyl}-3-methyl-1,2,3,4-tetrahydropyrimidine-2,4-dione (12.0 mg, 0.03 mmol). LC-MS (*method 2*): ^{t}R = 0.59 min; MS (ESI^{+}): *m/z* = 364 [M+H]$^{+}$. ^{1}H NMR (400

MHz, DMSO-d_6) δ ppm: 2.27 (s, 3 H), 3.16 (s, 3 H), 5.21 (s, 2 H), 5.78 (d, J = 7.86 Hz, 1 H), 6.90 (s, 1 H), 7.39 (s, 1 H), 7.87–7.95 (m, 1 H), 7.99 (s, 1 H), 10.23 (br s, 1 H).

1-{[2-(2-chloro-5-fluoro-3-hydroxyphenyl)-1,3-thiazol-5-yl]methyl}-3-methyl-1,2,3,4-tetrahydropyrimidine-2,4-dione (**38**).

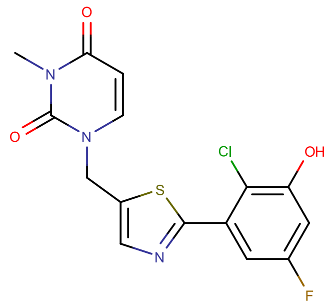

Step 1: (4-Methoxyphenyl)methanol (334 mg, 2.42 mmol, commercially available, CAS-RN: [1331-81-3]) was dissolved in DMF (1 mL) and cooled in an ice bath. Sodium hydride (60% in mineral oil, 145 mg, 2.42 mmol) was added and the grey suspension was stirred for 10 min in the ice bath. A solution of 1-bromo-2-chloro-3,5-difluorobenzene (500 mg, 2.20 mmol, commercially available, CAS-RN: [187929-82-4]) in DMF (5 mL) was then added slowly. The yellow suspension was stirred in the ice bath for 30 min, then at rt overnight. The reaction was quenched with water and the suspension was partitioned between EtOAc and water. The layers were separated, and the aqueous layer was further extracted with EtOAc. The combined organic layers were dried over $MgSO_4$, filtered and concentrated under reduced pressure. The residue was diluted with DMF, filtered, and purified by preparative HPLC (Sunfire C18, ACN, H_2O/TFA) to give 1-bromo-2-chloro-5-fluoro-3-[(4-methoxyphenyl)methoxy]benzene (**S-38A**, 490 mg, 65%). LC-MS (*method 2*): ^tR = 1.07 min; MS (ESI$^+$): m/z = 347 $[M+H]^+$. The product was used in the next step. Step 2: 1-[(2-{2-chloro-5-fluoro-3-[(4-methoxyphenyl)methoxy]phenyl}-1,3-thiazol-5-yl)methyl]-3-methyl-1,2,3,4-tetrahydropyrimidine-2,4-dione (**S-38B**, 45 mg, 22% yield) was prepared analogously to **24**, from **S-38A** (214 mg, 0.62 mmol) and **S-14A** (125 mg, 0.41 mmol). The reaction mixture was diluted with MeOH/DCM, then concentrated under reduced pressure. The residue was purified by preparative HPLC (Sunfire C18, ACN, H_2O/TFA). LC-MS (*method 2*): ^tR = 0.88 min; MS (ESI$^+$):

m/z = 488 [M+H]$^+$. The product was used in the next step. Step 3: To a solution of **S-38B** (55.0 mg, 0.11 mmol) in DCM (3 mL) was added TFA (1 mL) and the resulting mixture was stirred at rt for 2 h. The reaction mixture was concentrated under reduced pressure. The residue was dissolved in DMF, filtered, and purified by preparative HPLC (Sunfire C18, ACN, H_2O/TFA) to give **38** (28 mg, 68% yield). LC-MS (*method 2*): ^tR = 0.57 min; MS (ESI$^+$): *m/z* = 368 [M+H]$^+$. ^1H NMR (400 MHz, DMSO-d_6) δ ppm: 3.17 (s, 3 H), 5.22 (s, 2 H), 5.78 (d, *J* = 7.98 Hz, 1 H), 6.90 (dd, *J* = 9.63, 3.04 Hz, 1 H), 7.42 (dd, *J* = 9.76, 3.04 Hz, 1 H), 7.91 (d, *J* = 7.86 Hz, 1 H), 8.04 (s, 1 H), 11.11 (s, 1 H). HRMS (ESI, [M+H]$^+$): calcd for $C_{15}H_{12}ClFN_3O_3S$: 368.0266, found: 368.0268.

2.6. Synthesis of Compounds 39-44 and 46-48

1-{[5-(2,4-difluoro-3-hydroxyphenyl)-1,3,4-thiadiazol-2-yl]methyl}-3-methyl-1,2,3,4-tetra-

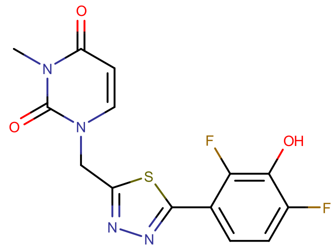

hydropyrimidine-2,4-dione (**39**). Compound **39** (6.7 mg, 19% yield) was prepared analogously to **13**, from 1-[(5-bromo-1,3,4-thiadiazol-2-yl)methyl]-3-methyl-1,2,3,4-tetrahydropyrimidine-2,4-dione (**S-13A**) (30.0 mg, 0.10 mmol) and **45F** (25.0 mg, 0.14 mmol). LC-MS (*method 7*): ^{t}R = 0.55 min; MS (ESI^{+}): *m/z* = 353 [M+H]$^{+}$. ^{1}H NMR (400 MHz, DMSO-d_6) δ ppm: 3.17 (s, 3 H), 5.47 (s, 2 H), 5.82 (d, *J* = 7.86 Hz, 1 H), 7.19–7.32 (m, 1 H), 7.58–7.70 (m, 1 H), 7.91 (d, *J* = 7.98 Hz, 1 H), 10.76 (s, 1 H). HRMS (ESI, [M+H]$^{+}$): calcd for $C_{14}H_{11}F_2N_4O_3S$: 353.0514, found: 353.0515.

1-{[2-(2,4-difluoro-3-hydroxyphenyl)-1,3-thiazol-5-yl]methyl}-3-ethyl-1,2,3,4-tetrahydro-

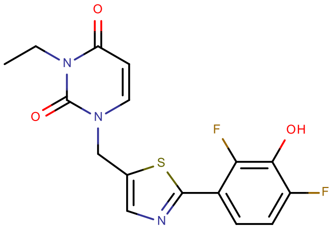

pyrimidine-2,4-dione (**40**). Step 1: Uracil (1.35 g, 12.0 mmol, commercially available, CAS-RN: [66-22-8]), hexamethyl disilazane (2.76 mL, 13.2 mmol) and chloro trimethyl silane (0.76 mL, 6.02 mmol) were suspended in ACN (10.0 mL), flushed with N_2, stirred at 140 °C for 5 h, then concentrated under reduced pressure. The residue was dissolved in ACN (22.0 mL). A solution of 2-bromo-5-(bromomethyl)-1,3-thiazole (2.90 g, 11.3 mmol, commercially available, CAS-RN: [131748-91-9]) in ACN (13.0 mL) was added slowly over a period of 20 min with stirring at 0 °C, then stirring was continued at 80 °C overnight. The reaction was allowed to cool to rt, then quenched with aq. $NaHCO_3$ (20 mL). The mixture was extracted with DCM (5 x). The combined organic layers were dried over Na_2SO_4, filtered, and concentrated. The residue was treated with diethyl ether, the product was collected by suction filtration and dried to provide 1-

[(2-bromo-1,3-thiazol-5-yl)methyl]-1,2,3,4-tetrahydropyrimidine-2,4-dione (**S-40A**, 2.66 g, 77% yield). LC-MS (*method 3*): ^tR = 0.67 min; MS (ESI$^+$): *m/z* = 288/290 [M+H]$^+$. The product was used in the next step without further purification. Step 2: **S-40A** (60.0 mg, 0.21 mmol) was dissolved in DMF (3 mL). Iodoethane (25.0 µL, 0.31 mmol) and potassium carbonate (58.0 mg, 0.42 mmol) were added and the resulting mixture was stirred at 50 °C for 1 h, then diluted with MeOH, filtered and purified by semi-preparative HPLC (XBridge C18, ACN, NH_4OH) to yield 1-[(2-bromo-1,3-thiazol-5-yl)methyl]-3-ethyl-1,2,3,4-tetrahydropyrimidine-2,4-dione (**S-40B**, 56 mg, 85% yield). LC-MS (*method 3*): ^tR = 0.88 min; MS (ESI$^+$): *m/z* = 316/318 [M+H]$^+$. The product was used in the next step. Step 3: The final compound **40** (82 mg, 55% yield) was prepared analogously to **45** (step d), from **S-40B** (130 mg, 0.41 mmol) and **45F** (110 mg, 0.63 mmol). LC-MS (*method 7*): ^tR = 0.66 min; MS (ESI$^+$): *m/z* = 366 [M+H]$^+$. ^{1}H NMR (400 MHz, DMSO-d_6) δ ppm: 1.09 (t, *J* = 7.03 Hz, 3 H), 3.84 (q, *J* = 7.05 Hz, 2 H), 5.20 (s, 2 H), 5.75 (d, *J* = 7.98 Hz, 1 H), 7.10–7.29 (m, 1 H), 7.59 (td, *J* = 8.33, 6.02 Hz, 1 H), 7.88 (d, *J* = 7.98 Hz, 1 H), 7.99 (d, *J* = 2.28 Hz, 1 H), 10.60 (br s, 1 H). HRMS (ESI, [M+H]$^+$): calcd for $C_{16}H_{14}F_2N_3O_3S$: 366.0718, found: 366.0715.

1-{[5-(2,4-difluoro-3-hydroxyphenyl)-1,3,4-thiadiazol-2-yl]methyl}-3-ethyl-1,2,3,4-tetrahydro-pyrimidine-2,4-dione (**41**). Step 1: **45B** (250 mg, 0.92 mmol) and uracil (205 mg, 1.83 mmol, commercially available, CAS-RN: [66-22-8]) were dissolved in DMF (5 mL). Potassium carbonate (316 mg, 2.29 mmol) was added and the resulting mixture was stirred at rt for 2 h. Iodoethane (184 µL, 2.29 mmol) was added and the resulting mixture was stirred at 50 °C for 1 h. The reaction mixture was filtered, diluted with a small amount of H_2O, acidified with TFA and

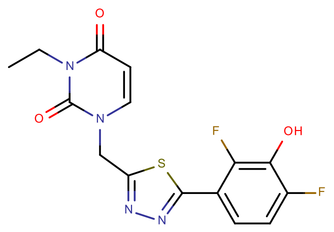

purified by preparative HPLC (Sunfire C18, ACN, H_2O/TFA) to give 1-[(5-bromo-1,3,4-thiadiazol-2-yl)methyl]-3-ethyl-1,2,3,4-tetrahydropyrimidine-2,4-dione (**S-41A**, 69 mg, 24% yield). LC-MS (*method 3*): ^tR = 0.76 min; MS (ESI^+): *m/z* = 317 $[M+H]^+$. Step 2: Compound **41** (60 mg, 78% yield) was prepared analogously to **45** (Step d), from **S-41A** (67.0 mg, 0.21 mmol) and **45** (55.0 mg, 0.32 mmol). LC-MS (*method 3*): ^tR = 0.84 min; MS (ESI^+): *m/z* = 367 $[M+H]^+$. ^1H NMR (400 MHz, DMSO-d_6) δ ppm: 1.09 (t, *J* = 7.03 Hz, 3 H), 3.84 (q, *J* = 7.05 Hz, 2 H), 5.46 (s, 2 H), 5.80 (d, *J* = 7.86 Hz, 1 H), 7.18–7.32 (m, 1 H), 7.64 (ddd, *J* = 8.87, 7.54, 5.89 Hz, 1 H), 7.89 (d, *J* = 7.98 Hz, 1 H), 10.76 (br s, 1 H). HRMS (ESI, $[M+H]^+$): calcd for $C_{15}H_{13}F_2N_4O_3S$: 367.0671, found: 367.0670.

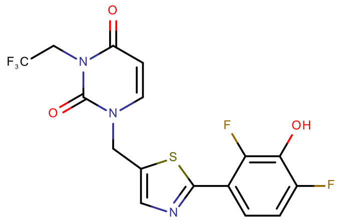

1-{[2-(2,4-difluoro-3-hydroxyphenyl)-1,3-thiazol-5-yl]methyl}-3-(2,2,2-trifluoroethyl)-1,2,3,4-tetrahydropyrimidine-2,4-dione (**42**). Step 1: **S-40A** (100 mg, 0.35 mmol) was dissolved in DMF (4 mL). 1,1,1-trifluoro-2-iodoethane (287 mg, 1.37 mmol, commercially available, CAS-RN: [353-83-3]) and potassium carbonate (106 mg, 0.76 mmol) were added and the resulting mixture was stirred at 50 °C for 1 h, then at 80 °C for 1 h and finally at 100 °C for 1.5 h. The reaction was filtered through Celite®, diluted with a small amount of H_2O, acidified with TFA and purified by preparative HPLC (Sunfire C18, ACN, H_2O/TFA) to provide 1-[(2-bromo-1,3-thiazol-5-yl)methyl]-3-(2,2,2-trifluoroethyl)-1,2,3,4-tetrahydropyrimidine-2,4-dione (**S-42A**, 70 mg, 55% yield). LC-MS (*method 2*): ^tR = 0.95 min; MS (ESI^+): *m/z* = 370 $[M+H]^+$. The product was used in the next step. Step 2: Compound **42** (31 mg, 39% yield) was prepared analogously to **45** (Step d), from **S-42A** (70.0 mg, 0.19 mmol) and **45F** (49.0 mg, 0.28 mmol). LC-MS (*method 3*): ^tR = 0.95 min; MS (ESI^+): *m/z* = 420 $[M+H]^+$. ^1H NMR (400 MHz, DMSO-d6) δ ppm: 4.64 (q,

J = 9.12 Hz, 2 H), 5.24 (s, 2 H), 5.87 (d, *J* = 7.98 Hz, 1 H), 7.12–7.24 (m, 1 H), 7.60 (ddd, *J* = 8.90, 7.89, 6.02 Hz, 1 H), 7.93–8.09 (m, 2 H), 10.59 (br s, 1 H). HRMS (ESI, $[M+H]^+$): calcd for $C_{16}H_{11}F_5N_3O_3S$: 420.0436, found: 420.0434.

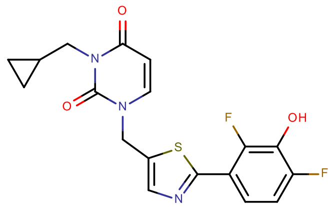

3-(cyclopropylmethyl)-1-{[2-(2,4-difluoro-3-hydroxyphenyl)-1,3-thiazol-5-yl]methyl}-1,2,3,4-tetrahydropyrimidine-2,4-dione (**43**). Step 1: **S-40A** (100 mg, 0.35 mmol) was dissolved in DMF (4 mL). (Iodomethyl)cyclopropane (76.0 mg, 0.42 mmol) and potassium carbonate (106 mg, 0.76 mmol) were added and the resulting mixture was stirred at 50 °C for 1 h. The reaction mixture was filtered through Celite®, diluted with a small amount of H_2O, acidified with TFA and purified by preparative HPLC (Sunfire C18, ACN, H_2O/TFA) to yield 1-[(2-bromo-1,3-thiazol-5-yl)methyl]-3-(cyclopropylmethyl)-1,2,3,4-tetrahydropyrimidine-2,4-dione (**S-43A**, 88 mg, 74% yield). LC-MS (*method 3*): ^tR = 0.96 min; MS (ESI^+): *m/z* = 342 $[M+H]^+$. The product was used in the next step. Step 2: Compound **43** (46 mg, 46% yield) was prepared analogously to **45** (Step d), from **S-43A** (87.0 mg, 0.25 mmol) and **45F** (66.0 mg, 0.38 mmol). LC-MS (*method 3*): ^tR = 0.95 min; MS (ESI^+): *m/z* = 392 $[M+H]^+$. ^1H NMR (400 MHz, DMSO-d_6) δ ppm: 0.27–0.49 (m, 4 H), 1.06–1.20 (m, 1 H), 3.70 (d, *J* = 7.10 Hz, 2 H), 5.21 (s, 2 H), 5.77 (d, *J* = 7.86 Hz, 1 H), 7.11–7.24 (m, 1 H), 7.60 (td, *J* = 8.40, 6.02 Hz, 1 H), 7.90 (d, *J* = 7.86 Hz, 1 H), 8.00 (d, *J* = 2.15 Hz, 1 H), 10.59 (s, 1 H). HRMS (ESI, $[M+H]^+$): calcd for $C_{18}H_{16}F_2N_3O_3S$: 392.0875, found: 392.0877.

1-{[2-(2,4-difluoro-3-hydroxyphenyl)-1,3-thiazol-5-yl]methyl}-3-ethyl-5-methyl-1,2,3,4-tetra-

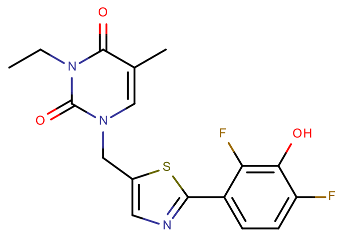

hydropyrimidine-2,4-dione (**44**). Step 1: 1-[(2-bromo-1,3-thiazol-5-yl)methyl]-5-methyl-1,2,3,4-tetrahydropyrimidine-2,4-dione (**S-44A**, 1.05 g, 88% yield) was prepared analogously to **45D** (Step b compound **45**), from thymin (**45C**) (500 mg, 3.97 mmol, commercially available, CAS-RN: [65-71-4]) and (2-bromo-1,3-thiazol-5-yl)methanol (1.12 g, 4.36 mmol, commercially available, CAS-RN: [687636-93-7]) at 80 °C for 2 h. LC-MS (*method 3*): ^{t}R = 0.74 min; MS (ESI^{+}): *m/z* = 302 [M+H]$^{+}$. The product was used in the next step. Step 2: **S-44A** (24.0 mg, 0.08 mmol) was dissolved in DMF (2 mL). Potassium carbonate (22.0 mg, 0.16 mmol) and iodoethane (19.0 µL, 0.24 mmol) were added and the resulting mixture was stirred at 50 °C for 1.5 h. Upon completion, the reaction was quenched with H_2O, acidified with TFA and purified by preparative HPLC (Sunfire C18, ACN, H_2O/TFA) to provide 1-[(2-bromo-1,3-thiazol-5-yl)methyl]-3-ethyl-5-methyl-1,2,3,4-tetrahydropyrimidine-2,4-dione (**S-44B**, 18 mg, 69% yield). LC-MS (*method 3*): ^{t}R = 0.93 min; MS (ESI^{+}): *m/z* = 330 [M+H]$^{+}$. The product was used in the next step. Step 3: Compound **44** (13 mg, 63% yield) was prepared analogously to **45** (Step d), from **S-44B** (18.0 mg, 0.06 mmol) and **45F** (25 mg, 0.15 mmol). LC-MS (*method 3*): ^{t}R = 0.93 min; MS (ESI^{+}): *m/z* = 380 [M+H]$^{+}$. ^{1}H NMR (400 MHz, DMSO-d_6) δ ppm: 1.09 (t, *J* = 7.03 Hz, 3 H), 1.81 (s, 3 H), 3.86 (q, *J* = 7.01 Hz, 2 H), 5.16 (s, 2 H), 7.13–7.24 (m, 1 H), 7.59 (td, *J* = 8.36, 6.08 Hz, 1 H), 7.77 (d, *J* = 0.89 Hz, 1 H), 7.99 (d, *J* = 2.03 Hz, 1 H), 10.59 (br s, 1 H).

1-{[5-(2,4-difluoro-3-hydroxyphenyl)-1,3,4-thiadiazol-2-yl]methyl}-3-ethyl-6-methyl-1,2,3,4-tetrahydropyrimidine-2,4-dione (**46**).

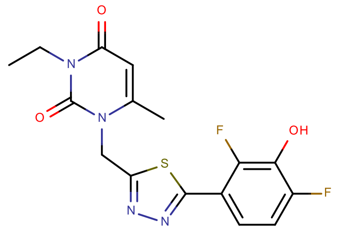

Compound **46** was prepared analogously to **41** in two steps. Step 1: 1-[(5-bromo-1,3,4-thiadiazol-2-yl)methyl]-3-ethyl-6-methyl-1,2,3,4-tetrahydro-pyrimidine-2,4-dione (**S-46A**, 34 mg, 14% yield) was obtained from compound **45B** (200 mg, 0.73 mmol), 6-methyluracil (185 mg, 1.47 mmol, commercially available, CAS-RN: [626-48-2]) and iodoethane (147 µL, 1.83 mmol). LC-MS (*method 3*): ^{t}R = 0.81 min; MS (ESI+): m/z = 331 [M+H]+. The product was used in the next step. Step 2: Compound **46** (23 mg, 63% yield) was prepared analogously to **45** (step d), from **S-46A** (32.0 mg, 0.10 mmol) and **45F** (25.0 mg, 0.15 mmol). LC-MS (*method 3*): ^{t}R = 0.89 min; MS (ESI+): m/z = 381 [M+H]+. ^{1}H NMR (400 MHz, DMSO-d_6) δ ppm: 1.09 (t, *J* = 7.03 Hz, 3 H), 2.37 (s, 3 H), 3.84 (q, *J* = 6.97 Hz, 2 H), 5.51 (s, 2 H), 5.73 (s, 1 H), 7.20–7.32 (m, 1 H), 7.65 (ddd, *J* = 8.93, 7.54, 5.83 Hz, 1 H), 10.76 (br s, 1 H).

1-{[5-(2,4-difluoro-3-hydroxyphenyl)-1,3,4-thiadiazol-2-yl]methyl}-3-ethyl-1H,2H,3H,4H,5H,6H,7H-cyclopenta[d]pyrimidine-2,4-dione (**47**).

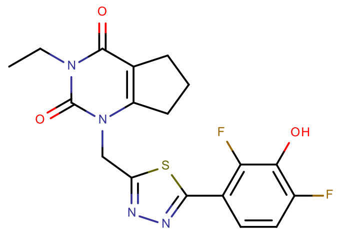

Step 1: (5-bromo-1,3,4-thiadiazol-2-yl)methanol (4.00 g, 20.5 mmol, commercially available, CAS-RN: [1339055-00-3]) and **45F** (4.60 g, 24.6mmol) were suspended in H_2O (10 mL) and EtOH (50 mL). Sodium carbonate (5.43 g, 51.3 mmol) was added and the mixture was degassed with N_2. Pd-PEPPSI 2Me-IPent Cl (0.86 g, 1.03 mmol) was added and the resulting mixture was stirred at 80 °C overnight. The reaction mixture was filtered through Celite®, the filtrate acidified with TFA while cooling in an ice bath. Next, the mixture was concentrated, the precipitate filtered and washed with

EtOH/H_2O followed by diethyl ether, and then dried in a vacuum drying chamber at 60 °C to provide 2,6-difluoro-3-[5-(hydroxymethyl)-1,3,4-thiadiazol-2-yl]phenol (**S-47A**, 3.37 g, 67% yield). LC-MS (*method 3*): ^{t}R = 0.72 min; MS (ESI^{+}): *m/z* = 245 [M+H]$^{+}$. The product was used in the next step. Step 2: **S-47A** (3.37 g, 13.8 mmol) and potassium carbonate (2.86 g, 20.7 mmol) were suspended in ACN (100 mL). 1-(Chloromethyl)-4-methoxybenzene (2.07 mL, 15.2 mmol, commercially available, CAS-RN: [824-94-2]) was added and the mixture was stirred at 80 °C for 8 h, then concentrated under reduced pressure. The residue was suspended in water, the precipitate was filtered and dried in a vacuum drying chamber at 60 °C to give (5-{2,4-difluoro-3-[(4-methoxyphenyl)methoxy]phenyl}-1,3,4-thiadiazol-2-yl)methanol (**S-47B**, 4.91 g, 98% yield). LC-MS (*method 3*): ^{t}R = 1.02 min; MS (ESI^{+}): *m/z* = 365 [M+H]$^{+}$. The product was used in the next step. Step 3: **S-47B** (3.01 g, 8.26 mmol) and triethylamine (1.73 mL, 12.4 mmol) were suspended in DCM (25 mL) and cooled to 0 °C. Methane sulfonyl chloride (0.96 mL, 12.4 mmol) was added and the resulting mixture was stirred at 0 °C for 10 min, then at rt for 5 h. The reaction mixture was diluted with aq. citric acid, the organic layer was separated, dried over Na_2SO_4, filtered and concentrated under reduced pressure to give 2-(chloromethyl)-5-{2,4-difluoro-3-[(4-methoxyphenyl)methoxy]phenyl}-1,3,4-thiadiazole (**S-47C**, 3.84 g, quantitative yield). LC-MS (*method 3*): ^{t}R = 1.10 min; MS (ESI^{+}): *m/z* = 383 [M+H]$^{+}$. The product was used in the next step. Step 4: 1H,2H,3H,4H,5H,6H,7H-cyclopenta[d]pyrimidine-2,4-dione (100 mg, 0.62 mmol, commercially available, CAS-RN: [5466-00-2]) was suspended in ACN (3 mL). *N*,*O*-bis(trimethylsilyl) acetamide (382 µL, 1.56 mmol) was added and the resulting mixture was stirred at rt overnight. **S-47C** (300 mg, 0.67 mmol) was dissolved in ACN (2.00 mL) and added to the reaction mixture. Tetrabutylammonium iodide (90.0 mg, 0.25 mmol) was added and the resulting mixture was stirred at rt for 30 min, then at 80 °C for 20 h. H_2O (2 mL) was slowly added, the

precipitate was filtered and washed with water, ACN and diethyl ether to give 1-[(5-{2,4-difluoro-3-[(4-methoxyphenyl)methoxy]phenyl}-1,3,4-thiadiazol-2-yl)methyl]-1H,2H,3H,4H,5H,6H,7H-cyclopenta[d]pyrimidine-2,4-dione (**S-47D**, 93 mg, 30% yield). LC-MS (*method 3*): ^{t}R = 0.96 min; MS (ESI$^+$): *m/z* = 499 [M+H]$^+$. The product was used in the next step. Step 5: **S-47D** (90.0 mg, 0.18 mmol) was dissolved in DMF (1 mL) and potassium carbonate (50.0 mg, 0.36 mmol) was added, followed by iodoethane (22 µL, 0.27 mmol). The reaction mixture was stirred at 80 °C for 2 h. Next, the reaction mixture was cooled to rt before TFA (2 mL) was added. The resulting mixture was stirred at rt for 5 days. The mixture was purified by semi-preparative HPLC (Sunfire C18, ACN, H2O/TFA) to give **47** (27 mg, 37% yield). LC-MS (*method 3*): ^{t}R = 0.84 min; MS (ESI$^+$): *m/z* = 407 [M+H]$^+$. ^{1}H NMR (400 MHz, DMSO-d_6) δ ppm: 1.09 (t, *J* = 6.97 Hz, 3 H), 2.01 (quin, *J* = 7.54 Hz, 2 H), 2.60 (br t, *J* = 7.35 Hz, 2 H), 3.03 (br t, *J* = 7.60 Hz, 2 H), 3.85 (q, *J* = 6.97 Hz, 2 H), 5.44 (s, 2 H), 7.09–7.42 (m, 1 H), 7.64 (ddd, *J* = 8.90, 7.51, 5.89 Hz, 1 H), 10.76 (br s, 1 H).

1-{[5-(2,4-difluoro-3-hydroxyphenyl)-1,3,4-thiadiazol-2-yl]methyl}-3-ethyl-1,2,3,4-tetra-hydroquinazoline-2,4-dione (**48**).

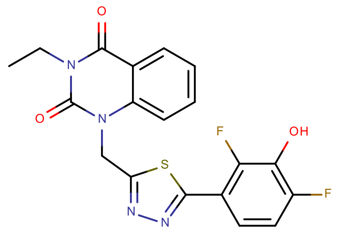

Compound **48** (9 mg, quantitative yield) was prepared analogously to **47** in two steps (step 4 and step 5) from **S-47C** (300 mg, 0.67 mmol) and 1,2,3,4-tetrahydroquinazoline-2,4-dione (100 mg, 0.62 mmol, commercially available, CAS-RN: [86-96-4]). LC-MS (*method 3*): ^{t}R = 0.90 min; MS (ESI$^+$): *m/z* = 417 [M+H]$^+$. ^{1}H NMR (400 MHz, DMSO-d_6) δ ppm: 1.20 (t, *J* = 7.03 Hz, 3 H), 4.04 (q, *J* = 7.05 Hz, 2 H), 5.84 (s, 2 H), 7.24 (ddd, *J* = 10.30, 8.90, 1.84 Hz, 1 H), 7.31–7.37 (m, 1 H), 7.59–7.64 (m, 1 H), 7.65 (d, *J* = 8.24 Hz, 1 H), 7.76–7.81 (m, 1 H), 8.10 (dd, *J* = 7.86, 1.52 Hz, 1 H), 10.75 (br s, 1 H).

2.7. Synthesis of Negative Probe 49

1-{[5-(2,4-difluoro-3-methoxyphenyl)-1,3,4-thiadiazol-2-yl]methyl}-3-ethyl-5-methyl-1,2,3,4-

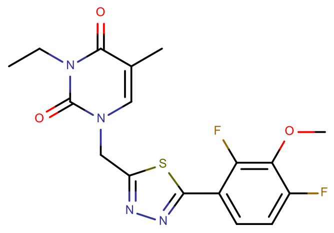

tetrahydropyrimidine-2,4-dione (**49**). Compound **45** (100 mg, 0.26 mmol) was dissolved in DMF (3 mL), and potassium carbonate (145 mg, 1.05 mmol) was added followed by iodomethane (38.5 µL, 0.53 mmol). The brown suspension was stirred at rt for 16 h. The reaction mixture was diluted with DMF and water, filtered, and purified by preparative HPLC (Sunfire C18, ACN, H2O/TFA) to provide **49** (52 mg, 50% yield). LC-MS (*method 2*): ^{t}R = 0.74 min; MS (ESI^{+}): m/z = 395 $[M+H]^{+}$. ^{1}H NMR (400 MHz, DMSO-d_6) δ ppm: 1.09 (t, J = 7.03 Hz, 3 H), 1.84 (d, J = 1.01 Hz, 3 H), 3.86 (q, J = 7.10 Hz, 2 H), 4.00 (s, 3 H), 5.43 (s, 2 H), 7.37 (ddd, J = 10.55, 9.03, 1.84 Hz, 1 H), 7.80 (d, J = 1.14 Hz, 1 H), 7.92 (ddd, J = 9.00, 7.73, 5.83 Hz, 1 H).

3. Selectivity Panel Data for 45 (BI-3231)

Table S2. SafetyScreen44 panel, Cerep

Probe name	TARGET	%CTRL_@10uM	TARGET_TYPE	MODE_OF_ACTION	ASSAY_TECHNOLOGY
BI-3231	5HT1B(H)	93	GPCR_CLASS_A	antagonist	Scintillation counting
BI-3231	CB1(HU)_AGON	101	GPCR_CLASS_A	agonist	Scintillation counting
BI-3231	COX-1	76	Hydrolase under EC 3	inhibitor	Fluorimetry
BI-3231	COX-2	49	Hydrolase under EC 3	inhibitor	Fluorimetry
BI-3231	KAPPA(KOP)_HU	94	GPCR_CLASS_A	inhibitor	Radioactive Assay
BI-3231	5HT1A/H	101	GPCR_CLASS_A		Radioactive Assay
BI-3231	5HT2AH_AGON	84	GPCR_CLASS_A	agonist	Scintillation counting
BI-3231	5HT2B/H AG	83	GPCR_CLASS_A	agonist	Radioactive Assay
BI-3231	5HT3/H	106	Ligand-gated ion channels	inhibitor	Radioactive Assay
BI-3231	A2A/H	103	GPCR_CLASS_A	antagonist	Radioligand Binding
BI-3231	ACE(HU_AMTCH400)	99			Photometry
BI-3231	ALPHA1AH_ANTAG	107	GPCR_CLASS_A	antagonist	Scintillation counting
BI-3231	ALPHA2A/HU	113	GPCR_CLASS_A	inhibitor	Radioactive Assay
BI-3231	ANDROGEN/H	102	Steroid hormone receptors		Radioactive Assay
BI-3231	BETA1/HUM	95	GPCR_CLASS_A		Radioactive Assay
BI-3231	BETA2/HUM	99	GPCR_CLASS_A	antagonist	Radioactive Assay
BI-3231	BZD/CENTR/R	115	Ligand-gated ion channels		Radioactive Assay
BI-3231	CA+/DHPSI/R	84	Voltage-gated ion channels	inhibitor	Radioactive Assay
BI-3231	CB2/PERIPH/H	98	GPCR_CLASS_A		Radioactive Assay
BI-3231	CCKA/H	78	GPCR_CLASS_A		Radioactive Assay
BI-3231	D1/H	105	GPCR_CLASS_A	antagonist	Radioactive Assay
BI-3231	D2SH_AGON	84	GPCR_CLASS_A	agonist	Scintillation counting
BI-3231	DATRANS/HUM	82	SLC superfamily of solute carriers	inhibitor	Radioactive Assay
BI-3231	DELTA2/H	95	GPCR_CLASS_A	inhibitor	Radioactive Assay
BI-3231	ETA/H	97	GPCR_CLASS_A		Radioactive Assay
BI-3231	GCORTICOID/H	96	Steroid hormone receptors	inhibitor	Radioactive Assay

BI-3231	H1/PYRIL/HS	84	GPCR_CLASS_A		Radioactive Assay
BI-3231	H2/APT/HS	105	GPCR_CLASS_A	antagonist	Radioactive Assay
BI-3231	HERG_DOFETILIDE	107	Voltage-gated ion channels	inhibitor	Scintillation counting
BI-3231	K+/VOLT/RA	103	Voltage-gated ion channels	inhibitor	Radioactive Assay
BI-3231	LCK_CE	91	Kinase under EC2.7	inhibitor	lanthanide chelation excitation
BI-3231	M1/H	99	GPCR_CLASS_A		Radioactive Assay
BI-3231	M2/H	106	GPCR_CLASS_A	antagonist	Radioactive Assay
BI-3231	M3/H	96	GPCR_CLASS_A		Radioactive Assay
BI-3231	MAO-A_ANTAG	104	Oxidoreductase under EC 1	antagonist	Scintillation counting
BI-3231	MU/H	87	GPCR_CLASS_A	inhibitor	Radioactive Assay
BI-3231	NA+/SITE2/R	73	Voltage-gated ion channels		Radioactive Assay
BI-3231	NEUP/H	88	SLC superfamily of solute carriers	inhibitor	Radioactive Assay
BI-3231	NMDA/R	104	Ligand-gated ion channels	inhibitor	Radioactive Assay
BI-3231	N_NEURO_A4B2	109	GPCR_CLASS_A	agonist	Radioligand Binding
BI-3231	PDE3A	94	Hydrolase under EC 3	inhibitor	HTRF-homogeneous time resolved fluorescence
BI-3231	PDE4D2	68	Hydrolase under EC 3	inhibitor	Scintillation counting
BI-3231	SLC6A4/H	106	SLC superfamily of solute carriers	antagonist	Scintillation counting
BI-3231	V1A/HUM	100	GPCR_CLASS_A	inhibitor	Radioactive Assay

4. Sequence Alignment for Homology Modelling

```
1FDU (B1)   1 ----ARTVVLITGCSSGIGLHLAVRLASDPSQSFKVYATLRDLKTQGRLWEAARALACPPGSLETLQLDVRDSKSVAAAR   76
HSD17B13    1 RKSVAGEIVLITGAGHGIGRQTTYEFAKRQS-----ILVLWDINKRG-VEETAAECRKLGVTAHAYVVDCSNREEIYRSL   74

1FDU (B1)  77 ERVT--EGRVDVLVCNAGLGLLGPLEALGEDAVASVLDVNVVGTVRMLQAFLPDMKRRGSGRVLVTGSVGGLMGLPFNDV  154
HSD17B13   75 NQVKKEVGDVTIVVNNAGTVYPADLLSTKDEEITKTFEVNILGHFWITKALLPSMMERNHGHIVTVASVCGHEGIPYLIP  154

1FDU (B1) 155 YCASKFALEGLCESLAVLLLPF---GVHLSLIECGPVHTAFMEKVLGSPEEVLDRTDIHTFHRFYQYLALSKQVFREAAQ  231
HSD17B13  155 YCSSKFAAVGFHRGLTSELQALGKTGIKTSCLCPVFVNTGFTKNPSTRLWPVLE-TDEVVRSLIDGILTNKKMIFVPSYI  233

1FDU (B1) 232 NPEEVAEVFLTALRAPKPTLRYFTTERFLPL-LRMRLDDPSGSNYVTAMHREVFG-------------------------  285
HSD17B13  234 -------NIFLRLQK-------FLPER-ASAILNRMQNIQFEAVVGHKIKMK----------------------------  270
```

Figure S2. Sequence alignment of HSD17B13 and HSD17B1 (Xray pdb code: 1FDU) employed for homology modelling.

5. References

(S1) Armarego, W. L. F.; Chai, C. L. L. Purification of Laboratory Chemicals, 5th ed.; Butterworth Heinemann: Oxford, U.K., **2003**.

6. UPLC traces for key compounds

Compd	HPLC-Purity	Method	Mass detected	Mass Expected
**1**	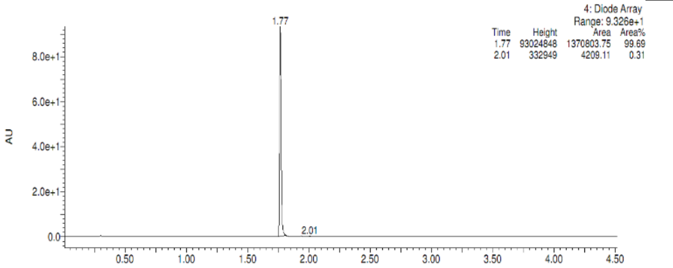	4	311.27	310.31
**2**	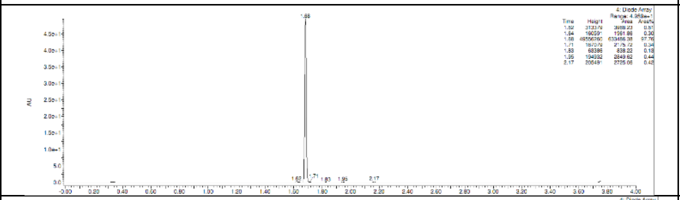	4	345.26	344.32
**3**	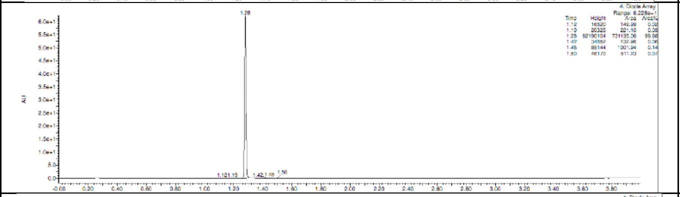	4	338.29	337.33
**4**	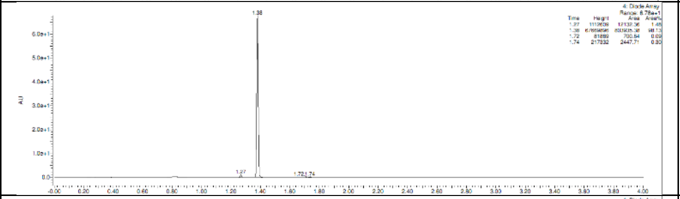	4	352.31	351.36
**5**	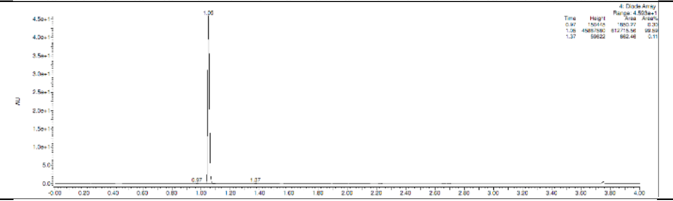	4	311.25	310.31
**6**	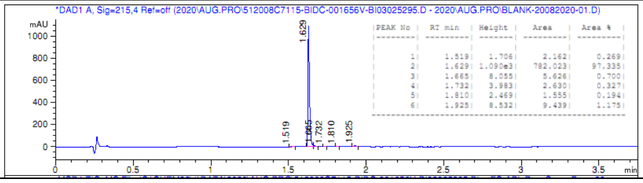	5	312.10	311.30

**7**	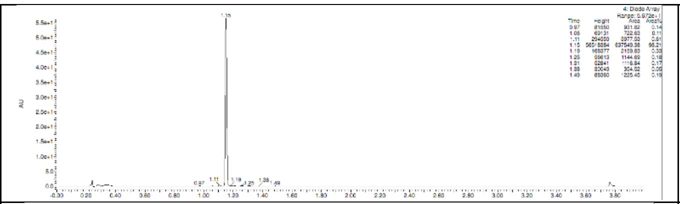	4	312.26	311.30
**8**	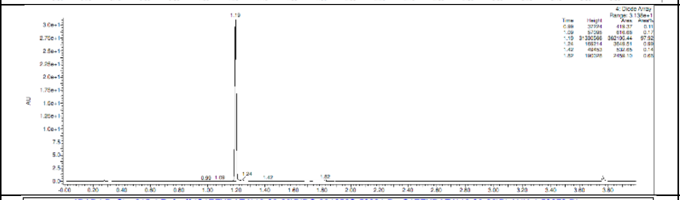	4	285.22	284.27
**9**	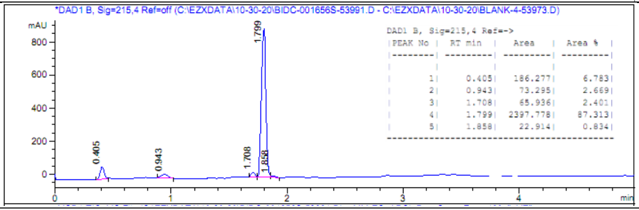	6	317.00	316.07
**10**	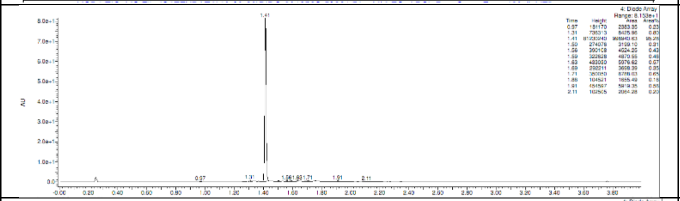	4	335.31	334.33
**11**	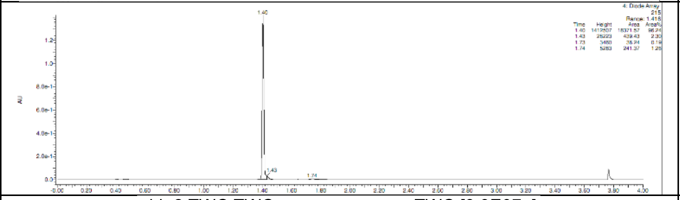	4	335.26	334.33
**12**	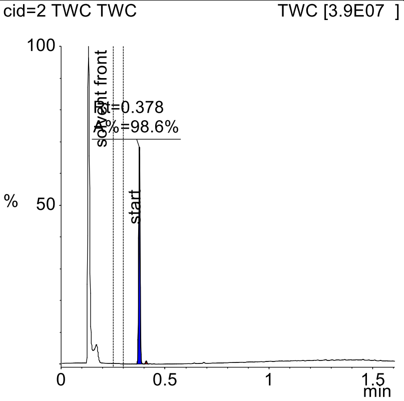	8	257.1	256.08

**13**	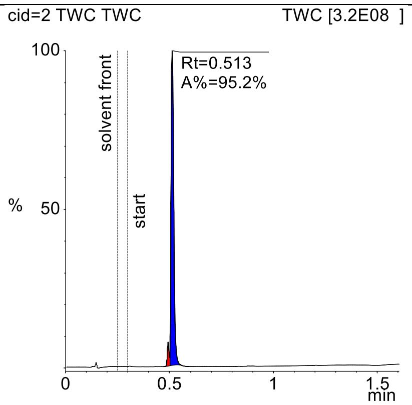	7	317.25	316.34
**14**	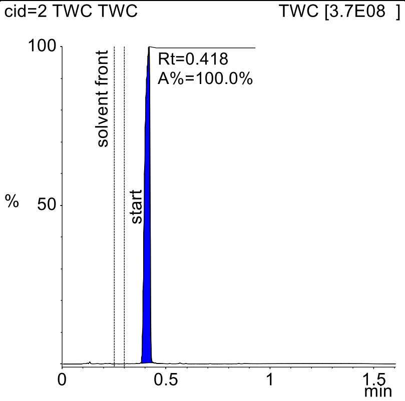	8	316.2	315.35
**15**	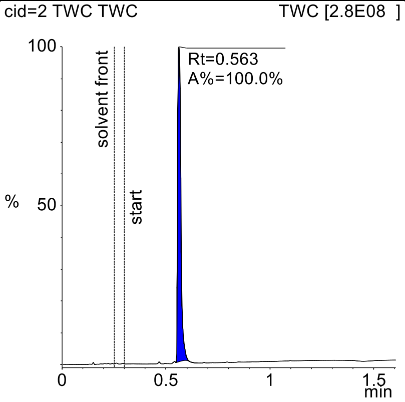	7	316	315.07
**16**	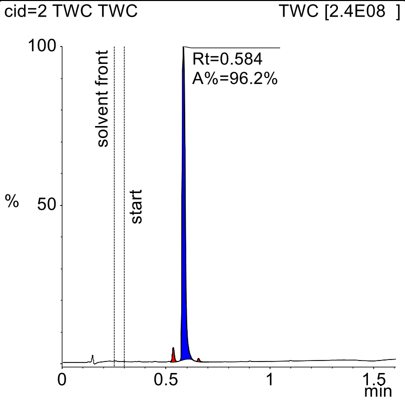	7	316.18	315.35

**17**	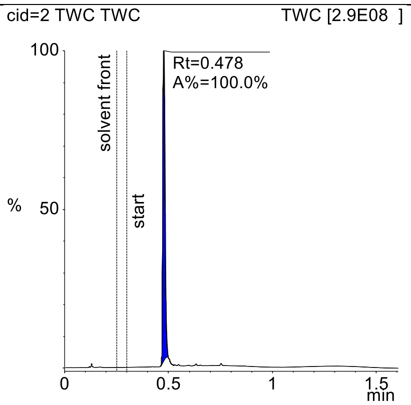	8	299.96	299.28
**18**	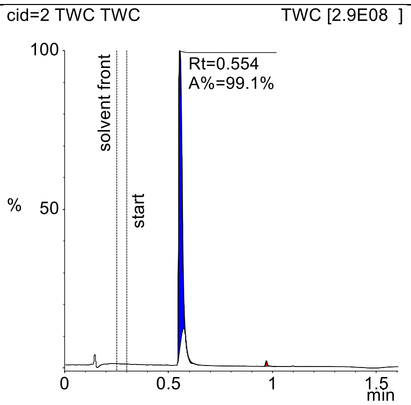	7	300.11	299.28
**19**	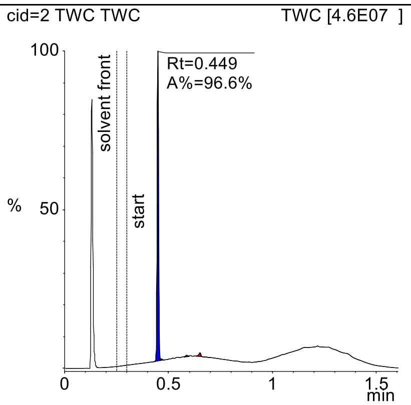	8	300	299.09
**20**	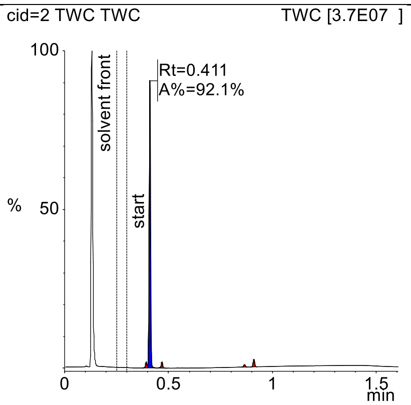	8	300	299.09

**21**	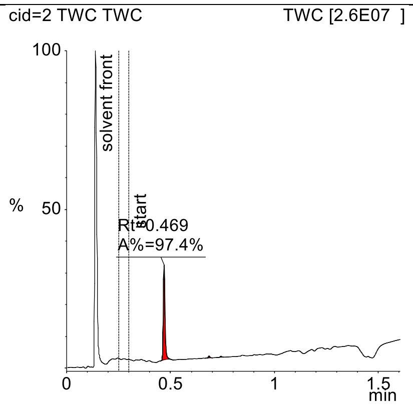	7	300.2	299.10
**22**	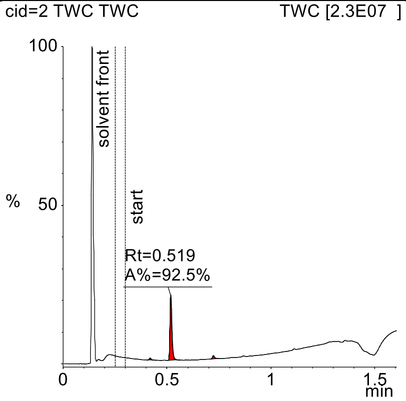	7	311.1	310.11
**23**	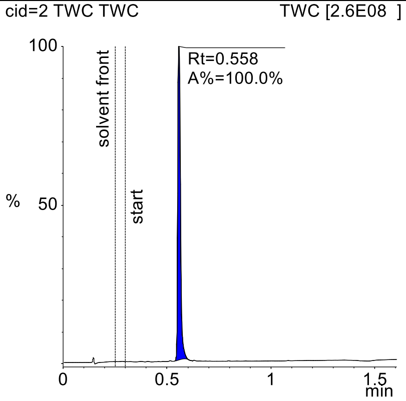	7	334	333.06
**24**	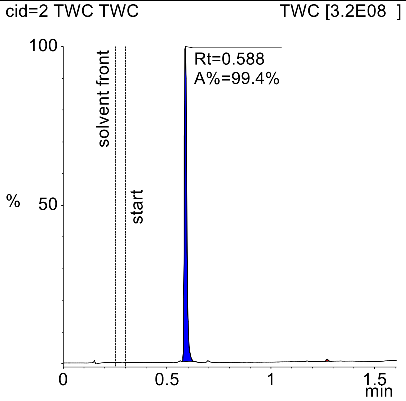	7	350.05	349.79

**25**	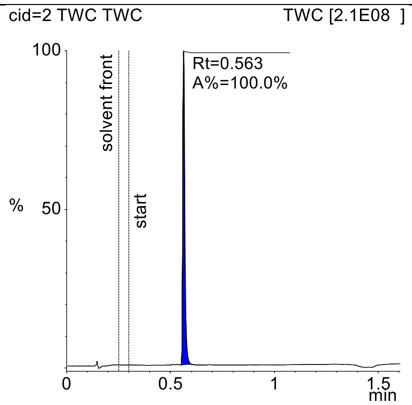	7	330	329.08
**26**	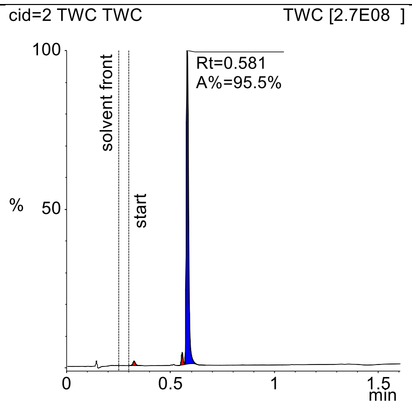	7	334	333.06
**27**	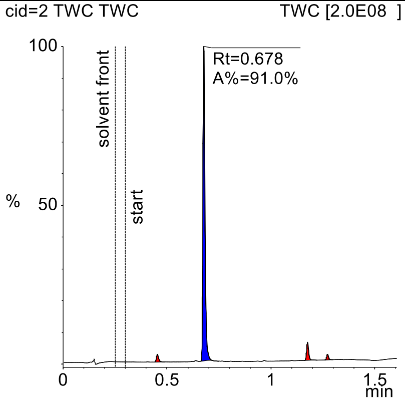	7	350.06	349.79
**28**	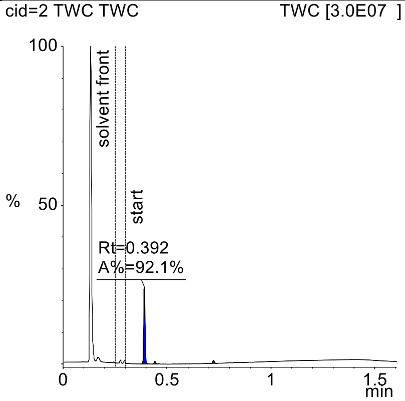	8	384.1	383.06

**30**	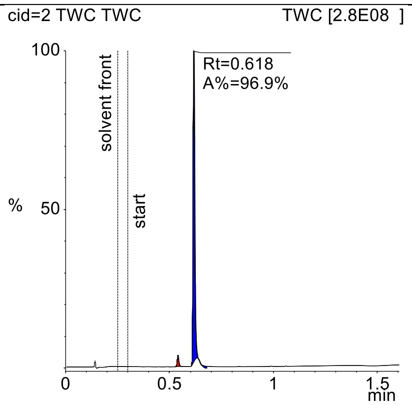	7	334.04	333.34
**31**	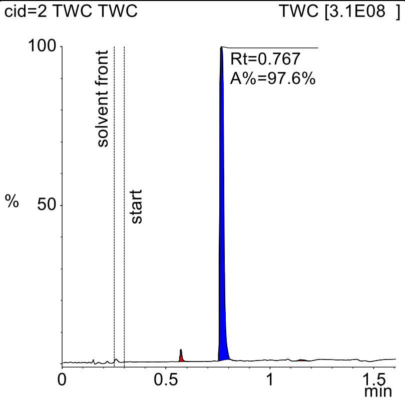	7	384.1	383.06
**32**	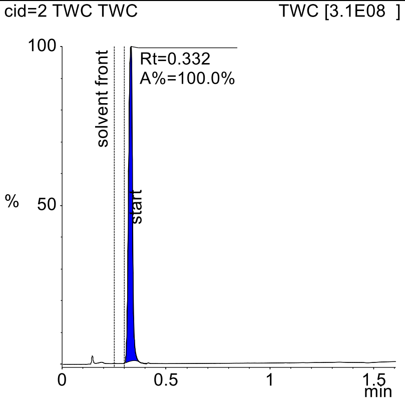	7	317	316.06
**33**	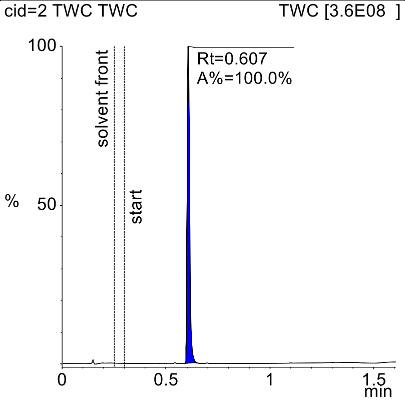	7	334.1	333.34

**34**	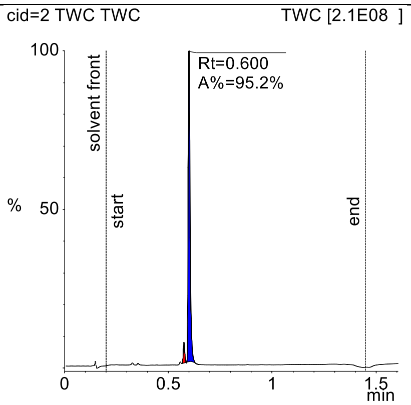	7	352	351.0
**35**	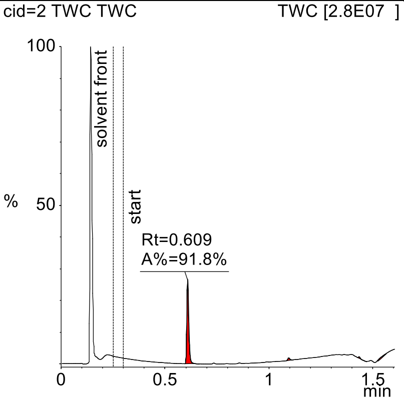	7	368.2	367.02
**36**	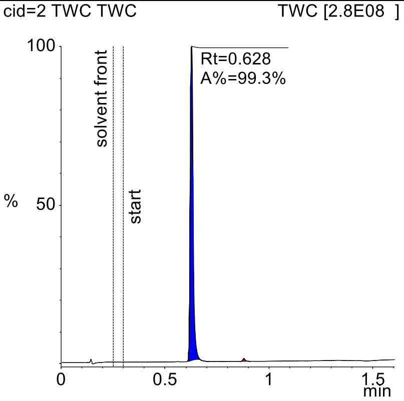	7	352	351.05
**37**	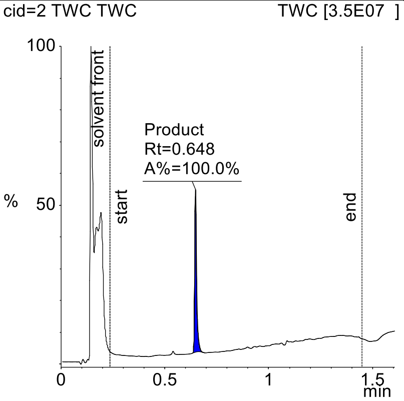	7	364	363.04

**38**	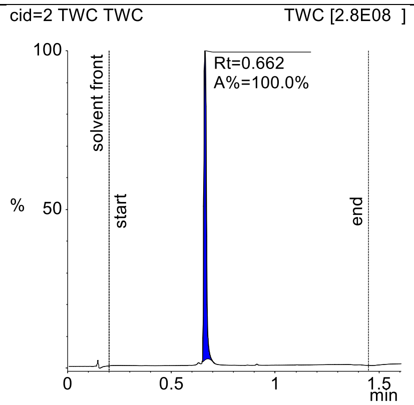	7	368	367.02
**39**	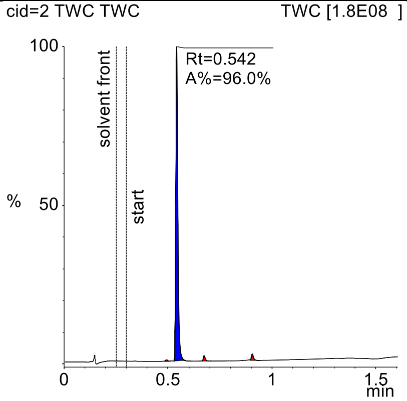	7	353.04	352.32
**40**	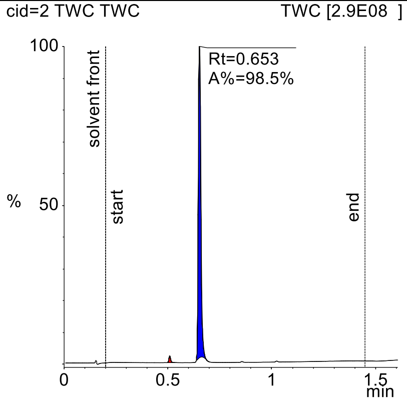	7	366.14	365.1
**41**	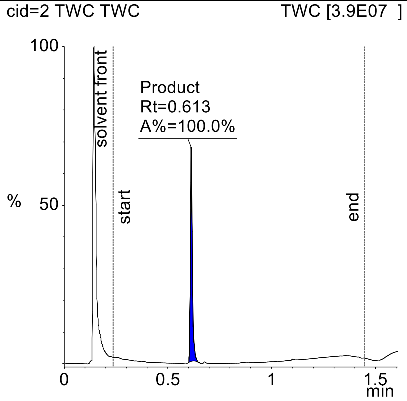	7	367.1	366.06

**42**	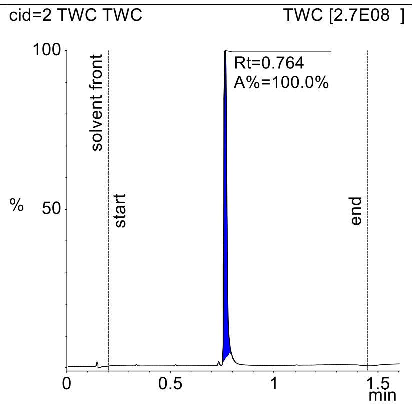	7	420	419.04
**43**	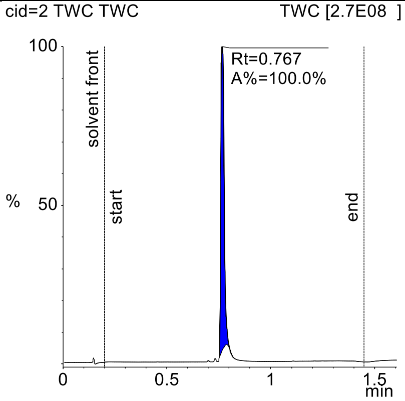	7	392	391.08
**44**	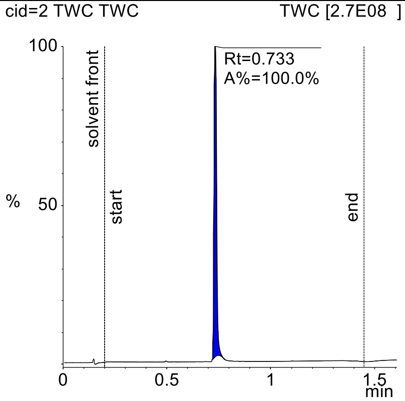	7	380.1	379.08
**45 (BI-3231)**	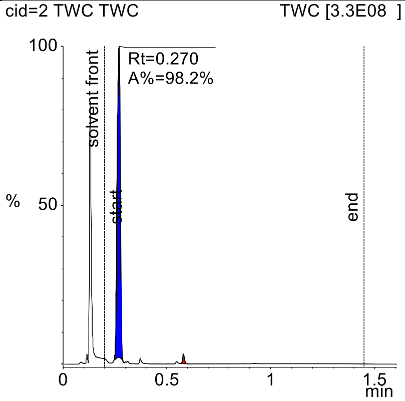	8	381	380.1

**46**	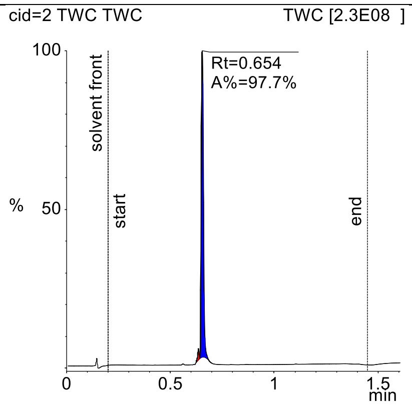	7	381	380.08

7. ^{1}H NMR spectra for key compounds

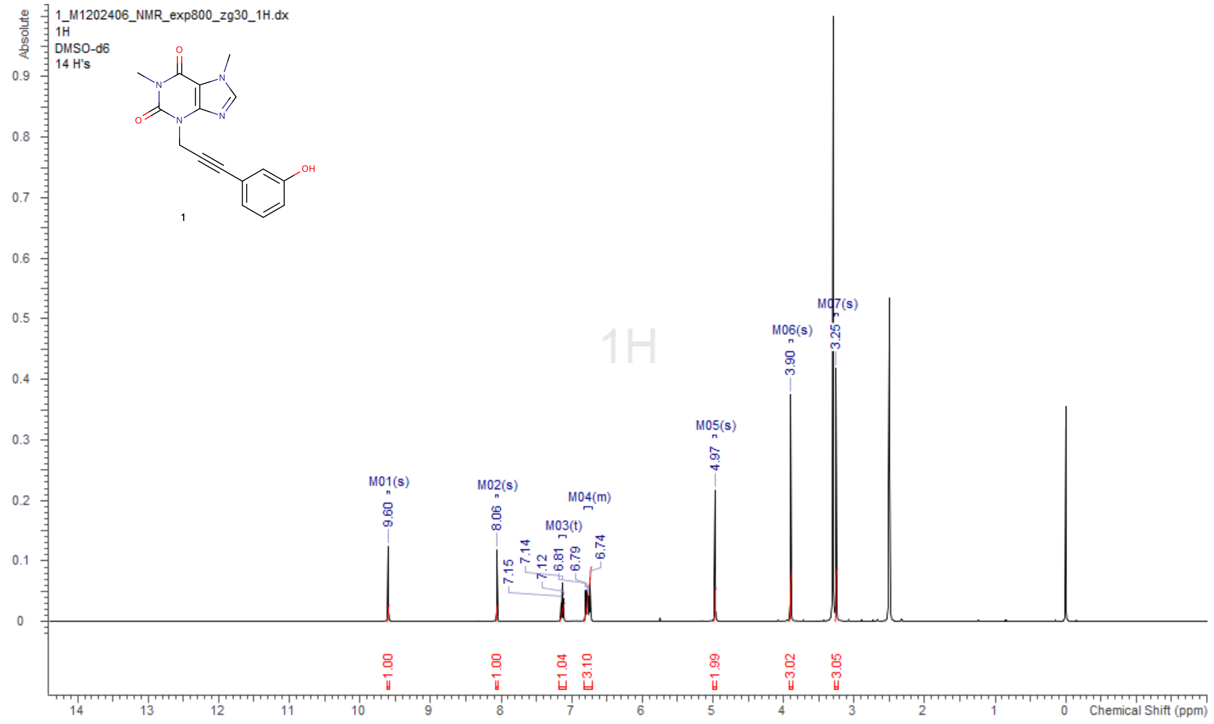

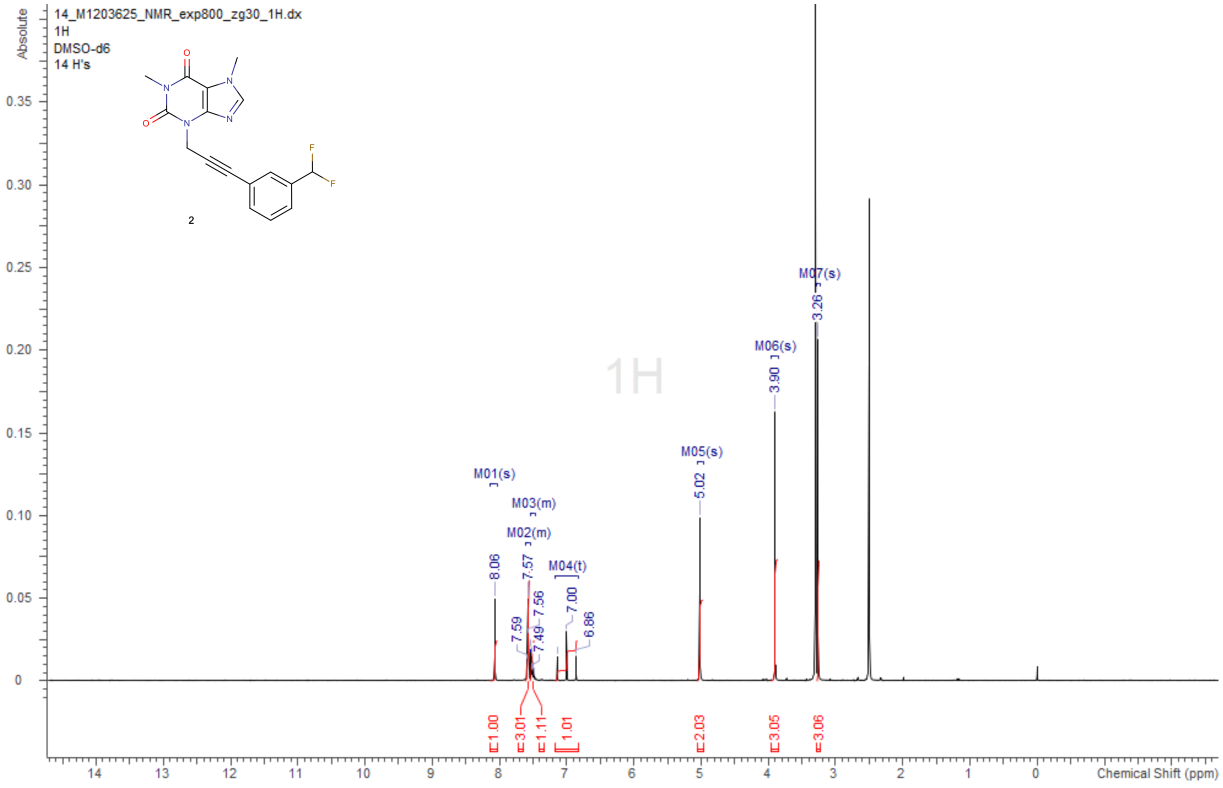

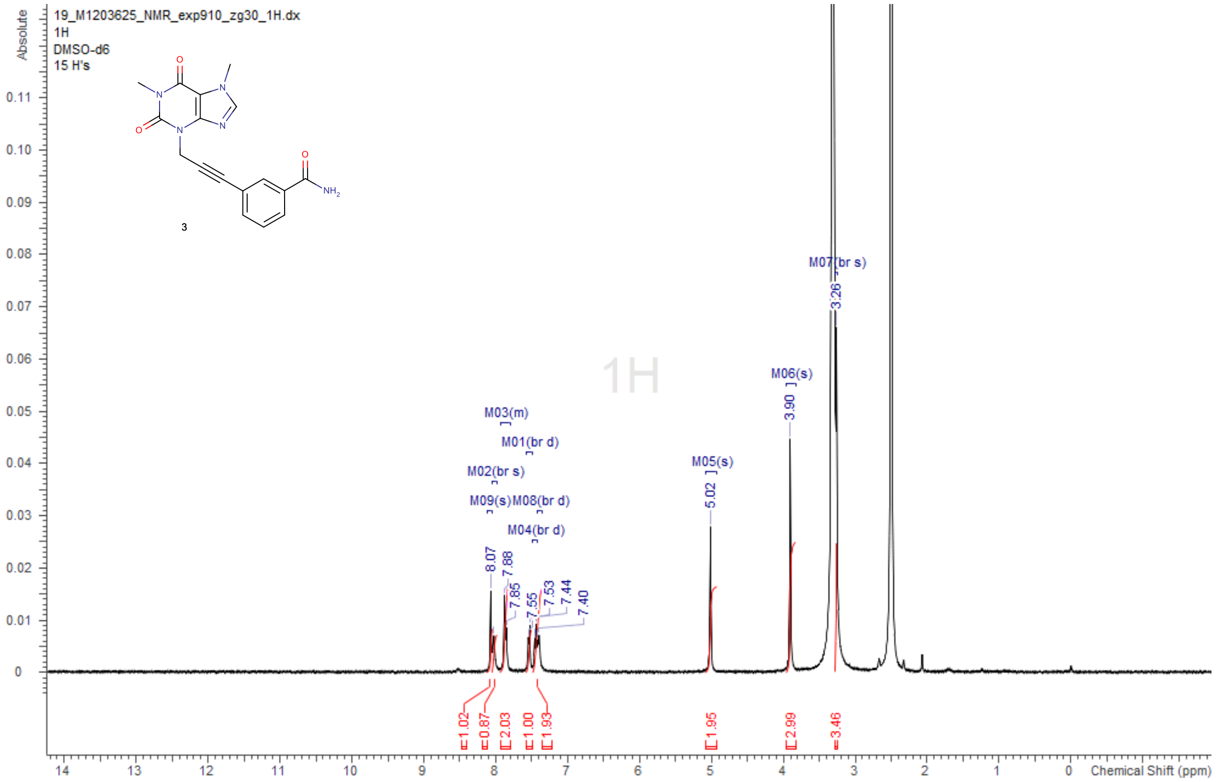
19_M1203625_NMR_exp910_zg30_1H.dx
1H
DMSO-d6
15 H's
3
M03(m)
M01(br d)
M02(br s)
M09(s)
M08(br d)
M04(br d)
M05(s)
M06(s)
M07(br s)
8.07
7.88
7.85
7.55
7.53
7.44
7.40
5.02
3.90
3.26
1.02
0.87
2.03
1.00
1.93
1.95
2.99
3.46
Chemical Shift (ppm)
Absolute

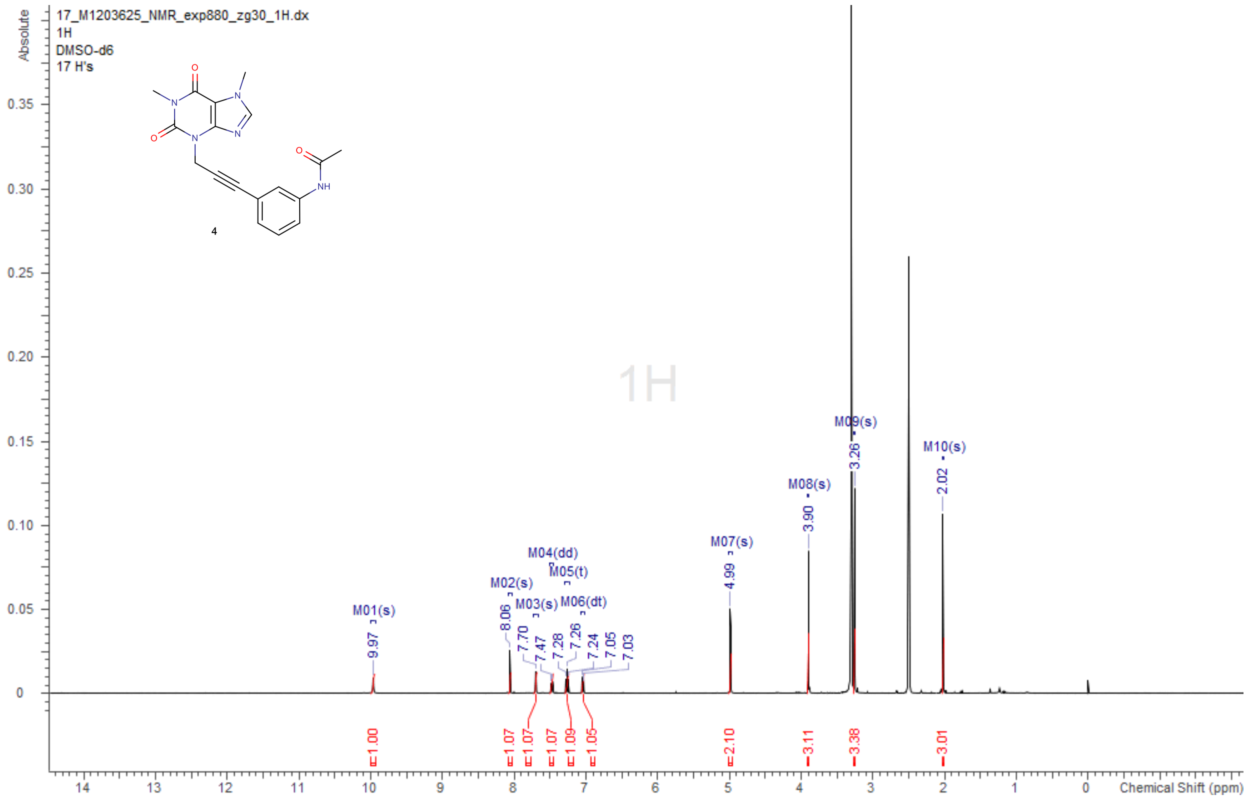
17_M1203625_NMR_exp880_zg30_1H.dx
1H
DMSO-d6
17 H's
4
M01(s)
M02(s)
M03(s)
M04(dd)
M05(t)
M06(dt)
M07(s)
M08(s)
M09(s)
M10(s)
9.97
8.06
7.70
7.47
7.28
7.26
7.24
7.05
7.03
4.99
3.90
3.26
2.02
1.00
1.07
1.07
1.07
1.09
1.05
2.10
3.11
3.38
3.01
Chemical Shift (ppm)
Absolute

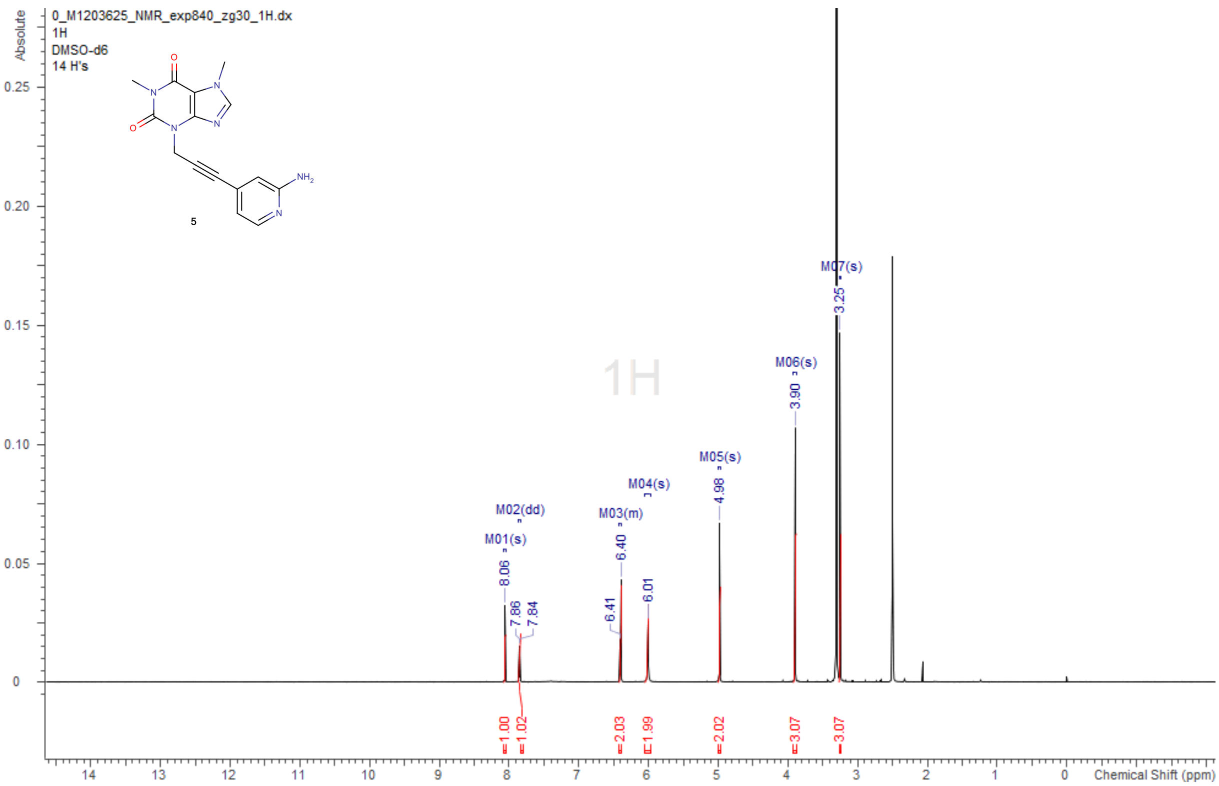

0_M1203625_NMR_exp840_zg30_1H.dx
1H
DMSO-d6
14 H's
5
M01(s)
M02(dd)
M03(m)
M04(s)
M05(s)
M06(s)
M07(s)
8.06
7.86
7.84
6.41
6.40
6.01
4.98
3.90
3.25
1.00
1.02
2.03
1.99
2.02
3.07
3.07
Absolute
Chemical Shift (ppm)

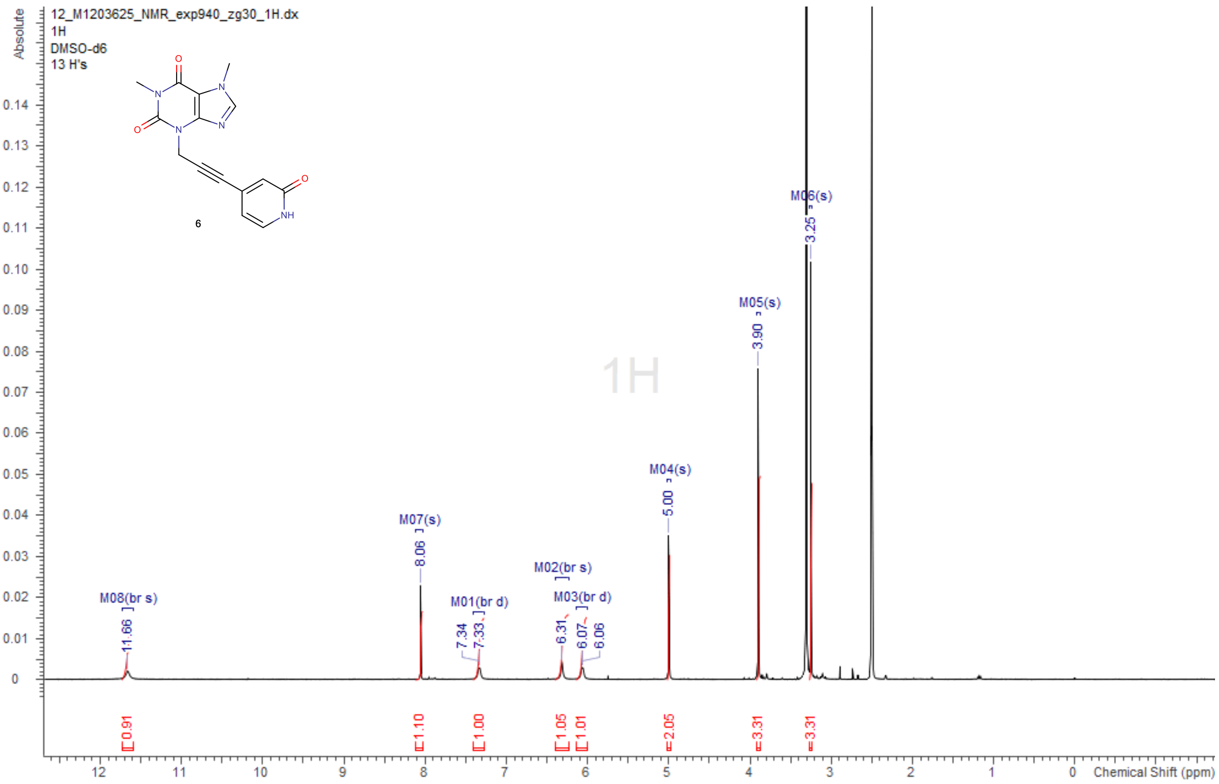

12_M1203625_NMR_exp940_zg30_1H.dx
1H
DMSO-d6
13 H's
6
M08(br s)
M07(s)
M01(br d)
M02(br s)
M03(br d)
M04(s)
M05(s)
M06(s)
11.66
8.06
7.34
7.33
6.31
6.07
6.06
5.00
3.90
3.25
0.91
1.10
1.00
1.05
1.01
2.05
3.31
3.31
Absolute
Chemical Shift (ppm)

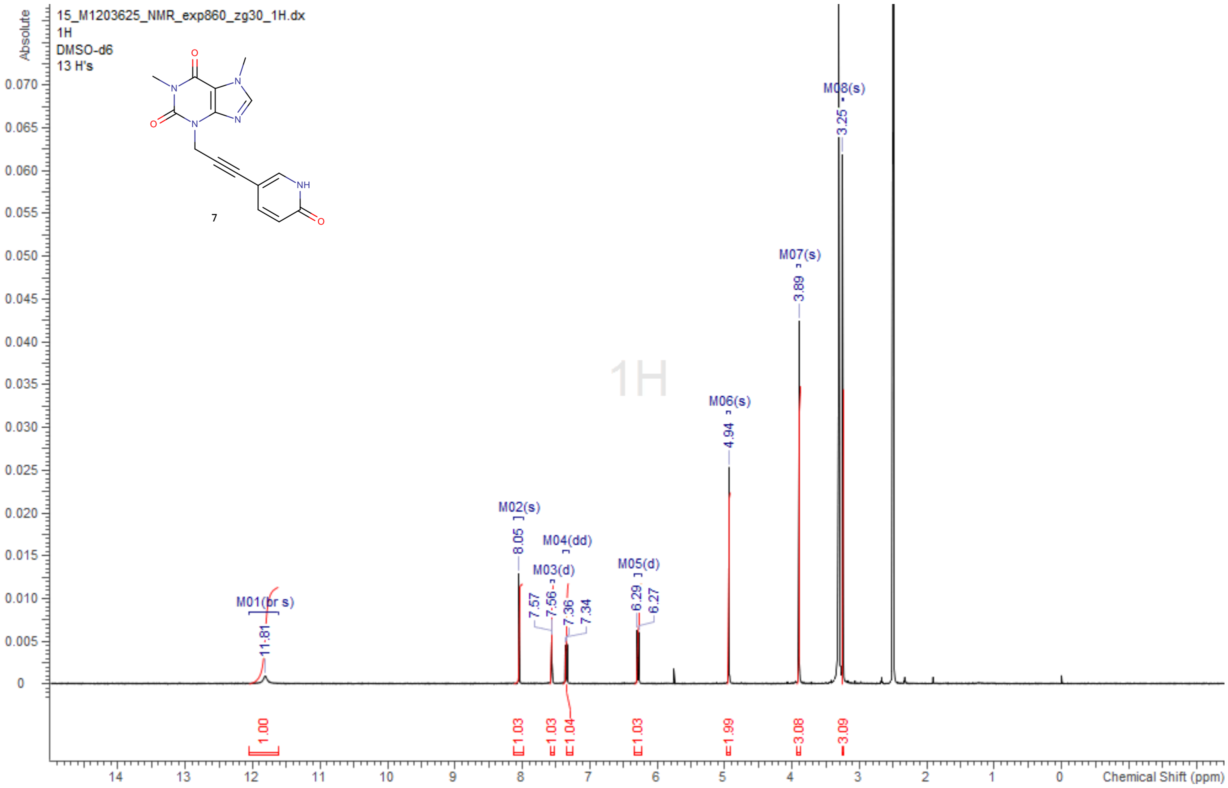

15_M1203625_NMR_exp860_zg30_1H.dx
1H
DMSO-d6
13 H's
7
M01(br s)
11.81
M02(s)
8.05
M03(d)
7.57
7.56
M04(dd)
7.36
7.34
M05(d)
6.29
6.27
M06(s)
4.94
M07(s)
3.89
M08(s)
3.25
1.00
1.03
1.03
1.04
1.03
1.99
3.08
3.09
Absolute
Chemical Shift (ppm)

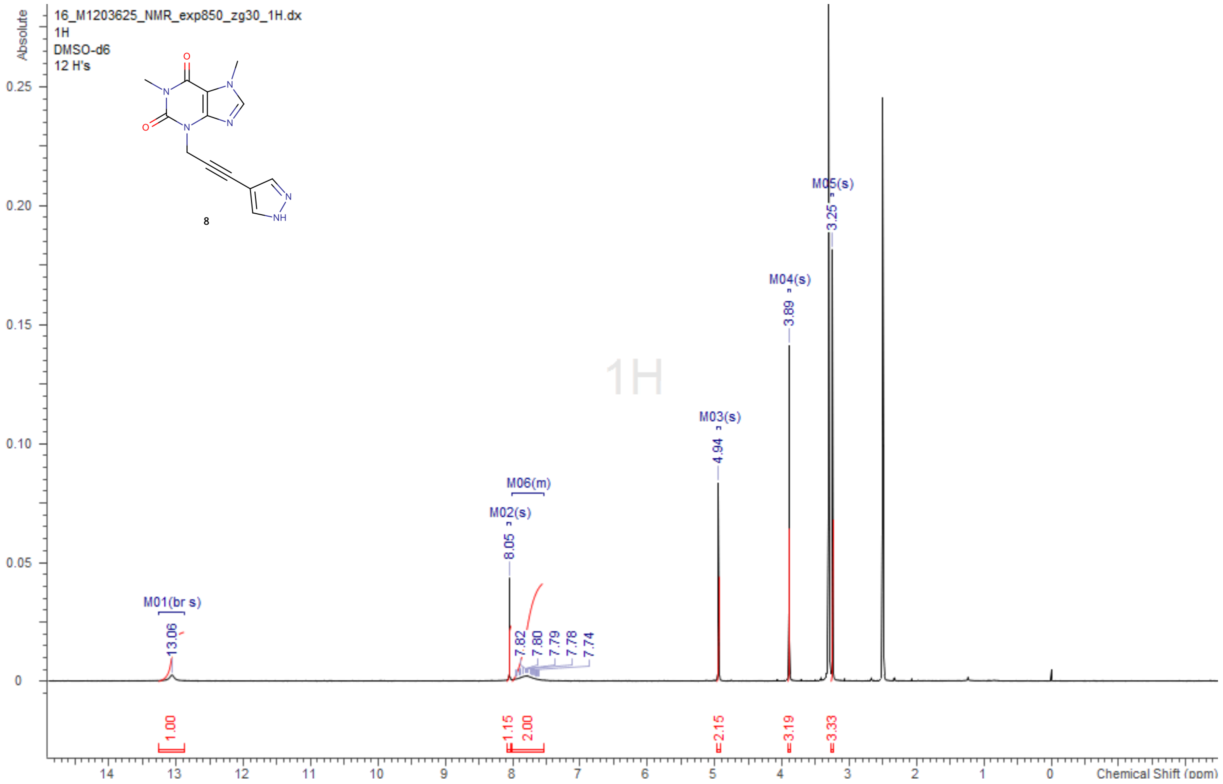

16_M1203625_NMR_exp850_zg30_1H.dx
1H
DMSO-d6
12 H's
8
M01(br s)
13.06
M02(s)
8.05
M06(m)
7.82
7.80
7.79
7.78
7.74
M03(s)
4.94
M04(s)
3.89
M05(s)
3.25
1.00
1.15
2.00
2.15
3.19
3.33
Absolute
Chemical Shift (ppm)

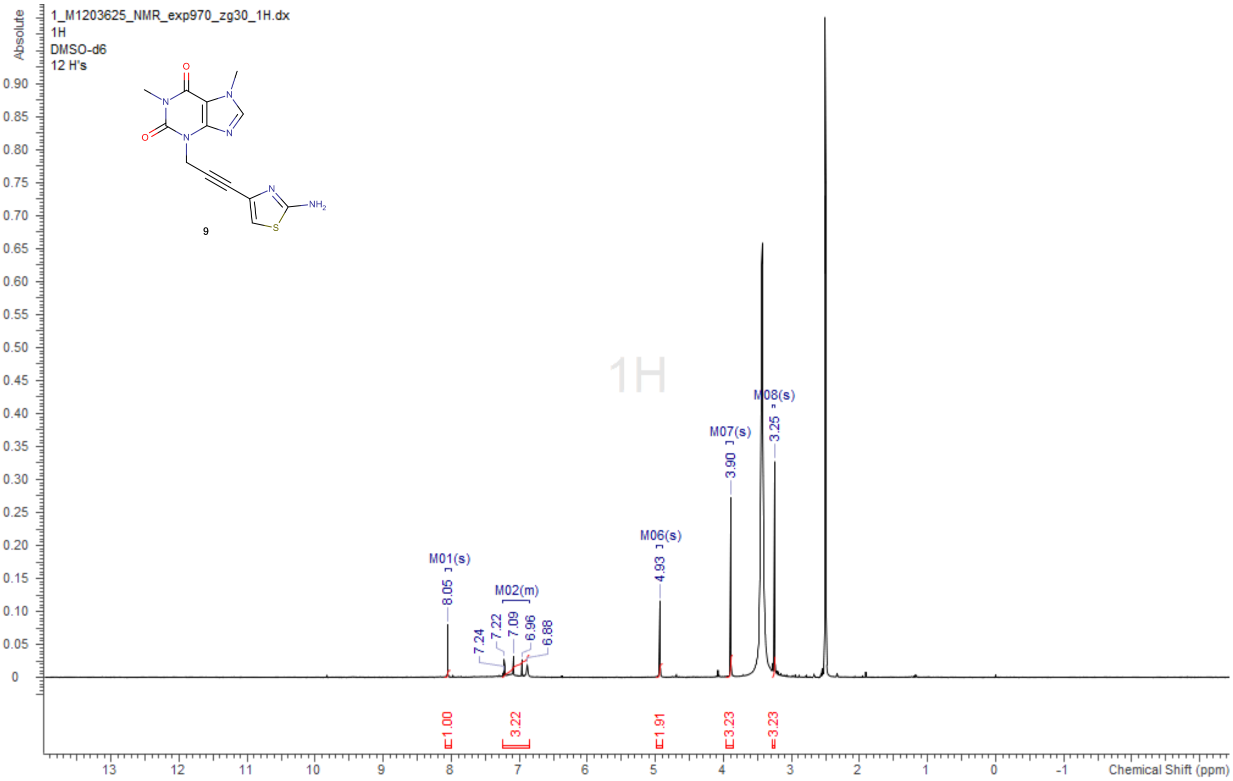
1_M1203625_NMR_exp970_zg30_1H.dx
1H
DMSO-d6
12 H's
9
Absolute
1H
M01(s)
8.05
M02(m)
7.24
7.22
7.09
6.96
6.88
M06(s)
4.93
M07(s)
3.90
M08(s)
3.25
1.00
3.22
1.91
3.23
3.23
Chemical Shift (ppm)

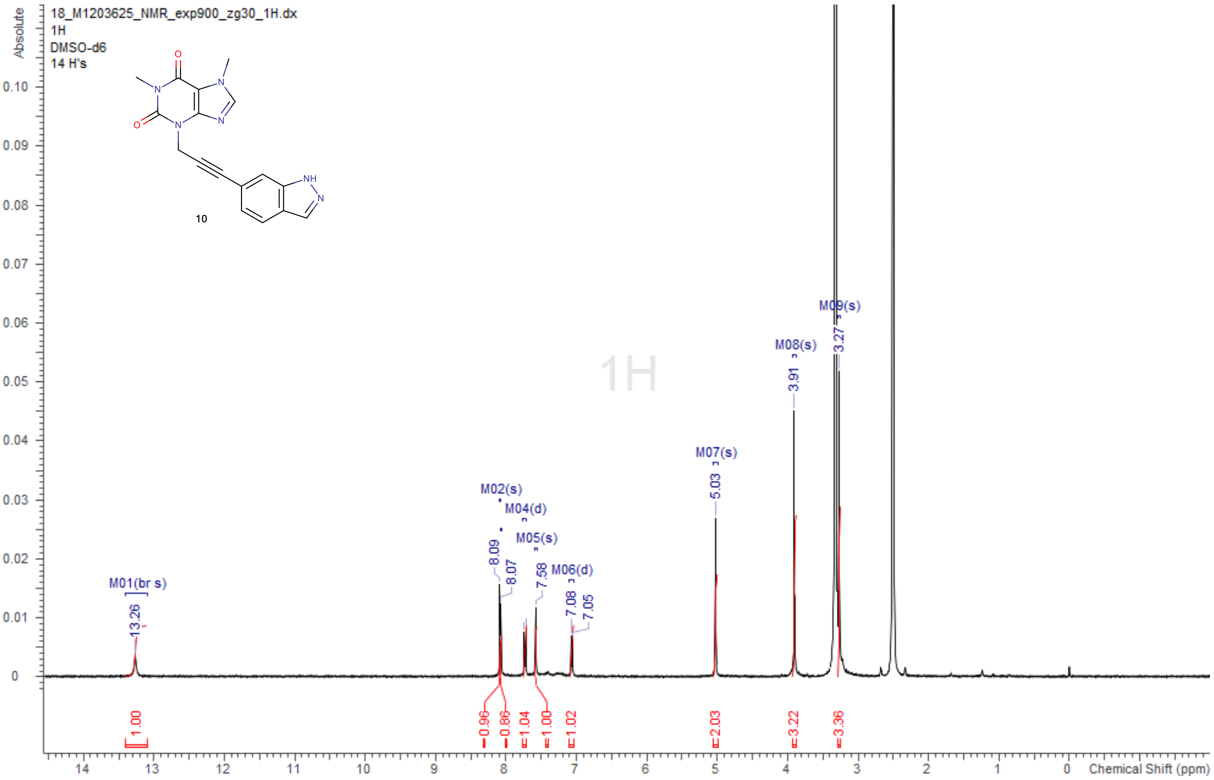
18_M1203625_NMR_exp900_zg30_1H.dx
1H
DMSO-d6
14 H's
10
Absolute
1H
M01(br s)
13.26
M02(s)
8.09
M04(d)
8.07
M05(s)
7.58
M06(d)
7.08
7.05
M07(s)
5.03
M08(s)
3.91
M09(s)
3.27
1.00
0.96
0.86
1.04
1.00
1.02
2.03
3.22
3.36
Chemical Shift (ppm)

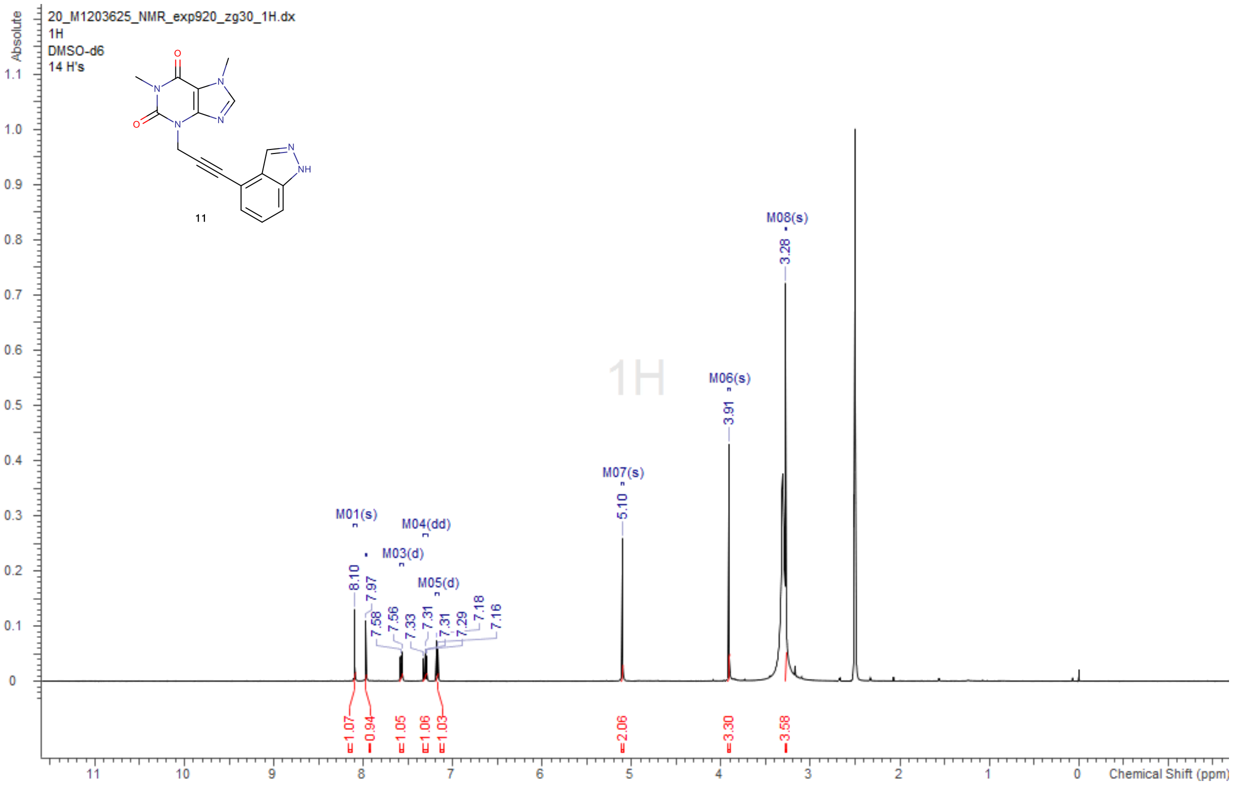
20_M1203625_NMR_exp920_zg30_1H.dx
1H
DMSO-d6
14 H's
11
M01(s)
M03(d)
M04(dd)
M05(d)
M06(s)
M07(s)
M08(s)
1H
Absolute
Chemical Shift (ppm)

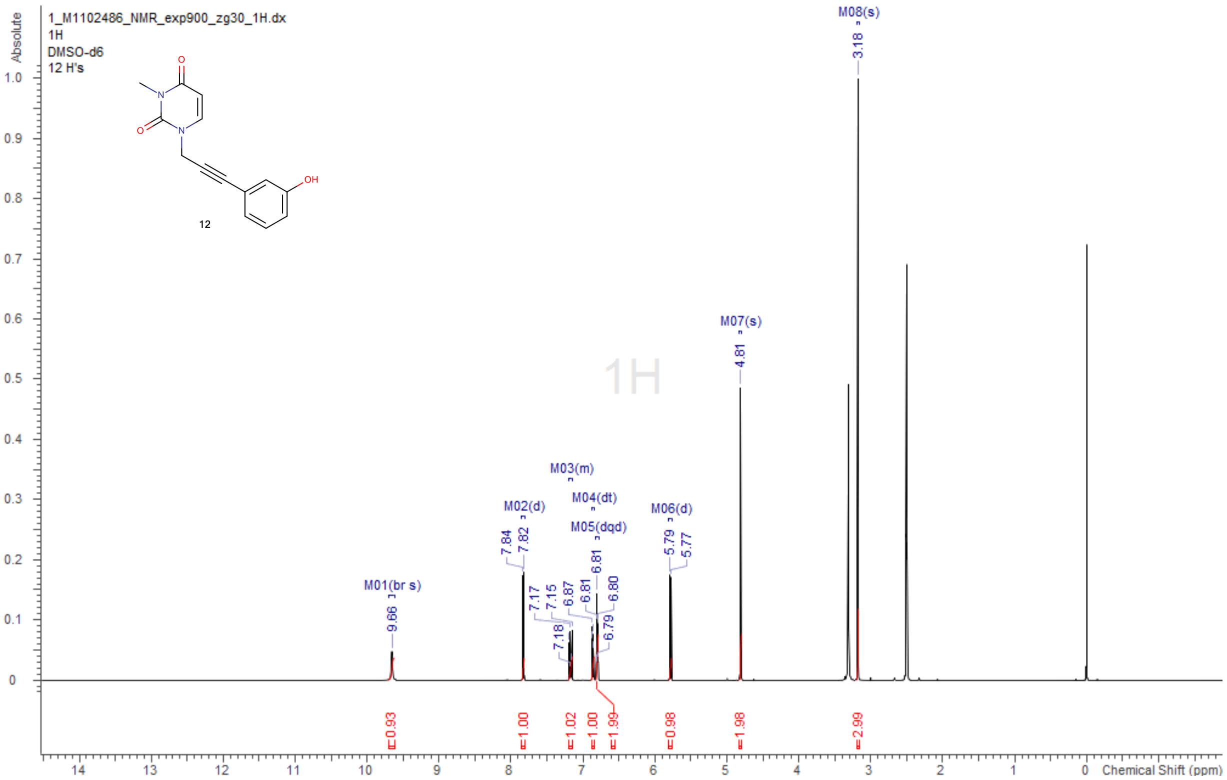
1_M1102486_NMR_exp900_zg30_1H.dx
1H
DMSO-d6
12 H's
12
M01(br s)
M02(d)
M03(m)
M04(dt)
M05(dqd)
M06(d)
M07(s)
M08(s)
1H
Absolute
Chemical Shift (ppm)

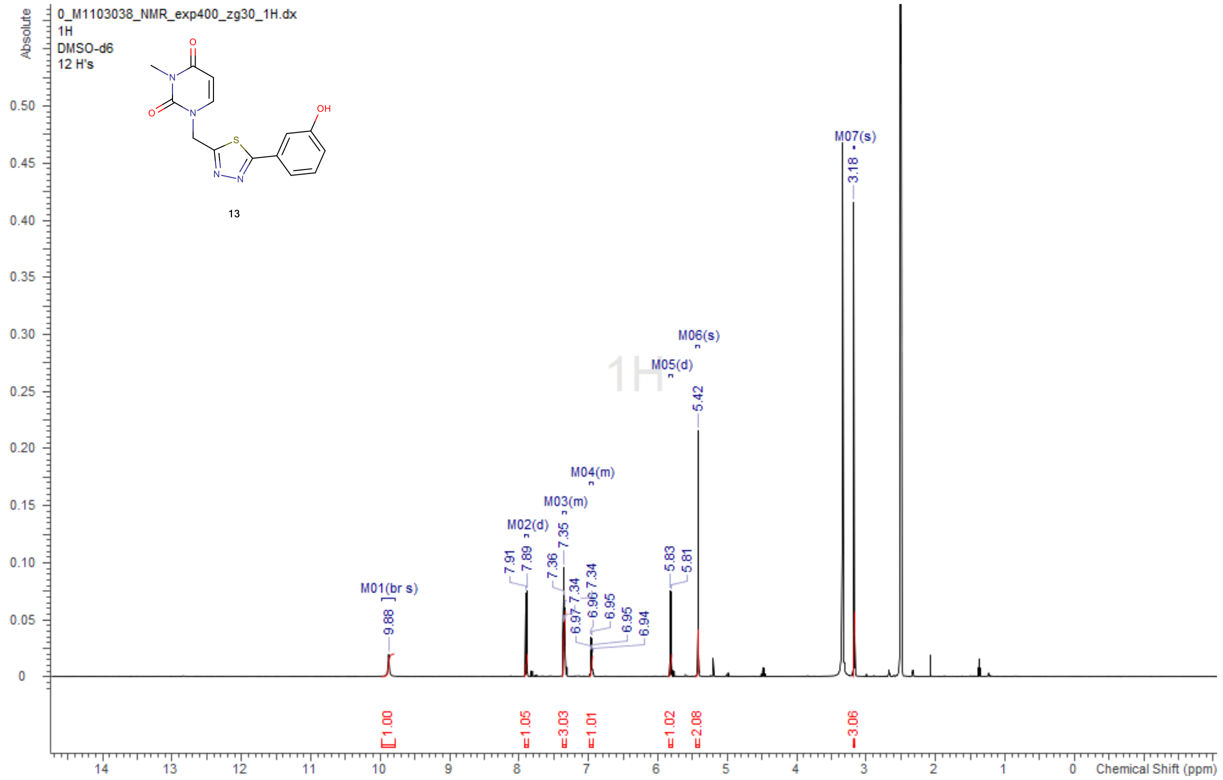

0_M1103038_NMR_exp400_zg30_1H.dx
1H
DMSO-d6
12 H's
13
M01(br s)
9.88
M02(d)
7.91
7.89
M03(m)
7.36
7.35
7.34
M04(m)
6.97
6.96
6.95
6.95
6.94
7.34
M05(d)
5.83
5.81
M06(s)
5.42
M07(s)
3.18
1.00
1.05
3.03
1.01
1.02
2.08
3.06
Absolute
Chemical Shift (ppm)

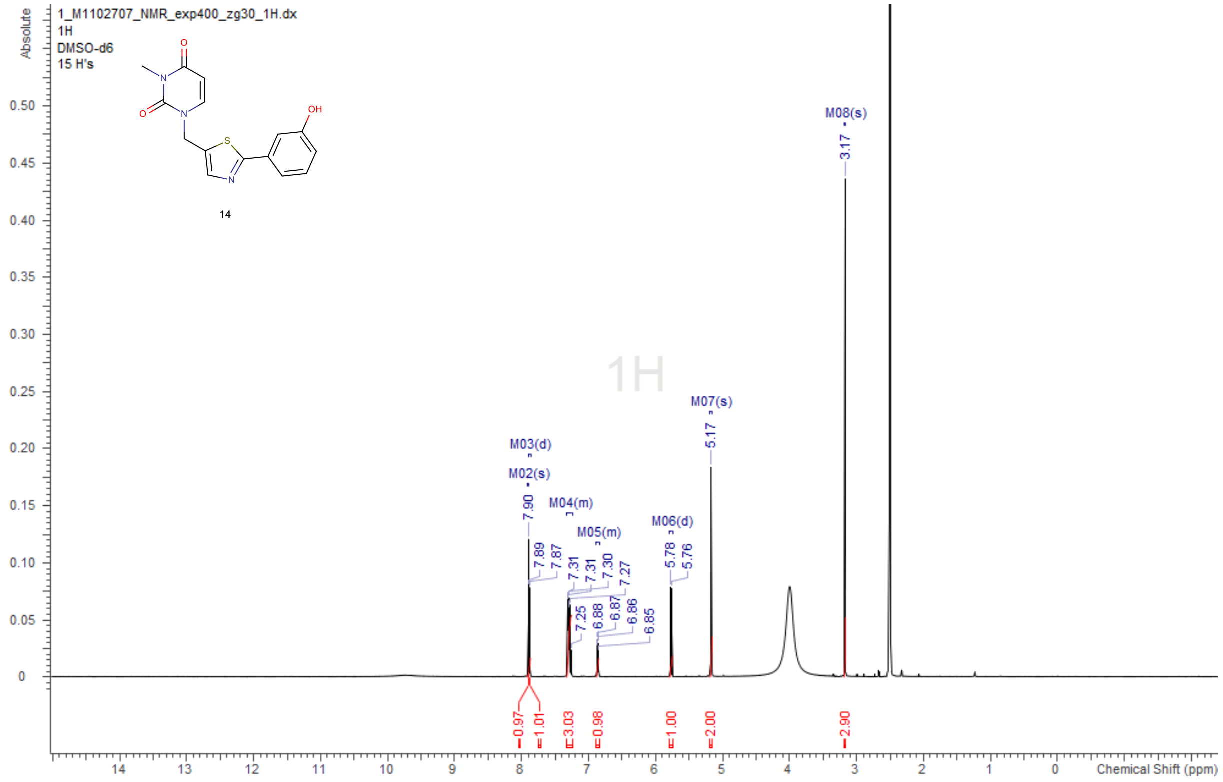

1_M1102707_NMR_exp400_zg30_1H.dx
1H
DMSO-d6
15 H's
14
M03(d)
M02(s)
7.90
7.89
7.87
M04(m)
7.31
7.31
7.30
7.27
7.25
M05(m)
6.88
6.87
6.86
6.85
M06(d)
5.78
5.76
M07(s)
5.17
M08(s)
3.17
0.97
1.01
3.03
0.98
1.00
2.00
2.90
Absolute
Chemical Shift (ppm)

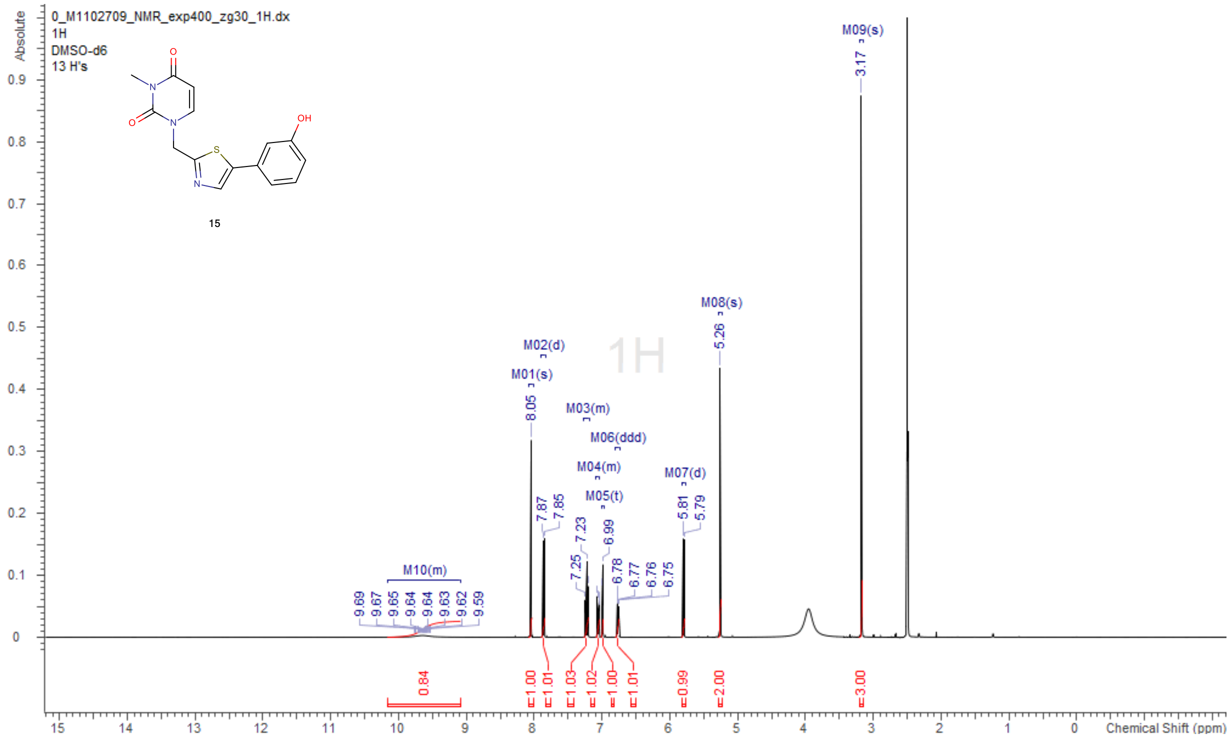
0_M1102709_NMR_exp400_zg30_1H.dx
1H
DMSO-d6
13 H's
15
M01(s)
M02(d)
M03(m)
M04(m)
M05(t)
M06(ddd)
M07(d)
M08(s)
M09(s)
M10(m)
Chemical Shift (ppm)
Absolute

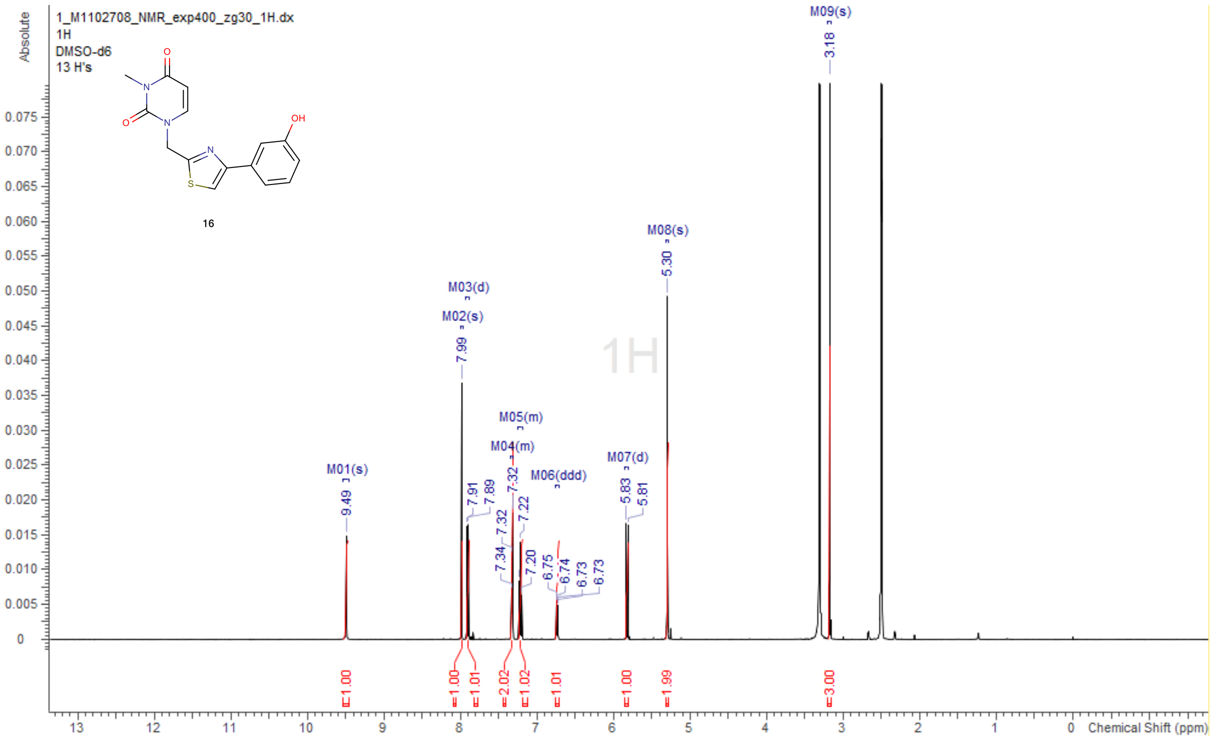
1_M1102708_NMR_exp400_zg30_1H.dx
1H
DMSO-d6
13 H's
16
M01(s)
M02(s)
M03(d)
M04(m)
M05(m)
M06(ddd)
M07(d)
M08(s)
M09(s)
Chemical Shift (ppm)
Absolute

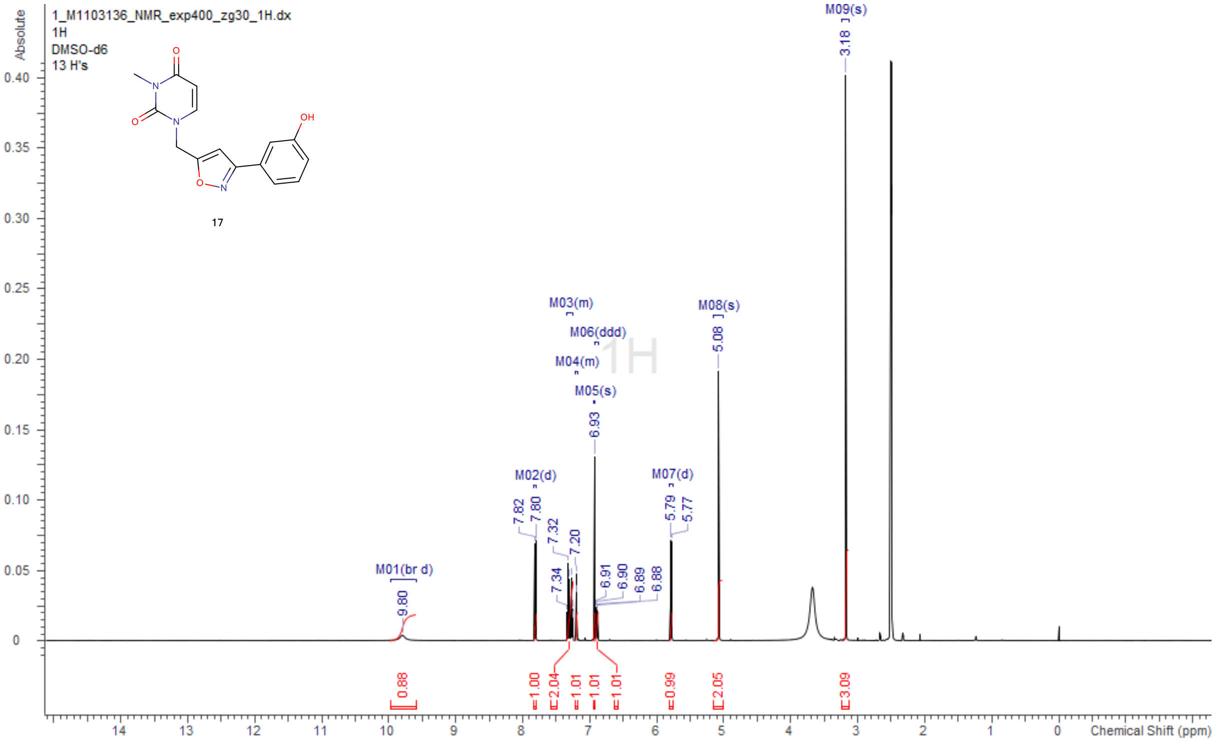
1_M1103136_NMR_exp400_zg30_1H.dx
1H
DMSO-d6
13 H's
Absolute
M09(s)
3.18
M08(s)
5.08
M03(m)
M06(ddd)
M04(m)
M05(s)
6.93
M02(d)
7.82
7.80
7.32
7.20
7.34
6.91
6.90
6.89
6.88
M07(d)
5.79
5.77
M01(br d)
9.80
0.88
1.00
2.04
1.01
1.01
1.01
0.99
2.05
3.09
Chemical Shift (ppm)

17

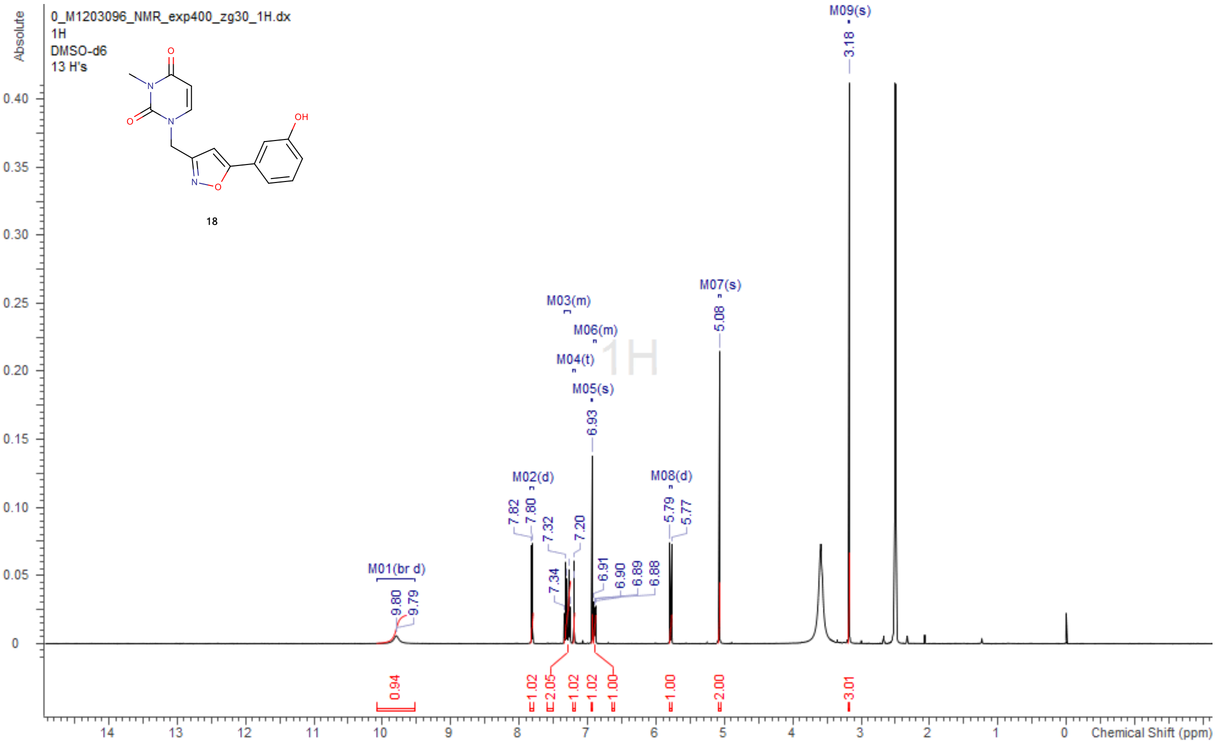
0_M1203096_NMR_exp400_zg30_1H.dx
1H
DMSO-d6
13 H's
Absolute
M09(s)
3.18
M07(s)
5.08
M03(m)
M06(m)
M04(t)
M05(s)
6.93
M02(d)
7.82
7.80
7.32
7.20
7.34
6.91
6.90
6.89
6.88
M08(d)
5.79
5.77
M01(br d)
9.80
9.79
0.94
1.02
2.05
1.02
1.02
1.00
1.00
2.00
3.01
Chemical Shift (ppm)

18

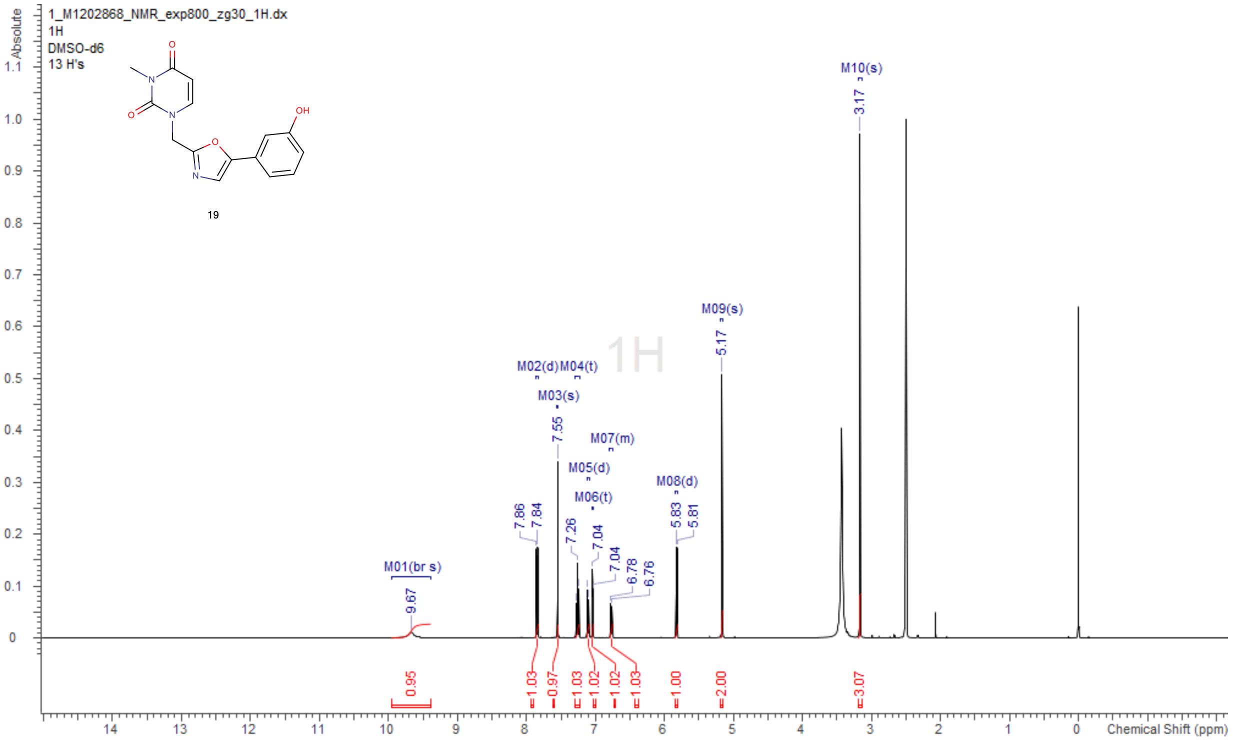
1_M1202868_NMR_exp800_zg30_1H.dx
1H
DMSO-d6
13 H's
19
M01(br s)
9.67
M02(d)
7.86
7.84
M03(s)
7.55
M04(t)
7.26
M05(d)
7.04
M06(t)
7.04
M07(m)
6.78
6.76
M08(d)
5.83
5.81
M09(s)
5.17
M10(s)
3.17
0.95
1.03
0.97
1.03
1.02
1.02
1.03
1.00
2.00
3.07
Absolute
Chemical Shift (ppm)

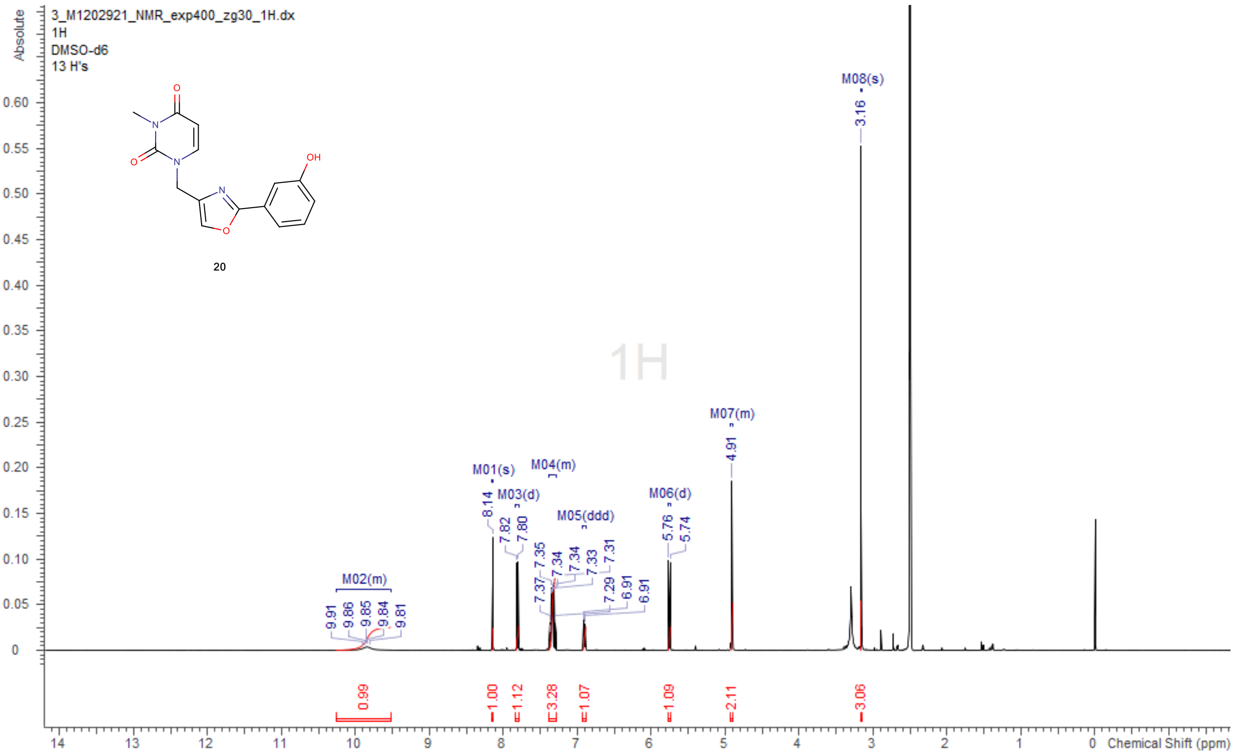
3_M1202921_NMR_exp400_zg30_1H.dx
1H
DMSO-d6
13 H's
20
M01(s)
8.14
M02(m)
9.91
9.86
9.85
9.84
9.81
M03(d)
7.82
7.80
M04(m)
7.37
7.35
7.34
7.34
7.33
7.31
M05(ddd)
7.29
6.91
6.91
M06(d)
5.76
5.74
M07(m)
4.91
M08(s)
3.16
0.99
1.00
1.12
3.28
1.07
1.09
2.11
3.06
Absolute
Chemical Shift (ppm)

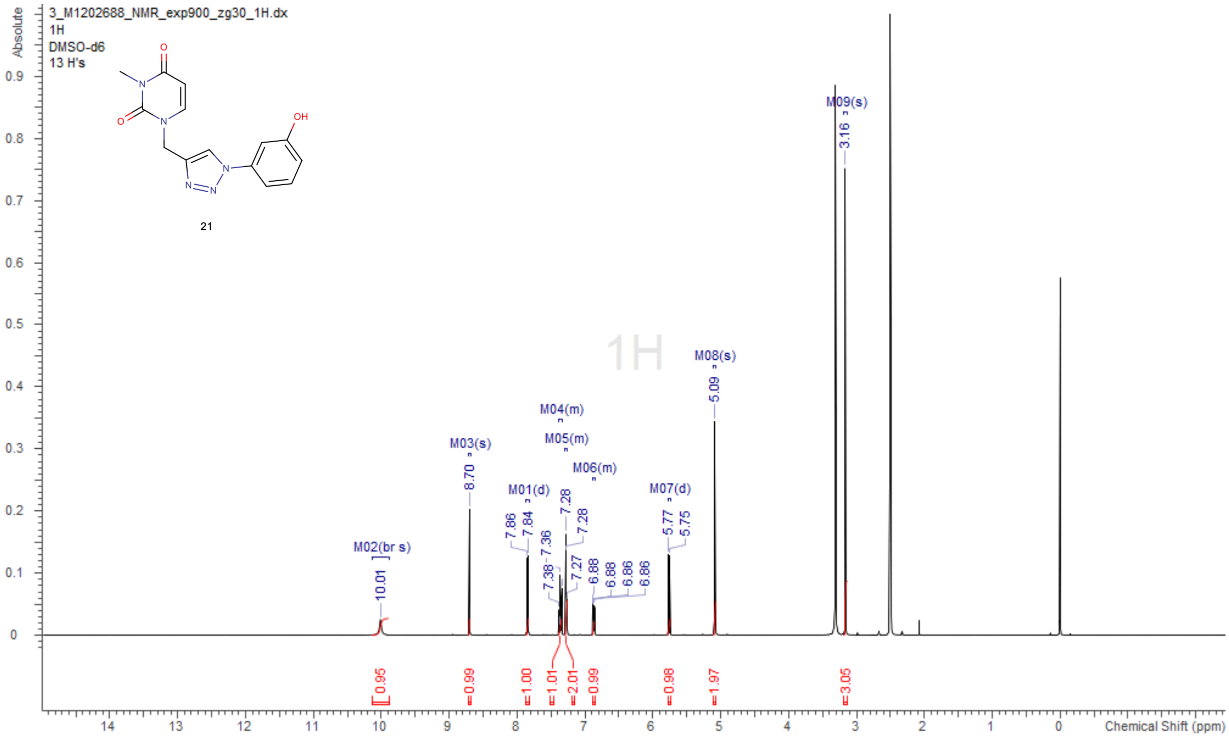
3_M1202688_NMR_exp900_zg30_1H.dx
1H
DMSO-d6
13 H's
21
M02(br s)
M03(s)
M01(d)
M04(m)
M05(m)
M06(m)
M07(d)
M08(s)
M09(s)
Absolute
Chemical Shift (ppm)

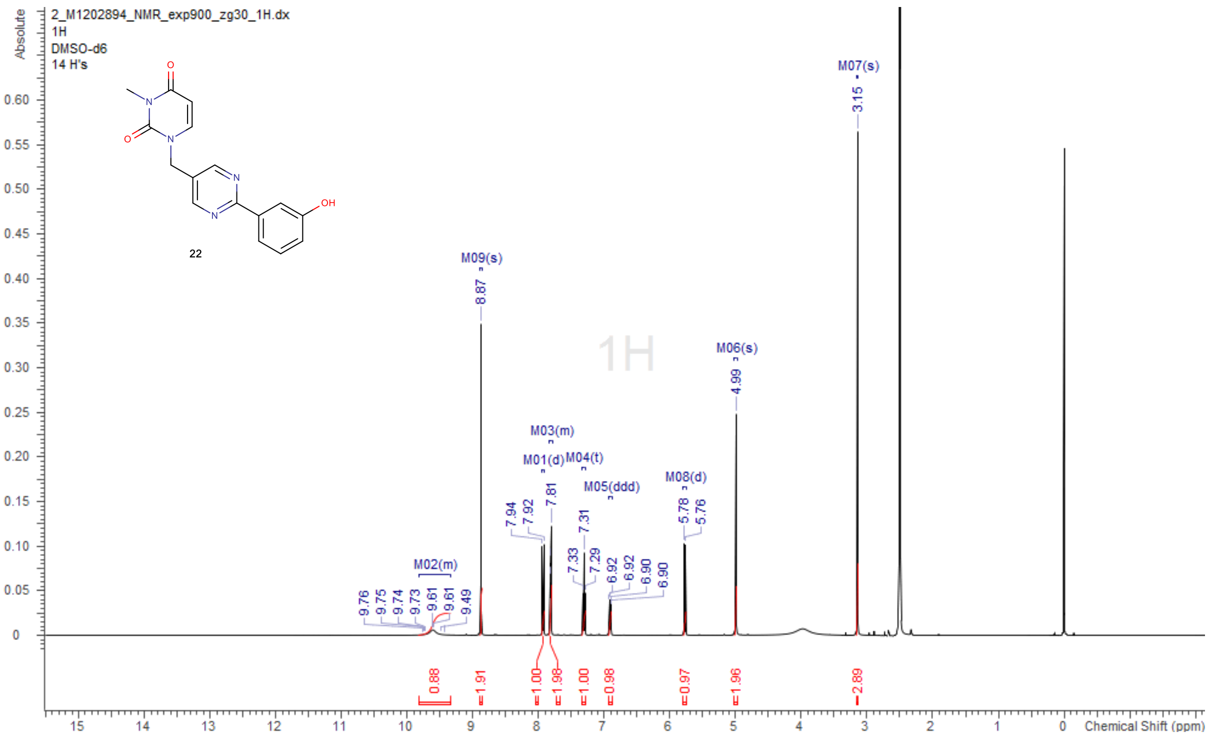
2_M1202894_NMR_exp900_zg30_1H.dx
1H
DMSO-d6
14 H's
22
M02(m)
M09(s)
M01(d)
M03(m)
M04(t)
M05(ddd)
M08(d)
M06(s)
M07(s)
Absolute
Chemical Shift (ppm)

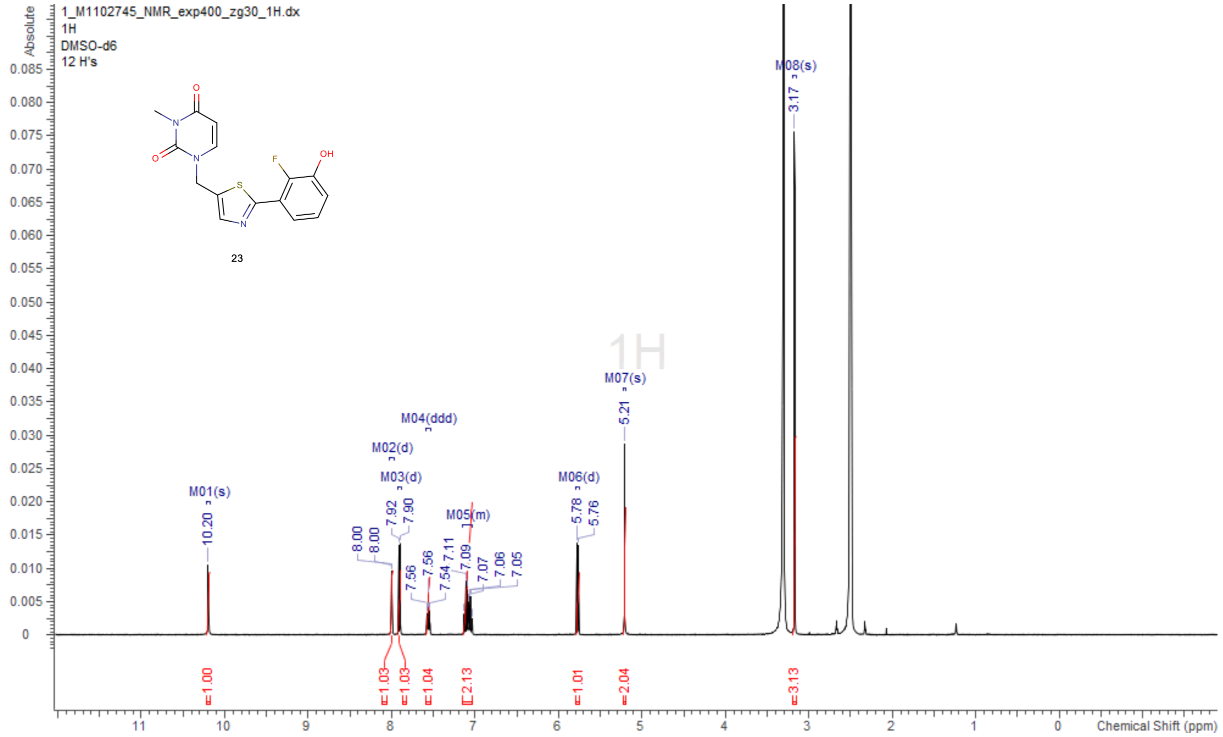
1_M1102745_NMR_exp400_zg30_1H.dx
1H
DMSO-d6
12 H's
23
M01(s)
10.20
M02(d)
M03(d)
M04(ddd)
M05(m)
M06(d)
M07(s)
M08(s)
8.00
8.00
7.92
7.90
7.56
7.56
7.54
7.11
7.09
7.07
7.06
7.05
5.78
5.76
5.21
3.17
1.00
1.03
1.03
1.04
2.13
1.01
2.04
3.13
Absolute
Chemical Shift (ppm)

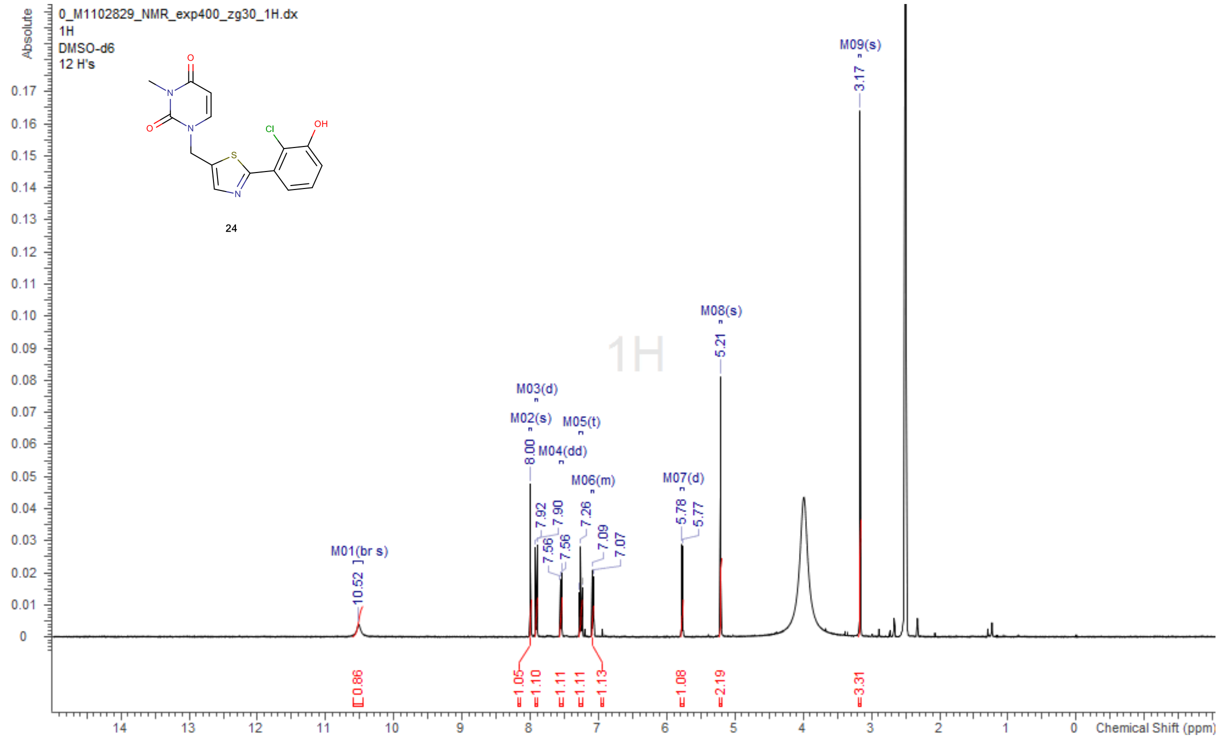
0_M1102829_NMR_exp400_zg30_1H.dx
1H
DMSO-d6
12 H's
24
M01(br s)
10.52
M02(s)
M03(d)
M04(dd)
M05(t)
M06(m)
M07(d)
M08(s)
M09(s)
8.00
7.92
7.90
7.56
7.56
7.26
7.09
7.07
5.78
5.77
5.21
3.17
0.86
1.05
1.10
1.11
1.11
1.13
1.08
2.19
3.31
Absolute
Chemical Shift (ppm)

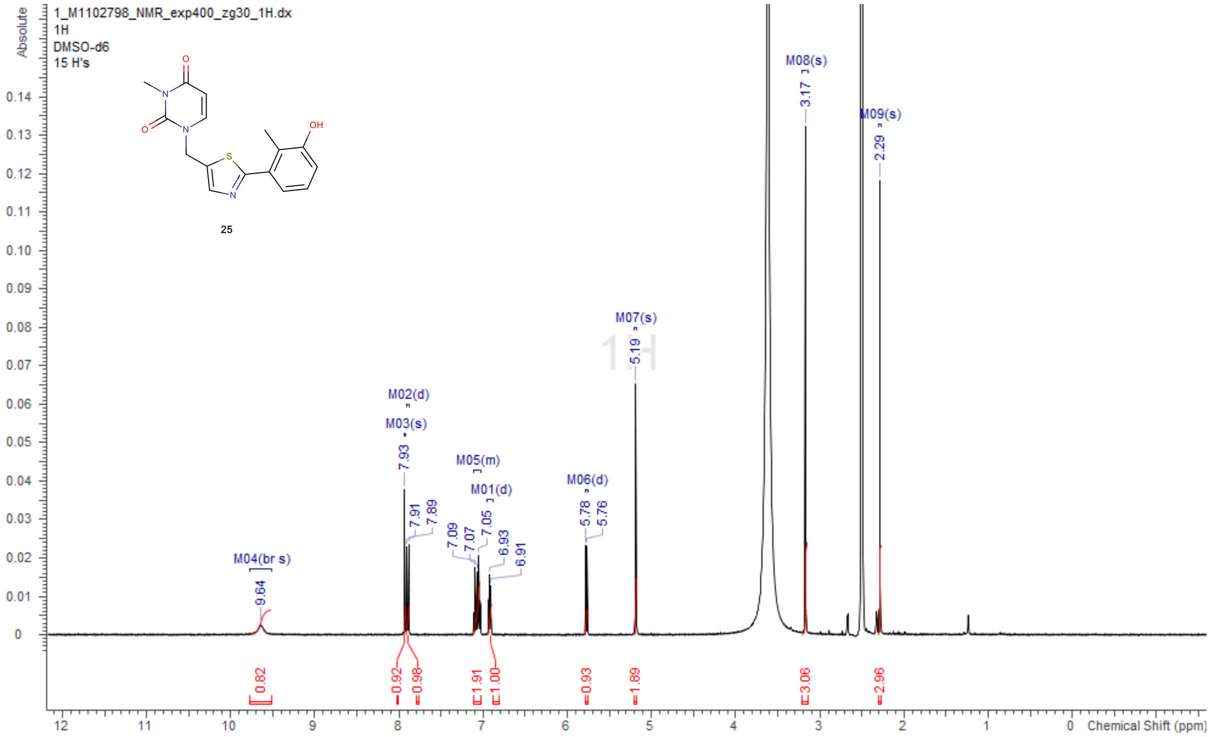
1_M1102798_NMR_exp400_zg30_1H.dx
1H
DMSO-d6
15 H's
25
M04(br s)
9.64
M02(d)
M03(s)
7.93
7.91
7.89
M05(m)
M01(d)
7.09
7.07
7.05
6.93
6.91
M06(d)
5.78
5.76
M07(s)
5.19
M08(s)
3.17
M09(s)
2.29
0.82
0.92
0.98
1.91
1.00
0.93
1.89
3.06
2.96
Absolute
Chemical Shift (ppm)

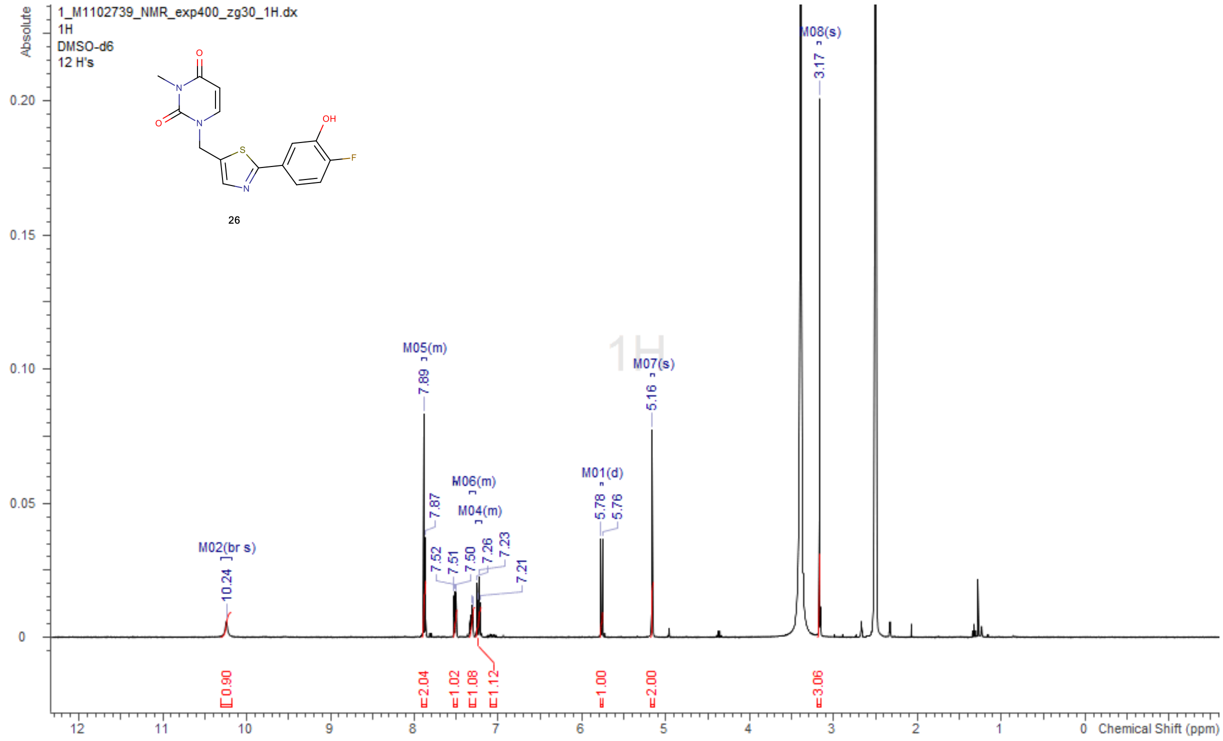
1_M1102739_NMR_exp400_zg30_1H.dx
1H
DMSO-d6
12 H's
26
M02(br s)
10.24
M05(m)
7.89
7.87
M06(m)
M04(m)
7.52
7.51
7.50
7.26
7.23
7.21
M01(d)
5.78
5.76
M07(s)
5.16
M08(s)
3.17
0.90
2.04
1.02
1.08
1.12
1.00
2.00
3.06
Absolute
Chemical Shift (ppm)

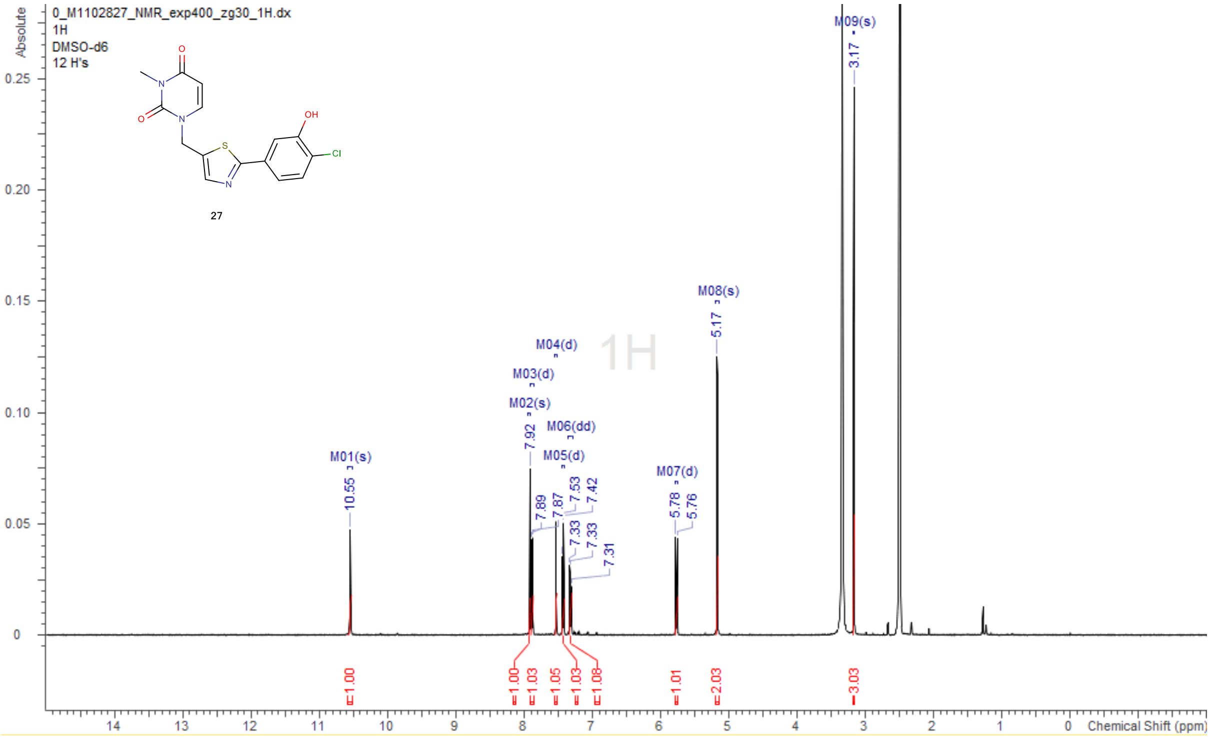
0_M1102827_NMR_exp400_zg30_1H.dx
1H
DMSO-d6
12 H's
Absolute
27
1H
M01(s) 10.55
M02(s) 7.92
M03(d) 7.89 7.87
M04(d) 7.53
M05(d) 7.42
M06(dd) 7.33 7.33 7.31
M07(d) 5.78 5.76
M08(s) 5.17
M09(s) 3.17
1.00 1.00 1.03 1.05 1.03 1.08 1.01 2.03 3.03
Chemical Shift (ppm)

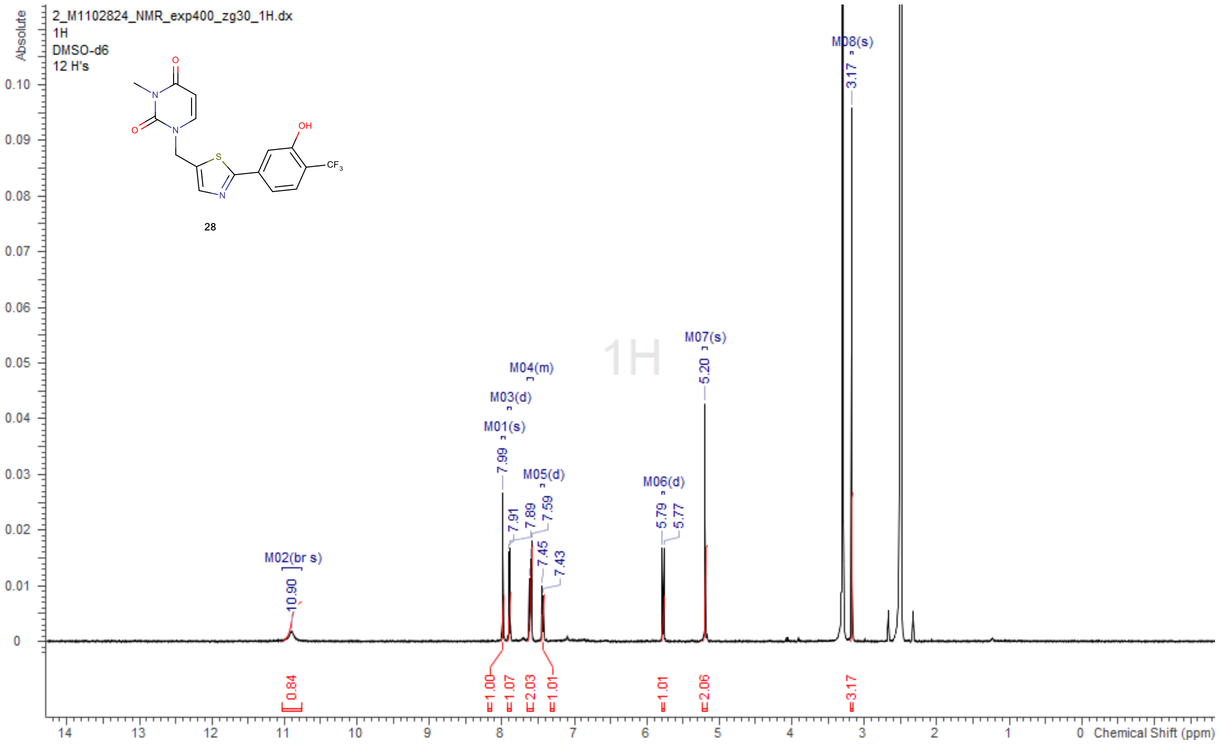
2_M1102824_NMR_exp400_zg30_1H.dx
1H
DMSO-d6
12 H's
Absolute
28
1H
M02(br s) 10.90
M01(s) 7.99
M03(d) 7.91 7.89
M04(m) 7.59
M05(d) 7.45 7.43
M06(d) 5.79 5.77
M07(s) 5.20
M08(s) 3.17
0.84 1.00 1.07 2.03 1.01 1.01 2.06 3.17
Chemical Shift (ppm)

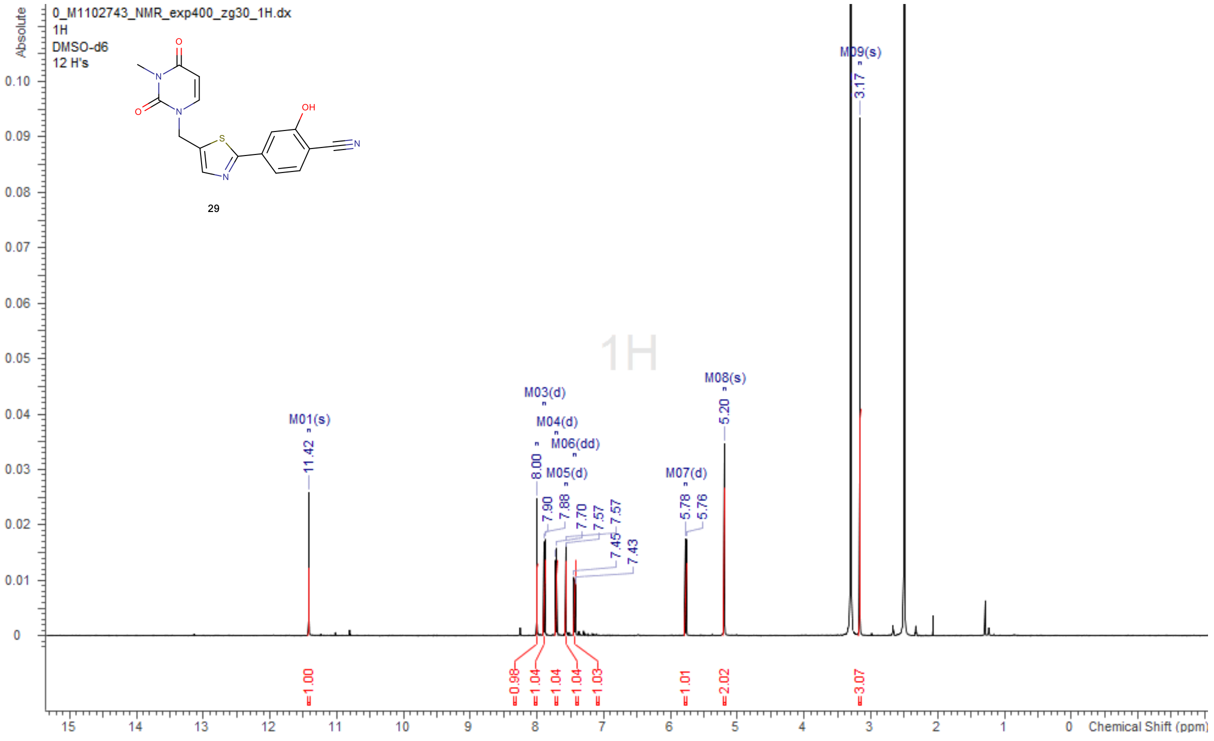
0_M1102743_NMR_exp400_zg30_1H.dx
1H
DMSO-d6
12 H's
29
M01(s)
11.42
M03(d)
M04(d)
M06(dd)
M05(d)
8.00
7.90
7.88
7.70
7.57
7.57
7.45
7.43
M07(d)
5.78
5.76
M08(s)
5.20
M09(s)
3.17
1.00
0.98
1.04
1.04
1.04
1.03
1.01
2.02
3.07
Absolute
Chemical Shift (ppm)

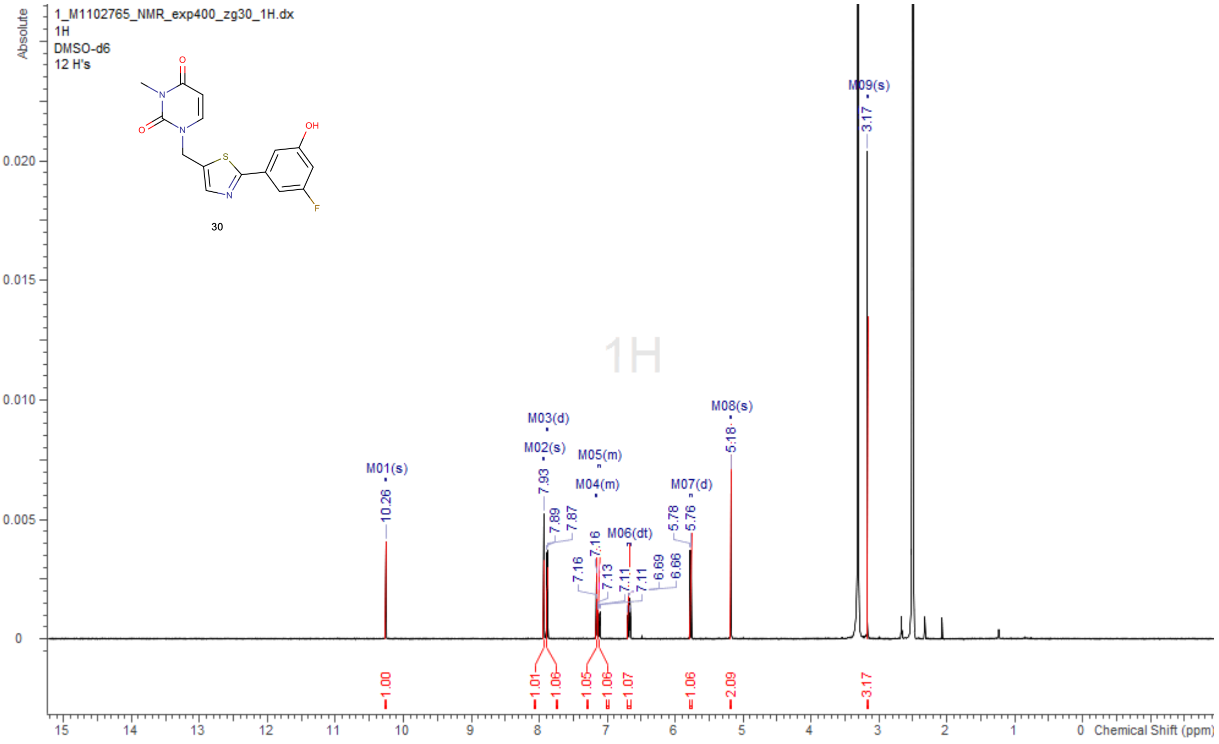
1_M1102765_NMR_exp400_zg30_1H.dx
1H
DMSO-d6
12 H's
30
M01(s)
10.26
M03(d)
M02(s)
M05(m)
M04(m)
M06(dt)
7.93
7.89
7.87
7.16
7.16
7.13
7.11
7.11
6.69
6.66
M07(d)
5.78
5.76
M08(s)
5.18
M09(s)
3.17
1.00
1.01
1.06
1.05
1.06
1.07
1.06
2.09
3.17
Absolute
Chemical Shift (ppm)

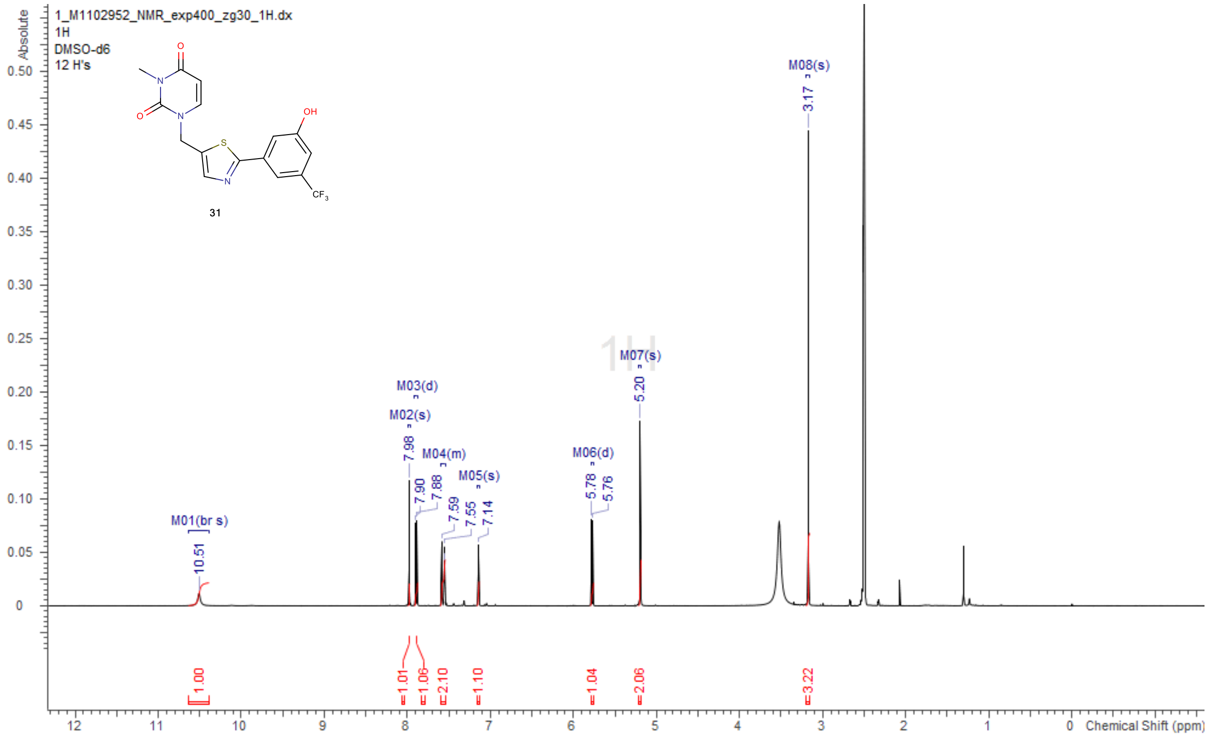
1_M1102952_NMR_exp400_zg30_1H.dx
1H
DMSO-d6
12 H's
31
M01(br s)
10.51
M02(s)
7.98
M03(d)
7.90
7.88
M04(m)
7.59
7.55
M05(s)
7.14
M06(d)
5.78
5.76
M07(s)
5.20
M08(s)
3.17
1.00
1.01
1.06
2.10
1.10
1.04
2.06
3.22
Absolute
Chemical Shift (ppm)

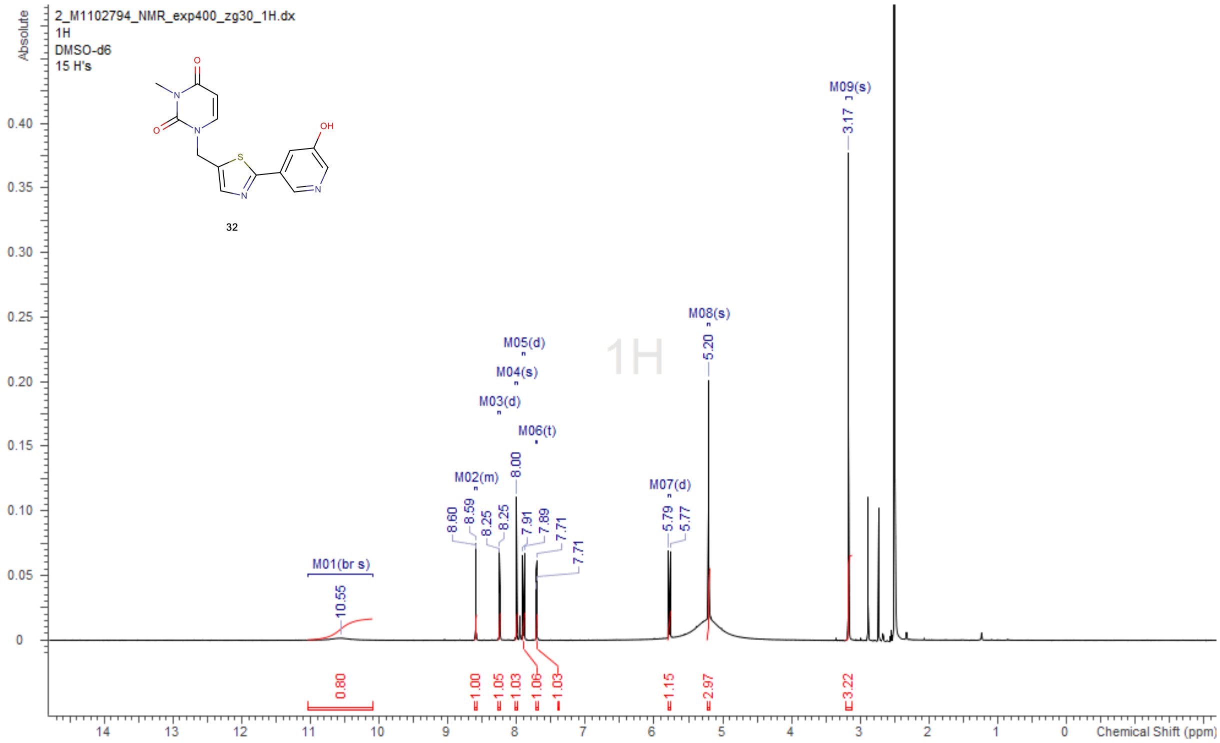
2_M1102794_NMR_exp400_zg30_1H.dx
1H
DMSO-d6
15 H's
32
M01(br s)
10.55
M02(m)
8.60
8.59
M03(d)
8.25
8.25
M04(s)
8.00
M05(d)
7.91
7.89
M06(t)
7.71
7.71
M07(d)
5.79
5.77
M08(s)
5.20
M09(s)
3.17
0.80
1.00
1.05
1.03
1.06
1.03
1.15
2.97
3.22
Absolute
Chemical Shift (ppm)

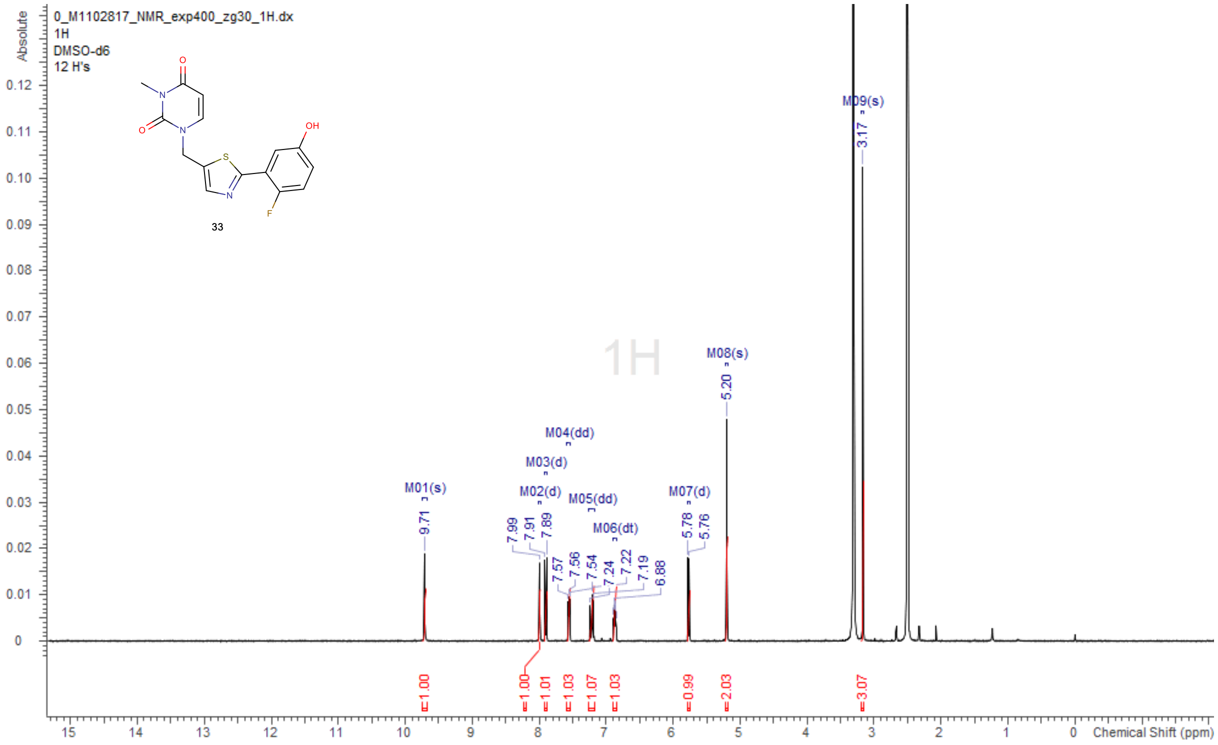

0_M1102817_NMR_exp400_zg30_1H.dx
1H
DMSO-d6
12 H's
33
M01(s)
M02(d)
M03(d)
M04(dd)
M05(dd)
M06(dt)
M07(d)
M08(s)
M09(s)
Chemical Shift (ppm)
Absolute

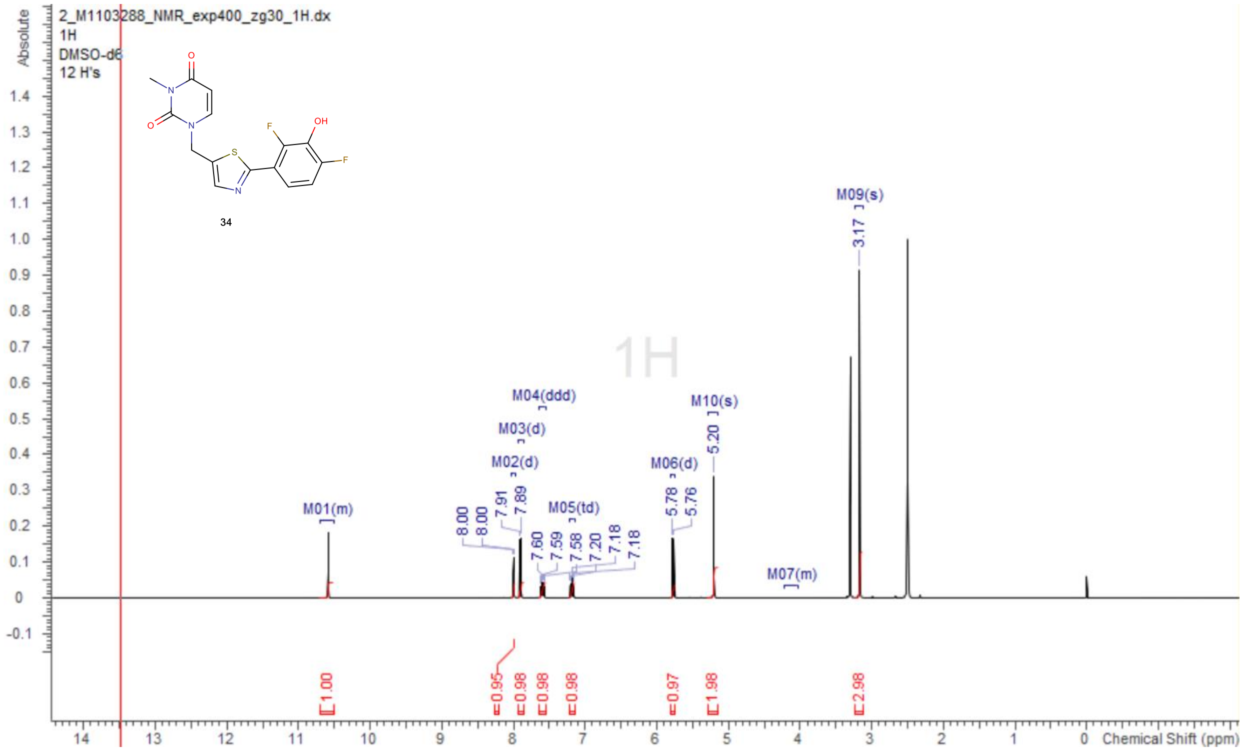

2_M1103288_NMR_exp400_zg30_1H.dx
1H
DMSO-d6
12 H's
34
M01(m)
M02(d)
M03(d)
M04(ddd)
M05(td)
M06(d)
M07(m)
M09(s)
M10(s)
Chemical Shift (ppm)
Absolute

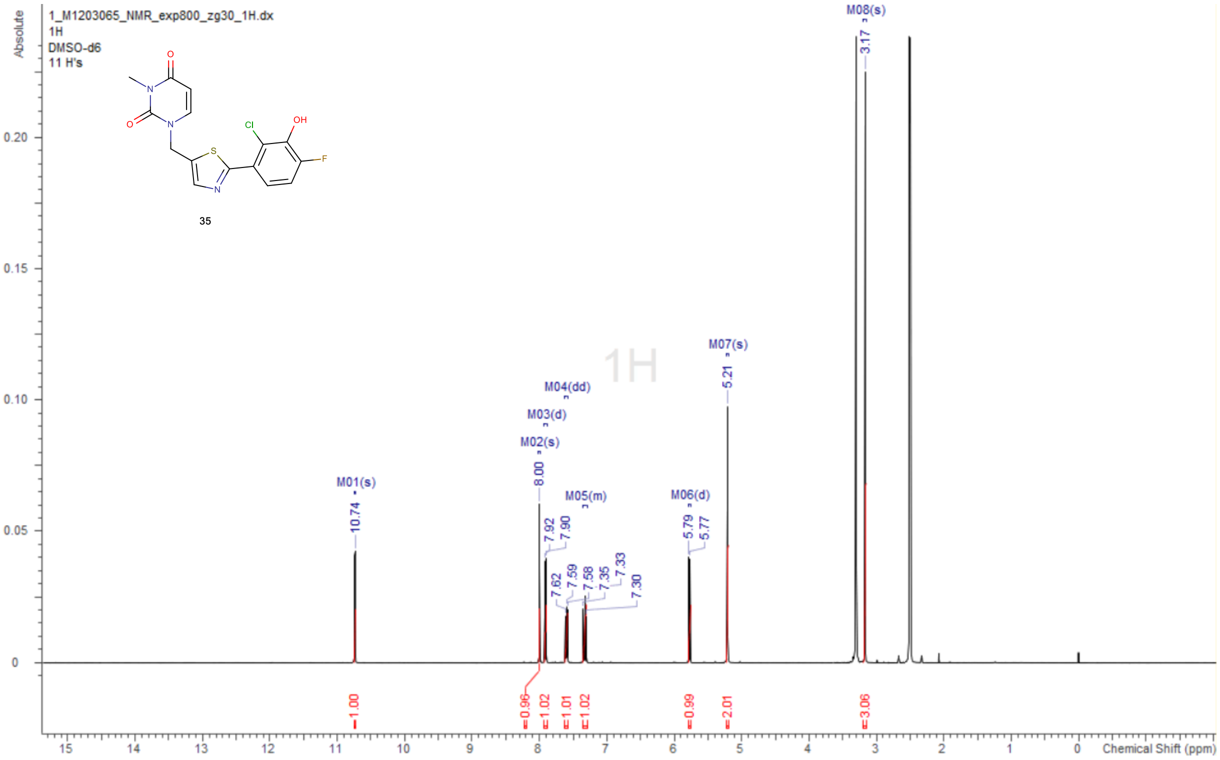
1_M1203065_NMR_exp800_zg30_1H.dx
1H
DMSO-d6
11 H's
35
M01(s) 10.74
M02(s) 8.00
M03(d) 7.92 7.90
M04(dd) 7.62 7.59 7.58
M05(m) 7.35 7.33 7.30
M06(d) 5.79 5.77
M07(s) 5.21
M08(s) 3.17
1.00 0.96 1.02 1.01 1.02 0.99 2.01 3.06
Chemical Shift (ppm)
Absolute

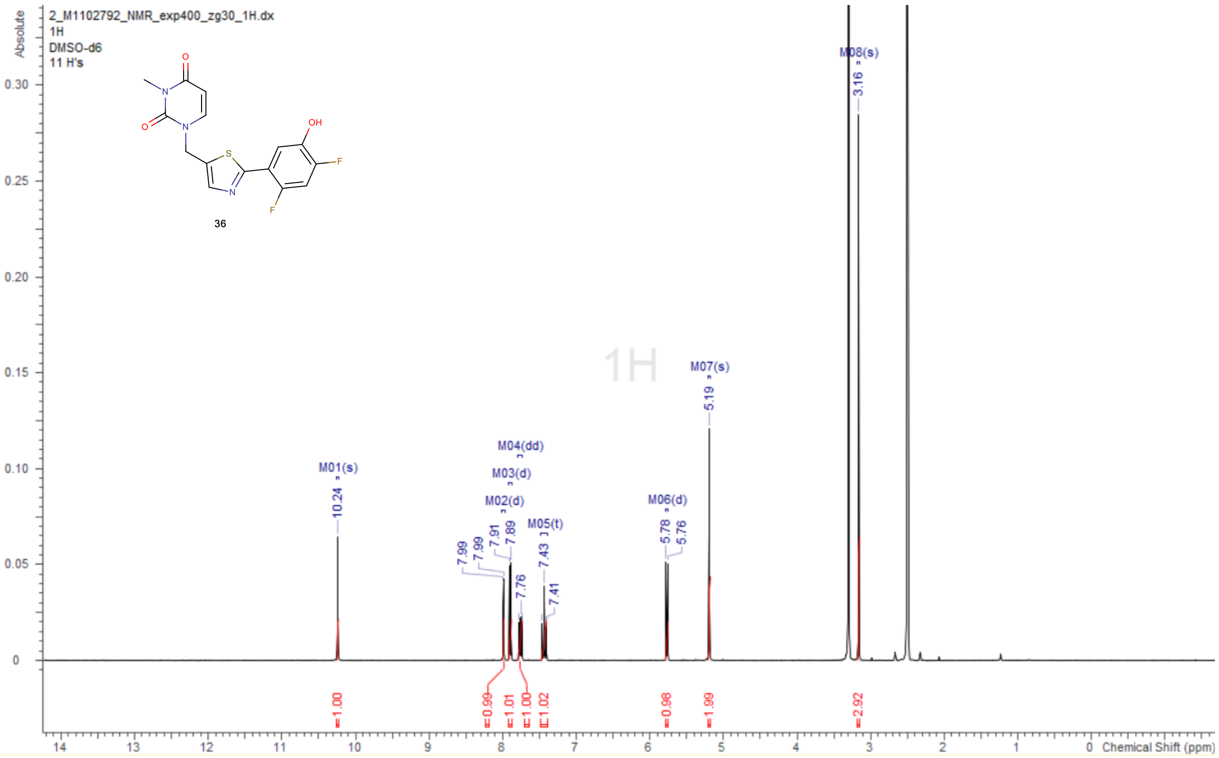
2_M1102792_NMR_exp400_zg30_1H.dx
1H
DMSO-d6
11 H's
36
M01(s) 10.24
M02(d) 7.99 7.99
M03(d) 7.91 7.89
M04(dd) 7.76
M05(t) 7.43 7.41
M06(d) 5.78 5.76
M07(s) 5.19
M08(s) 3.16
1.00 0.99 1.01 1.00 1.02 0.98 1.99 2.92
Chemical Shift (ppm)
Absolute

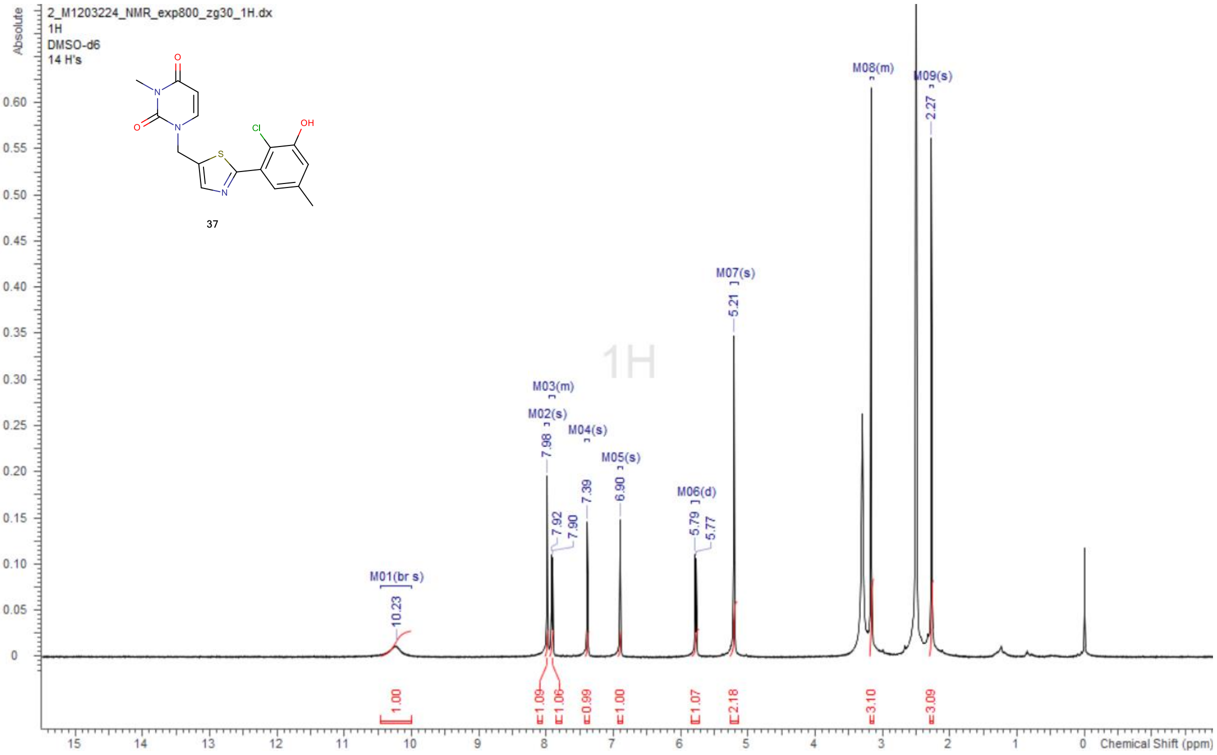
2_M1203224_NMR_exp800_zg30_1H.dx
1H
DMSO-d6
14 H's
37
Absolute
M01(br s)
10.23
M02(s)
7.98
M03(m)
7.92
7.90
M04(s)
7.39
M05(s)
6.90
M06(d)
5.79
5.77
M07(s)
5.21
M08(m)
M09(s)
2.27
1.00
1.09
1.06
0.99
1.00
1.07
2.18
3.10
3.09
Chemical Shift (ppm)

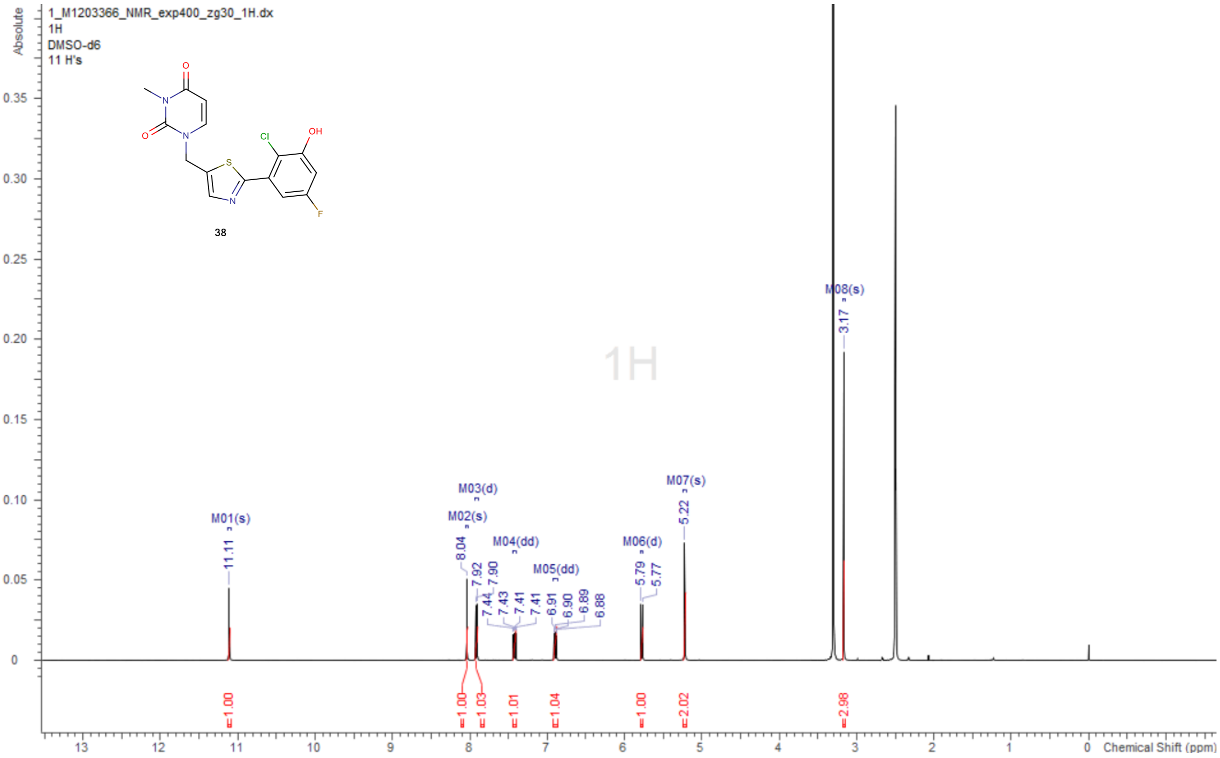
1_M1203366_NMR_exp400_zg30_1H.dx
1H
DMSO-d6
11 H's
38
Absolute
M01(s)
11.11
M02(s)
8.04
M03(d)
7.92
7.90
M04(dd)
7.44
7.43
7.41
7.41
M05(dd)
6.91
6.90
6.89
6.88
M06(d)
5.79
5.77
M07(s)
5.22
M08(s)
3.17
1.00
1.00
1.03
1.01
1.04
1.00
2.02
2.98
Chemical Shift (ppm)

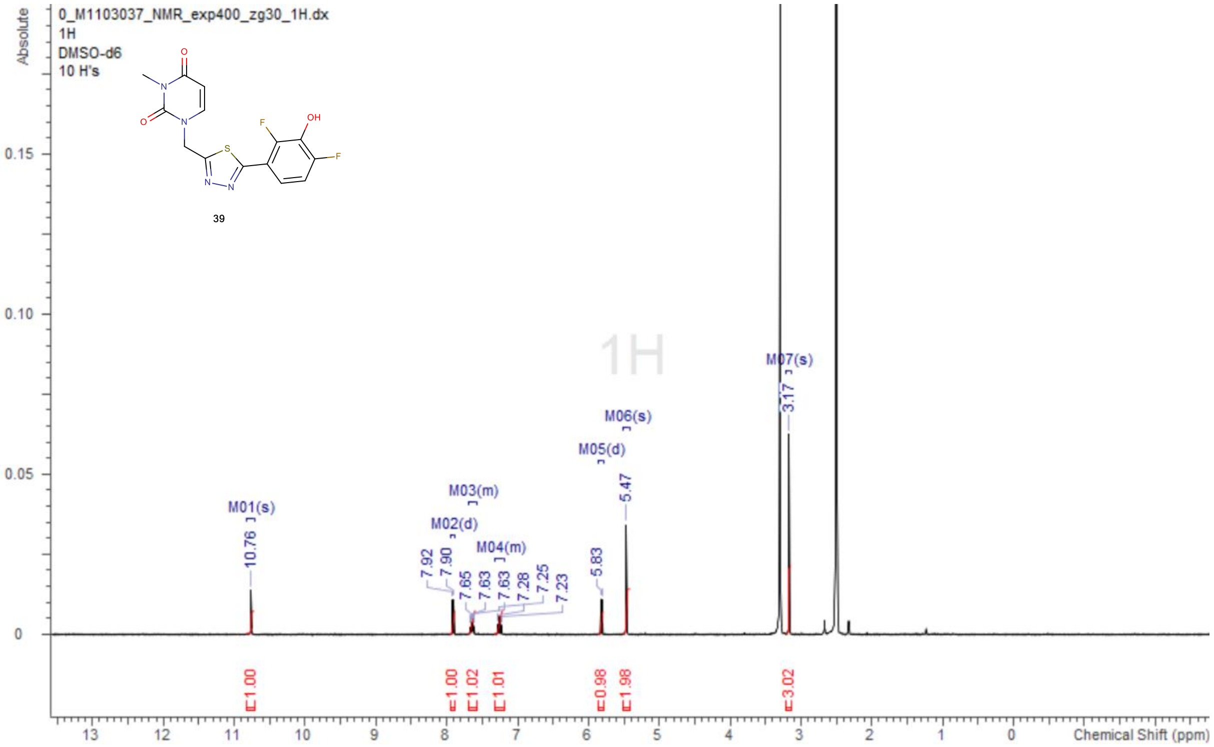
0_M1103037_NMR_exp400_zg30_1H.dx
1H
DMSO-d6
10 H's
39
M01(s)
10.76
M02(d)
7.92
7.90
M03(m)
7.65
7.63
7.63
M04(m)
7.28
7.25
7.23
M05(d)
5.83
M06(s)
5.47
M07(s)
3.17
1.00
1.00
1.02
1.01
0.98
1.98
3.02
Chemical Shift (ppm)
Absolute

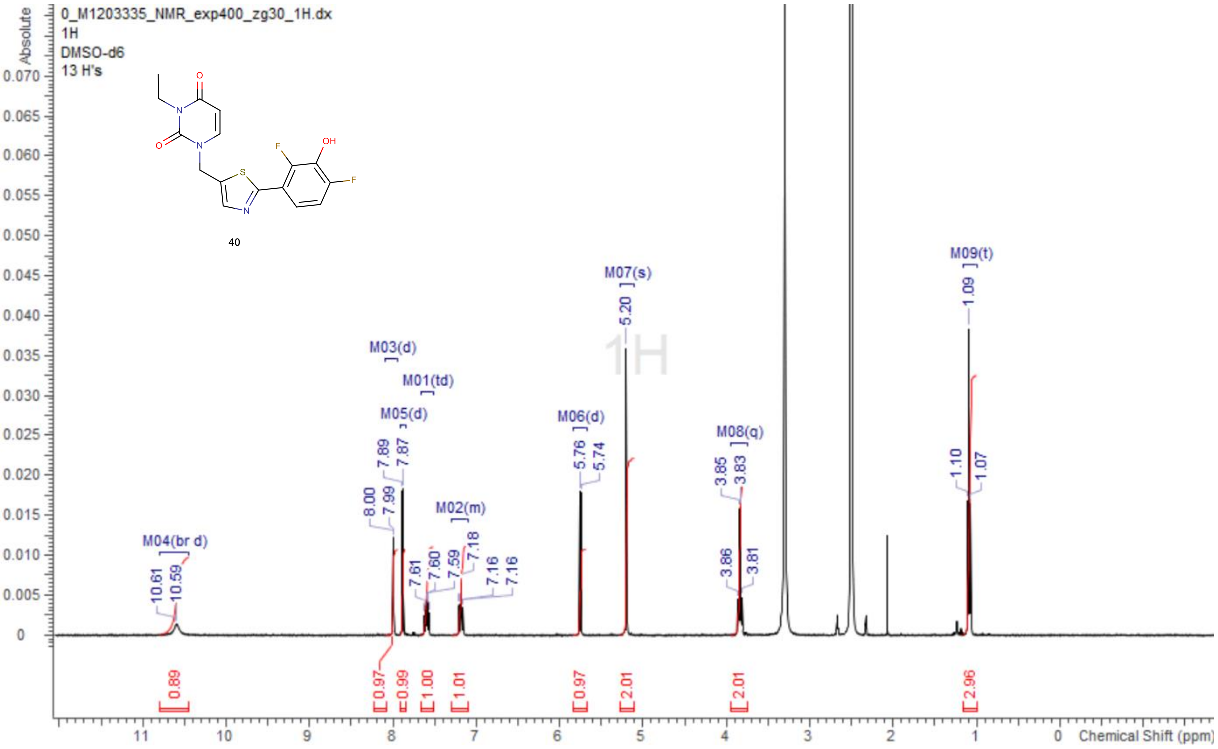
0_M1203335_NMR_exp400_zg30_1H.dx
1H
DMSO-d6
13 H's
40
M04(br d)
10.61
10.59
M03(d)
8.00
7.99
M05(d)
7.89
7.87
M01(td)
7.61
7.60
7.59
M02(m)
7.18
7.16
7.16
M06(d)
5.76
5.74
M07(s)
5.20
M08(q)
3.86
3.85
3.83
3.81
M09(t)
1.10
1.09
1.07
0.89
0.97
0.99
1.00
1.01
0.97
2.01
2.01
2.96
Chemical Shift (ppm)
Absolute

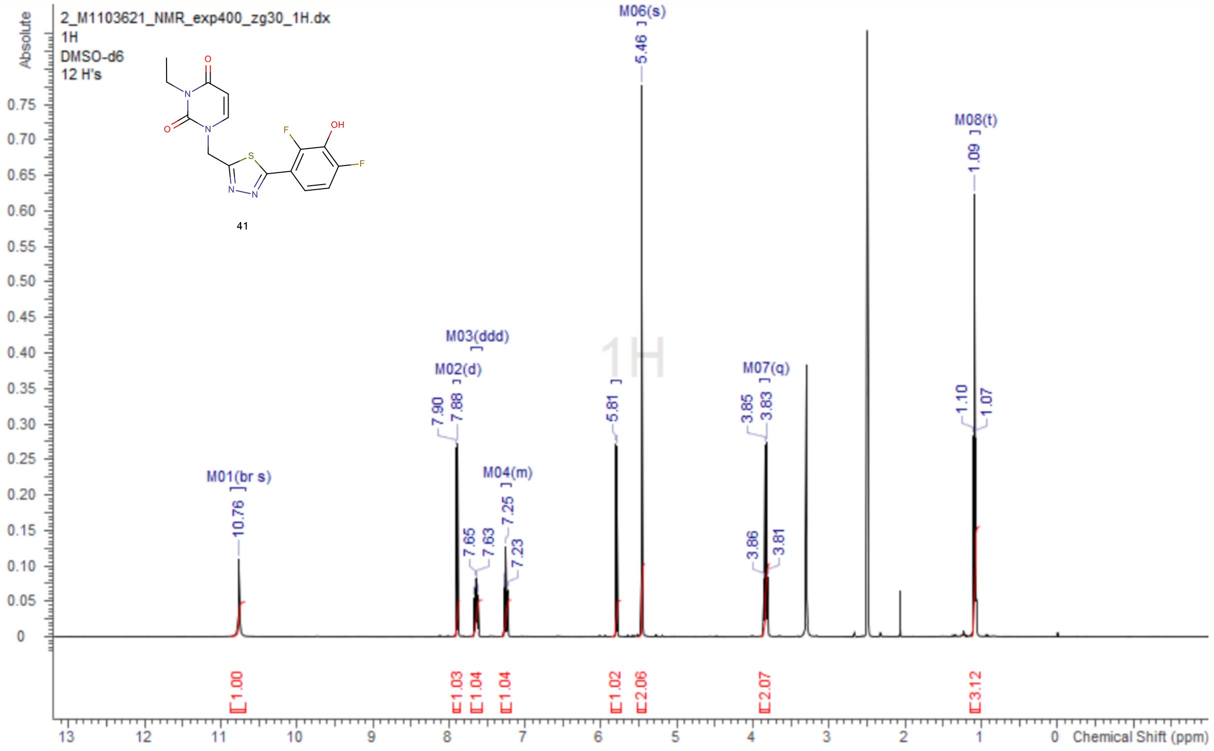
2_M1103621_NMR_exp400_zg30_1H.dx
1H
DMSO-d6
12 H's
41
Absolute
M01(br s)
10.76
M02(d)
7.90
7.88
M03(ddd)
7.65
7.63
M04(m)
7.25
7.23
5.81
M06(s)
5.46
M07(q)
3.86
3.85
3.83
3.81
M08(t)
1.10
1.09
1.07
1.00
1.03
1.04
1.04
1.02
2.06
2.07
3.12
Chemical Shift (ppm)

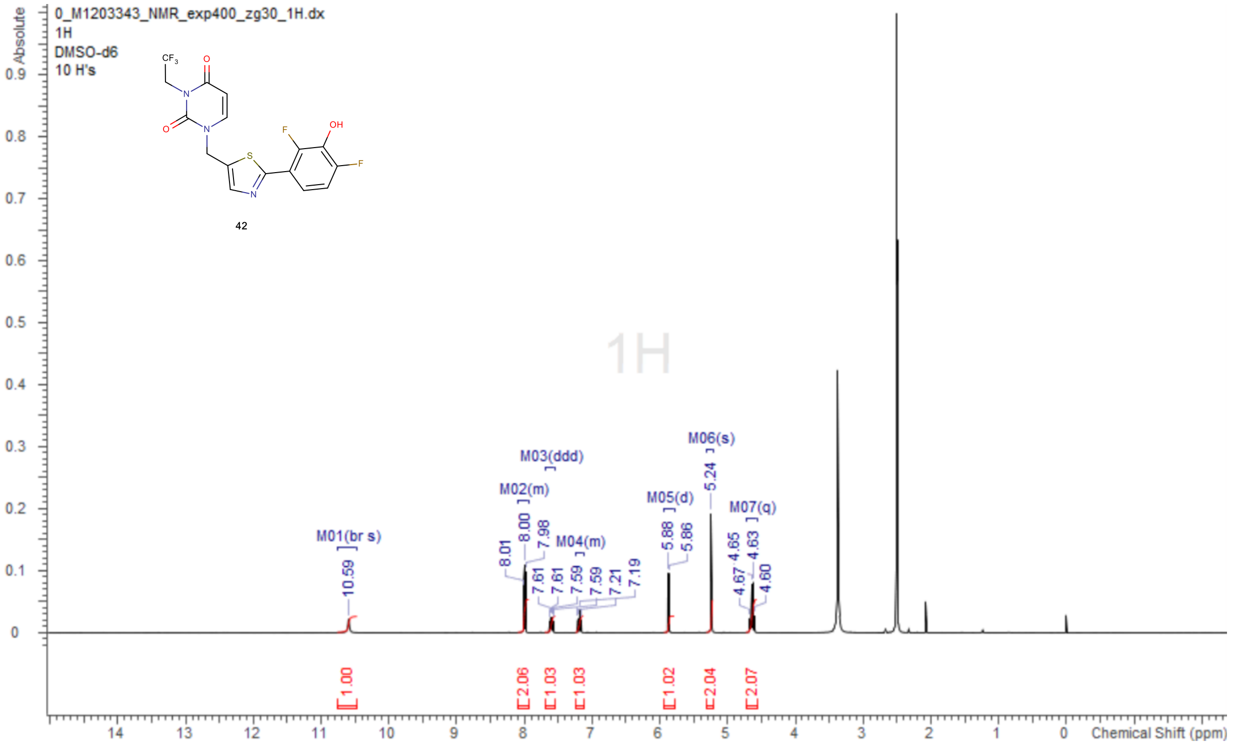
0_M1203343_NMR_exp400_zg30_1H.dx
1H
DMSO-d6
10 H's
42
Absolute
M01(br s)
10.59
M02(m)
8.01
8.00
7.98
M03(ddd)
7.61
7.61
7.59
7.59
M04(m)
7.21
7.19
M05(d)
5.88
5.86
M06(s)
5.24
M07(q)
4.67
4.65
4.63
4.60
1.00
2.06
1.03
1.03
1.02
2.04
2.07
Chemical Shift (ppm)

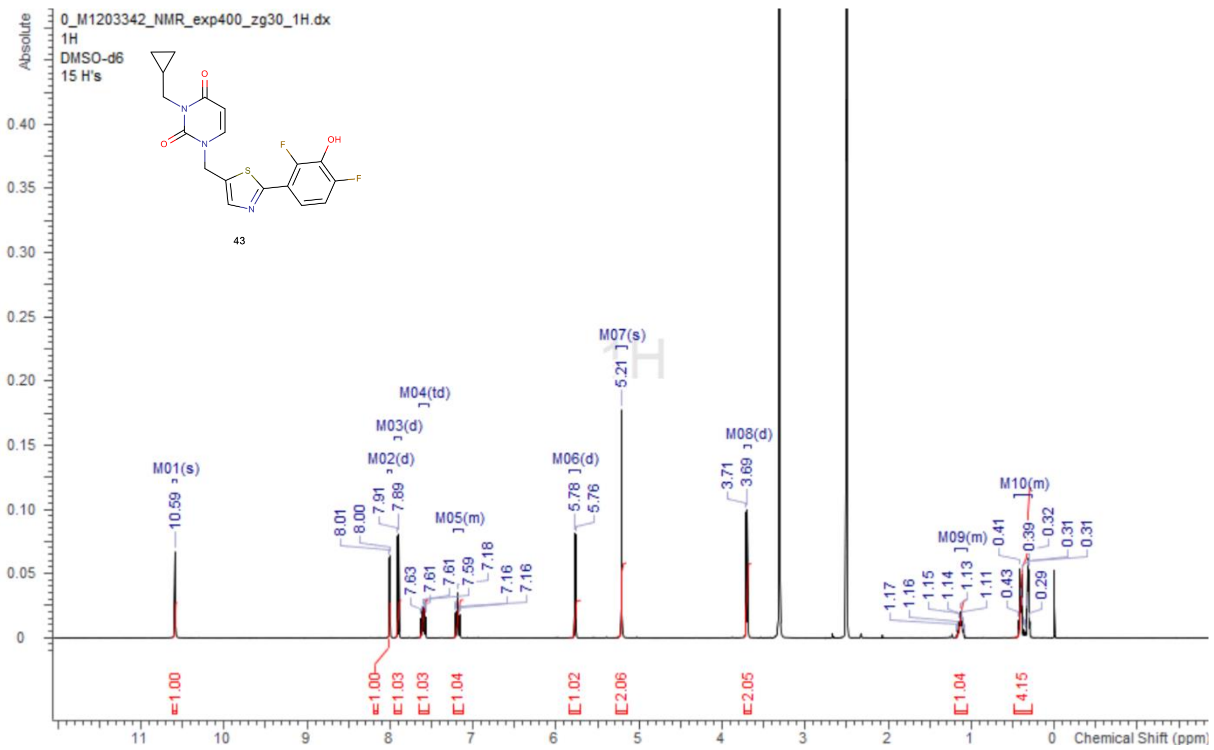
0_M1203342_NMR_exp400_zg30_1H.dx
1H
DMSO-d6
15 H's
43
Absolute
Chemical Shift (ppm)
M01(s)
M02(d)
M03(d)
M04(td)
M05(m)
M06(d)
M07(s)
M08(d)
M09(m)
M10(m)

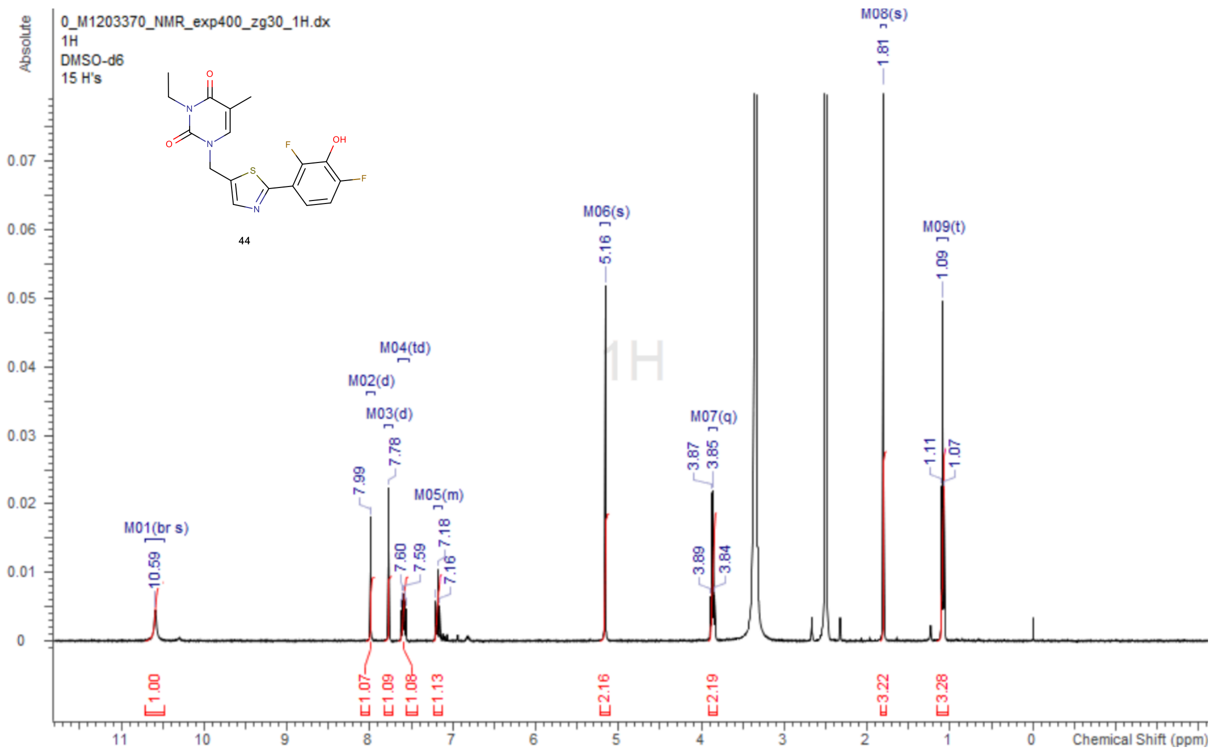
0_M1203370_NMR_exp400_zg30_1H.dx
1H
DMSO-d6
15 H's
44
Absolute
Chemical Shift (ppm)
M01(br s)
M02(d)
M03(d)
M04(td)
M05(m)
M06(s)
M07(q)
M08(s)
M09(t)

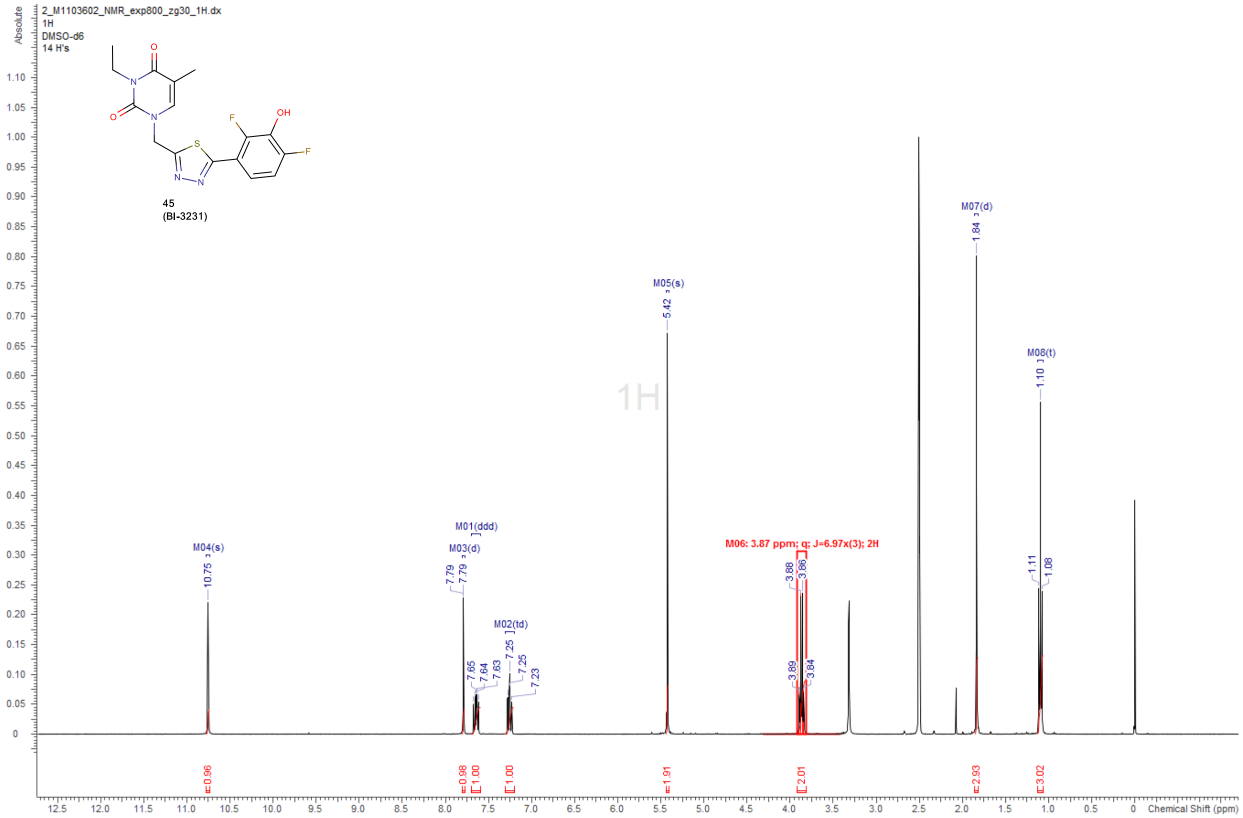

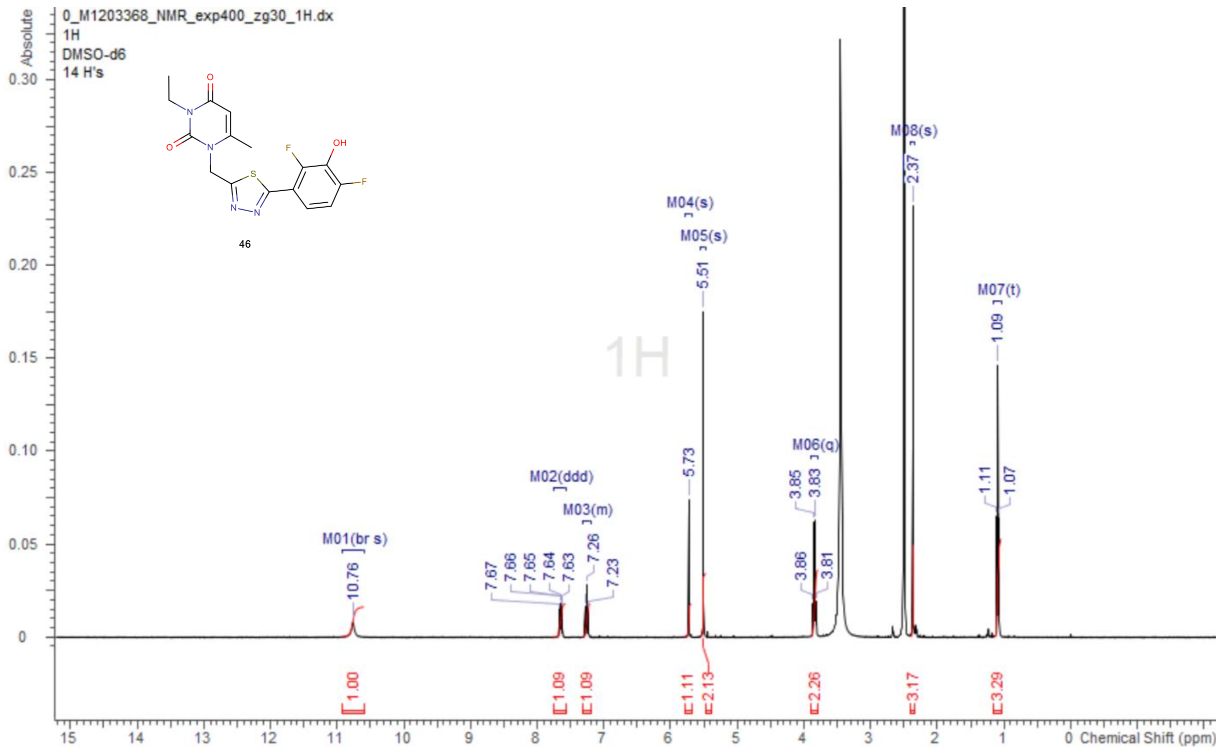

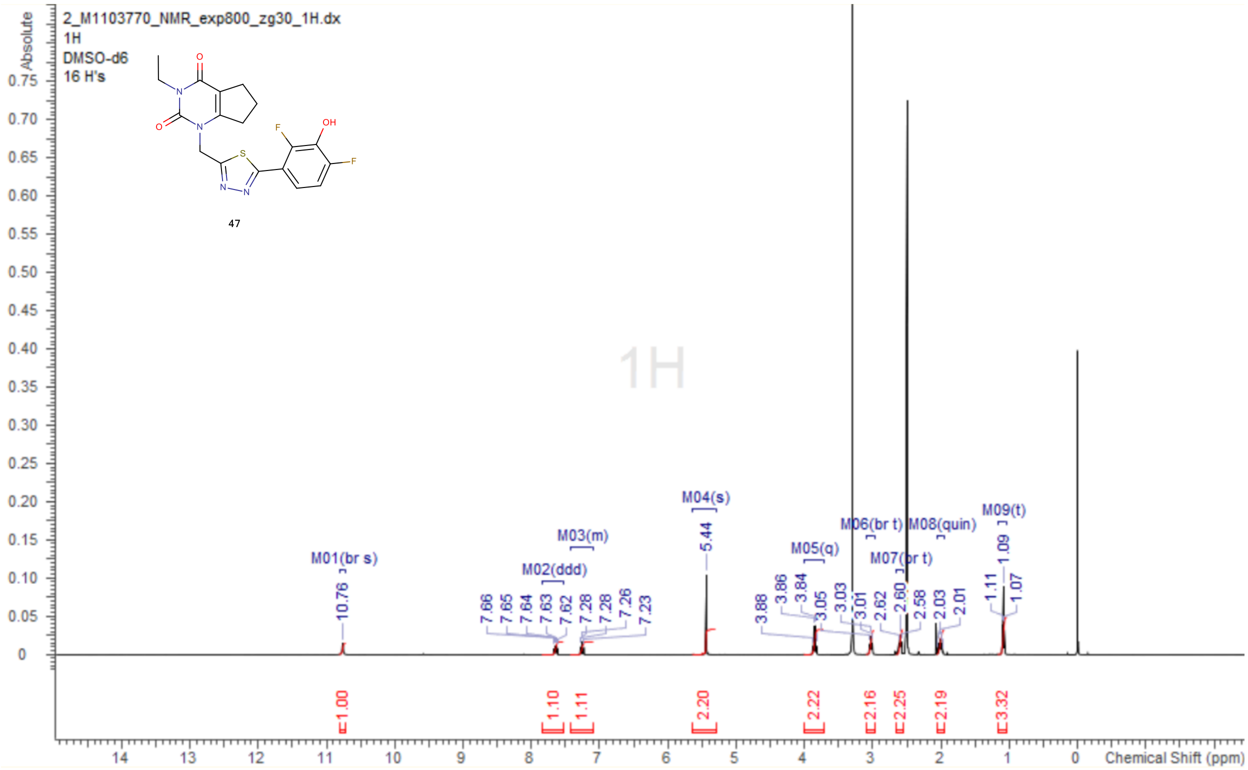

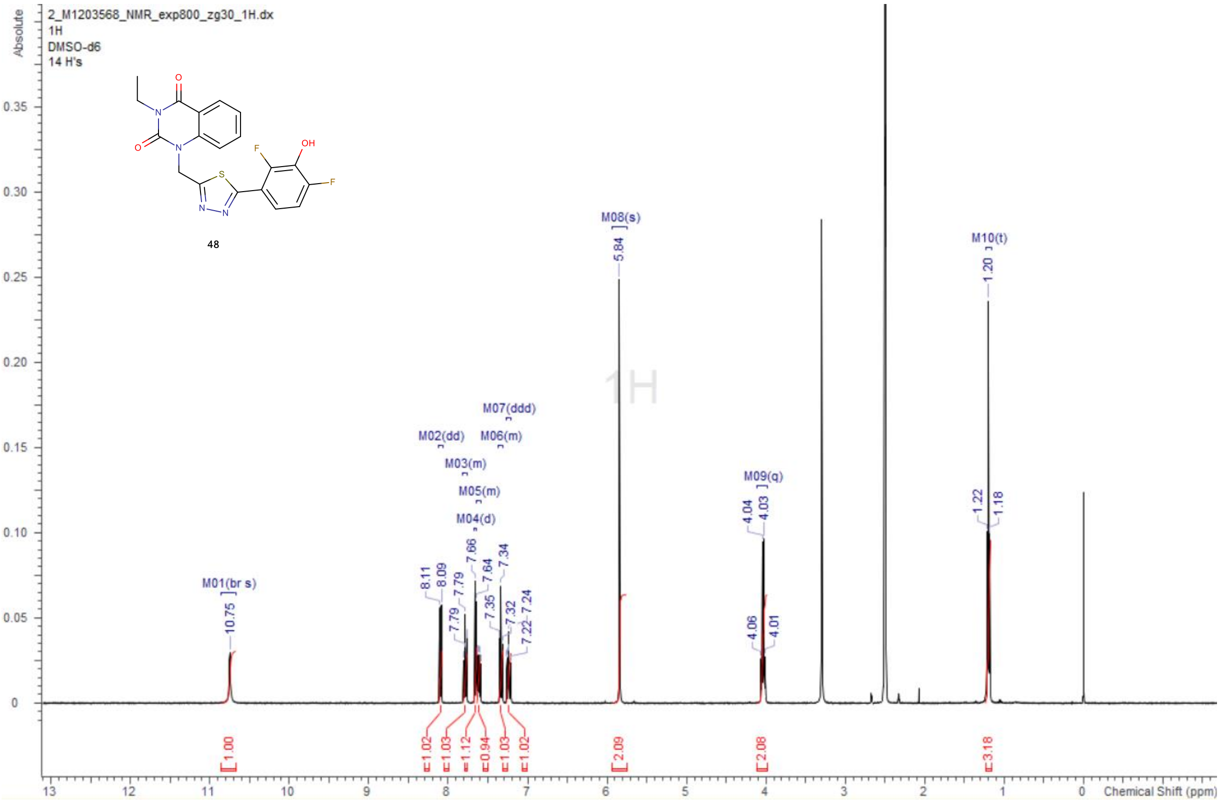

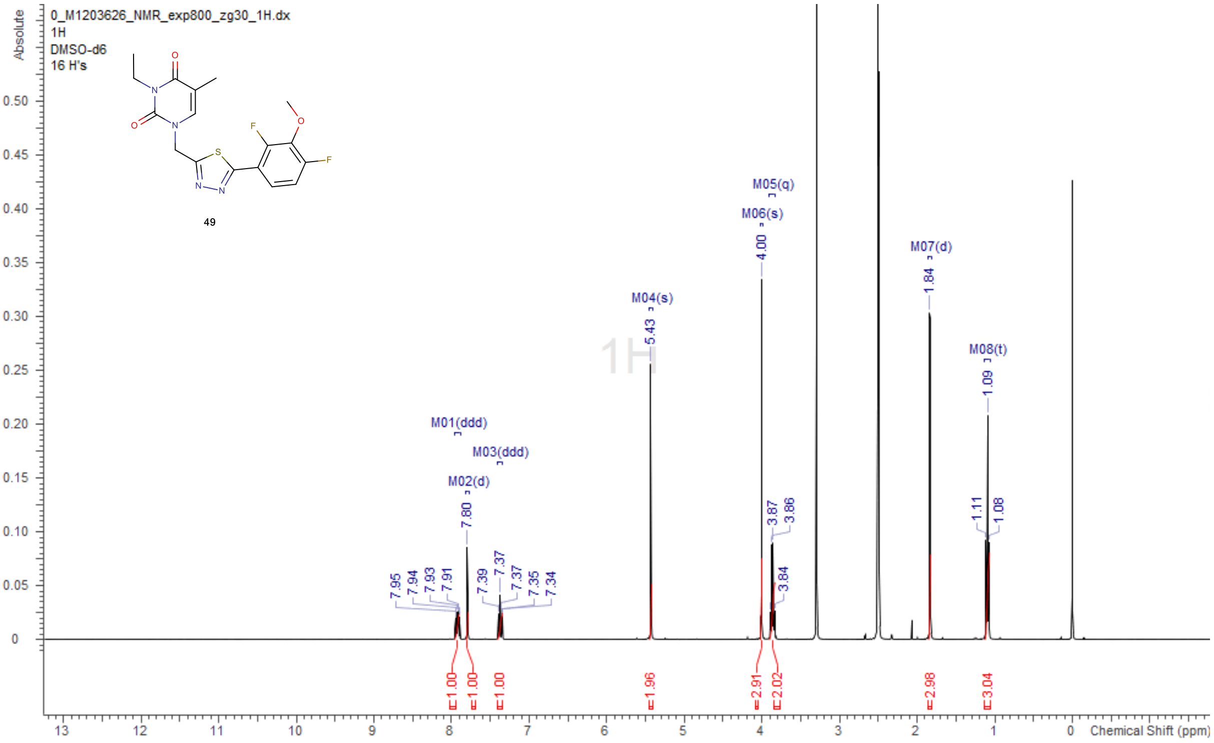

0_M1203626_NMR_exp800_zg30_1H.dx
1H
DMSO-d6
16 H's
49
Absolute
M01(ddd)
M02(d)
M03(ddd)
M04(s)
M05(q)
M06(s)
M07(d)
M08(t)
Chemical Shift (ppm)
